# Adenomyosis and association between ultrasonographic features and pregnancy outcomes in women with infertility or pregnancy loss: a systematic review and meta-analysis

**DOI:** 10.1016/j.ebiom.2026.106518

**Published:** 2026-10-21

**Authors:** Nicolaj Bruun Brandt, Cecilia Lindner, Karen Høgh Abrahamsen, Giuliana Beneduce, Sofie Bliddal, David Westergaard, Steen Joop Bonnema, Marianne Skovsager Andersen, Pernille Ravn, Dorte Glintborg, Henriette Svarre Nielsen

**Affiliations:** a Department of Gynecology and Obstetrics, Odense University Hospital, Denmark; b Department of Health, University of Southern Denmark, Denmark; c Department of Endocrinology, Odense University Hospital, Denmark; d Department of Obstetrics and Gynecology, Copenhagen University Hospital Hvidovre, DK-2650, Denmark; e Department of Clinical Medicine, University of Copenhagen, Denmark; f Department of Women's, Children's and Public Health Sciences, Università Cattolica del Sacro Cuore, Rome, Italy; g Department of Health Technology, Technical University of Denmark, Lyngby, Denmark

**Keywords:** Adenomyosis, Infertility, In vitro fertilization, Pregnancy loss, MUSA

## Abstract

**Background:**

Adenomyosis has been shown to reduce live birth rates and increase the risk of pregnancy loss. The diagnosis is often established by ultrasonographic assessment using the revised Morphological Uterus Sonographic Assessment (MUSA) criteria. However, the reproductive impact of specific sonographic features remains unclear. A systematic review and meta-analysis was conducted to investigate whether individual ultrasonographic adenomyosis features influence pregnancy outcomes in women with infertility or pregnancy loss.

**Methods:**

We searched MEDLINE (PubMed), Embase, Cochrane Library (CENTRAL), Web of Science, Scopus, and Google Scholar (most recent 200 records) from inception to May 11th, 2026. Observational and interventional studies reporting specific ultrasonographic features of adenomyosis in women with infertility and/or pregnancy loss were included. Primary outcomes were clinical pregnancy, live birth, and pregnancy loss. Two reviewers independently extracted data and assessed risk of bias using the Newcastle–Ottawa Scale and Joanna Briggs Institute critical appraisal tools, as appropriate by study design. Meta-analyses were performed using random-effects models, with common-effects models applied where appropriate; remaining evidence was synthesised narratively. Certainty of evidence was assessed by GRADE. The protocol was registered on PROSPERO (CRD420251077005).

**Findings:**

Of 2322 records screened, 29 studies comprising 19,510 patients (7569 with adenomyosis) were included. Diffuse adenomyosis was associated with lower odds of live birth compared with no adenomyosis (OR 0.52, 95% CI 0.34–0.79; I^2^ = 63%), as well as internal adenomyosis compared with no adenomyosis (OR 0.49 95% CI 0.42–0.57 I^2^ = 0%). Narrative synthesis suggested poorer reproductive outcomes with adenomyosis features located near the endometrium and with larger uterine volume. The overall certainty was low or very low for 20 out of 21 outcomes, and moderate for one, primarily driven by imprecision and indirectness related to variation in diagnostic criteria across studies.

**Interpretation:**

Reproductive outcomes in adenomyosis vary according to specific ultrasonographic features, supporting feature-based reporting beyond a binary diagnosis. Substantial clinical and methodological heterogeneity, including inconsistent ultrasonographic diagnostic criteria, variable application of MUSA definitions, and differences in fertility treatment protocols, warrants cautious interpretation of pooled estimates. Standardised definitions and reporting of adenomyosis features are needed to improve evidence synthesis and clinical translation.

**Funding:**

The Novo Nordisk Foundation and the Danish Research Council.

**Research in context**

**Evidence before this study**

Adenomyosis is often classified simply as either present or absent; however, recent studies have described detailed ultrasonographic characteristics of the disease, allowing for subtype classification. Although the association between adenomyosis and adverse reproductive outcomes is established, no comprehensive synthesis of the literature has yet evaluated whether specific features of adenomyosis confer greater detrimental effects than others.

A systematic search on MEDLINE (PubMed), Embase, Cochrane Library (CENTRAL), Web of Science, Scopus, and the 200 most recent publications on Google Scholar was conducted on the 11th of May 2026. Studies on patients with infertility and/or pregnancy loss that reported on specific ultrasonographic adenomyosis features were included. Studies were excluded if they did not report specific adenomyosis features, if only magnetic resonance imaging was used or if they were in a non-Latin language. A search block was built based on the terms adenomyosis, ultrasound and pregnancy outcomes with appropriate MeSH terms. The overall risk of bias was moderate to low in the included studies that reported on pregnancy outcomes.

**Added value of this study**

The findings suggest that patients with diffuse adenomyosis, lesions located close to the endometrium, or enlarged uterine volumes have poorer pregnancy prognoses compared with those who have focal or peripherally located adenomyosis or smaller uterine volumes. This study synthesises all available data on the prevalence of various adenomyosis features, revealing substantial heterogeneity across studies.

**Implications of all the available evidence**

Based on available literature, adenomyosis should be regarded as a spectrum of features associated with differing risks of infertility and pregnancy loss. We encourage the publication of comprehensive data on all adenomyosis features to facilitate more nuanced research and clinical interpretation.

## Introduction

Adenomyosis was first described in 1860, and is the benign invasion of endometrial glands into the myometrium.^1^ Adenomyosis was initially diagnosed in women through histological examination of hysterectomy specimens performed for menorrhagia. Over the past two decades, technological advances in transvaginal ultrasound have enabled swift evaluation of adenomyosis in a clinical outpatient setting in women with infertility and/or recurrent pregnancy loss (RPL).^2^

While histopathological examination after hysterectomy remains the gold standard for definitive diagnosis, non-invasive imaging modalities such as transvaginal ultrasound is now considered a reliable alternative for diagnosis and assessment of adenomyosis. In 2015, van den Bosch et al.^3^ conducted an international consensus study on how to diagnose adenomyosis with ultrasonography. The consensus was revised in 2022 by Harmsen et al.,^4^ resulting in the revised Morphological Uterus Sonographic Assessment (MUSA) criteria, thereby streamlining the adenomyosis diagnostic criteria and facilitating comparability across studies.

Recent systematic reviews and meta-analyses have shown that patients with adenomyosis have significantly lower odds of clinical pregnancy and live birth, and higher odds of pregnancy loss compared with controls.^5^, ^6^ However, it remains unclear whether specific ultrasonographic features of adenomyosis are more detrimental than others, as the condition is often treated as a single disease entity rather than a spectrum of distinct features. Moreover, studies focussing on individual adenomyosis features frequently report inconsistent findings, largely due to variability in the criteria used to define the presence and subtypes of adenomyosis.^3^

According to the revised MUSA criteria, the ultrasonographic features of adenomyosis can be categorised by type (including three direct and six indirect features), by distribution (focal or diffuse), and by the location of myometrial involvement (inner or outer myometrium).^4^ There is no consensus on how adenomyosis disease severity is defined, though many studies use the number of features as a way to express severity. Prior studies suggest that involvement of the inner myometrium (junctional zone) and the presence of certain adenomyosis features (interrupted junctional zone, diffuse involvement, and larger uterine volume) are more strongly linked to poorer reproductive outcomes, whereas other features appear to have less impact.^7^, ^8^, ^9^, ^10^ Increased uterine volume is not an adenomyosis feature per se but is a widely acknowledged consequence of the condition.

The primary aim of this review is to determine which specific ultrasonographic features of adenomyosis are associated with clinical pregnancy rate, live birth rate and pregnancy loss rate in women with infertility or RPL. The secondary aim is to summarise the prevalence of MUSA-defined features in patients with infertility or pregnancy loss.

## Methods

A systematic review with meta-analysis was conducted. A protocol was developed prior to the literature search and registered in PROSPERO (CRD420251077005) and is publicly available through the PROSPERO database. The review was conducted and reported in accordance with the PRISMA 2020 statement. After registration, several methodological refinements were made. Studies including women with co-existing endometriosis were retained when data specific to adenomyosis could not be separated, as such data were rarely reported separately. The exposure definition was expanded to include additional ultrasonographic indicators, including uterine volume and severity of disease. Planned risk-of-bias assessment using RoB-2 was replaced with the Joanna Briggs Institute (JBI) tool to better accommodate included study designs.

**Population**: Women with infertility or pregnancy loss.

**Exposure**: Ultrasound examination describing presence of adenomyosis features: MUSA features, focal/diffuse, question mark sign, inner/outer myometrial involvement, adenomyoma, uterine volume.

**Comparator**: Women with infertility or pregnancy loss without ultrasonographic adenomyosis features.

**Outcomes**: Biochemical pregnancy rate, clinical pregnancy rate, (ultrasonographically confirmed), live birth rate and pregnancy loss rate.

**Study design**: Prospective and retrospective cohort studies and cross-sectional prevalence studies reporting ultrasonographic adenomyosis features in women with infertility or pregnancy loss were eligible for inclusion.

Inclusion criteria were: Original peer-reviewed studies published from database inception to May 11th, 2026.

Exclusion criteria were: Studies that did not report on specific ultrasound features in diagnosing adenomyosis, studies that did not report on the specified outcome, studies that only used magnetic resonance imaging (MRI), studies not published in languages using the Latin alphabet, reviews, opinion papers and if only abstract was available. Case-control studies and RCTs were not considered in regards to prevalence estimates.

### Search strategy

Three search blocks were combined. The first block contained infertility/pregnancy loss, the second adenomyosis, and the third diagnostic criteria: ultrasonography. Search terms combined concepts related to infertility or pregnancy loss, adenomyosis, and ultrasonography using controlled vocabulary (e.g., MeSH terms) and free-text terms. The full search strategy for each database is provided in Supplementary Table A. The following databases were used: MEDLINE (PubMed), Embase, Cochrane Library (CENTRAL), Web of Science, Scopus, and the 200 most recent publications on Google Scholar, to identify recently published studies not yet indexed in other databases.

Meta-analyses and literature reviews were used as background information to make sure that relevant previous studies were included.

Clinical trial protocols identified through Embase were reviewed to assess potential publication bias. Conference abstracts found in the search were reviewed, to see if they were published or not.

### Study selection procedures

Screening: Two independent reviewers screened titles and abstracts, followed by full-text assessment using pre-piloted eligibility forms; disagreements were resolved by consensus, or a third reviewer.

### Data extraction and management

Two reviewers independently extracted all data using a standardised form in Covidence, and discrepancies were resolved by discussion. The following data were extracted: study design, country, patient characteristics (age and infertility diagnosis), presence of coexisting endometriosis, sonographic modality and operator characteristics (2D vs 3D ultrasound and use of MUSA definitions), specific sonographic features reported and their definitions, crude and adjusted effect estimates, confounders adjusted for, follow-up duration, and missing data.

When information required for data extraction was missing or unclear, the corresponding study authors were contacted to request clarification or additional data. Specifically, we contacted the authors of the studies by Cozzolino et al.,^11^ Alson et al.,^10^, ^12^ Han SJ et al.,^13^ Benlioğlu et al.,^14^ and Dason et al.^15^ If the requested information was unavailable, the data were recorded as not available (N/A), and no assumptions or imputations were made.

The primary outcomes were clinical pregnancy, pregnancy loss, and live birth, as defined by the original study authors. Data were extracted for all reported effect measures, including crude prevalence and adjusted odds ratios (ORs), where available. When multiple effect estimates were reported, the most fully adjusted estimate was extracted.

In instances where pooling was not appropriate due to clinical and methodological heterogeneity, or insufficient comparable data were available, findings were synthesised narratively by grouping studies according to the adenomyosis features they reported. Following data extraction, reported adenomyosis characteristics were grouped into conceptual categories (MUSA-defined features, anatomical location, type, volume, and severity). Within each category, crude and adjusted effect estimates were summarised descriptively. The aim was to characterise the range and reporting of associations rather than to pool effect estimates statistically.

Study characteristics and extracted data were summarised in structured tables. Results of quantitative syntheses were presented using forest plots, and qualitative findings were summarised descriptively.

### Risk of bias assessment and certainty of evidence

Risk of bias (RoB) was assessed using the Newcastle–Ottawa Scale (NOS) for cohort studies^16^ reporting on pregnancy outcome and the JBI checklist^17^ for studies reporting on prevalence of adenomyosis features. Two reviewers independently assessed RoB, and discrepancies were resolved by discussion. The NOS evaluates three domains: participant selection, group comparability, and outcome ascertainment, with a maximum score of nine. As only crude ORs were extracted for the meta-analysis, the comparability domain was assessed based on similarity in baseline characteristics rather than statistical adjustment. One star was awarded for comparability in age and an additional star for comparability in BMI. For prognostic studies including only women with adenomyosis, exposure status was defined according to the ultrasound feature or phenotype under investigation. Studies scoring 7–9 were rated as low, 5–6 as moderate, and <5 as high RoB. The JBI checklist assesses sampling, methodology, analysis, and response rate (score range 0–9). The JBI item assessing coverage of the identified sample was deemed not applicable, as included studies focused on predefined subgroups rather than population-based samples. This item was therefore marked as *N/A* for all studies, yielding a maximum possible JBI score of eight. Studies scoring 7–8 points were considered to have a low, 4–6 points a moderate, and 0–3 points a high RoB.

Certainty of evidence for reproductive outcomes was assessed using the GRADE approach adapted for prognostic factor research.^18^ The certainty of evidence was evaluated across the domains of risk of bias, inconsistency, indirectness, imprecision, and publication bias. Observational studies were evaluated using NOS prior to GRADE assessment.

For prevalence outcomes, certainty assessment using GRADE was not performed because the framework is not established for prevalence estimates; instead, methodological quality was assessed using the JBI critical appraisal checklist for prevalence studies.

### Publication bias

Funnel plots were generated for all meta-analysed comparisons and prevalence outcomes to assess small-study effects. As the majority of outcomes included less than the 10 studies recommended for adequately powered formal testing, funnel plots were displayed for visual assessment only.

### Ethics

No ethical permission was needed as this study is a literature study on publicly available data.

### Statistics

Prior to synthesis, studies were grouped according to the type of data reported. Studies reporting prevalence of specific ultrasonographic adenomyosis features were eligible for prevalence meta-analyses, while studies reporting associations between adenomyosis features and reproductive outcomes with sufficient event data were eligible for effect-estimate meta-analyses. Some studies contributed to both synthesis types if they reported both prevalence and outcome association data. Eligibility for pooling within each synthesis was determined by comparing outcome definitions, ultrasonographic diagnostic criteria and feature definitions, and availability of comparable data. Studies lacking sufficient numerical data to derive effect estimates were excluded from quantitative synthesis and included in narrative synthesis; no imputation was performed.

Effect measures were odds ratios (ORs) with 95% confidence intervals (CIs) for associations between adenomyosis features and reproductive outcomes. For prevalence analyses, pooled proportions were calculated.

Meta-analyses were based on crude event data extracted from individual studies, from which pooled ORs were calculated rather than combining reported adjusted estimates. Adjusted estimates were summarised narratively due to heterogeneity in confounder adjustment across studies.

Random-effects meta-analyses were performed when at least two studies reported comparable outcome definitions and sufficient data for pooling, using the Mantel-Haenszel method with the DerSimonian-Laird estimator for between-study variance (τ^2^). The Hartung-Knapp adjustment was applied to random-effects confidence intervals in meta-analysis of reproductive outcomes.

For prevalence estimates, analyses were restricted to studies reporting MUSA-defined features, and generalised linear mixed models (GLMM) with a logit link function and maximum-likelihood estimation were used. Between-study heterogeneity was assessed using the I^2^ statistic and Cochran's Q test. I^2^ values were interpreted as low (<25%), moderate (25–75%), and high (>75%) heterogeneity.

Possible sources of heterogeneity among study results were explored using subgroup analyses by study design (prospective vs retrospective), with subgroup differences evaluated using a Q-test for heterogeneity between subgroups. Sensitivity analyses were conducted to assess the robustness of pooled estimates and included leave-one-out analyses and exclusion of studies with high risk of bias. Meta-regression was not performed, as fewer than ten studies contributed to each comparison and candidate moderators were confounded with one another and inconsistently reported.

All analyses used R version 4.4.3 with the meta package (version 8.2–1). Two-tailed p-values <0.05 were considered statistically significant.

### Role of funders

The funder of the study had no role in study design, data collection, data analysis, data interpretation, or writing of the report.

## Results

The systematic search identified 2322 records. After screening and eligibility assessment, 29 studies met the inclusion criteria and were included in the qualitative synthesis (Fig. 1, PRISMA flowchart). Among the studies, 16 were retrospective, 12 were prospective, and one was cross-sectional (Table 1). Twenty-two studies reported pregnancy outcomes (Supplementary Table B), and 14 studies described the prevalence of individual adenomyosis features.

**Fig. 1:**
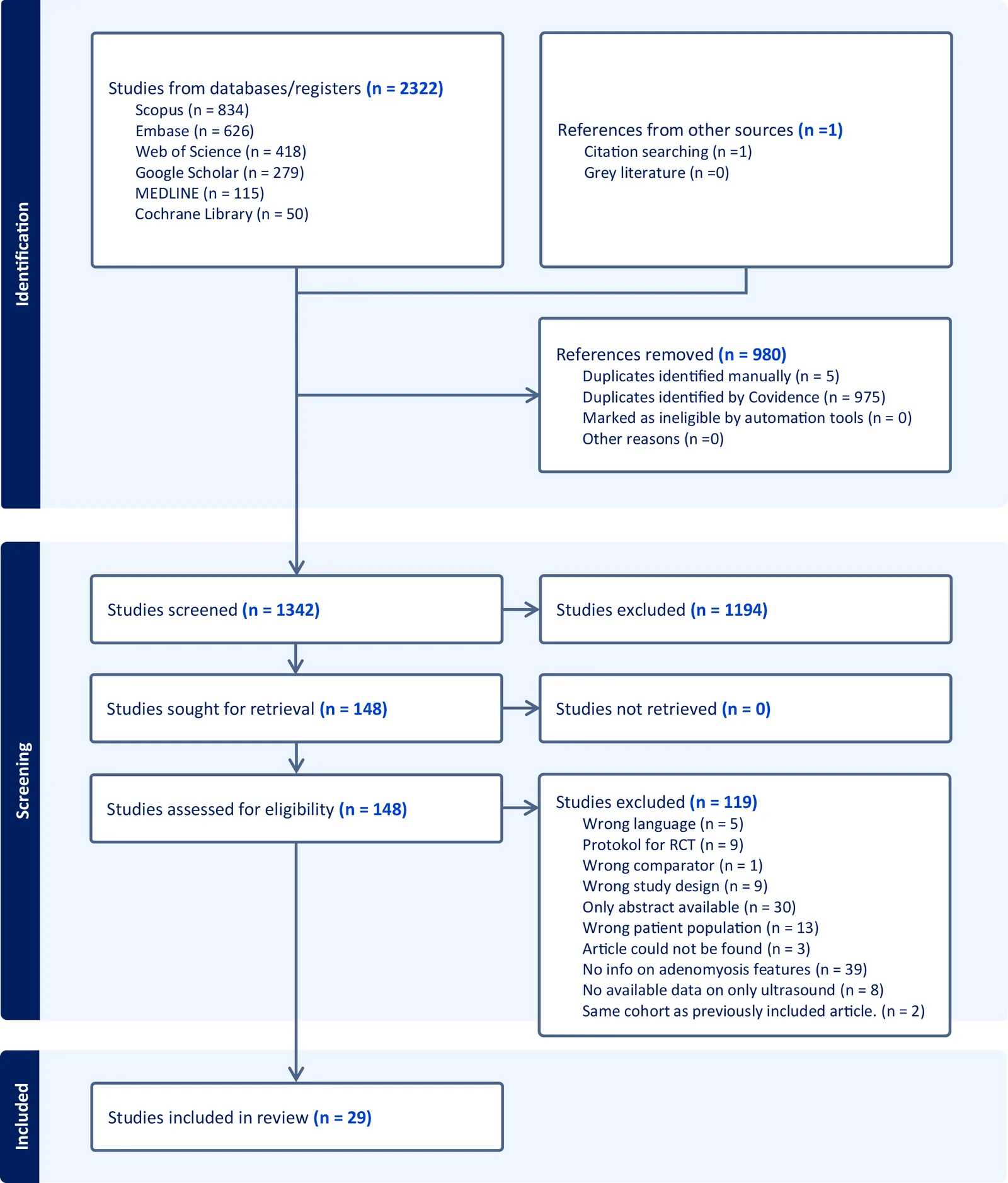
PRISMA flowchart.

**Table 1:** Overview of included studies.

Author (year)	Country	Study design	Patient description	Exclusion criteria	US type	Diagnostic criteria	n-adenomyosis/n-total (%)	Endometriosis n (% of n-adenomyosis)
Sharma et al. (2026)^19^	India	Single-centre prospective cohort study	Infertile women <40 years old with normal ovarian reserve, with exclusively diffuse adenomyosis or with male infertility without adenomyosis	Women with focal adenomyosis, fibroids, hydrosalpinx, uterine size >12 weeks, congenital anomalies, history of adenomyosis surgery, or AMH <1.1 ng/mL	2D-TVUS	≥2 MUSA criteria	368/585 (62.9)	NR
Zhang et al. (2023)^9^	China	Single-centre retrospective cohort study	Infertile women ≤45 years old with adenomyosis with regular menstrual cycle who underwent IVF-ET with adenomyosis	Women with intrauterine adhesions, uterine malformations, submucosal leiomyomas, or ≥5.0 cm in diameter leiomyoma; hydrosalpinx and systemic diseases; and women with other endocrine severe diseases, immune diseases, tumours, and abnormal chromosomes in either partner	TVUS	≥2: heterogeneous myometrial texture with the presence of a globular asymmetric uterus, thickening of the anterior and posterior myometrial wall, and irregular cystic areas within the myometrium	1155/1155 (100.0)	NR
Sharma et al. (2019)^20^	India	Single-centre retrospective cohort study	Infertile women <40 years old with endometriosis, adenomyosis, or both, and tubal factor infertility undergoing their first cycle of IVF–ICSI treatment.	Women with severe endometriosis who underwent excision or ablation, women with adenomyosis who underwent myolysis or wedge resection surgery, women with an endometrioma larger than 4 cm; women with fibroid, hydrosalpinx or uterine size greater than 12 weeks, FSH greater than 12 IU/mL.	2D-TVUS	≥3: globular uterus caused by overall increase in myometrial thickness; asymmetrically thickened anterior or posterior myometrial wall; poorly defined endo-myometrial interface; presence of heterogeneous myometrial area; and myometrial cysts	152/973 (15.6)	88 (57.9)
Benaglia et al. (2014)^21^	Italy	Single-centre prospective cohort study	Infertile women ≤42 years old undergoing IVF–ICSI with and without adenomyosis	Women whose uterine ultrasounds were doubtful, and those with fibroids or endometrial polyps. Women who did not achieve fresh embryo transfer because of cancelled cycle or unavailability of viable embryos were also excluded.	TVUS	≥1: asymmetrical thickening, irregular cystic areas, linear striations, irregular endometrial–myometrial junction	49/98 (50.0)	21 (42.9)
Park et al. (2016)^22^	South Korea	Single-centre retrospective cohort study	Infertile women included in this study have nonspecific infertile factors based on infertility work up except adenomyosis. They were divided into 3 groups according to which treatment they had: Group A: 116 pt with fresh ET without pretreatment. Group B: 87 patients with fresh with GNRH-a pretreatment and Group C: 38 patients with FET with GNRH pretreatment.	NR	TVUS	subjective enlargement of uterine corpus, asymmetrically thickened myometrium between anterior and posterior walls, heterogeneity of myometrium/hypoechoic striations, poor definition of endometrio-myometrial junction (minimum number not stated)	241/241 (100.0)	NR
Mavrelos et al. (2017)^23^	UK	Multi-centre prospective cohort study	Infertile women with or without adenomyosis who were planning to undergo their first IVF–ET cycle	Women who had received prolonged gonadotrophin-releasing hormone treatment at presentation, those with untreated submucous uterine fibroids or hydrosalpinges, those who did not undergo an ET due to poor embryonic development or endometrial polyp and those in whom the 3D volume dataset was missing or was not interpretable	3D-TVUS	≥1: asymmetrical myometrial thickening; parallel shadowing; linear striations; myometrial cysts; hyperechoic islands; adenomyomas; irregular endometrial–myometrial junction	72/375 (19.2)	NR
Higgins et al. (2021)^24^	Australia	Multi-centre prospective cohort study	Infertile women aged 18–45 undergoing fertility treatment	Women that had undergone vitrification oocyte thaw cycles or PGT	3D-TVUS	≥1 MUSA criteria	301/944 (31.9)	34 (11.3)
Neal et al. (2020)^25^	United States	Single-centre prospective cohort study	Infertile patients with or without adenomyosis planning to undergo endometrial preparation for transfer of a single euploid blastocyst	Women with BMI ≥40 kg/m^2^, congenital uterine anomaly, history of myomectomy, use of a gestational carrier (surrogat), and communicating hydrosalpinx	3D-TVUS	≥1 MUSA criteria	99/648 (15.3)	4 (4.0)
Trinchant et al. (2022)^26^	Spain	Multi-centre retrospective cohort study	Patients who underwent an ovum donation cycle due to advanced maternal age or low ovarian reserve. All received an elective single fresh embryo with and without adenomyosis	Women with multiple pregnancies, myomas, severe endometriosis (stages III–IV), uterine fibroids, uterine malformations, and/or those who underwent previous uterine surgeries.	TVUS	≥1: irregular endometrial–myometrial junction, ill-defined myometrial heterogeneity, presence of round anechoic myometrial cysts, asymmetrical thickening of anterior and posterior walls, adenomyoma	179/3307 (5.4)	Severe endometriosis (stages III–IV) exclude. Otherwise NR.
Han B et al. (2023)^27^	China	Single-centre retrospective cohort study	Infertile women ≤40 years old with adenomyosis or tubal infertility who underwent the first cycle of IVF-ET with ultra-long GnRH agonist protocol	Women with basal FSH level above 10 mIU/mL, previous surgery for adenomyosis, combined with uterine malformation, endometrial polyp, intrauterine adhesion, submucosal myoma, and combined with autoimmune or endocrine disorders. Also, for the adenomyosis groups, patients with uterine volume less than 56 cm^3^ were excluded, and for focal adenomyosis group (group B), patients with the mass diameter less than 3 cm were excluded.	TVUS	≥2: global uterine enlargement, asymmetrical myometrial thickening, linear striations, myometrial cysts, hyperechoic islands, and irregular or thickened junctional zone	1146/1665 (68.8)	NR
Cozzolino et al. (2024)^11^	Italy	Single-centre prospective cohort study	Infertile receivers of donor eggs with and without adenomyosis	Previous uterine surgery (myomectomy or adenomyomectomy), intramural (>3 cm) or submucosal uterine myoma. Mullerian abnormalities, endometrial abnormalities (e.g., polyps or hyperplasia), a thin endometrium (<6 mm), hydrosalpinx, vaginal or cervical abnormalities, thrombophilia or untreated thyroid dysfunction, or a sperm concentration of <5 million/mL.	2D/3D-TVUS	≥1 direct MUSA criteria: a round cystic area within the myometrium surrounded by a hyperechoic halo, hyperechogenic islands, and subendometrial hyperechogenic lines and buds.	114/228 (50.0)	20 (17.5)
Dason et al. (2023)^15^	Canada	Single-centre retrospective cohort study	Infertile receivers of donor eggs with and without adenomyosis	Women with abnormal uterine cavity (e.g., intracavitary fibroids and polyps) on sonohysterography or hysterosalpingography that was not corrected before embryo transfer	TVUS	≥1 MUSA criteria	76/100 (76.0)	10 (13.2)
Trinchant et al. (2025)^28^	Spain	Multi-centre retrospective cohort study	Patients that underwent a PGT-A program with and without adenomyosis (matched controls)	Women with multiple pregnancies, no clear diagnosis suggestive of adenomyosis.	2D/3D-TVUS	≥1 MUSA criteria (however, since the MUSA consensus was published midway through the study period, MUSA was not consistently applied).	228/456 (50.0)	31 (13.6)
Alson et al. (2024a)^10^	Sweden	Single-centre prospective cohort study	Women scheduled for their first ART with and without adenomyosis	Women with previous surgical destruction of superficial endometriotic lesions were excluded. Women with current hormonal treatment or previous uterine surgery	2D/3D-TVUS	≥1 MUSA criteria	290/1037 (27.9)	165 (56.9)
Arlıer et al. (2025)^29^	Turkey	Single-centre retrospective cross sectional study	Women with unexplained infertility who underwent IUI with and without adenomyosis	BMI ≥40 kg/m^2^, recent uterine surgery, resence of uterine and adnexal diseases, uterine myomas, breast disease incompatible with gonadotropin stimulation. Mild, incidental, or subclinical endometriosis identified retrospectively by ultrasound was not considered a primary exclusion from the unexplained infertility classification.	2D-TVUS and 3D-TAUS	MUSA criteria (minimum number not stated)	95/374 (25.4)	NR
Abu Hashim et al. (2020)^30^	Egypt	Single-centre cross sectional study	Infertile women ≤41 years the infertility clinic of a university teaching hospital with and without adenomyosis	Women with recurrent abortion and those with a previous diagnosis of adenomyosis.	2D-TVS	≥1 MUSA criteria	24/320 (7.5)	4 (19.0)^a^
Busnelli et al. (2024)^31^	Italy	Multi-centre retrospective cohort study	Women age between 25 and 42 affected by first-trimester RPL, defined as the previous spontaneous loss of two or more pregnancies	Women with infertiliy, relevant comorbidities (i.e. tumours, cardiovascular diseases, met-abolic diseases), women who had undergone metroplasty, myomectomy, minimally invasive treatments for uterine myomas (i.e. uterine artery embolisation, US-guided and magnetic resonance-guided high-intensity focused US, and transcervical radiofrequency ablation methods) or adenomyomectomy.	2D/3D-TVUS	≥1 MUSA criteria	100/442 (22.6)	NR
Alson et al. (2024b)^12^	Sweden	Single-centre prospective cross sectional study	Subfertile, non-smoking women between 25 and ≤39 years without common children with the present partner, with BMI ≤30 kg/m^2^ or a >10% weight loss for BMI <30–≤35 kg/m^2^, scheduled for their first ART.	Women with current hormonal treatment or previous uterine surgery, previous surgical destruction of superficial endometriotic lesions	2D/3D-TVUS	≥1 MUSA criteria	272/1160 (23.4)	22 (8.1)
Li X et al. (2021)^32^	China	Single-centre retrospective cohort study	Infertile women ≤40 years old with adenomyosis diagnosed on ultrasound who underwent IVF	Women wíth intrauterine adhesions, uterine malformations or leiomyomas that were submucosal or 2.0 cm or larger in diameter, patients with hydrosalpinx or systemic diseases; and those who had undergone preimplantation genetic test and egg donation cycles	TVUS	≥2: heterogeneous myometrial texture with the presence of a globular asymmetrical uterus, irregular cystic areas within the myometrium or thickening of the myometrial wall	158/158 (100.0)	39 (24.6)
Benlioglu et al. (2026)^14^	Turkey	Single-centre prospective cohort study	Infertile women 18–39 years old planned to start an IVF/ICSI cycle	Women with untreated hydrosalpinx or clinically significant intracavitary pathology, including FIGO type 0, 1, or 2 submucosal myomas, were not included in the study cohort unless these lesions had been surgically managed before ART. Couples with severe male factor infertility, such as azoospermia and patients with sonographically diagnosed with rASRM Grade 3–4 endometriosis were excluded.	2D-TVUS	In the presence of maximum junctional zone thickness >5 mm or maximum−minimum junctional zone difference >5 mm, plus other MUSA features (minimum number not stated)	76/314 (24.2)	Excluded women with severe endometriosis, otherwise NR.
Berbecaru et al. (2025)^33^	Roumania	Single-centre retrospective cohort study	Infertile women with adenomyosis compared to patients without infertility or gynaecological pathology	NR	2D/3D-TVUS	MUSA criteria (minimum number of criteria not stated)	100/140 (71.4)	NR
Etrusco et al. (2026)^34^	Spain	Single-centre retrospective cohort study	Infertile women <50 with a BMI ≤30 years old undergoing their first SET with donor oocytes with junctional zone adenomyosis and without adenomyosis	Age >50, BMI >30, women with concominant endometriosis, uterine fibroids of any size or location, müllerian anomalies, any endometrial pathology, thin endometrium (<6 mm), hydrosalpinx, previous uterine surgery, untreated endocrine disorders, severe dyspermia, double embryo transfer, or any SET other than the first, women with known thrombophilia or coagulation disorders	2D/3D TVUS	≥1 direct MUSA criteria in the inner myometrium (only junctional zone adenomyosis was included)	120/240 (50.0)	Excluded
Han SJ et al. (2026)^13^	South Korea	Single-centre retrospective cohort study	Infertile women <40 years women undergoing autologous embryo transfer with and without adenomyosis (matched-controls)	Women >40 years, women with endometrial thickness <7 mm att time of embryo transfer, women with uterine anomalies, adhesions, polyps, submycosal myomas or intramural myomas FIGO type 3, HIFU treatment, previous endometrial cancer or previous adenomyomectomy	2D/3D-TVUS	≥1 MUSA criteria or focal adenomyoma	300/600 (50.0)	NR
Li Z et al. (2025)^35^	China	Single-centre retrospective cohort study	Women <45 years diagnosed with adenomyosis who underwent their first IVF/ICSI cycle	Women with uterine malformations, intrauterine lesions or untreated hydrosalpinx	TVUS	MUSA criteria (minimum number of criteria not stated)	488/488 (100.0)	107 (21.9)
Li J et al. (2025)^36^	China	Single-centre retrospective cohort study	Infertile women diagnosed with adenomyosis with uterine diameters <70 mm	Malignant tumours, abnormal semen, chromosomal abnormality (either partner), AMH <0.5 ng/mL, age >45, oophorectomy/salpingo-oophorectomy, ectopic pregnancy history, hormone/GnRH therapy within 3 months, hydrosalpinx/tubal obstruction, cesarean scar diverticulum, intramural fibroids, intrauterine adhesions or uterine malformation, endometrial atypical hyperplasia, endocrine disorders (PCOS, thyroid), no intention of natural conception post-surgery.	TVUS	≥1 direct MUSA criteria	138/138 (100.0)	98 (71.0)
Madani et al. (2026)^37^	Iran	Single-centre prospective cross-sectional study	Infertile women seen in a national referral centre for infertility treatment	Women aged >45 years, women with multiple (more than two fibroids) and large fibroids (maximum diameter >3 cm), women who did not allow for vaginal examination (due to vaginis- mus), and those who did not feel inclined to participate in the study	2D-TVUS	Diffuse: ≥2 of the following–globular enlarged uterus, asymmetrical thickness of the myometrium, myometrial cysts and small hyperechoic myometrial islands, heterogeneous myometrial echotexture without considering fibroids, and hyperechoic sub-endometrial linear striations in the myometrium. Localised: heterogeneous mass without a defined margin	165/963 (17.1)	NR
Surcel et al. (2026)^38^	Roumania	Single-centre prospective cohort study	Infertile women with adenomyosis undergoing IVF treatment	Age >40 years; untreated hydrosalpinx confirmed by imaging; moderate or severe endometriosis; known inherited thrombophilia or antiphospholipid syndrome; chromosomal abnormalities in either partner; a body mass index greater than 35 kg/m^2^; or major uterine abnormalities, including congenital anomalies, submucosal fibroids, fibroids larger than 5 cm, large endometrial polyps, or severe intrauterine adhesions (synechiae)	TVUS	≥1 direct MUSA criteria	35/85 (41.2)	Excluded women with moderate or severe endometriosis, otherwise NR.
Tokhunts et al. (2026)^39^	Armenia	Multicenter prospective cohort study	Women with primary or secondary infertility, a history of two or more implantation failures following in-vitro fertilization (IVF), or two or more consecutive miscarriages.	Factors that impeded adequate ultrasound visualisation of the uterus, such as abdominal obesity, large or multiple fibroids, intracavitary pathology (e.g., submucosal fibroids, uterine synechiae), or significant pelvic adhesions.	2D/3D-TVUS	MUSA criteria (minimum number of criteria not stated)	149/518 (28.8)	44 (29.5) with DIE
Wang et al. (2025)^40^	China	Single-centre retrospective cohort study	Infertile women aged 20–42 undergoing their first FET cycle with high-quality embryos, with and without adenomyosis (matched controls)	Uterine anomaly, intrauterine adhesions, current pregnancy, presence of malignancy, PGT cycle poor-quality embryo, or not first FET cycle	2D/3D-TVUS	≥1 direct and ≥1 indirect MUSA criteria	879/1758 (50.0)	208 (23.7)

NR, not reported; IVF, in-vitro fertilization; FET, frozen embryo transfer; SET, single embryo transfer; US, ultrasound; BMI, Body Mass Index; AMH, anti-Müllerian hormone; MUSA, Morphological Uterus Sonographic Assessment; TVUS, trans-vaginal ultrasound; TAUS, trans-abdominal ultrasound; ICSI, intracytoplasmic sperm injection; FSH, follicle stimulating hormone; PGT-A, Pre-implantation Genetic Testing for Aneuploidies; RPL, recurrent pregnancy loss; ART, assisted reproductive technology; IUI, intrauterine insemination; DIE, deep infiltrating endometriosis.

a Diagnosis of endometriosis was based on a previous laparoscopic report or if an ovarian endometrioma was found during the 2D-TVS.

In total, the studies included 19,510 participants, of whom 7569 were diagnosed with adenomyosis. Twenty-seven studies investigated women with infertility, one included only women with recurrent pregnancy loss (RPL; ≥2 previous pregnancy losses), and one study included both women with infertility and RPL^39^ (Table 1).

We also identified 10 registered clinical trial protocols: five studies were still recruiting, four had been completed but remain unpublished, and one had been withdrawn. Additionally, our search retrieved 30 records available only as abstracts. Of these, 16 were never subsequently published in peer-reviewed journals; nine of these did not report the outcomes of interest. Among the remaining seven abstracts, two were of recent date (2024 and 2025) and therefore might not yet be published. Four of the unpublished abstracts reported on potentially relevant variables. A complete list of studies excluded at full-text screening with reasons for exclusion is provided in Supplementary Table C.

Studies were conducted across a wide range of countries, with European countries most represented (Table 1). Assessment of ultrasonographic characteristics in relation to the primary outcome varied across studies; seven studies assessed focal and diffuse adenomyosis,^11^, ^15^, ^21^, ^22^, ^26^, ^27^, ^28^ six studies evaluated inner and outer myometrial adenomyosis involvement,^10^, ^11^, ^15^, ^19^, ^34^, ^40^ seven evaluated uterine volume,^9^, ^13^, ^15^, ^21^, ^32^, ^35^, ^36^ four classified direct and indirect features,^10^, ^13^, ^14^, ^29^ nine assessed disease severity^10^, ^11^, ^13^, ^15^, ^20^, ^23^, ^24^, ^25^, ^34^ and four examined individual sonographic signs.^10^, ^13^, ^23^, ^25^ Fifteen studies reported a concomitant diagnosis of endometriosis in the adenomyosis population (ranging from 4% to 71%) while fourteen studies did not report on endometriosis status (Table 1).

Twenty studies consistently applied the MUSA criteria for defining adenomyosis, although the minimum number of features required for diagnosis varied; nine studies accepted only one feature, one required at least two features, four required at least one direct MUSA feature, one required at least one direct and one indirect feature, and five did not specify the minimum number (Table 1).

Three author groups each had two studies included in our data extraction and were assessed for cohort overlap. The studies by Sharma et al.^19^, ^20^ did not have overlapping recruitment periods, and the studies by Trinchant et al.^26^, ^28^ had different populations (donor oocyte receivers vs euploid PGTA transfers). The studies by Alson et al.^10^, ^12^ used overlapping cohorts; however one reported prevalence of adenomyosis features while the other reported on the number of features and pregnancy outcomes.

### Quality assessment and certainty of evidence

The 22 studies that reported on pregnancy outcomes were assessed via the NOS; four studies had moderate RoB and 18 had low RoB. Of the 14 studies that reported on prevalence, five had high RoB, five had moderate risk, and four had low RoB according to JBI (Supplementary Tables D and E). No studies were excluded from analyses based on RoB score. No formal assessment of reporting bias was conducted.

Certainty of evidence for reproductive outcomes, assessed using GRADE, is summarised in Table 2, with the categorial ratings and reasons for downgrading presented in Supplementary Table F. Certainty was very low for 18 out of 21 outcomes, primarily driven by indirectness related to variation in diagnostic criteria across studies and imprecision from confidence intervals crossing prespecified clinical thresholds (OR <0.75 or >1.25). Certainty was moderate for clinical pregnancy in the high-vs-low uterine volume comparison, and low for live birth in both the uterine volume and internal-vs-no-adenomyosis comparison, reflecting narrower confidence intervals and more consistent diagnostic definitions across the included studies.

**Table 2:** Summary of findings–GRADE.

Ultrasound feature	Clinical pregnancy		Live birth		Pregnancy loss	
	Effect estimate	Certainty	Effect estimate	Certainty	Effect estimate	Certainty
Diffuse vs focal	OR 0.92 (95% CI 0.56–1.50)	Very low	OR 0.95 (95% CI 0.40–2.29)	Very low	OR 1.15 (95% CI 0.51–2.63)	Very low
Focal vs no AM	OR 0.70 (95% CI 0.25–1.96)	Very low	OR 0.46 (95% CI 0.17–1.27)	Very low	OR 2.40 (95% CI 0.75–7.70)	Very low
Diffuse vs no AM	OR 0.67 (95% CI 0.45–1.00)	Very low	OR 0.52 (95% CI 0.34–0.79)	Very low	OR 2.25 (95% CI 0.93–5.43)	Very low
Large vs small uterine volume	OR 0.93 (95% CI 0.86–1.01)	Moderate	OR 0.67 (95% CI 0.42–1.07)	Low	OR 2.54 (95% CI 0.51–12.51)	Very low
Internal AM vs no AM	OR 0.62 (95% CI 0.48–0.81)	Very low	OR 0.49 (95% CI 0.42–0.57)	Low	OR 2.67 (95% CI 1.07–6.70)	Very low
Internal AM vs external AM	OR 0.50 (95% CI 0.08–3.25)	Very low	OR 0.48 (95% CI 0.12–1.95)	Very low	OR 1.75 (95% CI 0.03–100.59)	Very low
External AM vs no AM	OR 1.21 (95% CI 0.34–4.26)	Very low	OR 1.00 (95% CI 0.11–8.85)	Very low	OR 1.43 (95% CI 0.00–1245.26)	Very low

AM, adenomyosis; OR, odds ratio.

### Publication bias

Funnel plots were generated for all meta-analysed comparisons and prevalence outcomes (Supplementary Figures A–T). Visual inspection did not reveal consistent evidence of asymmetry.

### Qualitative analysis on features of adenomyosis and pregnancy outcome

The following section includes results regarding the primary outcome related to separate ultrasound features of adenomyosis.

#### Direct and indirect adenomyosis features

Four studies reported on pregnancy outcomes according to direct and indirect adenomyosis features.^10^, ^13^, ^14^, ^29^

Alson et al. (2024a)^10^ (n = 1037) found that women with at least one direct adenomyosis feature had a lower cumulative live birth rate than women without (24.5% vs 42.7%; p < 0.001), with an age-adjusted RR of 0.62 (95% CI 0.43–0.88; p = 0.007) and a higher risk of pregnancy loss after frozen embryo transfer (adjusted RR 2.88, 95% CI 1.49–5.57; p = 0.002).

Similarly, Benlioglu et al.^14^ (n = 314) showed that women with direct markers of adenomyosis had a significantly lower adjusted OR of live birth (aOR: 0.42, 95% CI 0.20–0.84) compared to women without adenomyosis, whereas women with any sign of adenomyosis did not reach significance (aOR 0.61, 95% CI 0.34–1.08).

In contrast, Han SJ et al. (2026)^13^ (n = 600) found a significantly higher biochemical pregnancy loss rate in women with adenomyosis (any feature) compared to controls (11.3% vs 6.0%; p = 0.020), but found no significant differences in clinical pregnancy, ongoing pregnancy, biochemical pregnancy loss, or pregnancy loss rate when comparing women with only direct features, only indirect features, or both.

Arlıer et al.^29^ (n = 374) compared the number of direct and indirect adenomyosis features between women who did and did not achieve pregnancy after IUI. Feature counts were slightly higher in women who did not achieve pregnancy, but the difference was not statistically significant for either direct (1.94 vs 2.17; p = 0.984) or indirect features (1.38 vs 1.82; p = 0.401).

#### Adenomyosis in outer and inner myometrium

Six studies reported on pregnancy outcomes in relation to inner and outer myometrial adenomyosis involvement.^10^, ^11^, ^15^, ^19^, ^34^, ^40^

Sharma et al. (2025)^19^ (n = 585) found that in women with diffuse adenomyosis, junctional zone involvement was associated with lower clinical pregnancy rate (23.4% vs 42.5%; crude OR 0.41, 95% CI 0.26–0.65) and live birth rate (16.4% vs 25.8%; crude OR 0.57, 95% CI 0.34–0.94) compared to outer myometrium lesions.

Similarly, Cozzolino et al.^11^ (n = 228) reported in a prospective donor oocyte transfer cohort that, among women with adenomyosis, junctional zone involvement was associated with a higher risk of pregnancy loss compared with adenomyosis not involving the junctional zone (crude RR 3.28, 95% CI 1.38–7.78), as was combined junctional zone and outer myometrium involvement (RR 2.17, 95% CI 1.18–4.99). In contrast, involvement of the outer myometrium alone was associated with a lower risk of pregnancy loss (RR 0.30, 95% CI 0.13–0.72).

Studies comparing inner and outer myometrial involvement to women without adenomyosis show similar results. Etrusco et al.^34^ (n = 240) showed that adenomyosis located in the junctional zone had a decreased OR of live birth (aOR = 0.43, 95% CI 0.24–0.76, p = 0.004) and an increased odds of pregnancy loss (aOR = 3.32, 95% CI 1.42–7.82, p = 0.005), compared to women without adenomyosis. Likewise, Wang et al.^40^ (n = 1758) reported that women with adenomyosis in the junctional zone had significantly lower live birth rates (crude OR 0.52, 95% CI 0.34–0.77) and clinical pregnancy rates (crude OR 0.63, 95% CI 0.43–0.92) than matched controls, along with a higher rate of early pregnancy loss (crude OR 2.21, 95% CI 1.11–4.40). In contrast, women with adenomyosis in the outer myometrium showed no significant differences in live birth (crude OR 0.96, 95% CI 0.74–1.24), clinical pregnancy (crude OR 0.99, 95% CI 0.77–1.28), or early pregnancy loss (crude OR 1.09, 95% CI 0.69–1.75) compared with matched controls.

Alson et al. (2024a)^10^ found that, compared to women without adenomyosis, features located in the inner myometrium markedly reduced live birth rate (adjusted RR 0.29, 95% CI 0.11–0.74), whereas features confined to the outer myometrium increased live birth rate (adjusted RR 2.61, 95% CI 1.42–4.80).

By contrast, Dason et al.^15^ (n = 100) observed that all women diagnosed with adenomyosis had an interrupted junctional zone as their defining feature. Overall, there were no significant differences in live birth, clinical pregnancy, biochemical pregnancy, or pregnancy loss rates between women with and without adenomyosis. However, when restricted to a focal interrupted junctional zone located in the fundal (crude OR 0.42, 95% CI 0.18–0.95) or posterior (crude OR 0.40, 95% CI 0.18–0.88) uterus, clinical pregnancy rate was significantly reduced compared with women without adenomyosis, though live birth rate did not differ significantly.

Three studies were included in the meta-analysis of internal vs external adenomyosis and pregnancy outcomes, across three comparisons: internal vs external, internal vs no adenomyosis, and external vs no adenomyosis (Supplementary Figures A–C). Internal adenomyosis was associated with a significant decrease in clinical pregnancy (OR 0.62, CI: 0.48–0.81) and live birth (OR 0.49, CI: 0.42–0.57), and a significant increase in pregnancy loss (OR 2.67, CI: 1.07–6.70), compared with no adenomyosis in the random effects model; none of these associations were robust to exclusion of individual studies. External adenomyosis vs no adenomyosis and internal vs external adenomyosis showed no significant associations with any pregnancy outcome.

#### Diffuse and focal adenomyosis

Seven studies compared focal and diffuse adenomyosis.^11^, ^15^, ^21^, ^22^, ^26^, ^27^, ^28^

Han B et al. (2023)^27^ (n = 1665) found that compared to focal adenomyosis, diffuse adenomyosis was associated with lower clinical pregnancy rate (32.5% vs 41.5%) and live birth rate (18.7% vs 30.2%), and higher PL risk (41.7% vs 27.2%); adjusted analyses (age, BMI, and antral follicle count) confirmed reduced odds of clinical pregnancy (adjusted OR 0.71, 95% CI 0.54–0.93) and live birth (adjusted OR 0.53, 95% CI 0.39–0.73), and increased odds of pregnancy loss (adjusted OR 1.88, 95% CI 1.18–2.99). Cozzolino et al.^11^ reported no differences in implantation, clinical pregnancy rate, or live birth rate between focal and diffuse adenomyosis, but diffuse adenomyosis lesions, particularly severe or junctional zone-involving, doubled the pregnancy loss risk (crude RR 2.29, 95% CI 1.22–4.30).

Conversely, Trinchant et al. (2025)^28^ (n = 456) found that compared to diffuse adenomyosis, focal adenomyosis was linked to lower live birth rate (15% vs 28%; adjusted OR 0.30, 95% CI 0.10–0.87) and implantation rate (adjusted OR 0.41, 95% CI 0.17–0.94), without differences in pregnancy loss rate. Dason et al.^15^ showed that larger focal lesions reduced live birth rate (adjusted OR 0.09 per 1-cm increase, 95% CI 0.01–0.63). Park et al.^22^ (n = 241) observed nonsignificant trends towards higher clinical pregnancy rate in focal vs diffuse adenomyosis.

Two studies by Trinchant et al. (2022)^26^ (n = 3307) and Benaglia et al.^21^ (n = 98) reported no differences in clinical pregnancy rate and live birth rate between focal and diffuse disease.

Seven studies were included in a meta-analysis of diffuse and focal adenomyosis and pregnancy outcomes, across three comparisons: diffuse vs no adenomyosis, focal vs no adenomyosis, and focal vs diffuse adenomyosis (Fig. 2). Diffuse adenomyosis was associated with a significant decrease in live birth (OR 0.52, CI: 0.34–0.79, I^2^: 63.3%) and a non-significant decrease in clinical pregnancy (OR 0.67, CI: 0.45–1.00, I^2^: 53.1%) compared to no adenomyosis in the random effects model; both associations were driven by retrospective studies (test for subgroup differences, p < 0.05 for both), and neither was fully robust on leave-one-out sensitivity analysis. Focal adenomyosis showed non-significant trends towards lower clinical pregnancy and live birth and higher pregnancy loss compared with no adenomyosis, with a significant subgroup difference by design for clinical pregnancy (p = 0.0204; Supplementary Figure D). Pregnancy outcomes for focal vs diffuse adenomyosis were comparable in the random effects model (Supplementary Figure F).

**Fig. 2:**
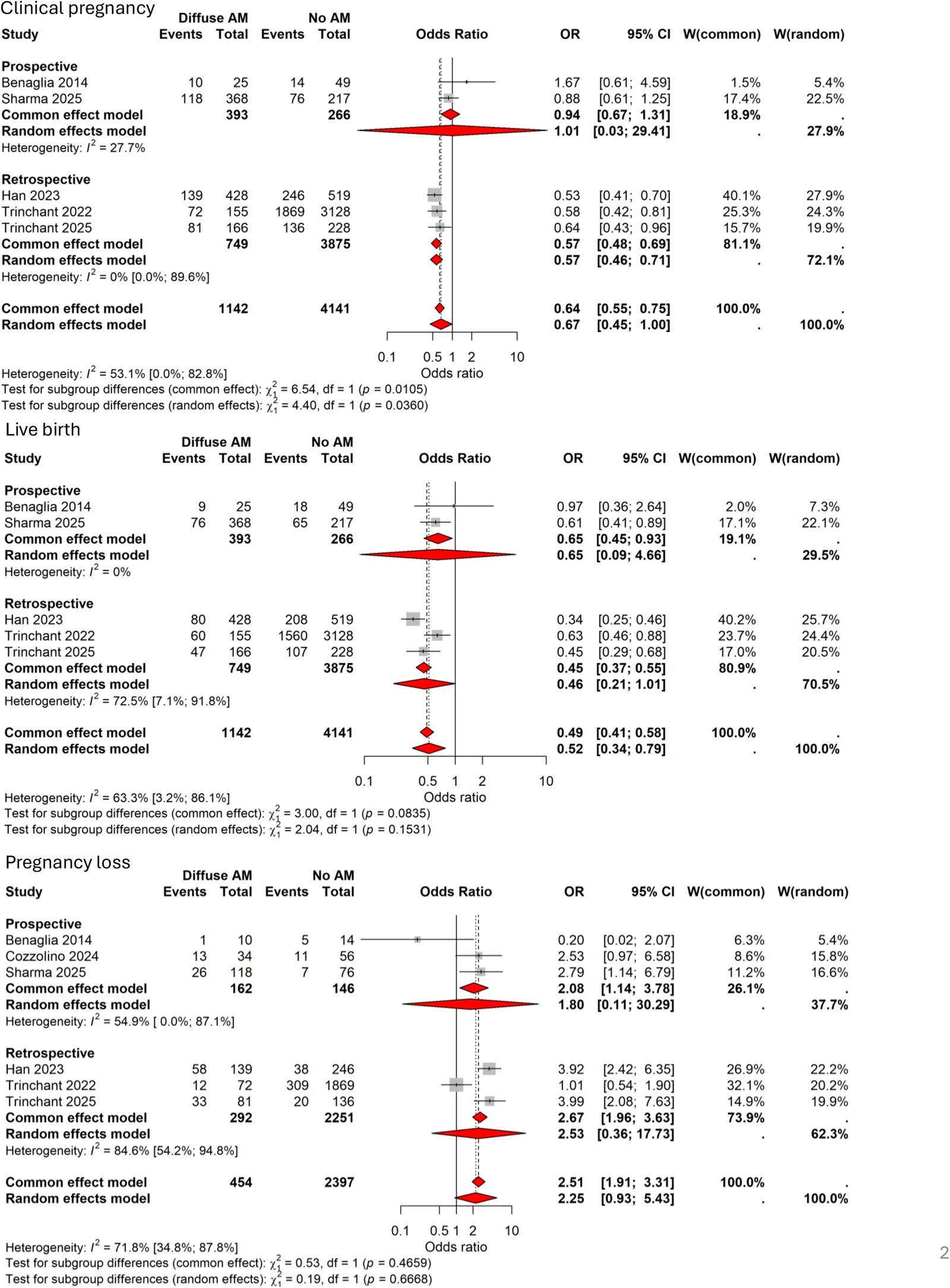
Meta-analysis by design of diffuse adenomyosis vs no adenomyosis. N adenomyosis/controls: Clinical pregnancy (n = 1142/4141), live birth (n = 1142/4141) and pregnancy loss (n = 454/2397). AM, Adenomyosis; OR, Odds ratio.

#### Severity of adenomyosis

Nine studies examined the severity of adenomyosis and pregnancy outcomes.^10^, ^11^, ^13^, ^15^, ^20^, ^23^, ^24^, ^25^, ^34^

The severity of adenomyosis was expressed by the number of MUSA features on ultrasound in all but one case.^34^ Mavrelos et al.^23^ (n = 375) reported progressively lower clinical pregnancy rate with increasing number of adenomyosis features, with ≥4 features significantly reducing success (adjusted OR 0.35, 95% CI 0.15–0.82), independent of age and ovarian reserve. Alson et al. (2024a)^10^ similarly found that cumulative live birth declined with more adenomyosis features, from an adjusted RR of 0.45 (95% CI 0.21–0.95) with one feature to 0.12 (95% CI 0.03–0.55) with three features. Cozzolino et al.^11^ reported no effect of adenomyosis severity on implantation rate, clinical pregnancy rate, or live birth rate, but severe adenomyosis (defined as a summation score of >7 based on the size and location of adenomyosis features) increased the pregnancy loss risk (adjusted OR 2.83, 95% CI 1.10–7.26). In contrast, Higgins et al.^24^ (n = 944), Neal et al.^25^ (n = 648), Dason et al.^15^ and Han SJ et al. (2026).^13^ found no significant differences in in-vitro fertilization (IVF) outcomes by adenomyosis severity, while Sharma et al. (2019)^20^ (n = 973) observed nonsignificant trends towards higher pregnancy loss and lower live birth with ≥5 features.

In a subgroup analysis restricted to women with junctional zone-only adenomyosis who achieved pregnancy, Etrusco et al.^34^ found that the relative risk of maintaining an ongoing pregnancy (vs pregnancy loss) declined with increasing disease extent: RR 0.85 (95% CI 0.40–1.78, p = 0.62) for mild involvement (<25% of junctional zone surface), RR 0.65 (95% CI 0.33–1.27, p = 0.21) for moderate involvement (25–50%), and RR 0.17 (95% CI 0.04–0.67, p = 0.007) for severe involvement (>50%), with only the severe category reaching statistical significance.

#### Uterine size

Seven studies assessed uterine size and pregnancy outcomes in women with adenomyosis.^9^, ^13^, ^15^, ^21^, ^32^, ^35^, ^36^

Li X et al. (2021)^32^ (n = 158) reported higher PL rate (51.3% vs 16.3%, p = 0.001) and lower live birth rate (25.3% vs 43.4%, p = 0.020) with higher uterine volume (>98.8 cm^3^), which remained significant after adjustment for age and BMI (adjusted OR for PL 8.51, 95% CI 2.29–25.75; adjusted OR for live birth rate 0.45, 95% CI 0.21–0.97). Zhang et al.^9^ (n = 1155) found similar clinical pregnancy rate by uterine size but higher PL rate (40.2% vs 25.3%, p < 0.001) and lower live birth rate (22.1% vs 28.9%, p = 0.004) when uterine volume exceeded 8-week gestational size. Li J. et al. (2025)^36^ (n = 138) reported that in women with adenomyosis, those who achieved a live birth had a smaller mean initial uterine diameter than those who did not (5.71 ± 1.27 cm vs 6.17 ± 1.46 cm, p = 0.004), and Li Z et al. (2025)^35^ (n = 488) similarly found a higher mean uterine volume among women with adenomyosis who did not achieve a live birth compared with those who did (60.4 cm^3^ vs 48.7 cm^3^, p = 0.003).

In contrast, Benaglia et al.^21^ and Dason et al.^15^ found no association between uterine volume and pregnancy outcomes. Similarly, Han SJ et al. (2026),^13^ analysed uterine volume as a continuous covariate and found no association with clinical pregnancy rate (crude OR 1.00, 95% CI 0.99–1.01), ongoing pregnancy rate (crude OR 1.00, 95% CI 0.99–1.00), pregnancy loss rate (crude OR 1.00, 95% CI 0.99–1.01), or biochemical pregnancy loss rate (crude OR 0.99, 95% CI 0.99–1.01) per mL increment, despite uterine volume being significantly larger in their adenomyosis group than in matched controls (85.3 ± 48.7 cm^3^ vs 58.9 ± 21.2 cm^3^, p < 0.001).

Three studies were included in the meta-analysis of high vs low uterine volume and pregnancy outcomes (Fig. 3). High uterine volume showed a non-significant trend towards reduced live birth (OR 0.67, CI: 0.42–1.07, I^2^: 0.0%) and clinical pregnancy (OR 0.93, CI: 0.86–1.01, I^2^: 0.0%) compared to low uterine volume, with no significant difference in pregnancy loss rate. Leave-one-out sensitivity analysis showed that the clinical pregnancy estimate reached significance upon exclusion of Li X et al. (2021),^32^ while other outcomes were robust (Supplementary Figure I).

**Fig. 3:**
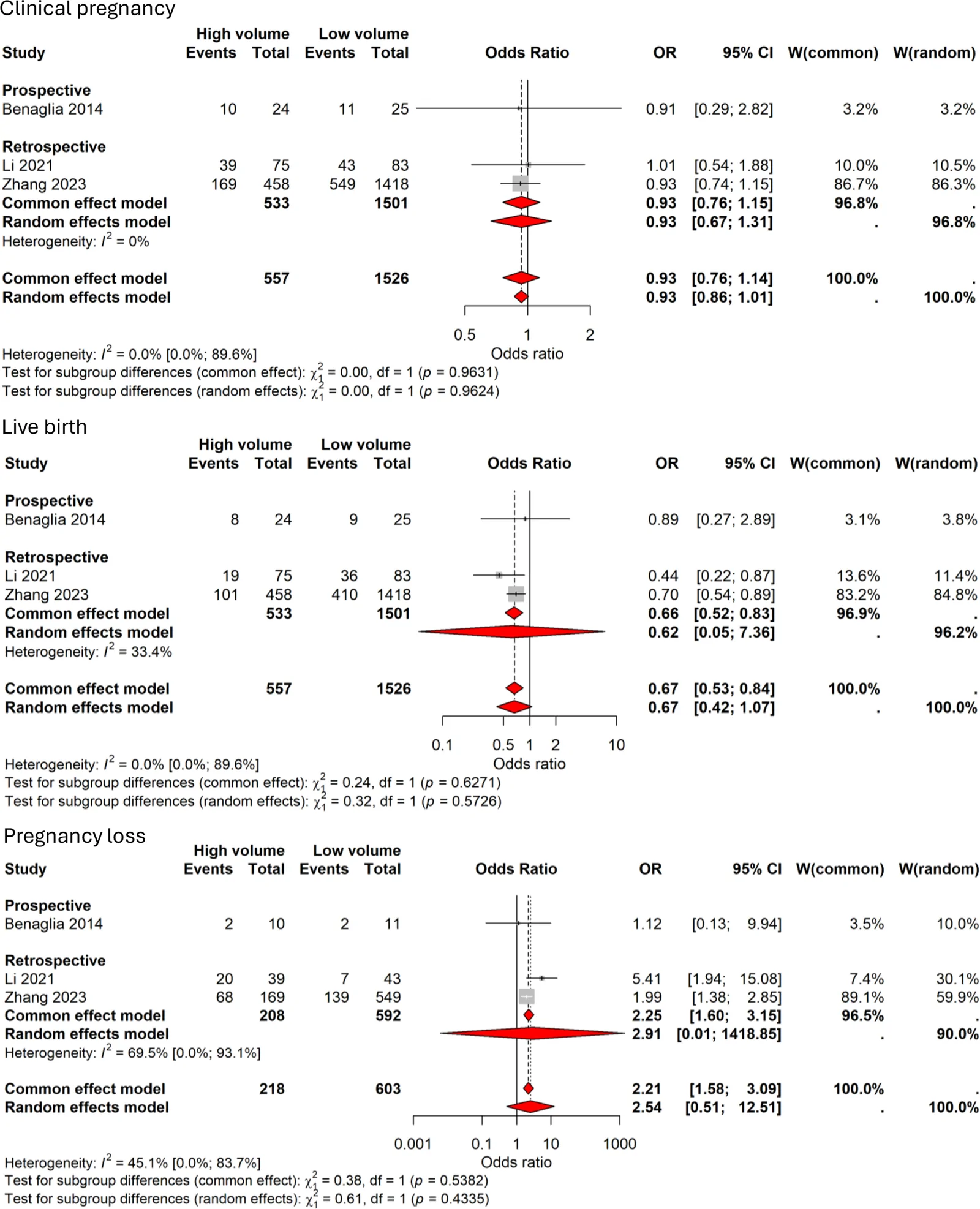
Meta-analysis, by design of high vs low uterine volume. N high volume/low volume: Clinical pregnancy (n = 557/1526), live birth (n = 557/1526) and, pregnancy loss (n = 218/603). OR, Odds ratio.

#### Individual adenomyosis features

Four studies reported outcomes based on individual adenomyosis features.^10^, ^13^, ^23^, ^25^

Alson et al. (2024a)^10^ found that among individual MUSA features, only an interrupted junctional zone was significantly associated with poorer IVF outcomes, with a markedly reduced live birth rate (adjusted RR 0.34, 95% CI 0.28–0.51, p < 0.001), compared to women without junctional zone involvement (including those with and without adenomyosis). This finding was confirmed by Han SJ et al. (2026),^13^ who, after adjusting for age, AMH, number of embryos transferred, and PGT-A utilisation, found that an interrupted junctional zone was associated with a significantly lower clinical pregnancy rate (aRR 0.75, 95% CI 0.58–0.95, p = 0.020) and ongoing pregnancy rate (aRR 0.72, 95% CI 0.52–0.99, p = 0.049), along with a significantly increased risk of biochemical pregnancy loss (aRR 1.98, 95% CI 1.02–3.84, p = 0.044) compared with women without junctional zone involvement. Pregnancy loss risk in this study was not significantly increased (aRR 1.21, 95% CI 0.64–2.32, p = 0.557).

Mavrelos et al.^23^ reported that no single feature consistently predicted clinical pregnancy rate, though an irregular junctional zone showed a strong negative trend (adjusted OR 0.13, 95% CI 0.02–1.00). In contrast, Neal et al.^25^ observed no independent association between any MUSA feature and pregnancy outcomes after single euploid blastocyst transfer.

#### Prevalence of adenomyosis features

Of the 14 studies reporting the prevalence of adenomyosis features,^12^, ^13^, ^15^, ^23^, ^24^, ^25^, ^27^, ^29^, ^30^, ^31^, ^33^, ^37^, ^38^, ^39^ ten assessed prevalence in infertile women using the MUSA criteria (Table 1). Ten studies reported on hyperechogenic islands with a mean prevalence of 31% (CI: 13–58%, I^2^: 97.6%), thirteen on myometrial cysts with a mean prevalence of 36% (CI: 15–64%, I^2^: 98.3%), eight on lines and buds with a mean prevalence of 27% (CI: 11–52%, I^2^: 97.8%), eleven on asymmetrical wall thickening with a mean prevalence of 36% (CI: 17–60%, I^2^: 98.7%), nine on fan-shaped shadowing with a mean prevalence of 20% (CI: 7–46%, I^2^: 96.3%), seven on globular uterus with a mean prevalence of 22% (CI: 6–54%, I^2^: 98.6%), four on interrupted junctional zone with a mean prevalence of 57% (CI: 4–98%, I^2^: 46.4%), eight on irregular junctional zone with a mean prevalence of 26% (CI: 5–71%, I^2^: 97.5%), and five on translesional vascularity with a mean prevalence of 13% (CI: 2–49%, I^2^: 96.0%).

Sensitivity analyses excluding studies at high RoB yielded comparable pooled prevalence estimates for most MUSA features, with only minor differences (Supplementary Figures L–T). The pooled prevalence of interrupted junctional zone decreased after exclusion of high-risk studies; this reflected the exclusion of Dason et al.^15^ which reported a prevalence of 100% while the remaining three studies reported consistent results.

In the single study reporting on 100 women with recurrent pregnancy loss and adenomyosis, adenomyosis was identified in 23% (95% CI: 19–27%).^31^ Among the direct features, myometrial cysts were most common (66%, 95% CI: 56–75%), followed by hyperechogenic islands (53%, 95% CI: 43–63%) and echogenic subendometrial lines and buds (27%). Among the indirect features, asymmetrical myometrial thickening (50%, 95% CI: 40–60%) and irregular junctional zone (53%) were most frequent, followed by interrupted junctional zone (50%) and globular uterus (44%), with fan-shaped shadowing (34%) and translesional vascularity (17%) least common.

## Discussion

In this systematic review and meta-analysis we studied the impact of different subtypes of ultrasound-diagnosed adenomyosis on IVF outcomes and the prevalence of adenomyosis features in women with infertility or pregnancy loss.

Our first main finding is that internal (junctional zone) adenomyosis was more strongly associated with adverse pregnancy outcomes. In our meta-analysis, internal adenomyosis showed significantly poorer clinical pregnancy and live birth outcomes compared with no adenomyosis, and two recent large studies^10^, ^13^ independently identified an interrupted junctional zone as the single feature most strongly associated with adverse IVF outcomes. The junctional zone plays a central role in reproduction by coordinating uterine peristalsis required for sperm transport and implantation, and junctional zone thickening has been identified as an independent risk factor for implantation failure in IVF.^7^ Indeed, adenomyosis-related junctional zone dysfunction has been linked to impaired endometrial receptivity, including dysregulated expression of implantation-related genes (namely the *HOXA* genes), progesterone resistance, and a non-receptive endometrium during the window of implantation.^41^, ^42^ Structural junctional zone abnormalities may also contribute to defective placentation and impaired trophoblast invasion,^43^ potentially driven by local hyperestrogenism and chronic inflammation.^44^ Such alterations may impair spiral artery remodelling and uteroplacental perfusion, thereby increasing the risk of pregnancy loss.^31^, ^45^ However, the certainty of evidence for the association between internal adenomyosis and pregnancy outcomes was rated low to very low using GRADE, and the pooled effect was not robust to exclusion of individual studies in sensitivity analysis.

Our second main finding is that diffuse adenomyosis was associated with a significantly reduced live birth rate compared with women without adenomyosis, alongside non-significant trends towards a lower clinical pregnancy rate and a higher pregnancy loss rate. These findings were largely driven by the large, low-risk-of-bias study by Han B et al.^27^ (2023) (n = 1665), and align with those of previous reports.^8^, ^46^ Biologically, diffuse adenomyosis may confer poorer reproductive outcomes due to global disruption of the junctional zone and myometrium, leaving limited healthy tissue for functional compensation. In contrast, our meta-analysis revealed no statistically significant differences when comparing diffuse and focal adenomyosis directly, perhaps due to the inconsistency in ultrasonographic definitions of focal and diffuse adenomyosis and the lack of a mixed-type classification. Unlike diffuse disease, focal adenomyosis is difficult to characterise as a single entity, as its reproductive impact appears to depend heavily on lesion size and location,^15^ with endometrium-near lesions being more detrimental than those located in the outer myometrium.^10^, ^11^ Furthermore, focal adenomyosis located in the outer myometrium has been linked to increased presence of concomitant endometriosis, presenting additional confounding.^47^

Our third main finding is that higher uterine volume showed a non-significant trend towards lower clinical pregnancy and live birth rates, with no significant difference in pregnancy loss rate. Although not a MUSA criterion, uterine enlargement commonly accompanies adenomyosis due to muscular hypertrophy, producing the globular uterine shape considered an indirect feature of the disease, and may reflect disease progression and lesion accumulation.^4^ Even in women without adenomyosis, higher uterine volume has been linked to reduced clinical pregnancy and live birth rates^48^, ^49^ and increased risk of pregnancy loss,^50^ suggesting uterine size itself may independently affect reproductive outcomes. However, not all studies support this association: Han SJ et al. (2026) (N = 600) found no significant relationship between uterine volume and any reproductive outcome when analysed continuously, despite significantly larger volumes in their adenomyosis group than in matched controls.^13^ This discrepancy may partly reflect the absence of standardised volume cut-offs across studies and it remains uncertain whether the association between uterine volume and adverse reproductive outcomes is causal or simply reflects greater disease severity.

Our qualitative analysis found that evidence on the prognostic relevance of direct vs indirect adenomyosis features is limited and inconsistent. Two studies found that direct features were associated with reduced live birth rates.^10^, ^14^ In contrast, two other studies found no significant association between feature type and pregnancy outcomes.^13^, ^29^ Direct features indicate the presence of ectopic endometrial tissue in the myometrium and are pathognomonic, while indirect features are those that are secondary to the presence of endometrial tissue in the myometrium, merely suggestive of the disease.^4^ However, there is considerable variation in diagnostic criteria across studies, with some studies requiring a direct feature for diagnosis of adenomyosis,^11^ excluding indirect-only cases, while others accept any MUSA feature. This heterogeneity contributes to inconsistent results and limits cross-study comparability. Whether the distinction between direct and indirect features has independent prognostic relevance for reproductive outcomes or primarily serves as a diagnostic construct warrants further investigation.

Qualitative analysis further revealed that the effect of increasing adenomyosis severity on reproductive outcomes, quantified by the number of MUSA features, was inconsistent across studies, with roughly half reporting a significant association with at least one outcome and the remainder finding no effect. Of the nine studies that assessed disease severity, four found at least a partially significant association with pregnancy outcome,^10^, ^11^, ^23^, ^34^ four did not,^13^, ^15^, ^24^, ^25^ and one observed a non-significant trend towards reduced live birth rate among women with five or more MUSA features.^20^ Although counting MUSA features offers a pragmatic means of estimating severity, it remains a subjective approach without standardised thresholds for mild, moderate, or severe adenomyosis. Moreover, it does not take into account the specific features and their location within the uterus. This lack of consensus likely contributes to variability in reported reproductive outcomes and highlights the need for uniform severity criteria.

Our secondary objective was to systematically summarise the prevalence of adenomyosis features. We found substantial heterogeneity in the reported prevalence of individual features among infertile women, exceeding 90% for most studies. Three of the 14 studies did not consistently apply MUSA criteria, representing a possible limitation. Even among studies that used MUSA and included similar patient populations, there were large discrepancies in prevalence estimates, and sensitivity analyses excluding studies at high RoB resulted in only marginal changes for most MUSA features, indicating this was not the primary driver of heterogeneity. Our findings underscore that interobserver variability remains a major challenge in the sonographic assessment of adenomyosis. Indeed, a 2025 study showed that inter- and intraobserver agreement of the identification of ultrasonographic features of adenomyosis as defined by the revised MUSA was moderate, with indirect features showing poor interobserver reproducibility.^51^ Analyses were further limited by the low prevalence of individual features and the small sample sizes of most studies. The use of 3D-US allows better visualisation of certain adenomyosis lesions, such as the junctional zone, yet only 9 out of 14 studies reported its use, suggesting some lesions may be underreported. To improve diagnostic consistency and comparability across studies, further refinement of the MUSA definitions, with clearer criteria and size thresholds for features such as cysts and islands, is needed. Greater emphasis on operator training, standardised imaging protocols, and global alignment in the interpretation of ultrasonographic features will be essential to enhance reliability and reduce variability in adenomyosis diagnosis.

Prevalence data for the RPL population were derived from a single study reporting on 100 women with adenomyosis,^31^ precluding meaningful comparison with the pooled infertility estimates. Notably, however, this study specifically excluded women with infertility, suggesting that the features of adenomyosis observed in this RPL population are unlikely to simply reflect an underlying subfertility phenotype. Although outcomes were not reported by individual subtype, adenomyosis was associated with more severe forms of RPL, suggesting a link between disease severity and reproductive prognosis that merits further study.

Key strengths of this review include the explorative approach on adenomyosis features which ensured that all studies reporting on subtypes of adenomyosis were regarded. The focus on individual adenomyosis features allows a more detailed assessment of whether specific imaging findings are associated with clinical pregnancy, live birth, or pregnancy loss. Although our pooled analysis benefits from a large sample size, the findings should be interpreted with caution. The certainty of evidence was very low for 18 of the 21 outcomes assessed using GRADE, reflecting substantial heterogeneity in patient populations, diagnostic criteria for adenomyosis, IVF-treatment protocols and embryo transfer strategies, which may attenuate true associations between specific adenomyosis features and study outcomes. Several pooled estimates were not robust to exclusion of individual studies in sensitivity analysis, further underscoring the fragility of these findings. Formal statistical testing for publication bias was not possible for most comparisons, as fewer than the recommended ten studies contributed to each, and only allowed for visual assessment. Additional limitations include the frequent coexistence of endometriosis (4–71%), which may confound the observed effects of adenomyosis on reproductive outcomes, and the fact that few studies adjusted for confounders such as age or BMI in subgroup analyses.

In conclusion, adenomyosis is a complex disease with expected variable pregnancy outcomes depending on which features are present. While the prognostic relevance of individual features of adenomyosis remains uncertain, documenting specific MUSA criteria, rather than simply noting disease presence, can improve risk assessment. Focussing on adenomyosis features with stronger and more consistent associations with pregnancy outcomes, such as diffuse adenomyosis or lesions involving the junctional zone, may further enhance prognostic accuracy and guide clinical decision-making.

## Contributors

NB conceived the study, contributed to the study protocol design, directly accessed and verified the underlying data reported in the manuscript, co-wrote the manuscript, prepared the tables, contributed to the interpretation of the results, and revised the final manuscript.

CL contributed to the study protocol design, directly accessed and verified the underlying data reported in the manuscript, co-wrote the manuscript, prepared the tables, contributed to the interpretation of the results, and revised the final manuscript.

KHA supervised the development of the study protocol and the data extraction process, contributed to the study protocol design and the interpretation of the results, and revised the final manuscript.

DW performed the meta-analyses, contributed to the study protocol design and the interpretation of the results, and revised the final manuscript.

MA contributed to the study protocol design, interpretation of the results, and final revision of the manuscript. SB contributed to the study protocol design, interpretation of the results, and final revision of the manuscript. GB contributed to the study protocol design, interpretation of the results, and final revision of the manuscript. SJB contributed to the study protocol design, interpretation of the results, and final revision of the manuscript. PR contributed to the study protocol design, interpretation of the results, and final revision of the manuscript. DG contributed to the study protocol design, interpretation of the results, and final revision of the manuscript. HS contributed to the study protocol design, interpretation of the results, and final revision of the manuscript.

All authors read and approved the final version of the manuscript.

## Data sharing statement

All data that contributed to this article are available in the text, or in online Supplementary Files.

The statistical analysis code is available from the corresponding author upon reasonable request.

## Declaration of interests

HSN has received funding from Freya Biosciences ApS, Ferring Pharmaceuticals, BioInnovation Institute, Ministry of Education, Novo Nordisk Foundation, Augustinus Fonden, Oda og Hans Svenningsens Fond, Demant Fonden, Ole Kirks Fond and the Independent Research Fund Denmark, as well as speaker fees from Ferring Pharmaceuticals, Merck A/S, Astra Zeneca, Cook Medical, Gedeon Richter, Ibsa Nordic, Novo Nordisk A/S. HSN has co-developed a safe delivery app and is founder and chairman of the Maternity Foundation as well as co-founder of Lulu Health.

SB has received a speaker's fee for lectures in thyroid in pregnancy at Extempore and travel grants to attend ECE 2025 and ITC 2025 by the Danish Endocrine Society and the Danish Thyroid Association.

NB, CL, GB, SJB, DG, DW, KHA, MSA, and PR have no interests to declare.

## Appendix A Supplementary data

Protocol
