# Supplementary material for: Adenomyosis and association between ultrasonographic features and pregnancy outcomes in women with infertility or pregnancy loss: a systematic review and meta-analysis
Source: eBioMedicine. 2026 Oct 21;133:106518. doi: 10.1016/j.ebiom.2026.106518 (PMC13716959; doi:10.1016/j.ebiom.2026.106518)

Supplementary figures

Contents

Reproductive outcomes: Meta-analyses by design, funnel plots and leave-one-out 2
Fig. A: External AM vs no AM, by design 2
Fig. B: External AM vs internal AM, by design 3
Fig. C: Internal AM vs no AM, by design 4
Fig. D: Focal AM vs no AM, by design 5
Fig. E: Focal AM vs no AM- Leave one out 6
Fig. F: Diffuse AM vs focal AM, by design 7
Fig. G: Diffuse AM vs focal AM - Leave one out 8
Fig. H: High vs low uterus volume, by design 9
Fig. I: High vs low uterus volume - Leave one out 10
Fig. J: Diffuse AM vs no AM, by design 11
Fig. K: Diffuse AM vs no AM - Leave one out 12
Prevalence estimates: Meta-analyses, funnel plots and leave-one-out 13
Fig. L: Myometrial cysts 13
Fig. M: Hyperechogenic islands 14
Fig. N: Lines and buds 15
Fig. O: Asymmetrical thickening 16
Fig. P: Globular uterus 17
Fig. Q: Translesional vascularity 18
Fig. R: Fan-shaped shadowing 19
Fig. S: Interrupted junction zone 20
Fig. T: Irregular junction zone 21

Fig. A: External AM vs no AM, by design

Clinical pregnancy

Study	External AM Events	External AM Total	No AM Events	No AM Total	OR	95% CI	W(common)	W(random)
**Prospective**								
Sharma 2025	71	167	76	217	1.37	[0.91; 2.08]	35.5%	37.9%
**Retrospective**								
Wang 2025	270	476	116	215	1.12	[0.81; 1.55]	64.5%	62.1%
**Common effect model**		**643**		**432**	**1.21**	**[0.94; 1.56]**	**100.0%**	**.**
**Random effects model**					**1.21**	**[0.34; 4.26]**	**.**	**100.0%**

Odds ratio (axis: 0.5, 1, 2)

Heterogeneity: $I^2 = 0.0\%$
Test for subgroup differences (common effect): $\chi^2_1 = 0.58$, df = 1 ($p = 0.4466$)
Test for subgroup differences (random effects): $\chi^2_1 = 0.58$, df = 1 ($p = 0.4466$)

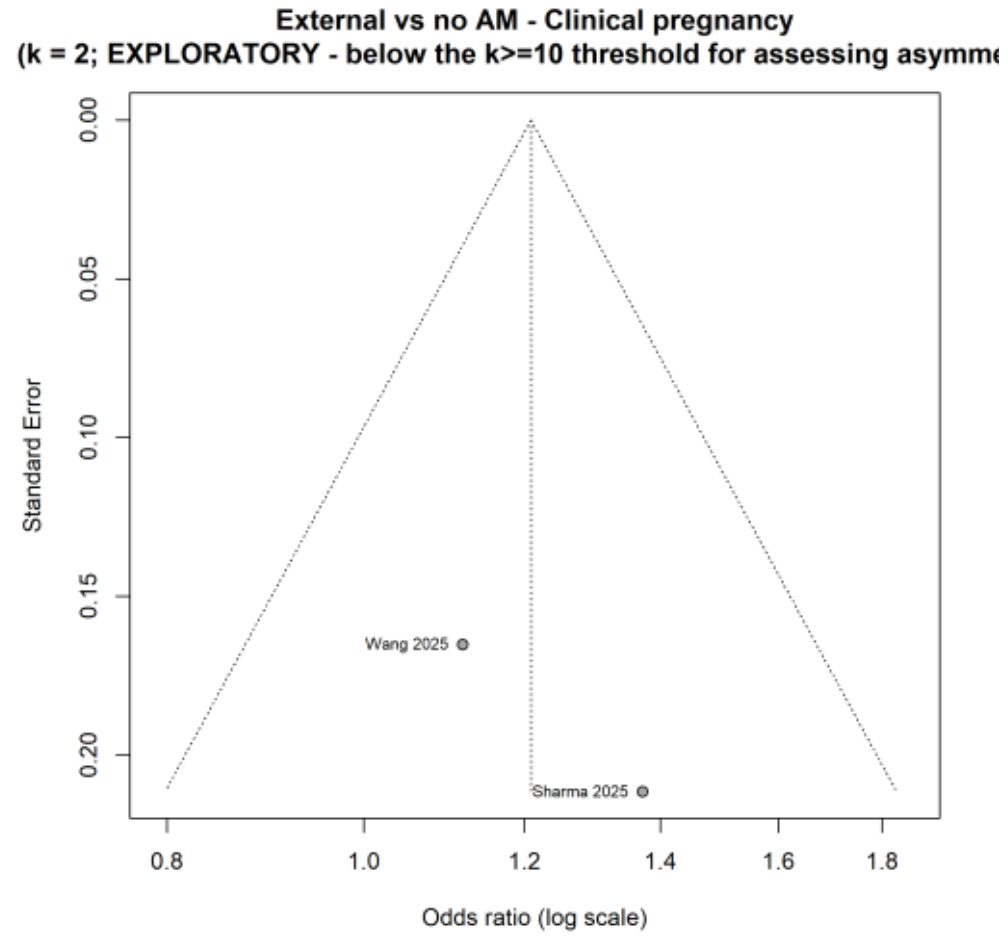

Live birth

Study	External AM Events	External AM Total	No AM Events	No AM Total	OR	95% CI	W(common)	W(random)
**Prospective**								
Sharma 2025	43	167	65	217	0.81	[0.52; 1.27]	38.2%	39.5%
**Retrospective**								
Wang 2025	218	476	91	215	1.15	[0.83; 1.59]	61.8%	60.5%
**Common effect model**		**643**		**432**	**1.02**	**[0.79; 1.33]**	**100.0%**	**.**
**Random effects model**					**1.00**	**[0.11; 8.85]**	**.**	**100.0%**

Odds ratio (axis: 0.2, 0.5, 1, 2, 5)

Heterogeneity: $I^2 = 34.2\%$
Test for subgroup differences (common effect): $\chi^2_1 = 1.52$, df = 1 ($p = 0.2175$)
Test for subgroup differences (random effects): $\chi^2_1 = 1.52$, df = 1 ($p = 0.2175$)

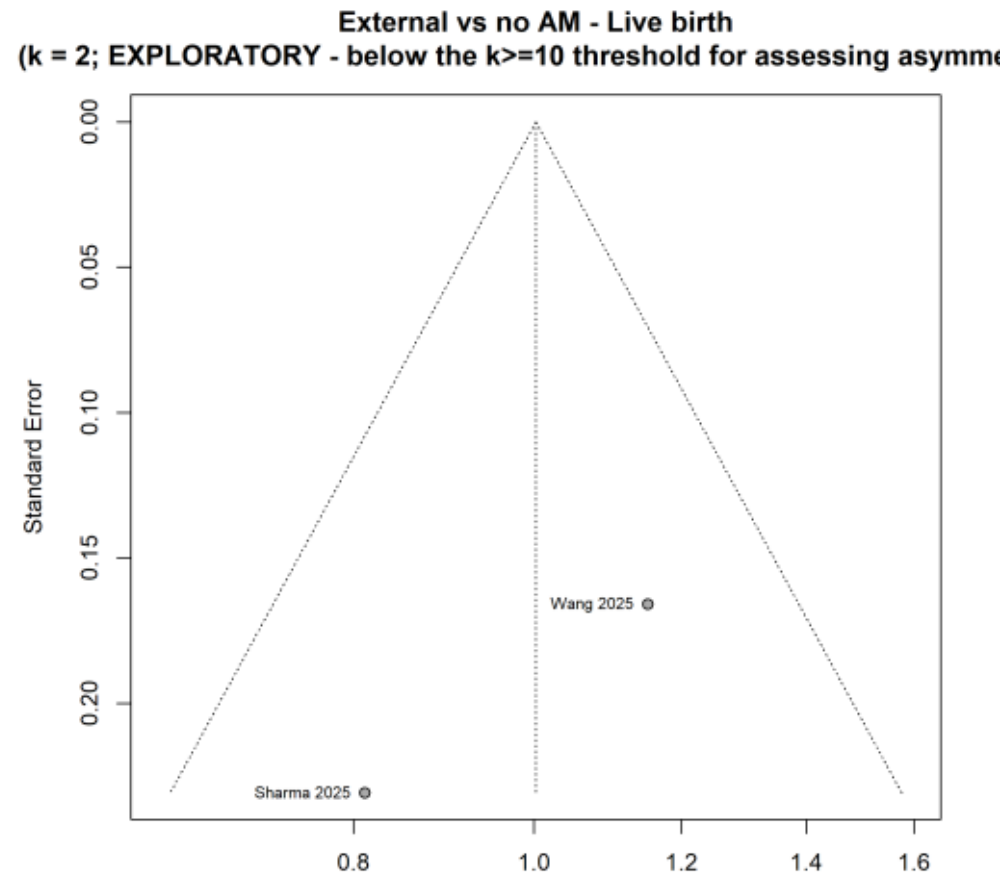

Pregnancy loss

Study	External AM Events	External AM Total	No AM Events	No AM Total	OR	95% CI	W(common)	W(random)
**Prospective**								
Sharma 2025	15	71	7	76	2.64	[1.01; 6.92]	17.3%	43.1%
**Retrospective**								
Wang 2025	47	270	22	116	0.90	[0.51; 1.58]	82.7%	56.9%
**Common effect model**		**341**		**192**	**1.20**	**[0.74; 1.95]**	**100.0%**	**.**
**Random effects model**					**1.43**	**[0.00; 1245.26]**	**.**	**100.0%**

Odds ratio (axis: 0.001, 0.1, 1, 10, 1000)

Heterogeneity: $I^2 = 72.1\%$ [0.0%; 93.7%]
Test for subgroup differences (common effect): $\chi^2_1 = 3.57$, df = 1 ($p = 0.0587$)
Test for subgroup differences (random effects): $\chi^2_1 = 3.57$, df = 1 ($p = 0.0587$)

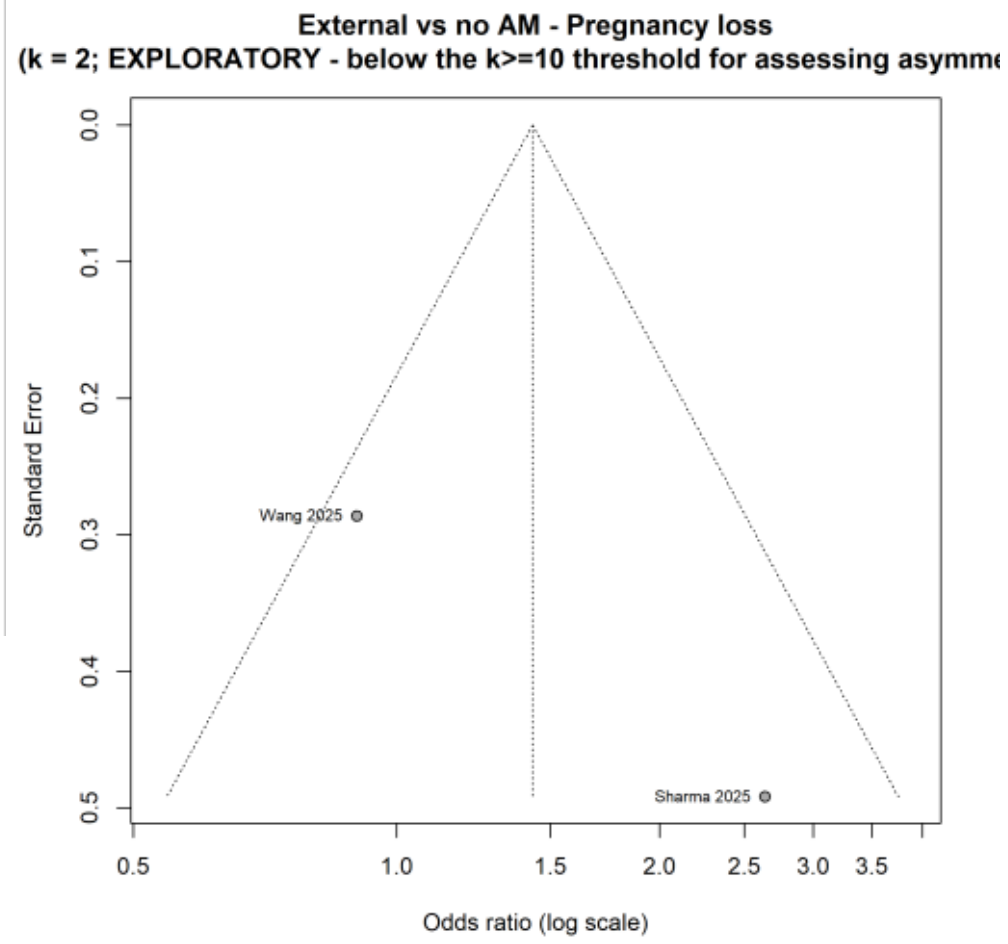

Fig. B: External AM vs internal AM, by design

Clinical pregnancy

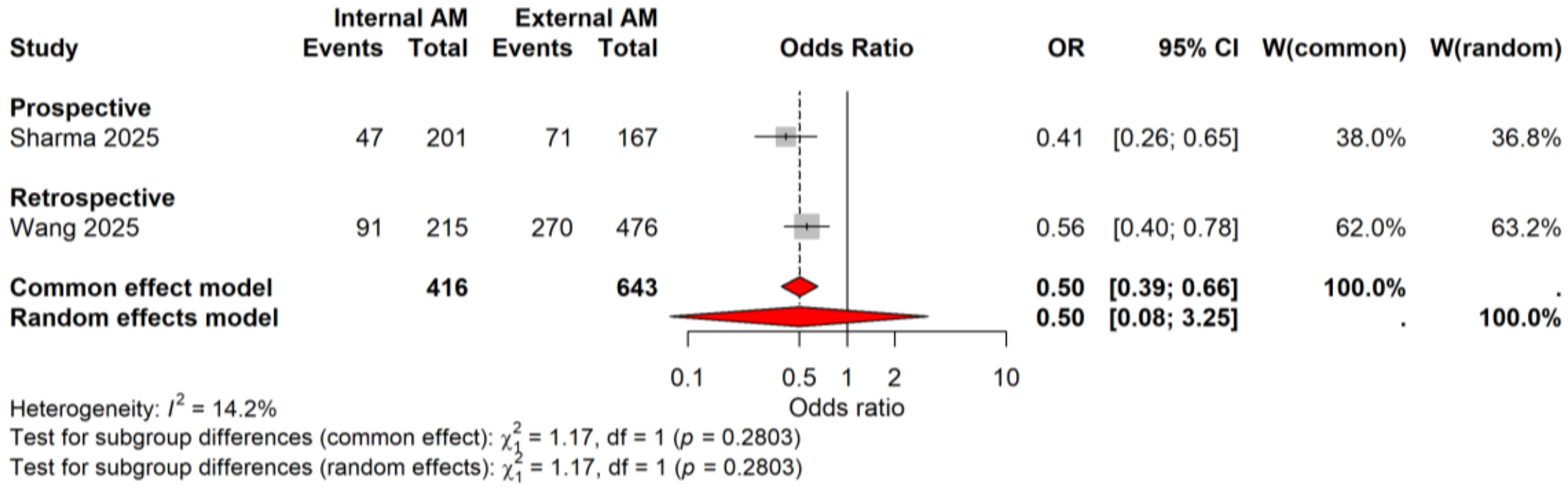

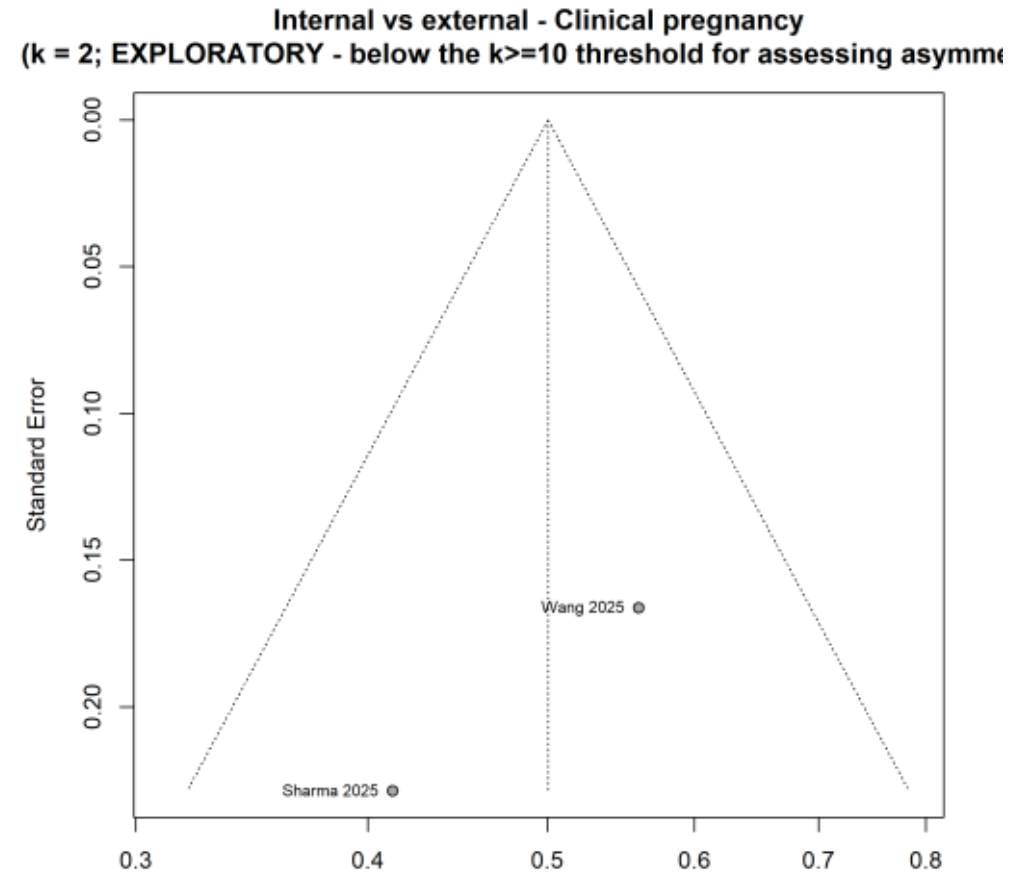

Live birth

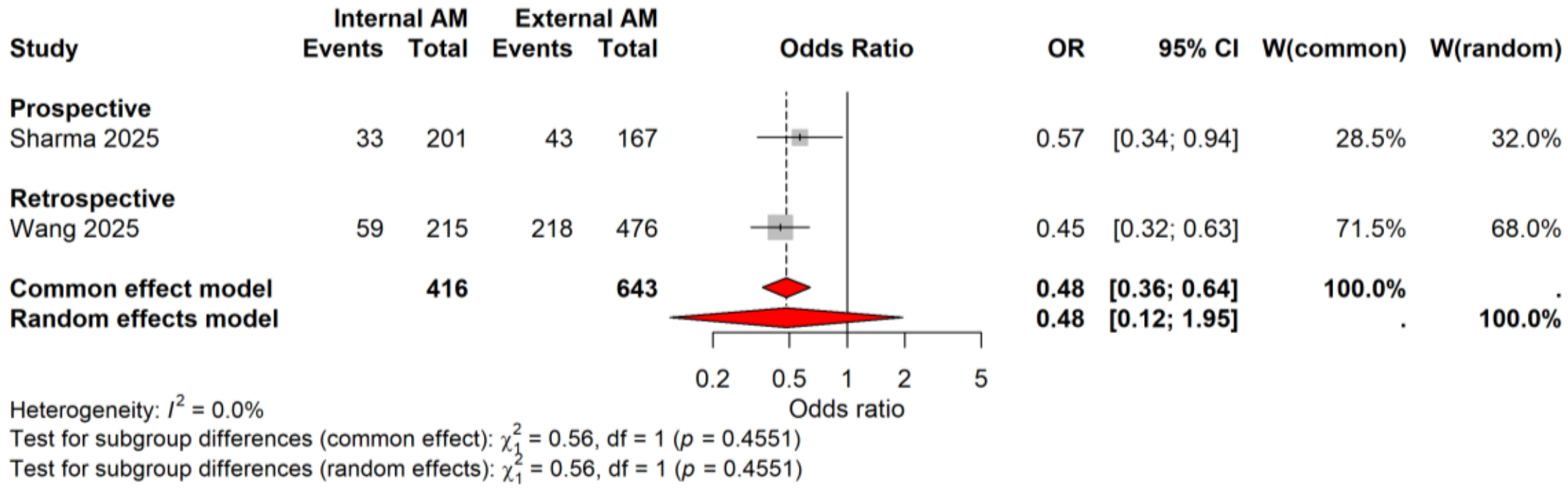

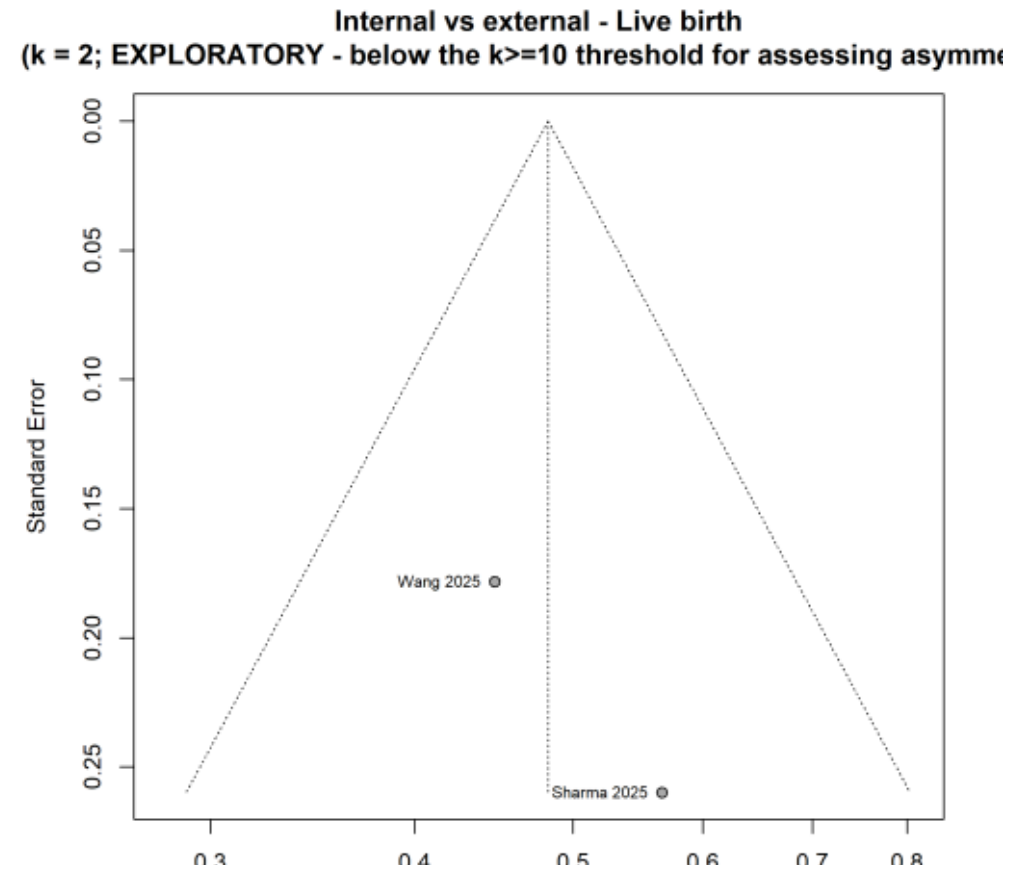

Pregnancy loss

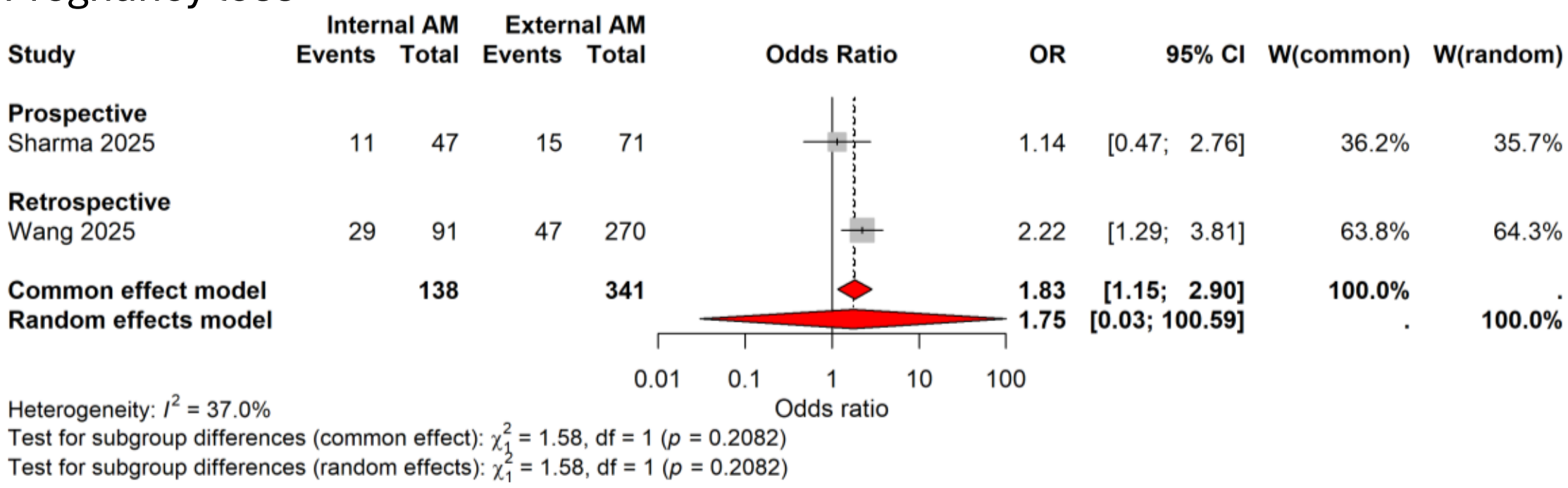

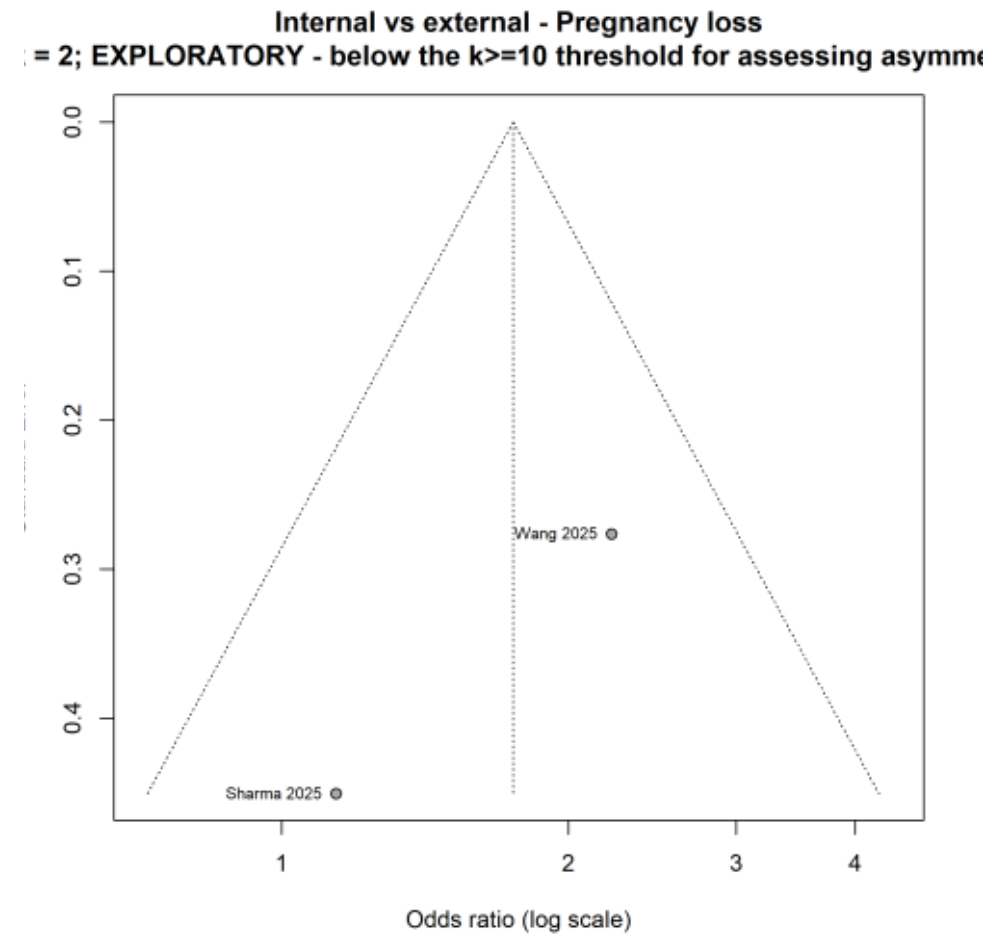

Fig. C: Internal AM vs no AM, by design

Clinical pregnancy

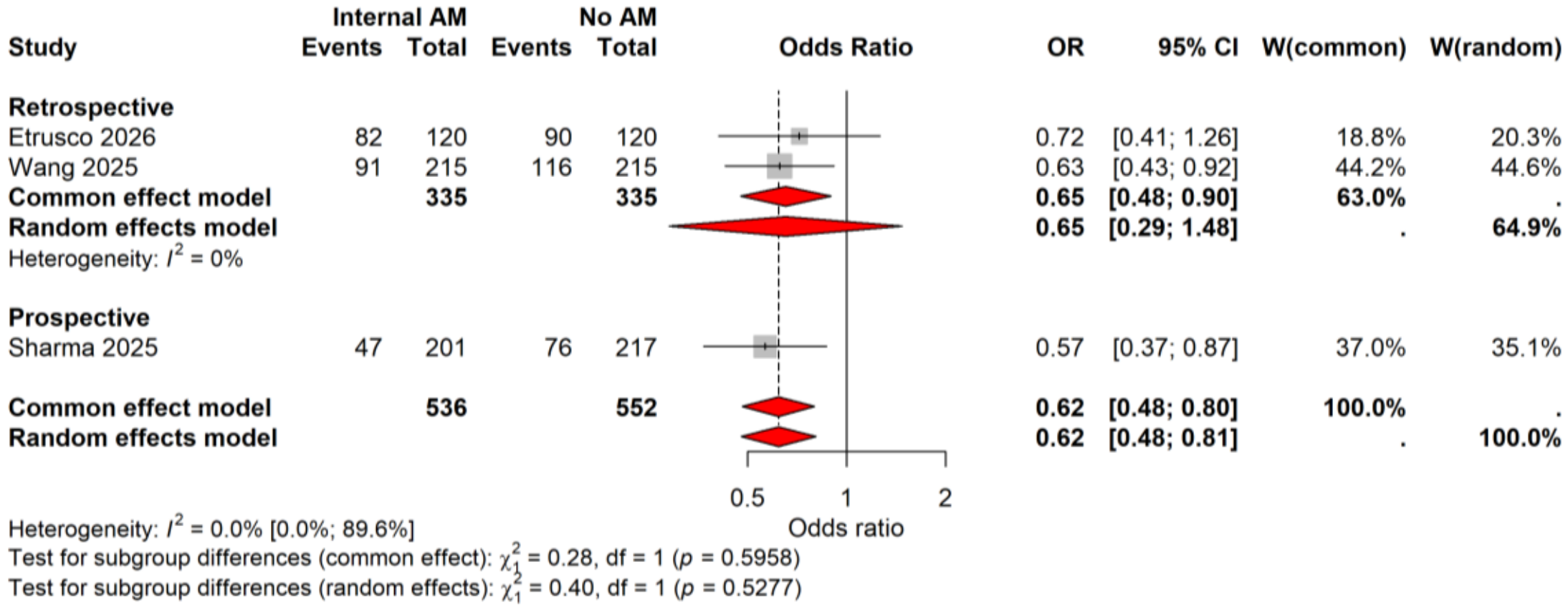

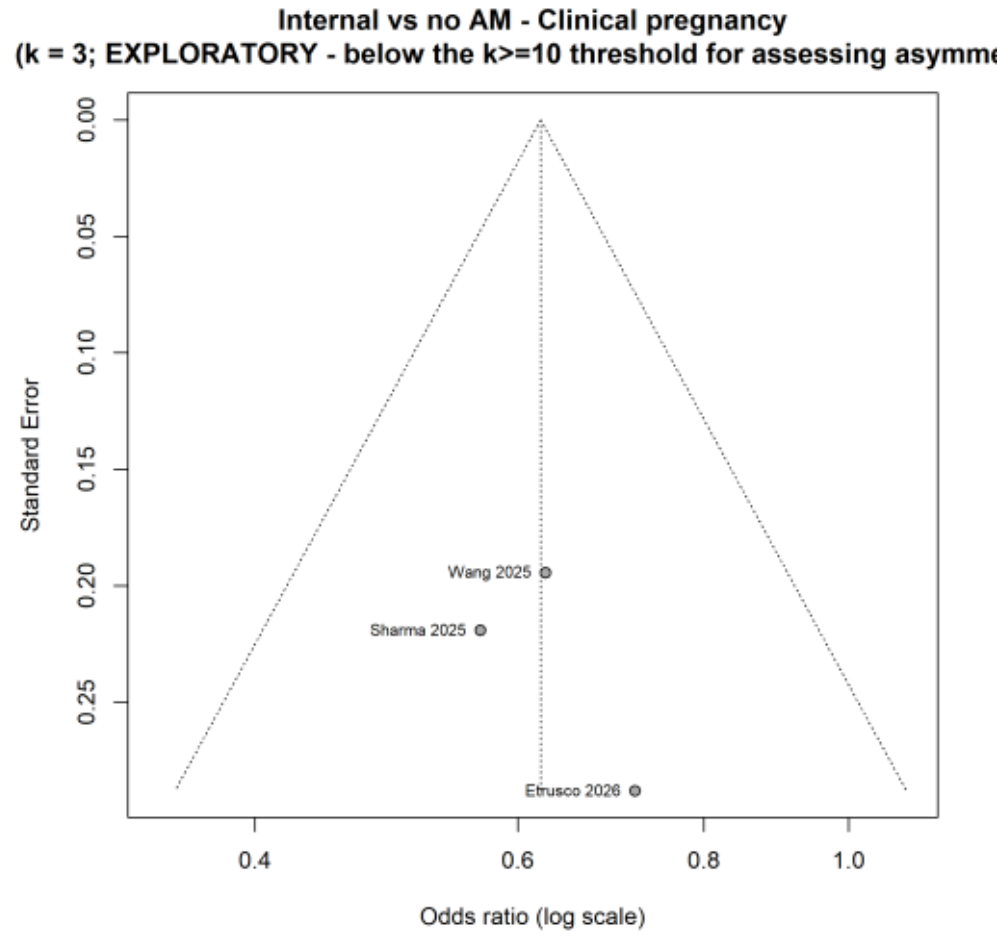

Live birth

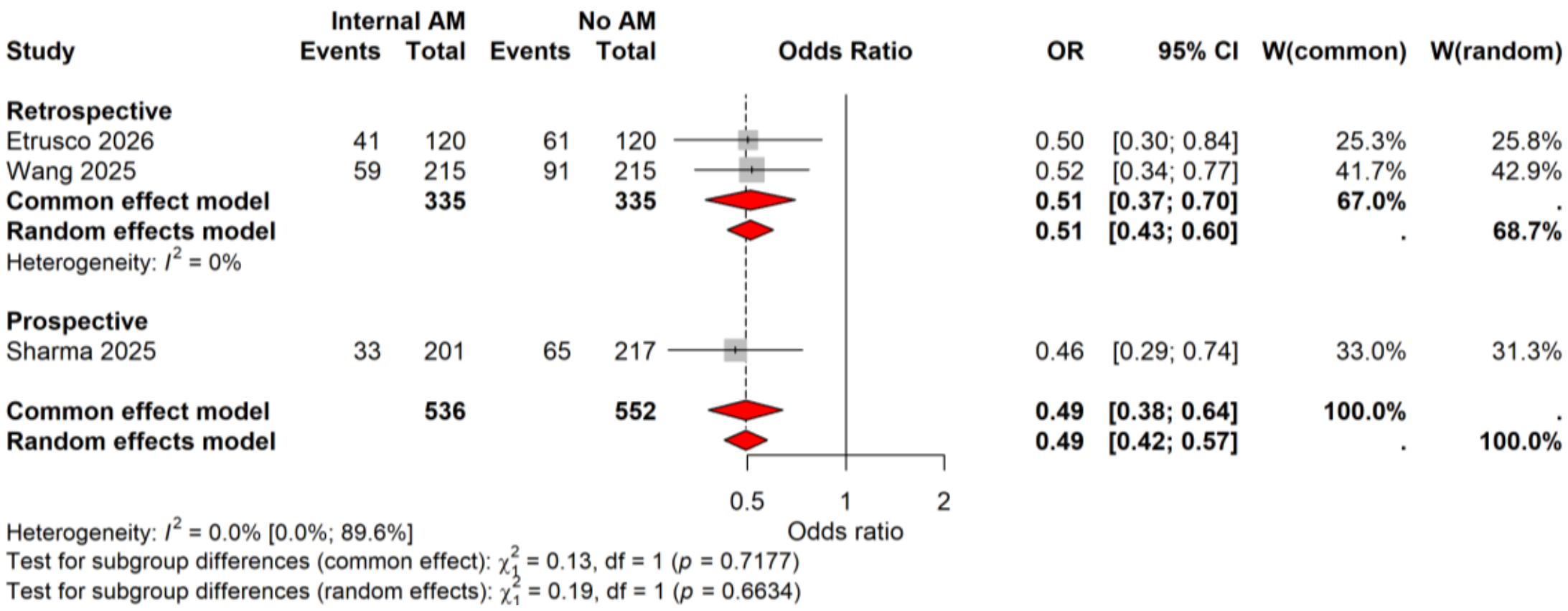

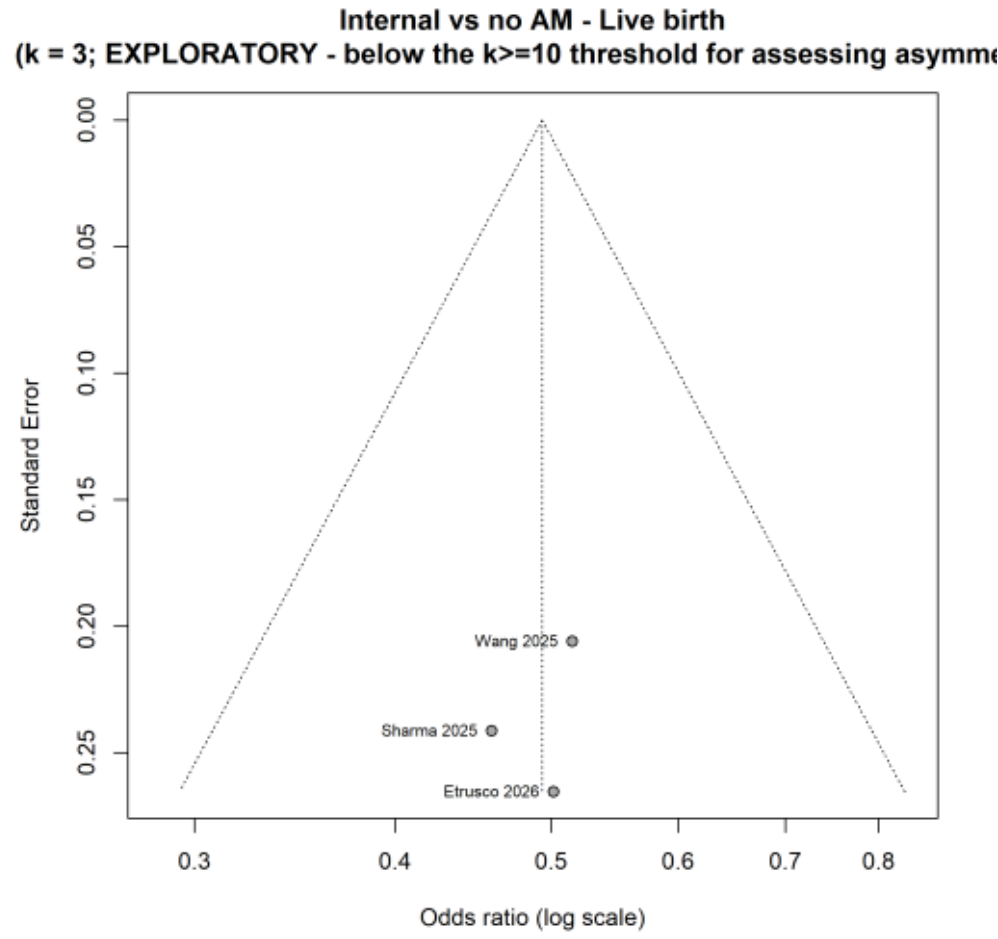

Pregnancy loss

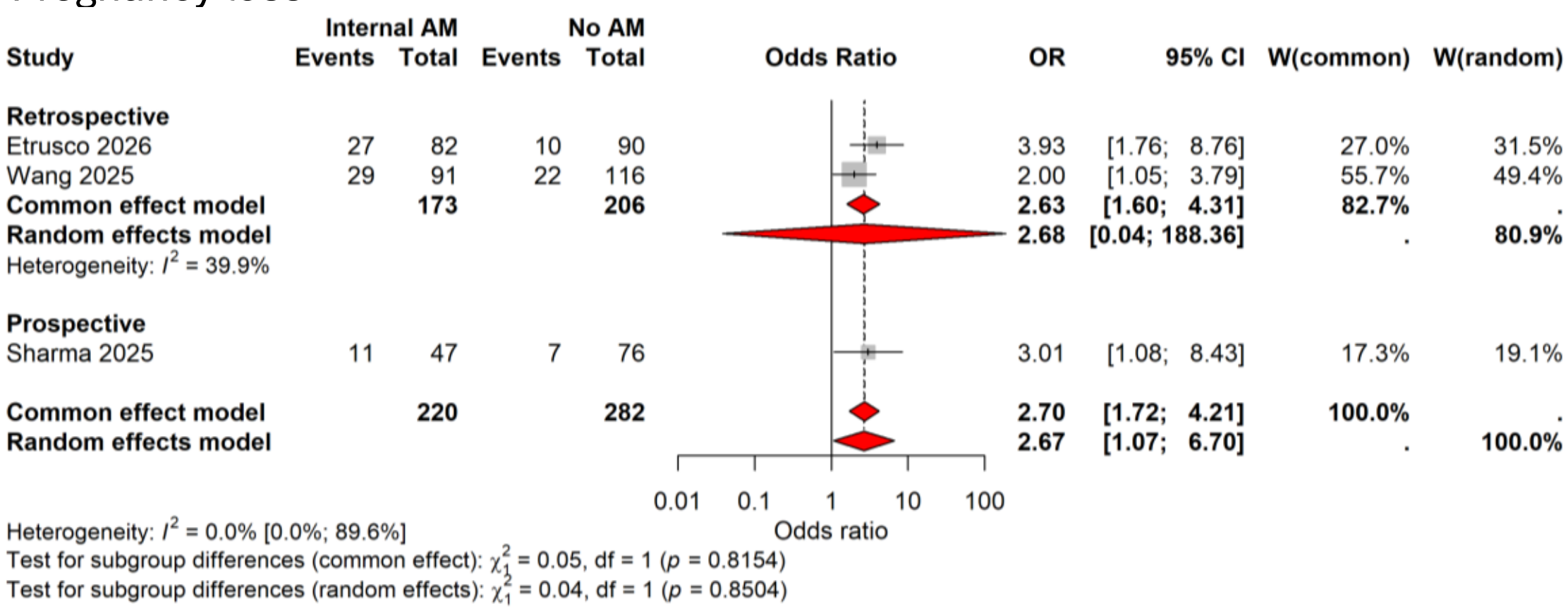

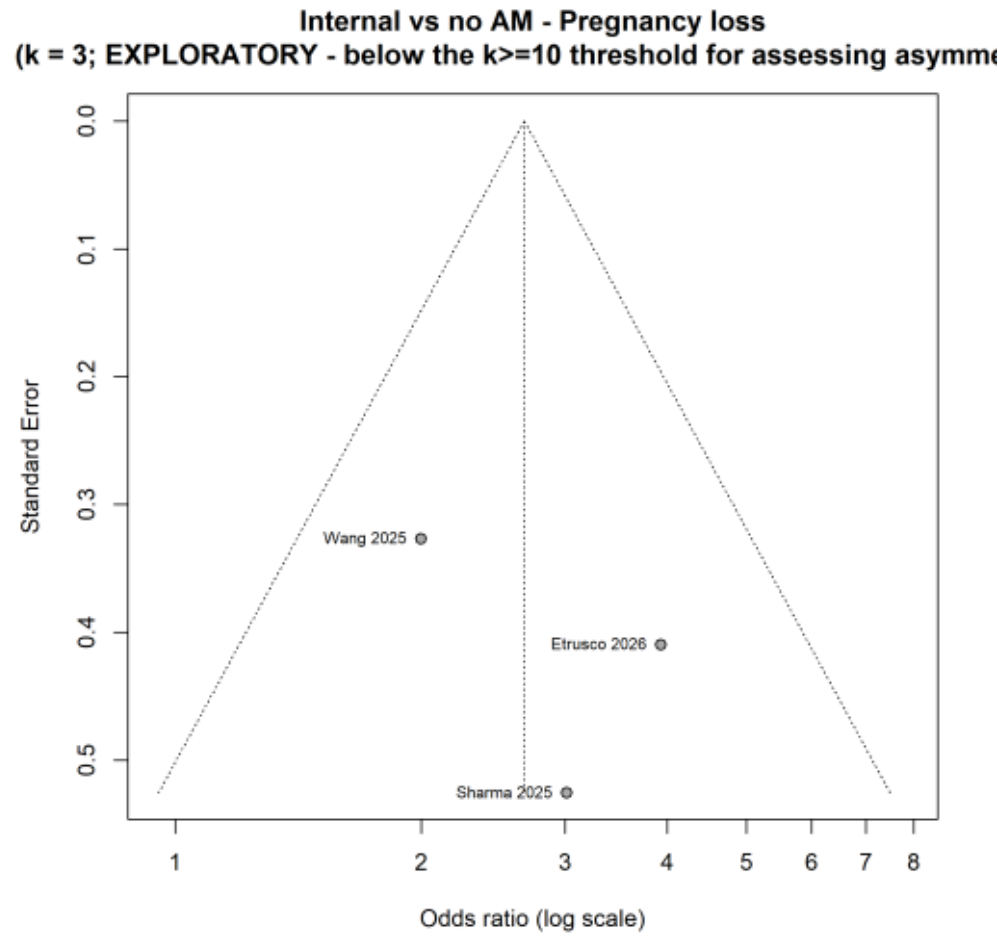

Fig. D: Focal AM vs no AM, by design

Clinical pregnancy

Study	Focal AM Events	Focal AM Total	No AM Events	No AM Total	OR	95% CI	W(common)	W(random)
**Prospective**								
Benaglia 2014	11	24	14	49	2.12	[0.77; 5.83]	2.2%	16.3%
**Retrospective**								
Han 2023	298	718	246	519	0.79	[0.63; 0.99]	74.2%	36.4%
Trinchant 2022	10	24	1869	3128	0.48	[0.21; 1.09]	7.4%	20.5%
Trinchant 2025	23	62	136	228	0.40	[0.22; 0.71]	16.2%	26.8%
**Common effect model**		**804**		**3875**	**0.70**	**[0.57; 0.86]**	**97.8%**	.
**Random effects model**					**0.58**	**[0.23; 1.47]**	.	**83.7%**
Heterogeneity: I^2 = 63.4% [0.0%; 89.5%]								
**Common effect model**		**828**		**3924**	**0.73**	**[0.60; 0.89]**	**100.0%**	.
**Random effects model**					**0.70**	**[0.25; 1.96]**	.	**100.0%**

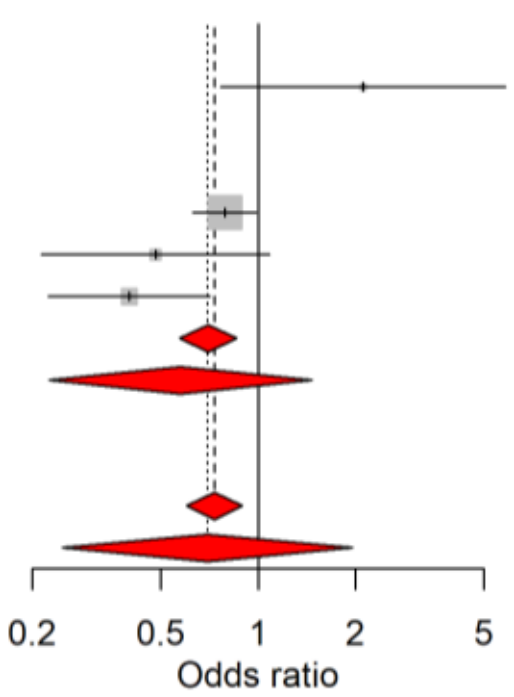

Heterogeneity: I^2 = 69.5% [12.1%; 89.4%]
Test for subgroup differences (common effect): χ^2_1 = 4.39, df = 1 (p = 0.0361)
Test for subgroup differences (random effects): χ^2_1 = 5.38, df = 1 (p = 0.0204)

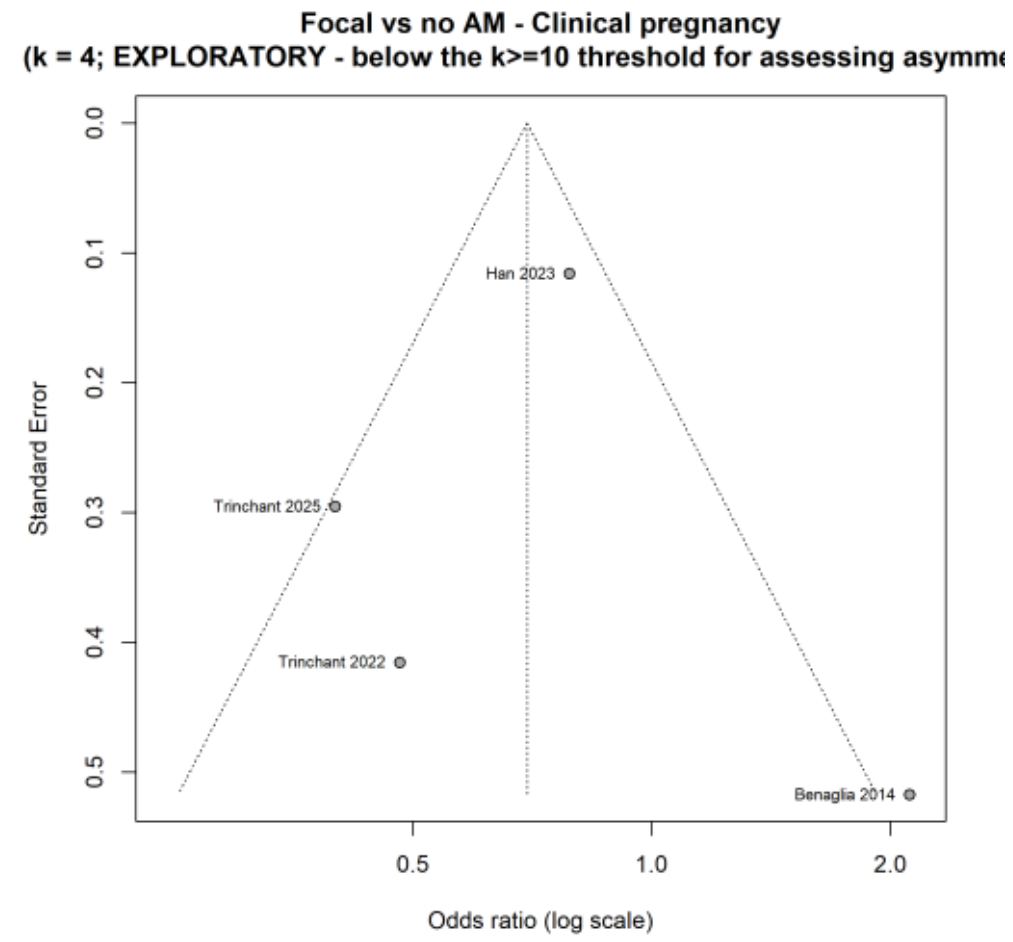

Live birth

Study	Focal AM Events	Focal AM Total	No AM Events	No AM Total	OR	95% CI	W(common)	W(random)
**Prospective**								
Benaglia 2014	8	24	18	49	0.86	[0.31; 2.41]	3.4%	18.6%
**Retrospective**								
Han 2023	217	718	208	519	0.65	[0.51; 0.82]	72.5%	35.6%
Trinchant 2022	7	24	1560	3128	0.41	[0.17; 1.00]	7.2%	21.5%
Trinchant 2025	9	62	107	228	0.19	[0.09; 0.41]	16.8%	24.3%
**Common effect model**		**804**		**3875**	**0.55**	**[0.44; 0.68]**	**96.6%**	.
**Random effects model**					**0.39**	**[0.08; 1.87]**	.	**81.4%**
Heterogeneity: I^2 = 79.4% [34.5%; 93.5%]								
**Common effect model**		**828**		**3924**	**0.56**	**[0.45; 0.69]**	**100.0%**	.
**Random effects model**					**0.46**	**[0.17; 1.27]**	.	**100.0%**

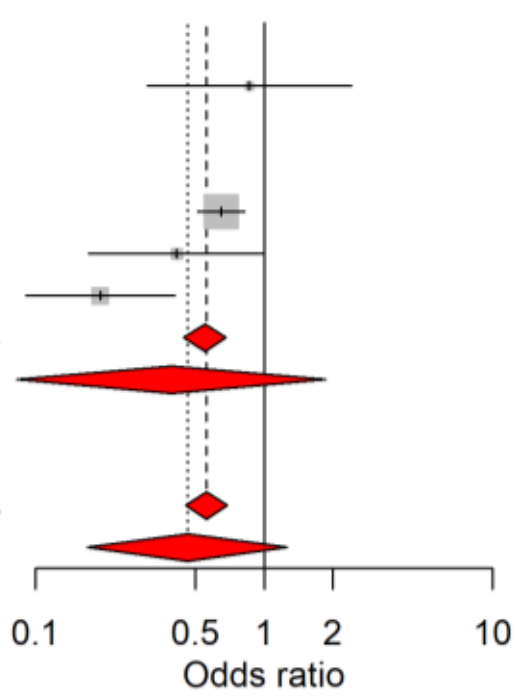

Heterogeneity: I^2 = 70.9% [16.9%; 89.8%]
Test for subgroup differences (common effect): χ^2_1 = 0.70, df = 1 (p = 0.4043)
Test for subgroup differences (random effects): χ^2_1 = 1.51, df = 1 (p = 0.2189)

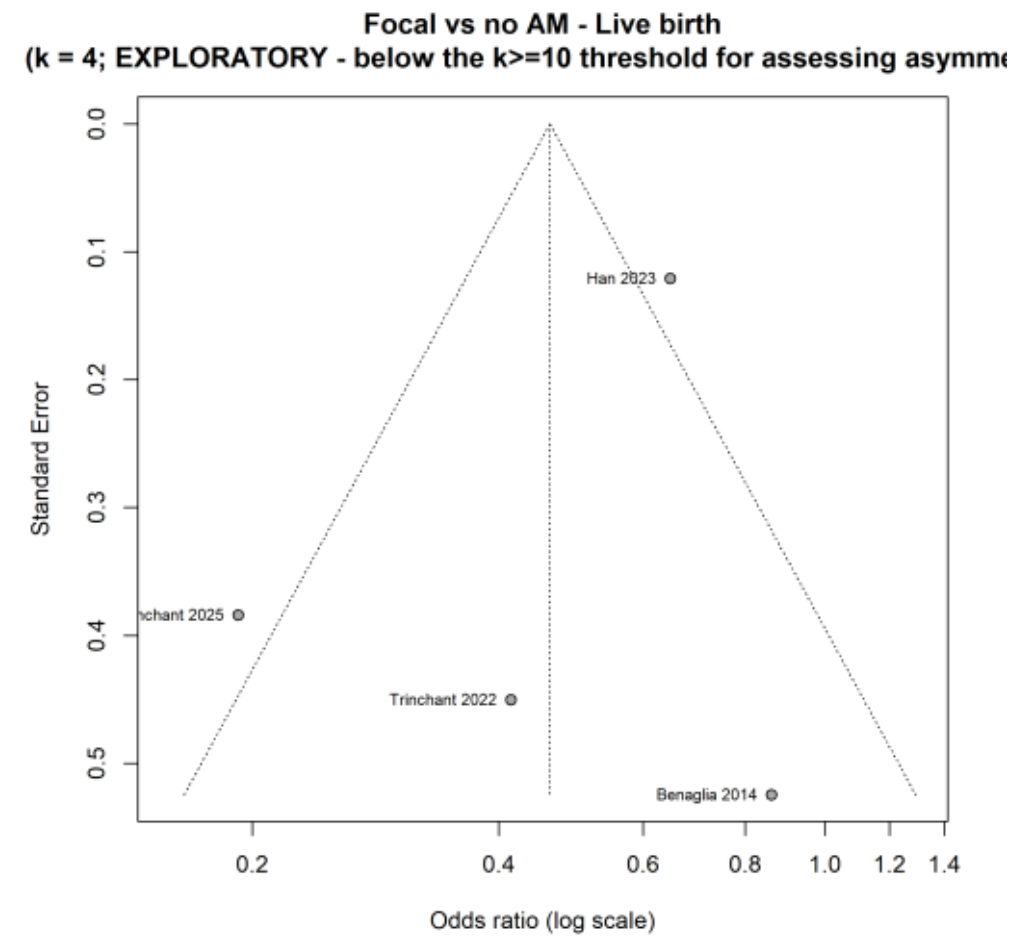

Pregnancy loss

Study	Focal AM Events	Focal AM Total	No AM Events	No AM Total	OR	95% CI	W(common)	W(random)
**Prospective**								
Benaglia 2014	2	10	5	14	0.45	[0.07; 3.00]	7.8%	10.7%
Cozzolino 2024	8	25	11	56	1.93	[0.66; 5.60]	10.8%	20.2%
**Common effect model**		**35**		**70**	**1.31**	**[0.53; 3.25]**	**18.6%**	.
**Random effects model**					**1.16**	**[0.00; 7723.65]**	.	**31.0%**
Heterogeneity: I^2 = 41.8%								
**Retrospective**								
Han 2023	81	298	38	246	2.04	[1.33; 3.14]	70.8%	31.0%
Trinchant 2022	3	10	309	1869	2.16	[0.56; 8.41]	5.4%	16.1%
Trinchant 2025	14	23	20	136	9.02	[3.45; 23.62]	5.3%	21.9%
**Common effect model**		**331**		**2251**	**2.50**	**[1.72; 3.65]**	**81.4%**	.
**Random effects model**					**3.35**	**[0.42; 26.98]**	.	**69.0%**
Heterogeneity: I^2 = 74.1% [13.5%; 92.2%]								
**Common effect model**		**366**		**2321**	**2.28**	**[1.61; 3.22]**	**100.0%**	.
**Random effects model**					**2.40**	**[0.75; 7.70]**	.	**100.0%**

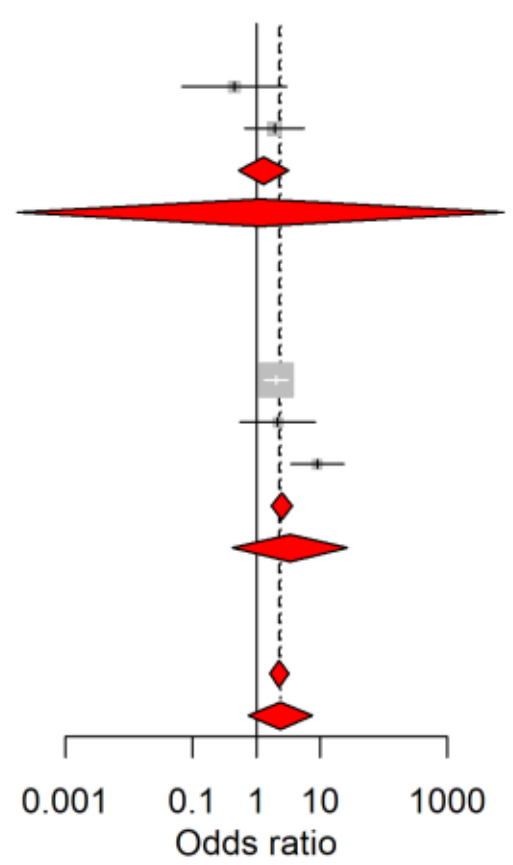

Heterogeneity: I^2 = 63.7% [4.2%; 86.2%]
Test for subgroup differences (common effect): χ^2_1 = 1.67, df = 1 (p = 0.1961)
Test for subgroup differences (random effects): χ^2_1 = 1.58, df = 1 (p = 0.2090)

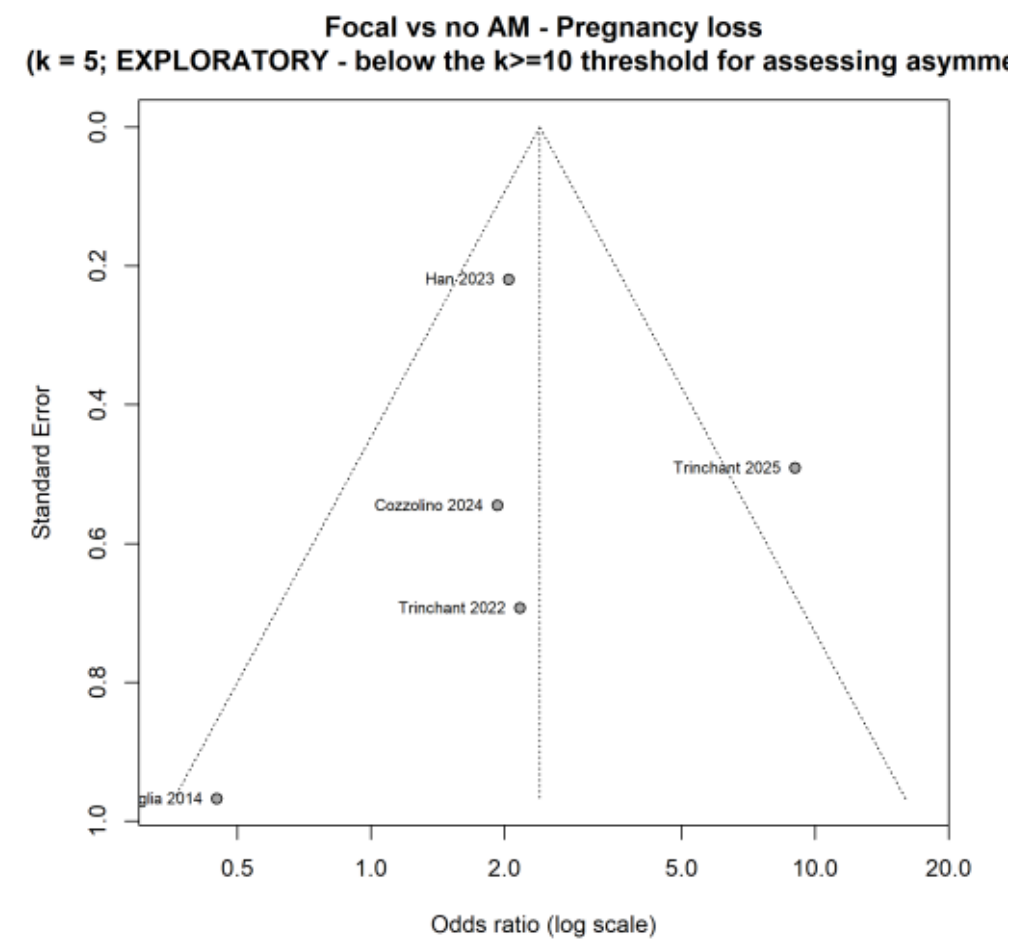

Fig. E: Focal AM vs no AM

Leave one out

Clinical pregnancy

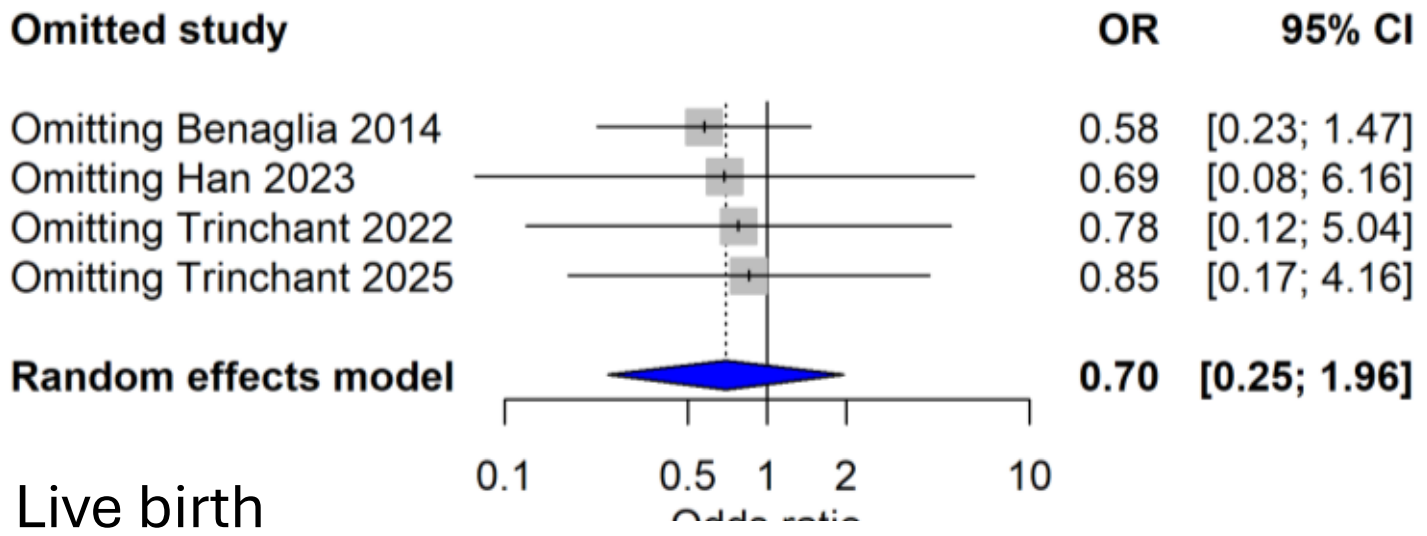

Live birth

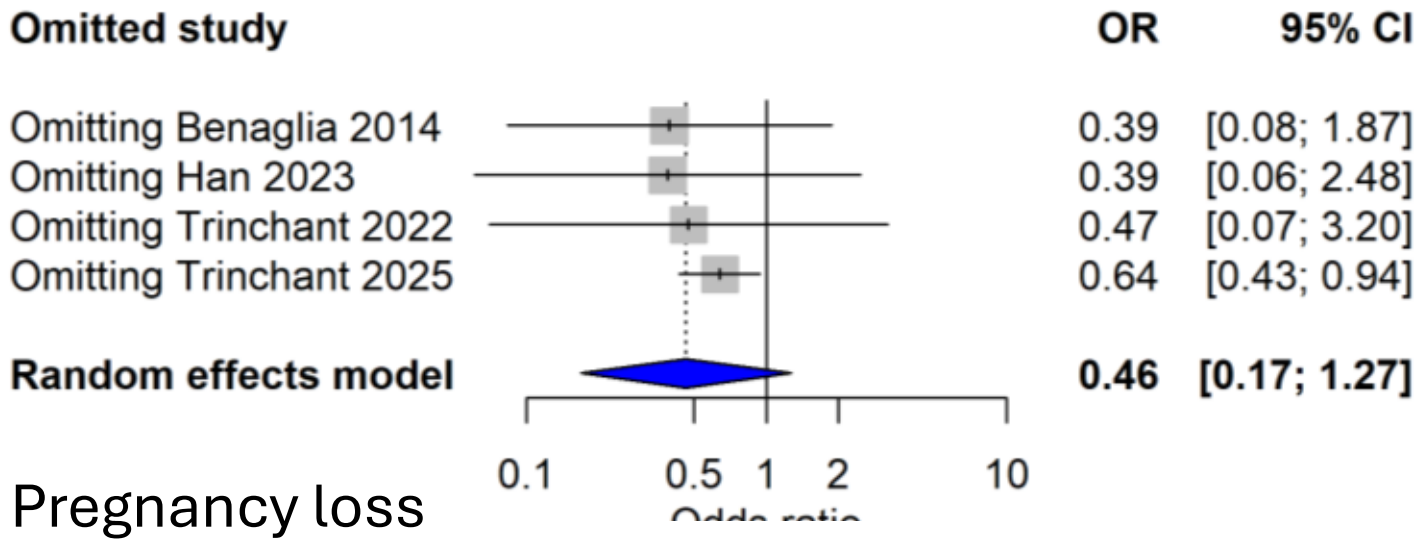

Pregnancy loss

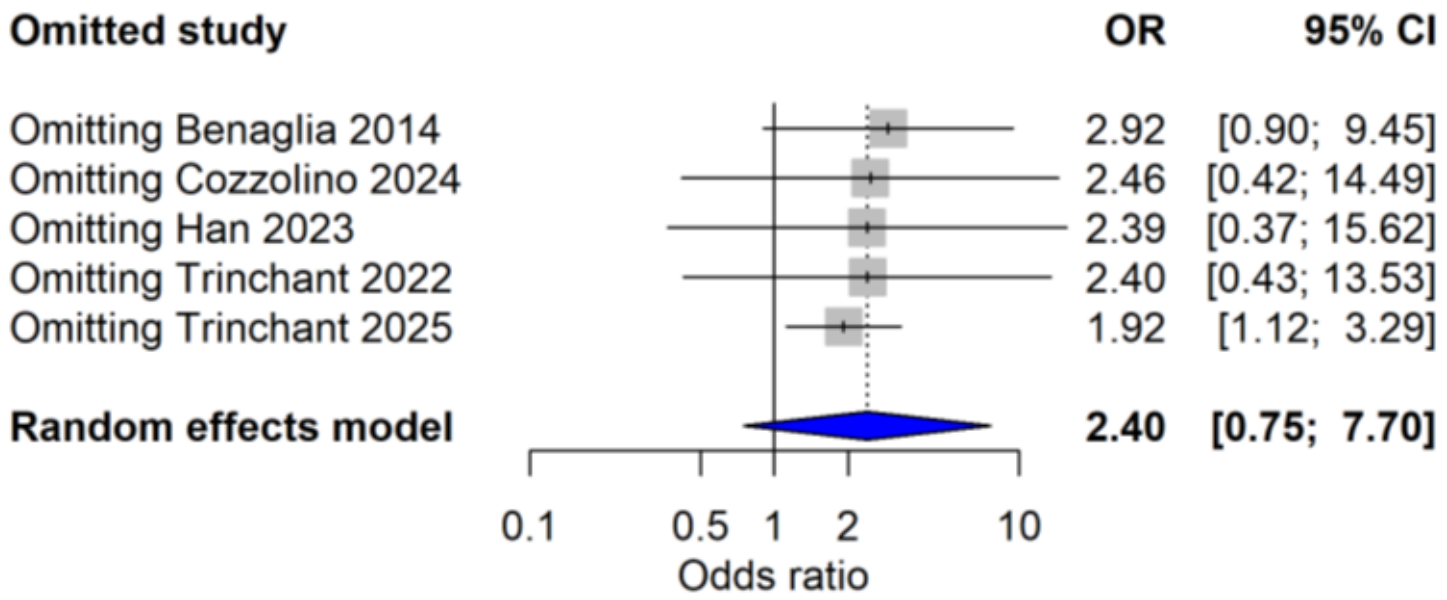

Fig. F: Diffuse AM vs focal AM, by design

Clinical pregnancy

Study	Diffuse AM Events	Diffuse AM Total	Focal AM Events	Focal AM Total	OR	95% CI	W(common)	W(random)
**Prospective**								
Benaglia 2014	10	25	11	24	0.79	[0.25; 2.45]	3.4%	9.4%
**Retrospective**								
Han 2023	139	428	298	718	0.68	[0.53; 0.87]	74.8%	37.1%
Park 2016	16	55	33	93	0.75	[0.36; 1.53]	8.7%	17.7%
Trinchant 2022	72	155	10	24	1.21	[0.51; 2.90]	4.6%	13.9%
Trinchant 2025	81	166	23	62	1.62	[0.89; 2.94]	8.5%	21.7%
**Common effect model**		**804**		**897**	**0.79**	**[0.64; 0.98]**	**96.6%**	.
**Random effects model**					**0.95**	**[0.48; 1.86]**	.	**90.6%**
Heterogeneity: I^2 = 61.9% [0.0%; 87.2%]								
**Common effect model**		**829**		**921**	**0.79**	**[0.64; 0.98]**	**100.0%**	.
**Random effects model**					**0.92**	**[0.56; 1.50]**	.	**100.0%**

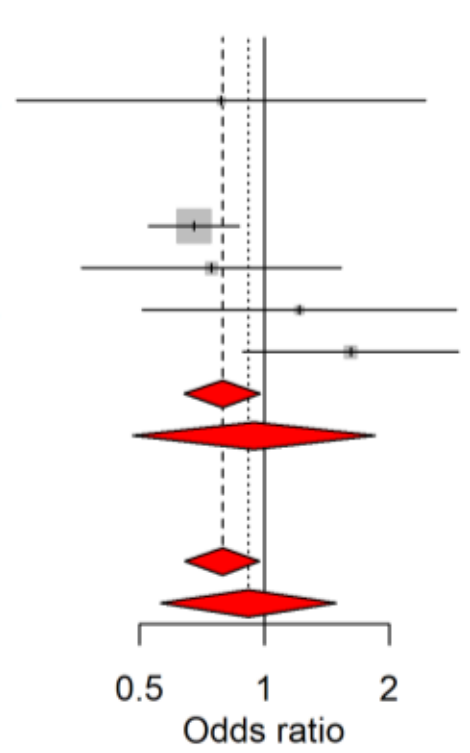

Heterogeneity: I^2 = 49.3% [0.0%; 81.4%]
Test for subgroup differences (common effect): χ^2_1 = 0.00, df = 1 (p = 0.9921)
Test for subgroup differences (random effects): χ^2_1 = 0.09, df = 1 (p = 0.7675)

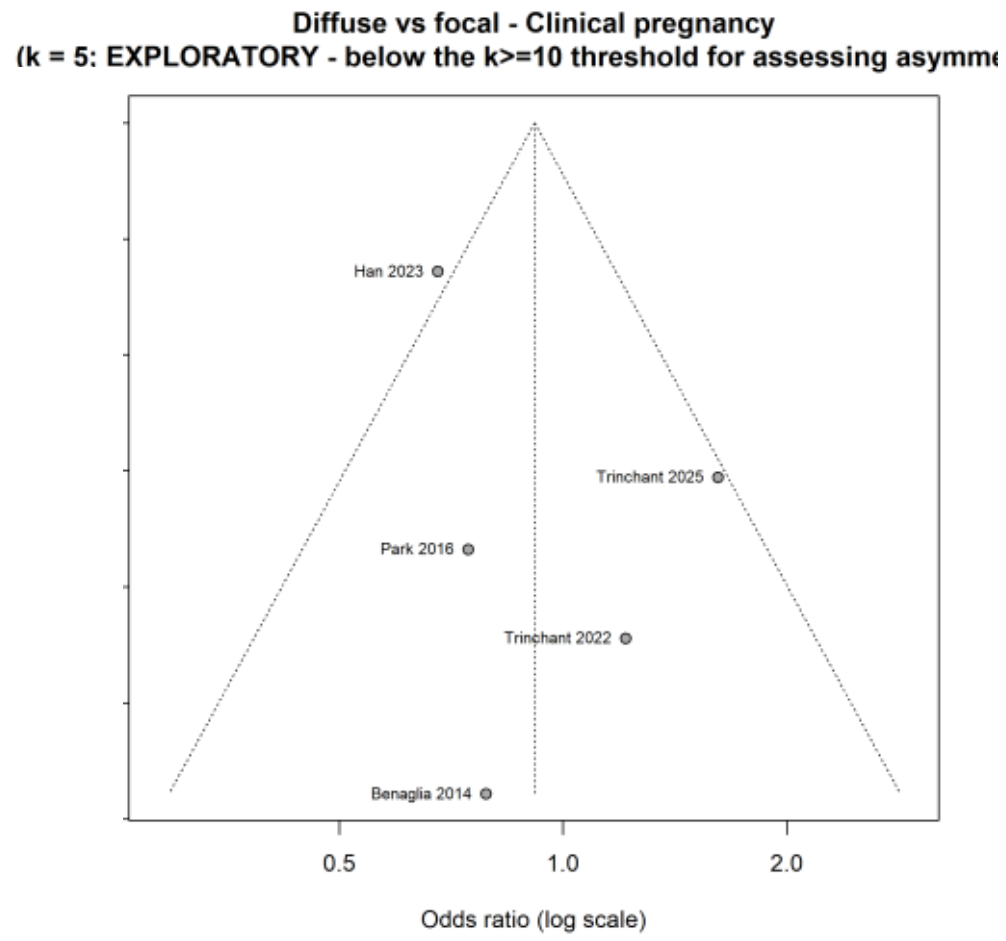

Live birth

Study	Diffuse AM Events	Diffuse AM Total	Focal AM Events	Focal AM Total	OR	95% CI	W(common)	W(random)
**Prospective**								
Benaglia 2014	9	25	8	24	1.12	[0.35; 3.65]	3.1%	15.2%
**Retrospective**								
Han 2023	80	428	217	718	0.53	[0.40; 0.71]	77.7%	26.9%
Park 2016	8	55	25	93	0.46	[0.19; 1.11]	9.4%	19.1%
Trinchant 2022	60	155	7	24	1.53	[0.60; 3.92]	4.4%	18.3%
Trinchant 2025	47	166	9	62	2.33	[1.06; 5.09]	5.5%	20.5%
**Common effect model**		**804**		**897**	**0.67**	**[0.53; 0.85]**	**96.9%**	.
**Random effects model**					**0.93**	**[0.26; 3.27]**	.	**84.8%**
Heterogeneity: I^2 = 81.1% [50.5%; 92.8%]								
**Common effect model**		**829**		**921**	**0.69**	**[0.54; 0.87]**	**100.0%**	.
**Random effects model**					**0.95**	**[0.40; 2.29]**	.	**100.0%**

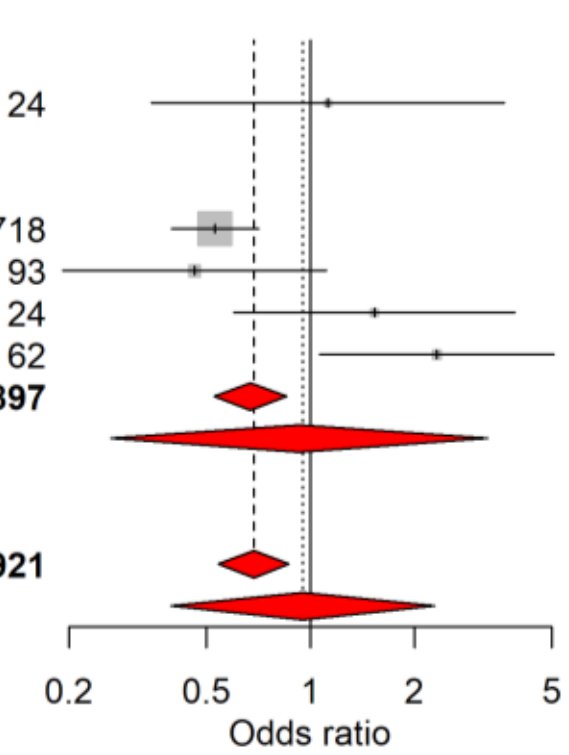

Heterogeneity: I^2 = 75.9% [41.1%; 90.2%]
Test for subgroup differences (common effect): χ^2_1 = 0.71, df = 1 (p = 0.4009)
Test for subgroup differences (random effects): χ^2_1 = 0.07, df = 1 (p = 0.7921)

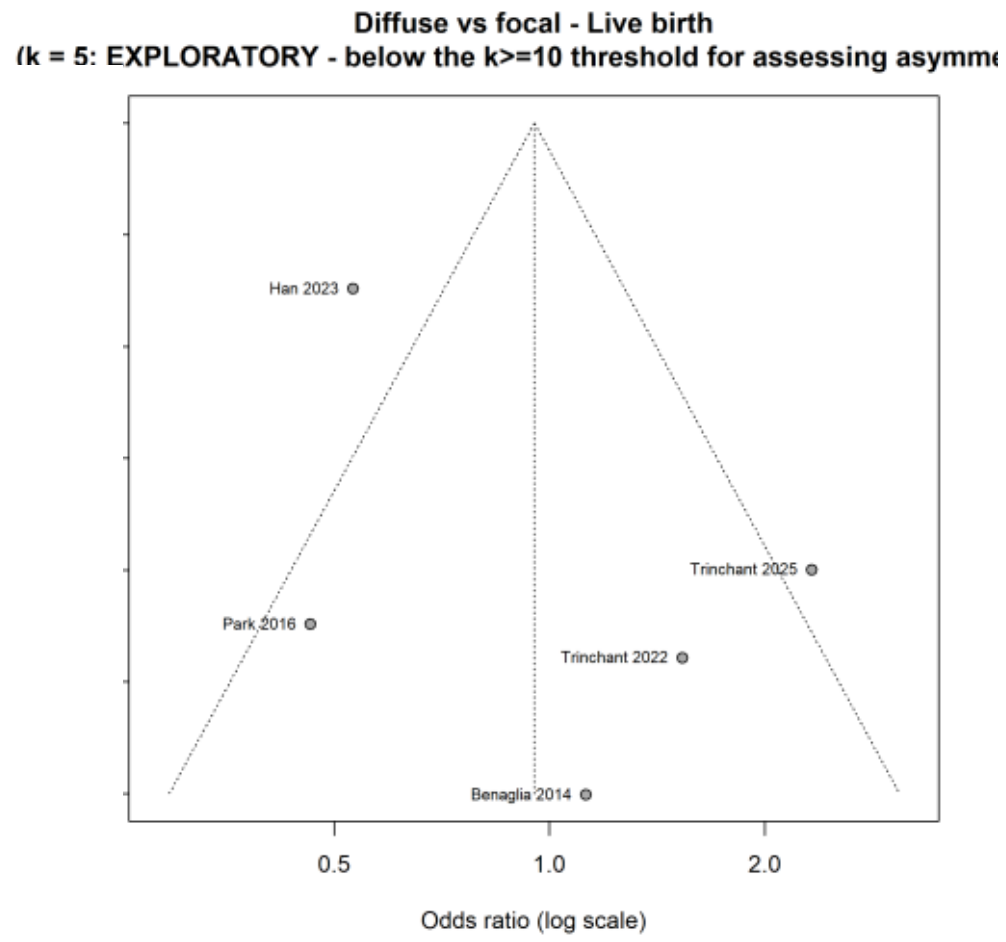

Pregnancy loss

Study	Diffuse AM Events	Diffuse AM Total	Focal AM Events	Focal AM Total	OR	95% CI	W(common)	W(random)
**Prospective**								
Benaglia 2014	2	11	2	10	0.89	[0.10; 7.86]	3.0%	7.1%
Cozzolino 2024	13	34	8	25	1.32	[0.44; 3.91]	9.9%	17.3%
**Common effect model**		**45**		**35**	**1.22**	**[0.46; 3.22]**	**12.9%**	.
**Random effects model**					**1.22**	**[0.17; 8.91]**	.	**24.4%**
Heterogeneity: I^2 = 0%								
**Retrospective**								
Han 2023	58	139	81	298	1.92	[1.26; 2.93]	52.3%	28.9%
Park 2016	8	16	8	33	3.12	[0.88; 11.05]	4.6%	14.9%
Trinchant 2022	12	72	3	10	0.47	[0.11; 2.07]	7.7%	12.3%
Trinchant 2025	33	81	14	23	0.44	[0.17; 1.14]	22.5%	19.5%
**Common effect model**		**308**		**364**	**1.47**	**[1.03; 2.10]**	**87.1%**	.
**Random effects model**					**1.11**	**[0.24; 5.15]**	.	**75.6%**
Heterogeneity: I^2 = 73.6% [25.9%; 90.6%]								
**Common effect model**		**353**		**399**	**1.44**	**[1.03; 2.01]**	**100.0%**	.
**Random effects model**					**1.15**	**[0.51; 2.63]**	.	**100.0%**

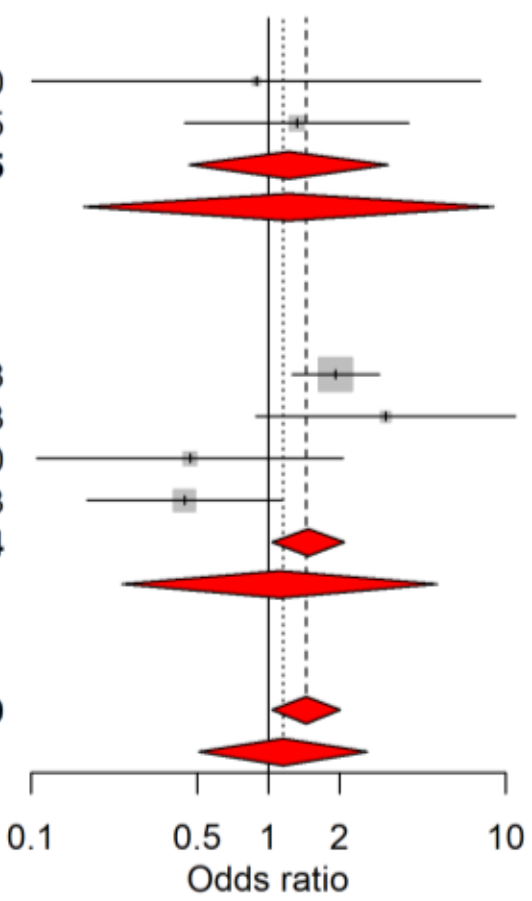

Heterogeneity: I^2 = 56.9% [0.0%; 82.6%]
Test for subgroup differences (common effect): χ^2_1 = 0.13, df = 1 (p = 0.7183)
Test for subgroup differences (random effects): χ^2_1 = 0.03, df = 1 (p = 0.8629)

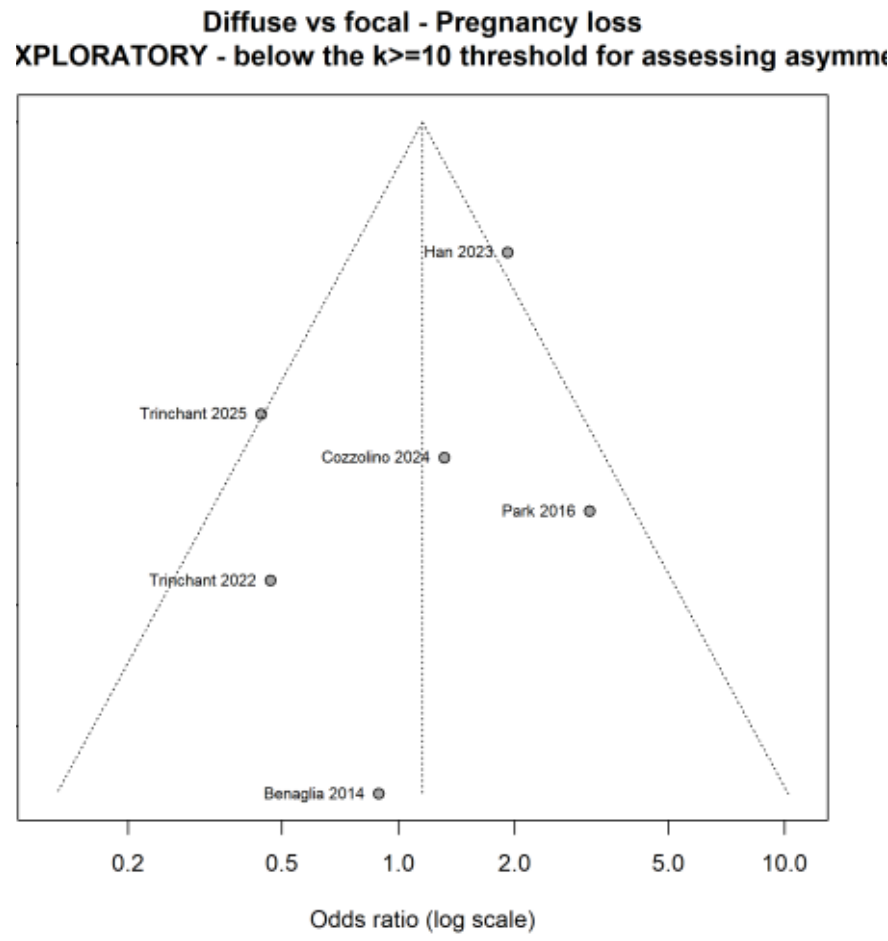

Fig. G: Diffuse AM vs focal AM

Leave one out

Clinical pregnancy

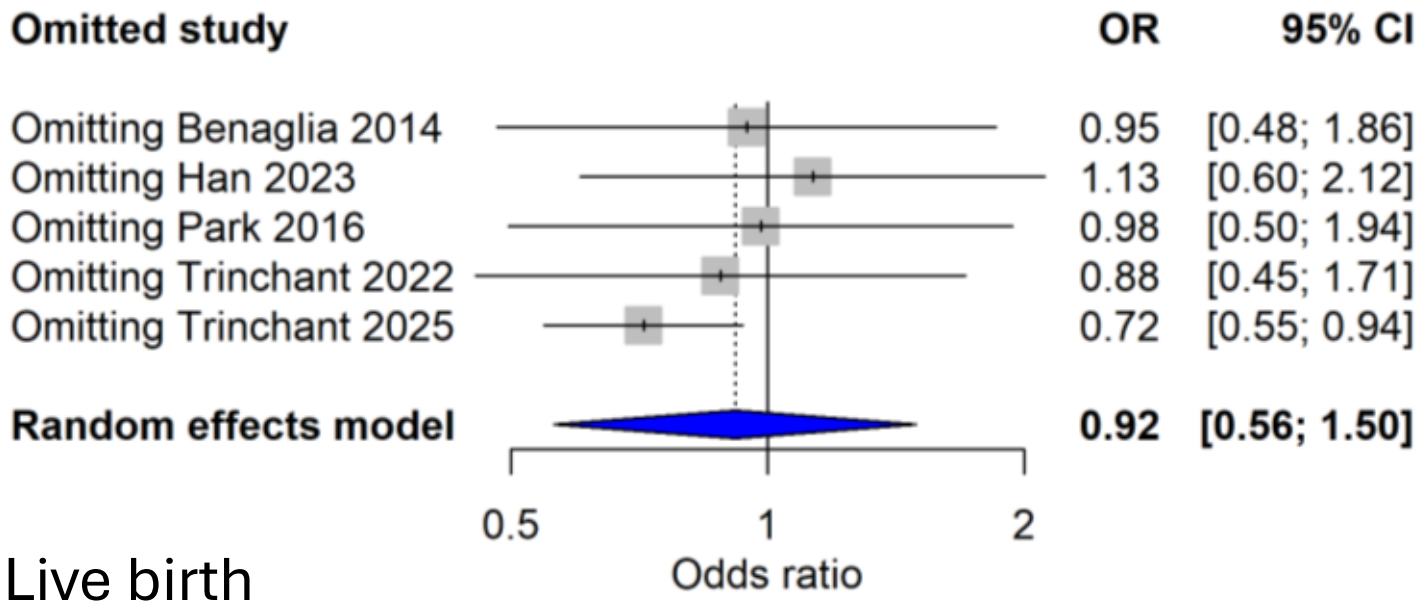

Live birth

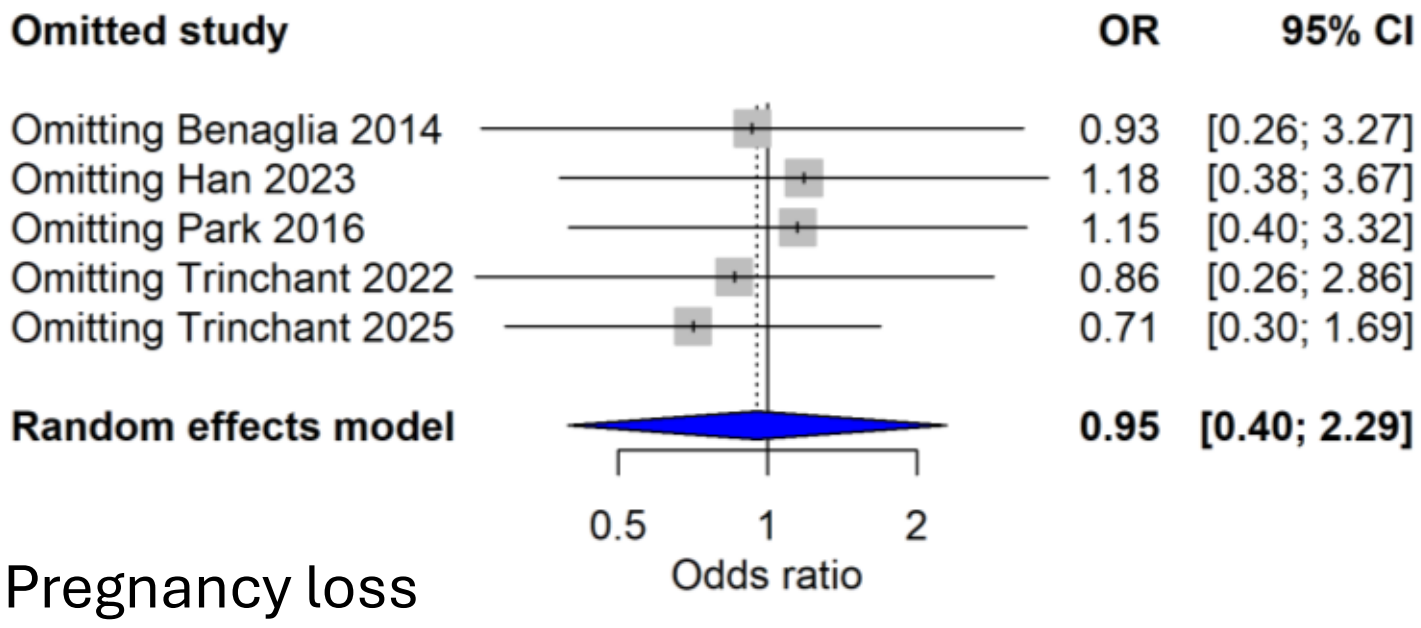

Pregnancy loss

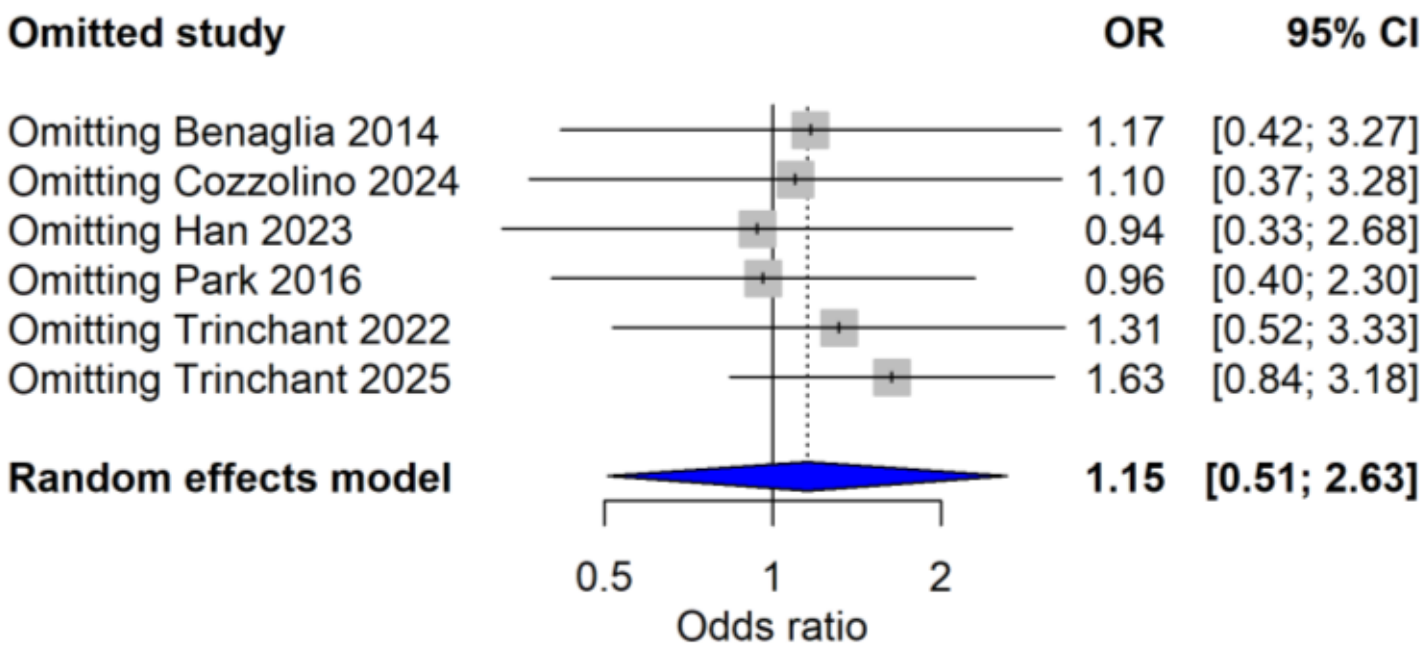

Fig. H: High vs low uterus volume, by design

Clinical pregnancy

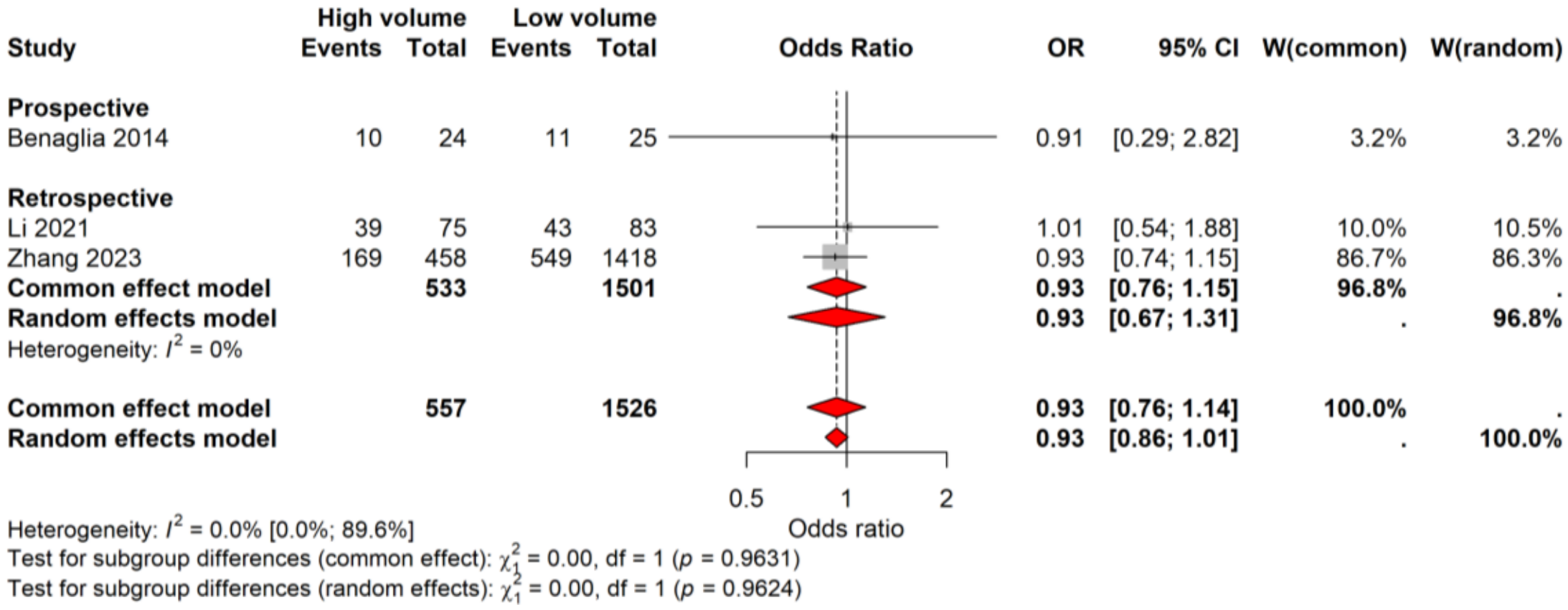

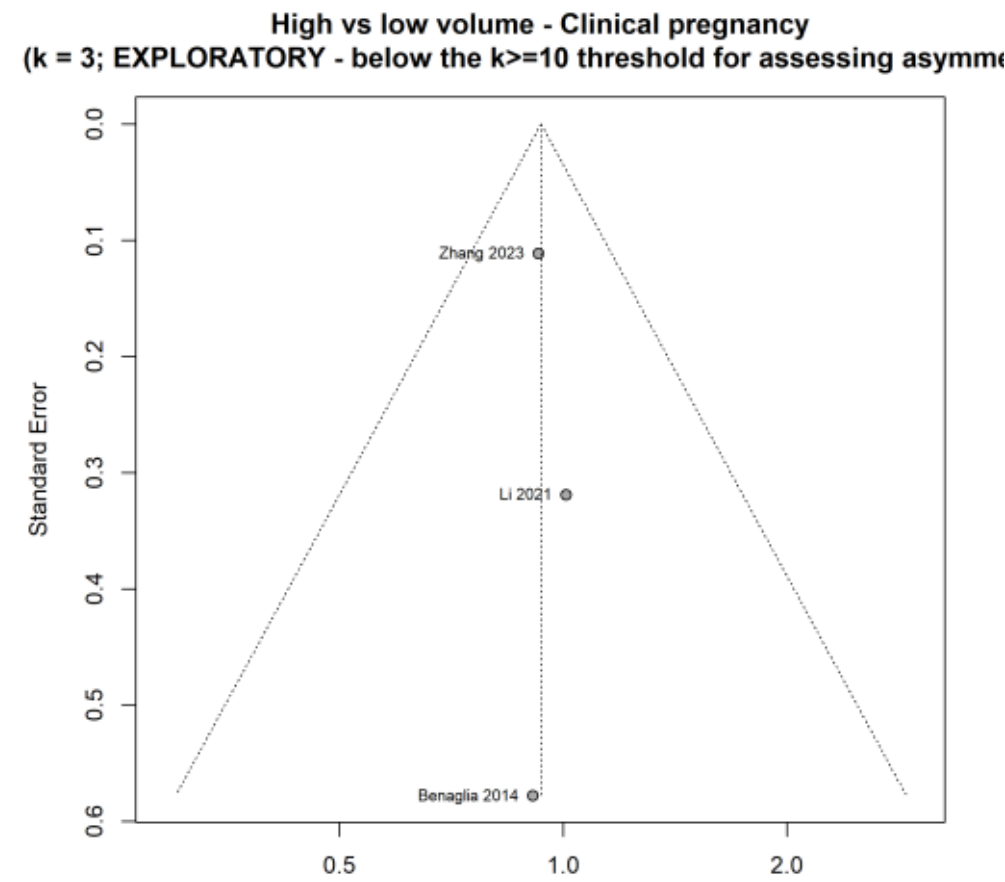

Live birth

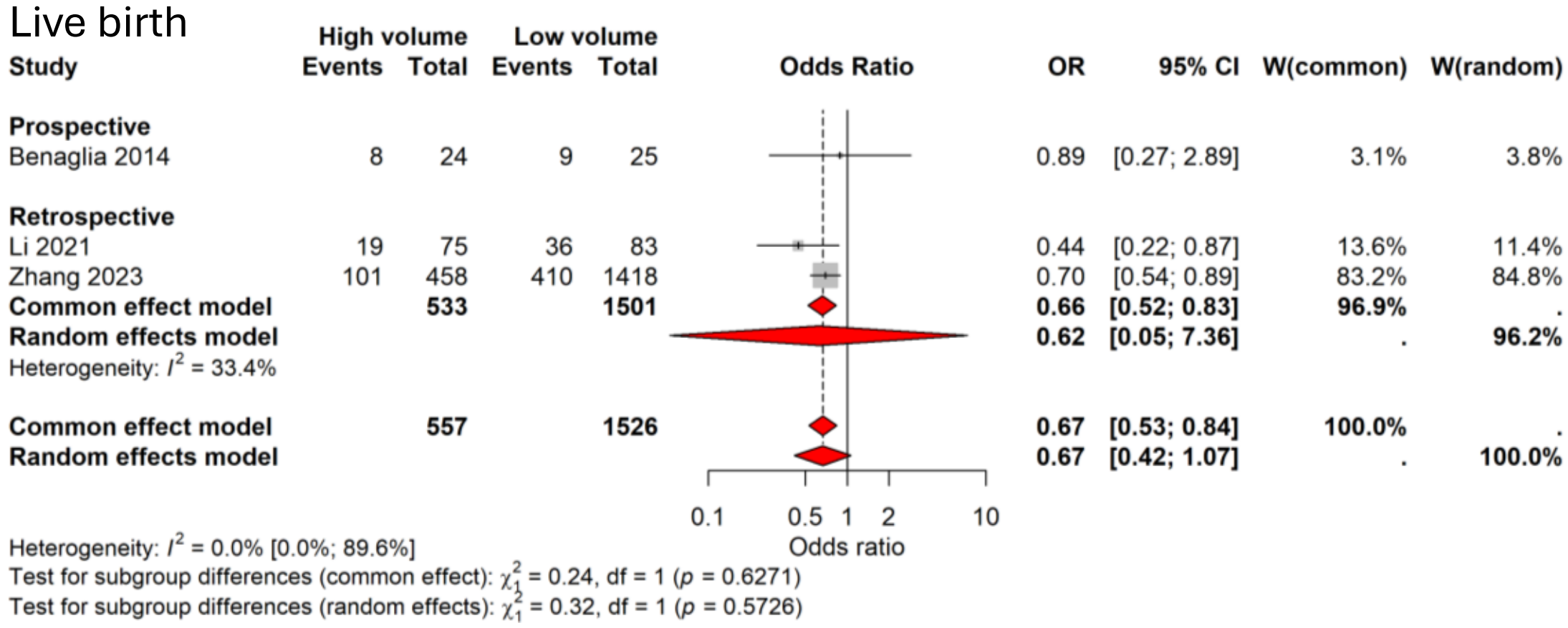

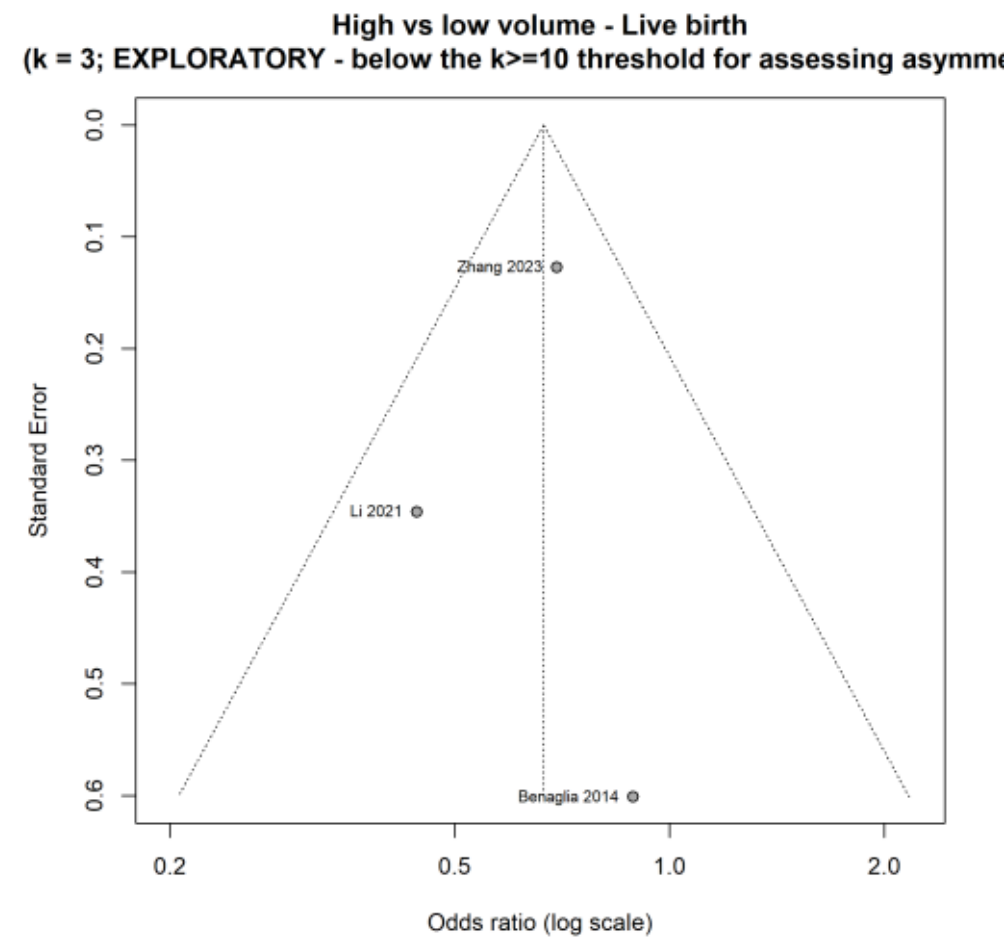

Pregnancy loss

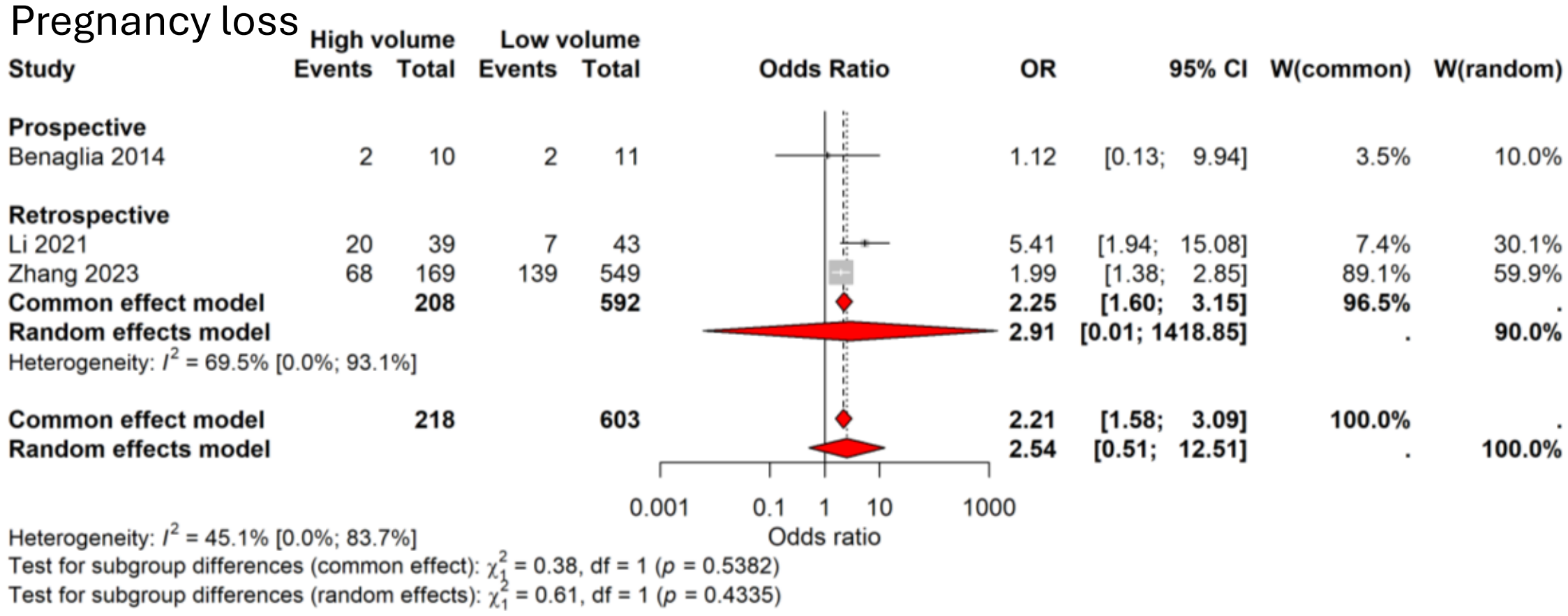

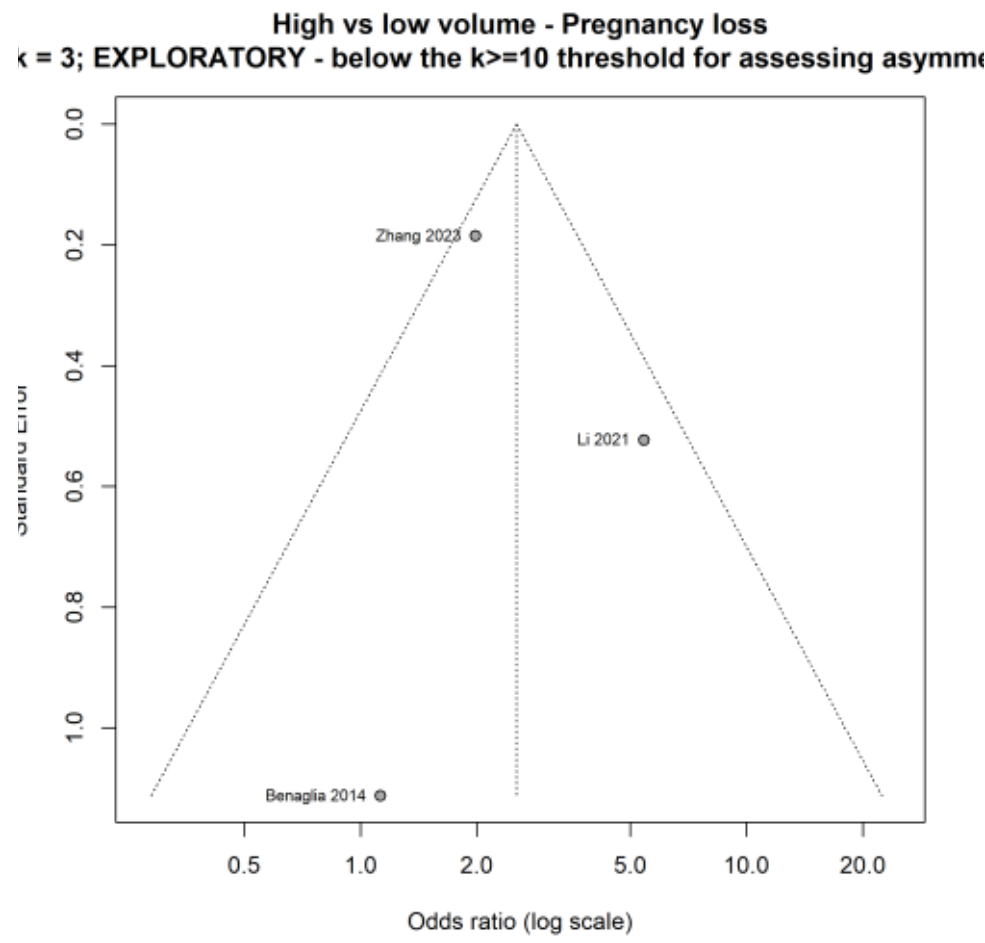

Fig. I: High vs low uterus volume

Leave one out

Clinical pregnancy

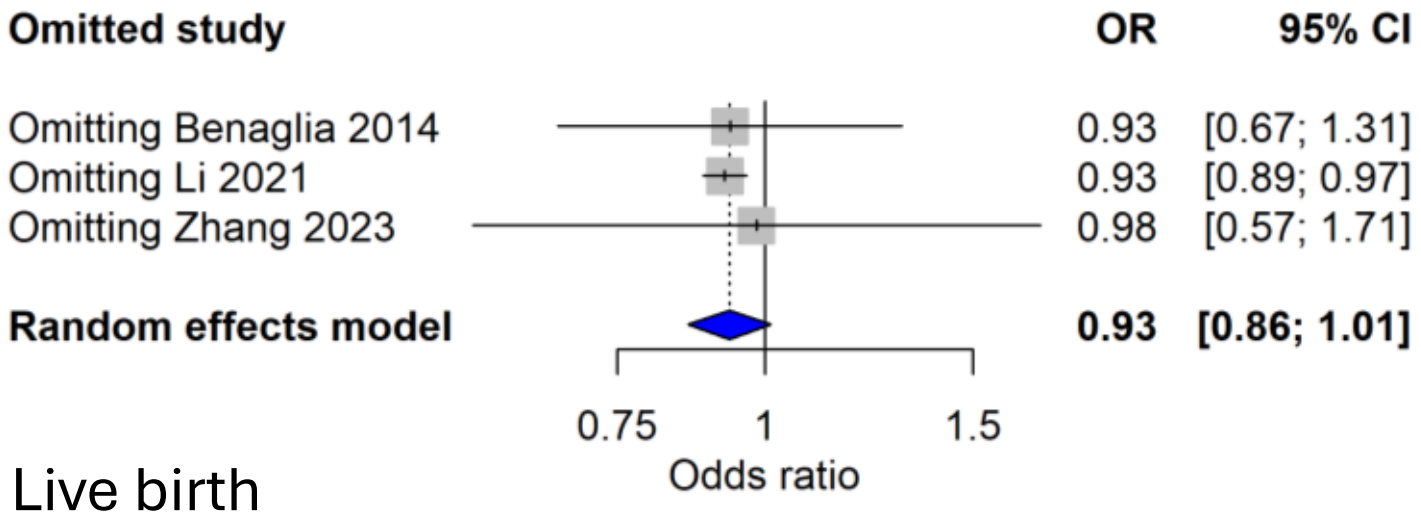

Live birth

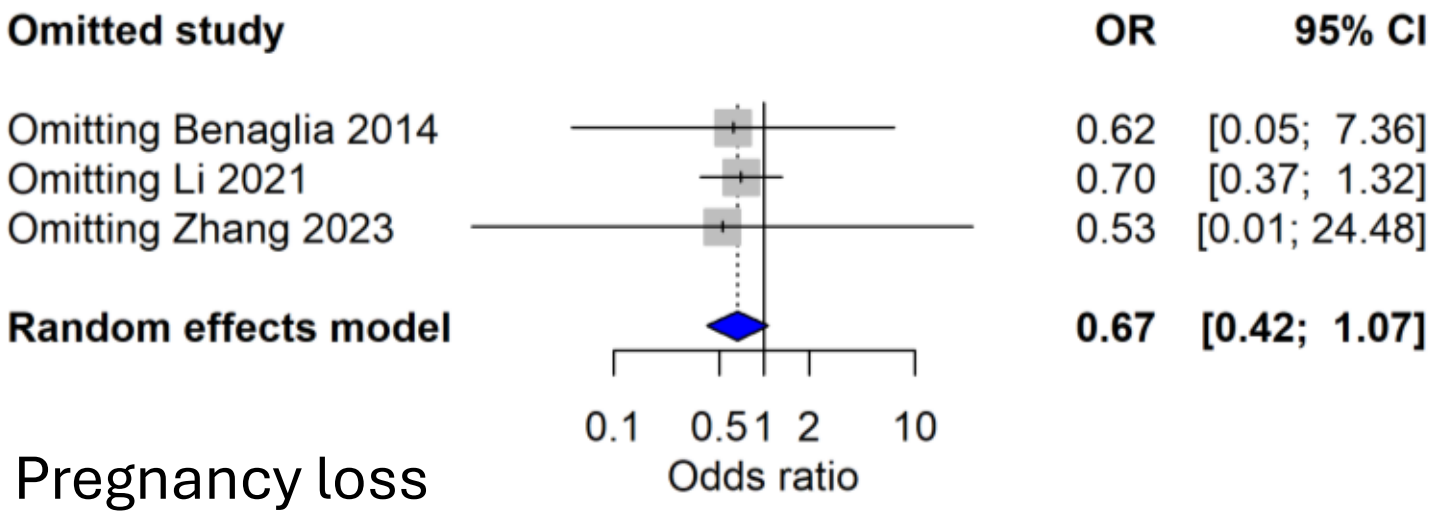

Pregnancy loss

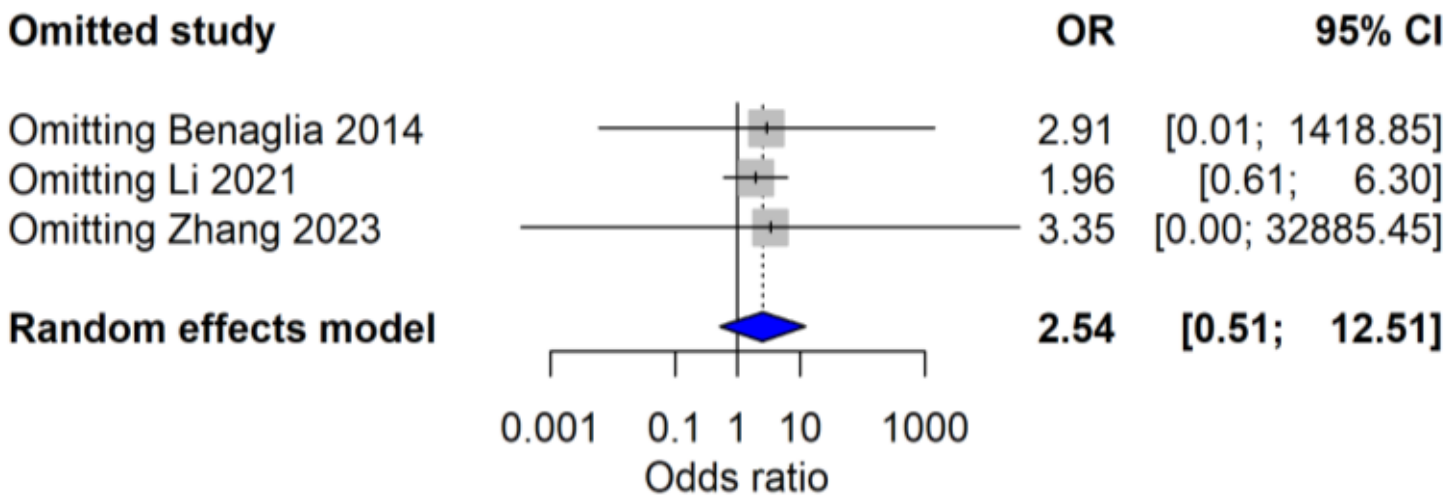

Fig. J: Diffuse AM vs no AM, by design

Clinical pregnancy

Study	Diffuse AM Events	Diffuse AM Total	No AM Events	No AM Total	OR	95% CI	W(common)	W(random)
**Prospective**								
Benaglia 2014	10	25	14	49	1.67	[0.61; 4.59]	1.5%	5.4%
Sharma 2025	118	368	76	217	0.88	[0.61; 1.25]	17.4%	22.5%
**Common effect model**		**393**		**266**	**0.94**	**[0.67; 1.31]**	**18.9%**	.
**Random effects model**					**1.01**	**[0.03; 29.41]**	.	**27.9%**
Heterogeneity: I^2 = 27.7%								
**Retrospective**								
Han 2023	139	428	246	519	0.53	[0.41; 0.70]	40.1%	27.9%
Trinchant 2022	72	155	1869	3128	0.58	[0.42; 0.81]	25.3%	24.3%
Trinchant 2025	81	166	136	228	0.64	[0.43; 0.96]	15.7%	19.9%
**Common effect model**		**749**		**3875**	**0.57**	**[0.48; 0.69]**	**81.1%**	.
**Random effects model**					**0.57**	**[0.46; 0.71]**	.	**72.1%**
Heterogeneity: I^2 = 0% [0.0%; 89.6%]								
**Common effect model**		**1142**		**4141**	**0.64**	**[0.55; 0.75]**	**100.0%**	.
**Random effects model**					**0.67**	**[0.45; 1.00]**	.	**100.0%**

Odds ratio (axis: 0.1, 0.5, 1, 2, 10)

Heterogeneity: I^2 = 53.1% [0.0%; 82.8%]
Test for subgroup differences (common effect): χ^2_1 = 6.54, df = 1 (p = 0.0105)
Test for subgroup differences (random effects): χ^2_1 = 4.40, df = 1 (p = 0.0360)

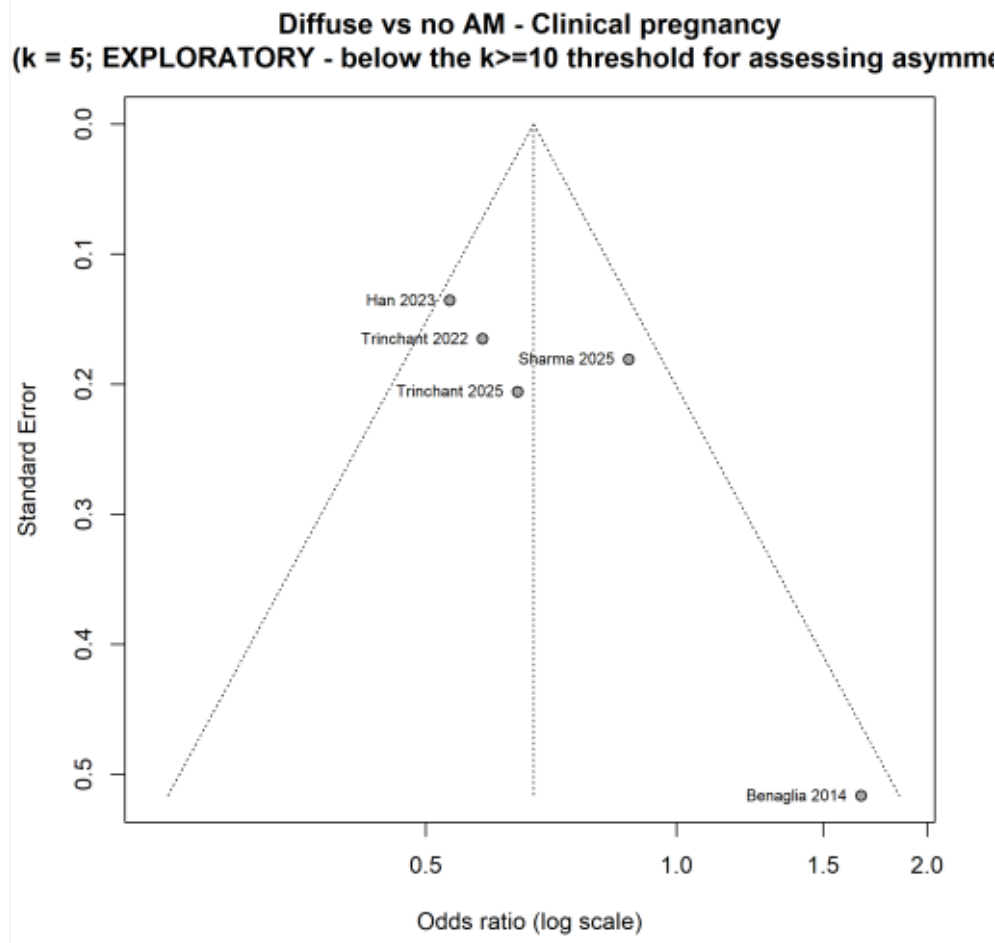

Live birth

Study	Diffuse AM Events	Diffuse AM Total	No AM Events	No AM Total	OR	95% CI	W(common)	W(random)
**Prospective**								
Benaglia 2014	9	25	18	49	0.97	[0.36; 2.64]	2.0%	7.3%
Sharma 2025	76	368	65	217	0.61	[0.41; 0.89]	17.1%	22.1%
**Common effect model**		**393**		**266**	**0.65**	**[0.45; 0.93]**	**19.1%**	.
**Random effects model**					**0.65**	**[0.09; 4.66]**	.	**29.5%**
Heterogeneity: I^2 = 0%								
**Retrospective**								
Han 2023	80	428	208	519	0.34	[0.25; 0.46]	40.2%	25.7%
Trinchant 2022	60	155	1560	3128	0.63	[0.46; 0.88]	23.7%	24.4%
Trinchant 2025	47	166	107	228	0.45	[0.29; 0.68]	17.0%	20.5%
**Common effect model**		**749**		**3875**	**0.45**	**[0.37; 0.55]**	**80.9%**	.
**Random effects model**					**0.46**	**[0.21; 1.01]**	.	**70.5%**
Heterogeneity: I^2 = 72.5% [7.1%; 91.8%]								
**Common effect model**		**1142**		**4141**	**0.49**	**[0.41; 0.58]**	**100.0%**	.
**Random effects model**					**0.52**	**[0.34; 0.79]**	.	**100.0%**

Odds ratio (axis: 0.1, 0.5, 1, 2, 10)

Heterogeneity: I^2 = 63.3% [3.2%; 86.1%]
Test for subgroup differences (common effect): χ^2_1 = 3.00, df = 1 (p = 0.0835)
Test for subgroup differences (random effects): χ^2_1 = 2.04, df = 1 (p = 0.1531)

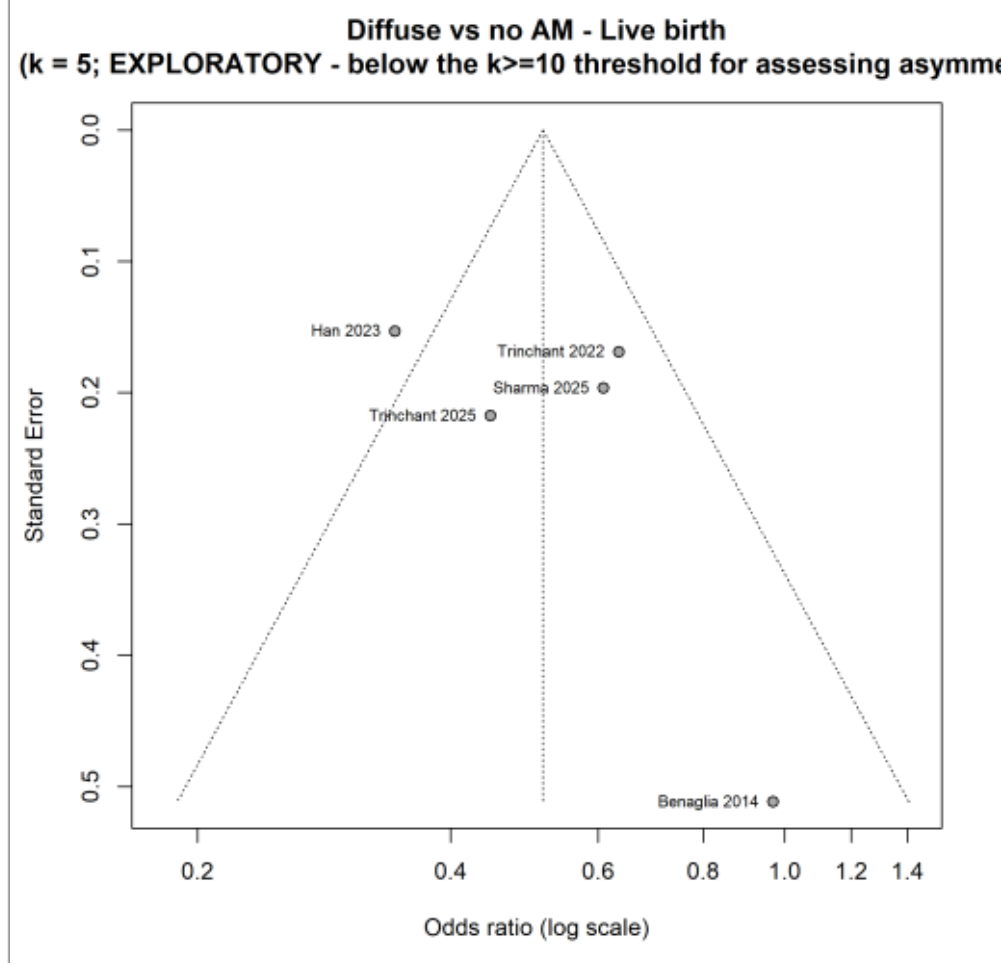

Pregnancy loss

Study	Diffuse AM Events	Diffuse AM Total	No AM Events	No AM Total	OR	95% CI	W(common)	W(random)
**Prospective**								
Benaglia 2014	1	10	5	14	0.20	[0.02; 2.07]	6.3%	5.4%
Cozzolino 2024	13	34	11	56	2.53	[0.97; 6.58]	8.6%	15.8%
Sharma 2025	26	118	7	76	2.79	[1.14; 6.79]	11.2%	16.6%
**Common effect model**		**162**		**146**	**2.08**	**[1.14; 3.78]**	**26.1%**	.
**Random effects model**					**1.80**	**[0.11; 30.29]**	.	**37.7%**
Heterogeneity: I^2 = 54.9% [0.0%; 87.1%]								
**Retrospective**								
Han 2023	58	139	38	246	3.92	[2.42; 6.35]	26.9%	22.2%
Trinchant 2022	12	72	309	1869	1.01	[0.54; 1.90]	32.1%	20.2%
Trinchant 2025	33	81	20	136	3.99	[2.08; 7.63]	14.9%	19.9%
**Common effect model**		**292**		**2251**	**2.67**	**[1.96; 3.63]**	**73.9%**	.
**Random effects model**					**2.53**	**[0.36; 17.73]**	.	**62.3%**
Heterogeneity: I^2 = 84.6% [54.2%; 94.8%]								
**Common effect model**		**454**		**2397**	**2.51**	**[1.91; 3.31]**	**100.0%**	.
**Random effects model**					**2.25**	**[0.93; 5.43]**	.	**100.0%**

Odds ratio (axis: 0.1, 0.5, 1, 2, 10)

Heterogeneity: I^2 = 71.8% [34.8%; 87.8%]
Test for subgroup differences (common effect): χ^2_1 = 0.53, df = 1 (p = 0.4659)
Test for subgroup differences (random effects): χ^2_1 = 0.19, df = 1 (p = 0.6668)

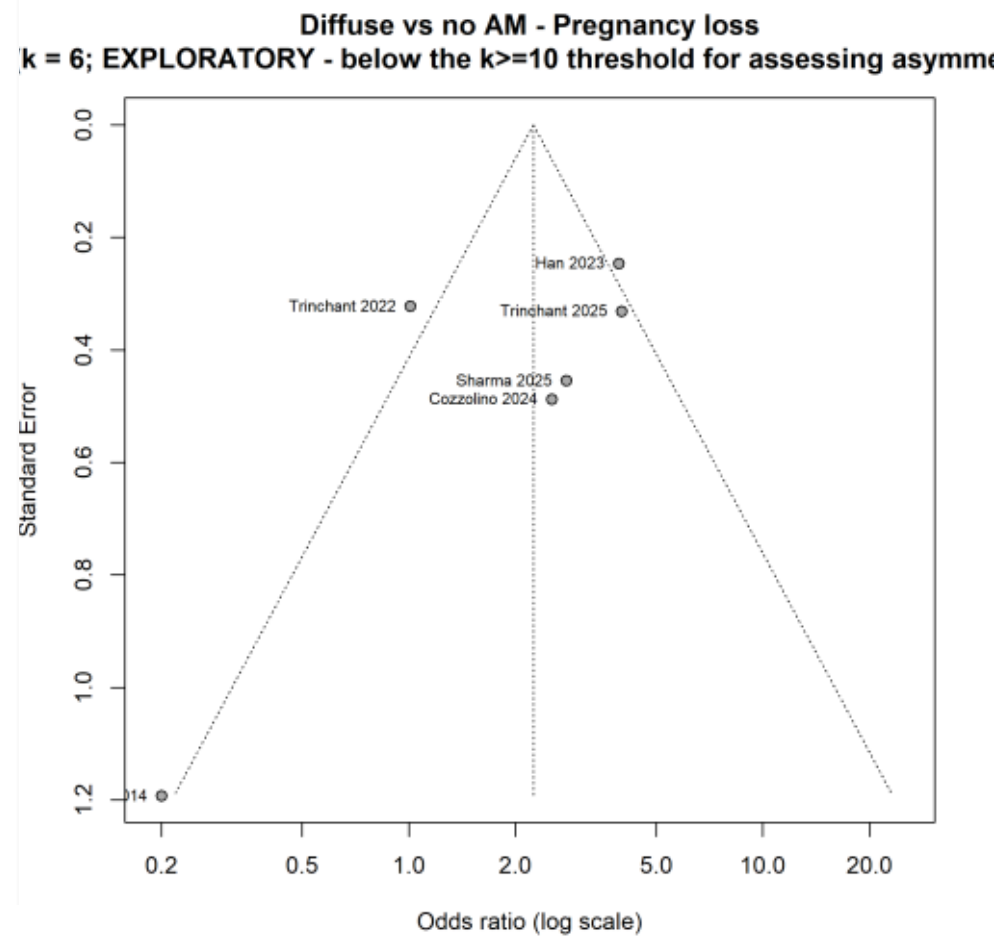

Fig. K: Diffuse AM vs no AM

Leave one out

Clinical pregnancy

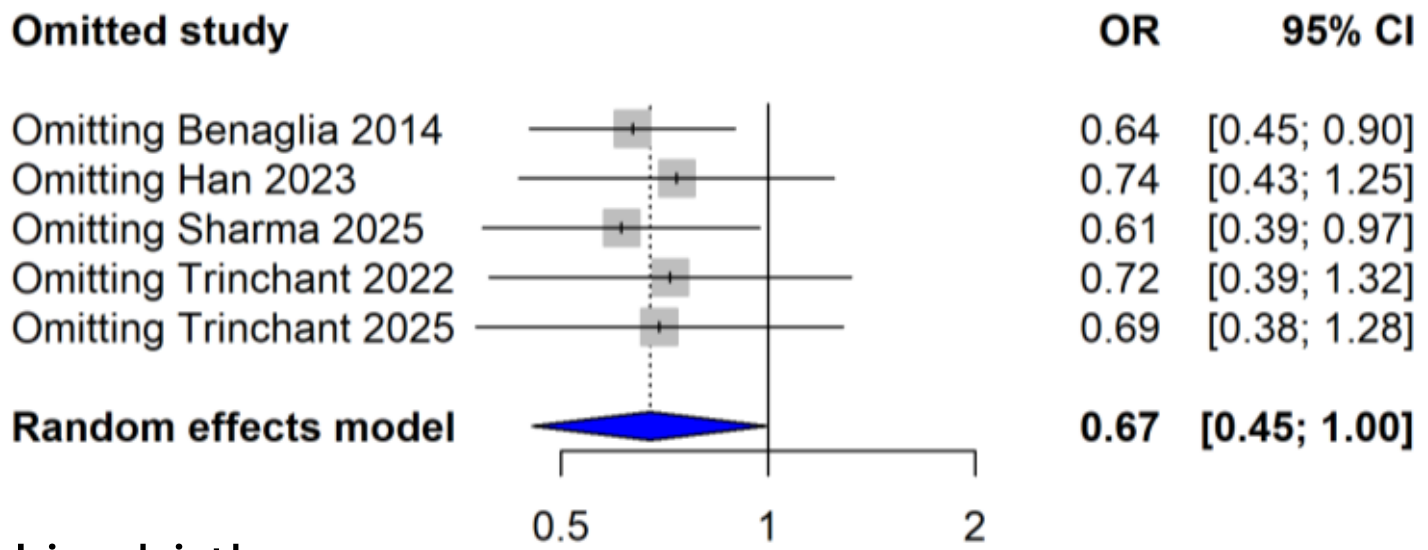

Live birth

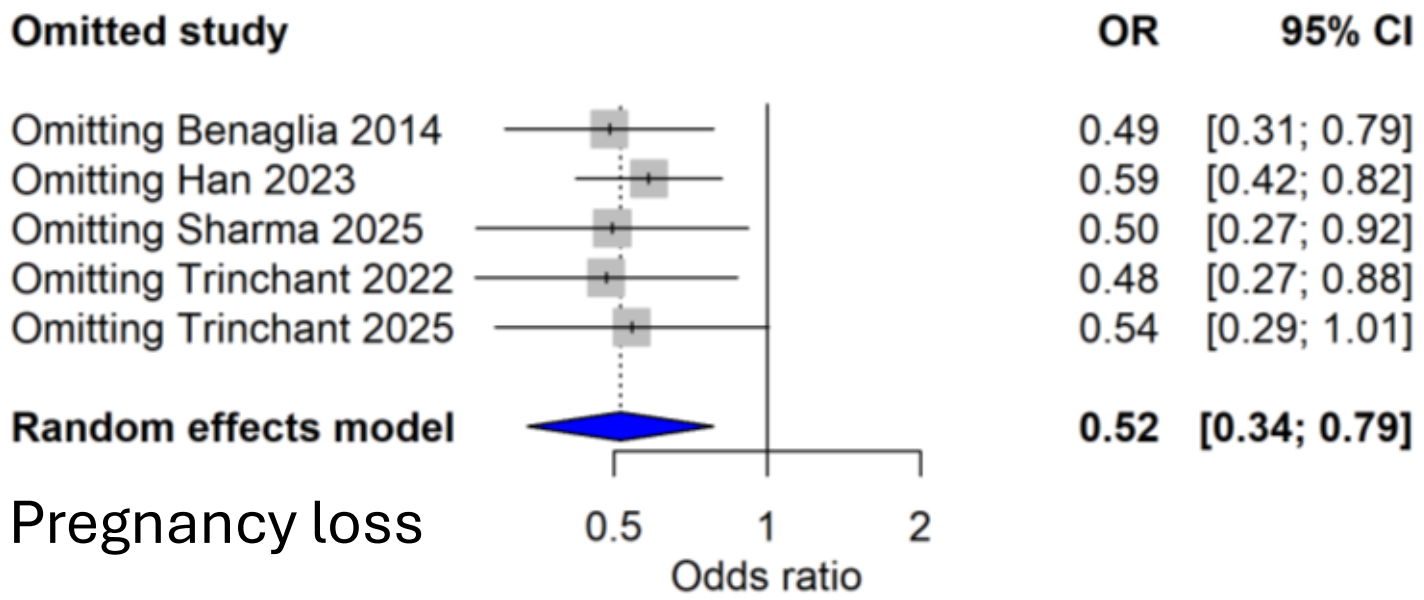

Pregnancy loss

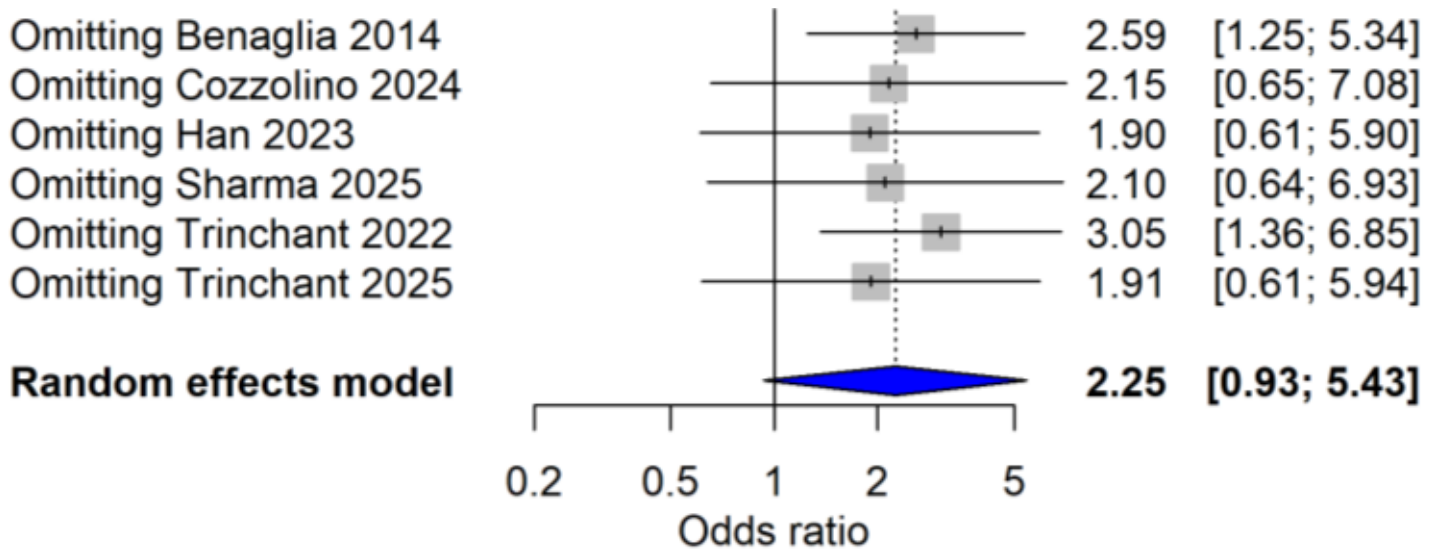

Fig. L: Myometrial cysts

All studies

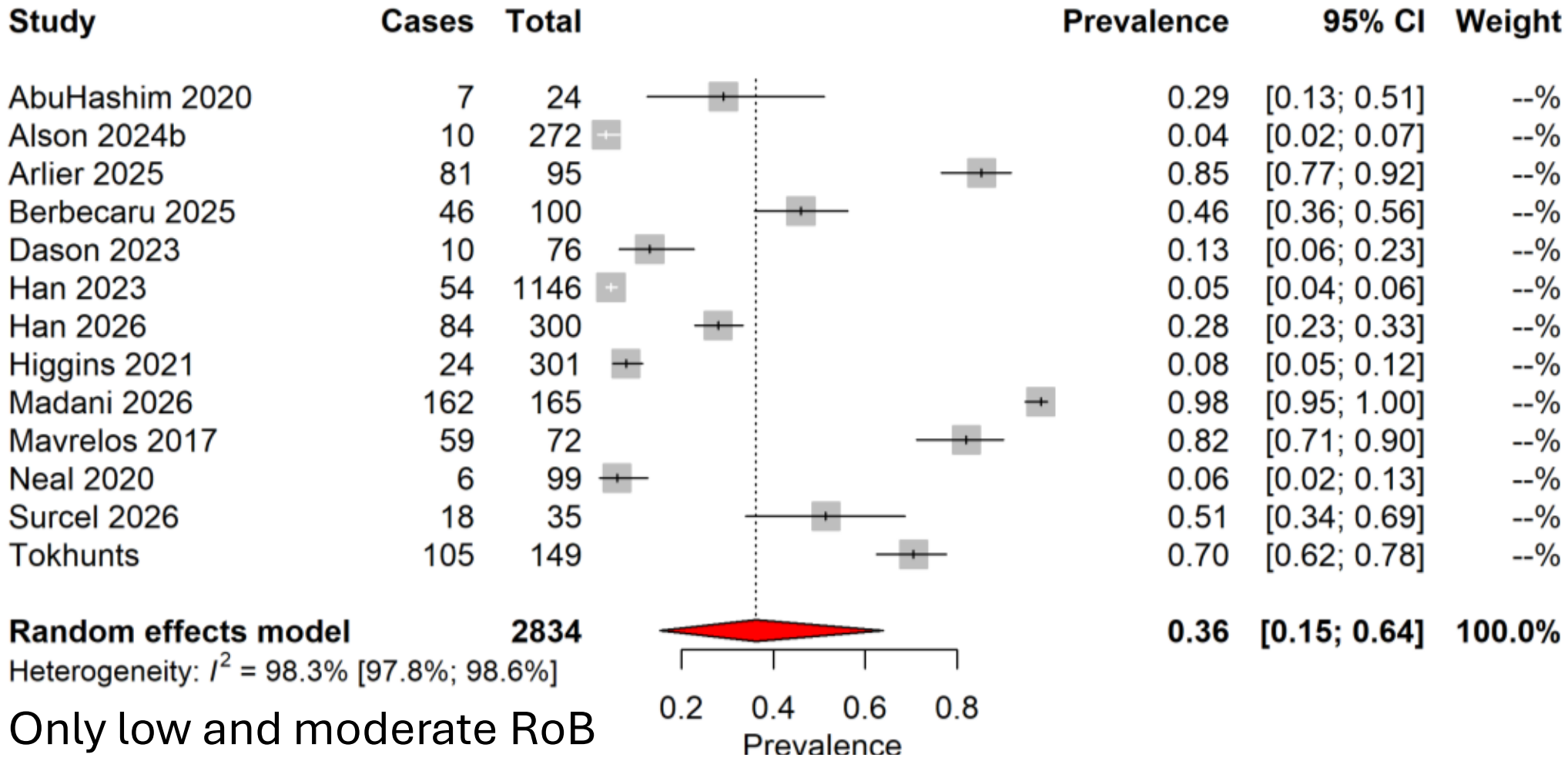

Only low and moderate RoB

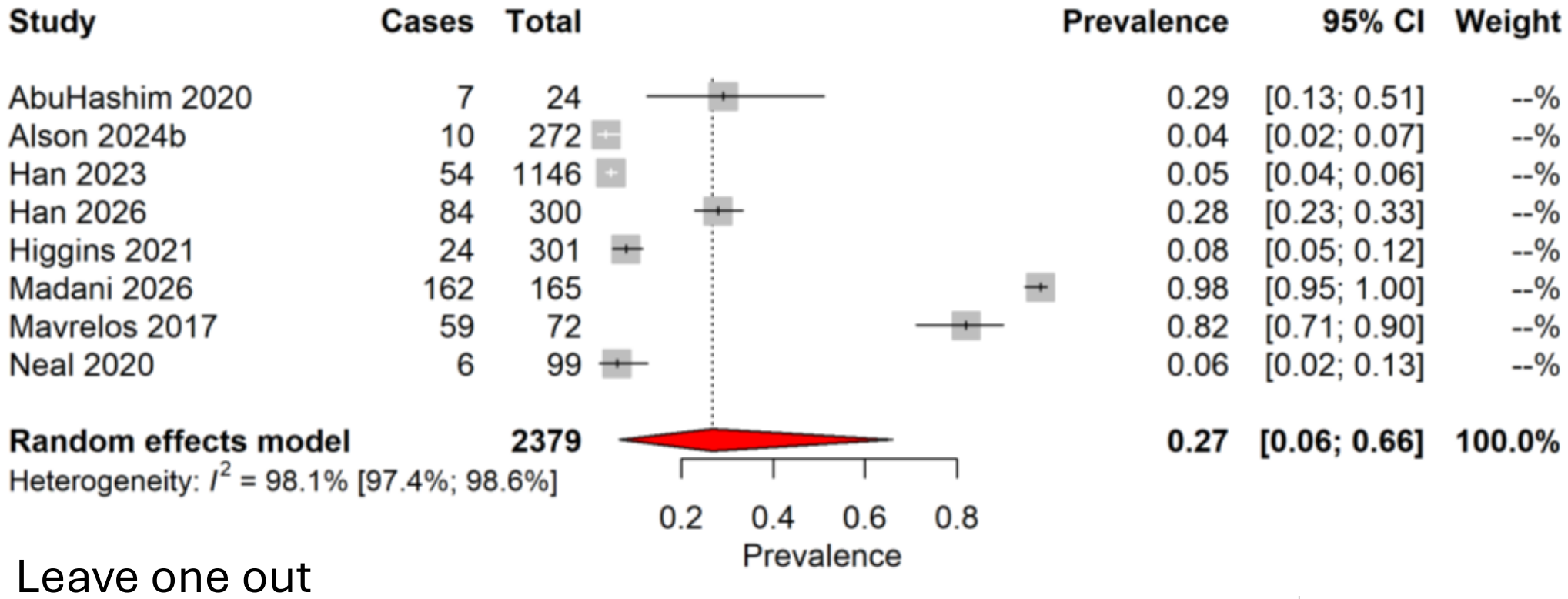

Leave one out

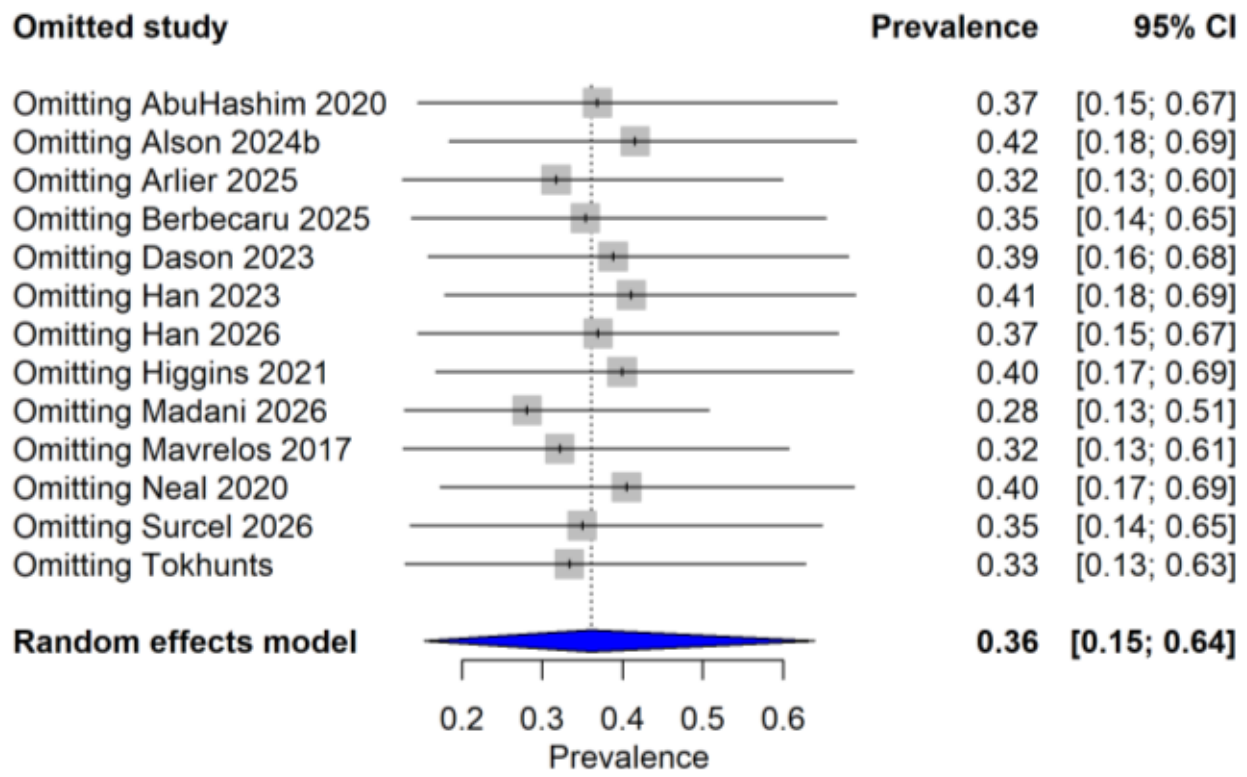

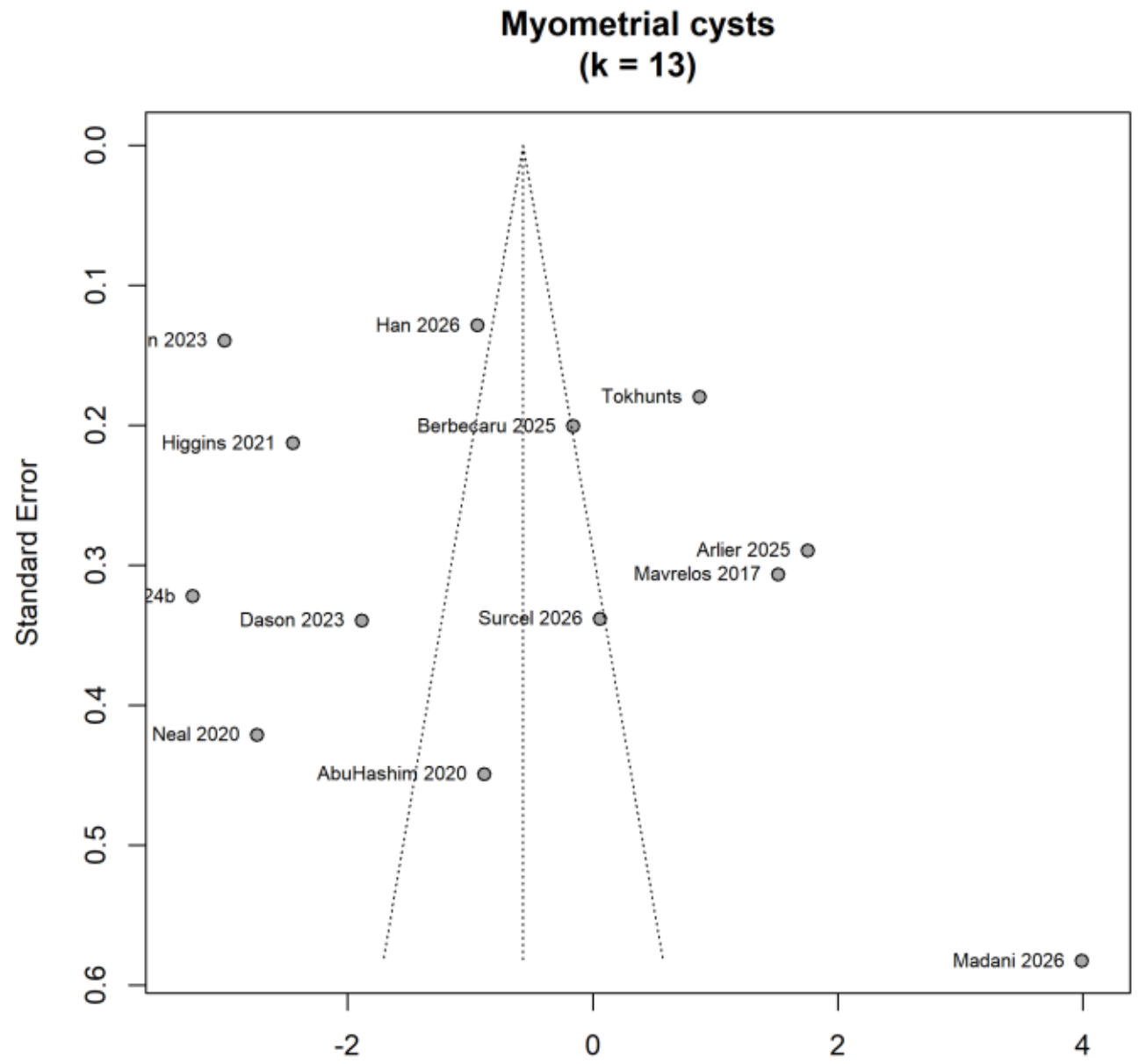

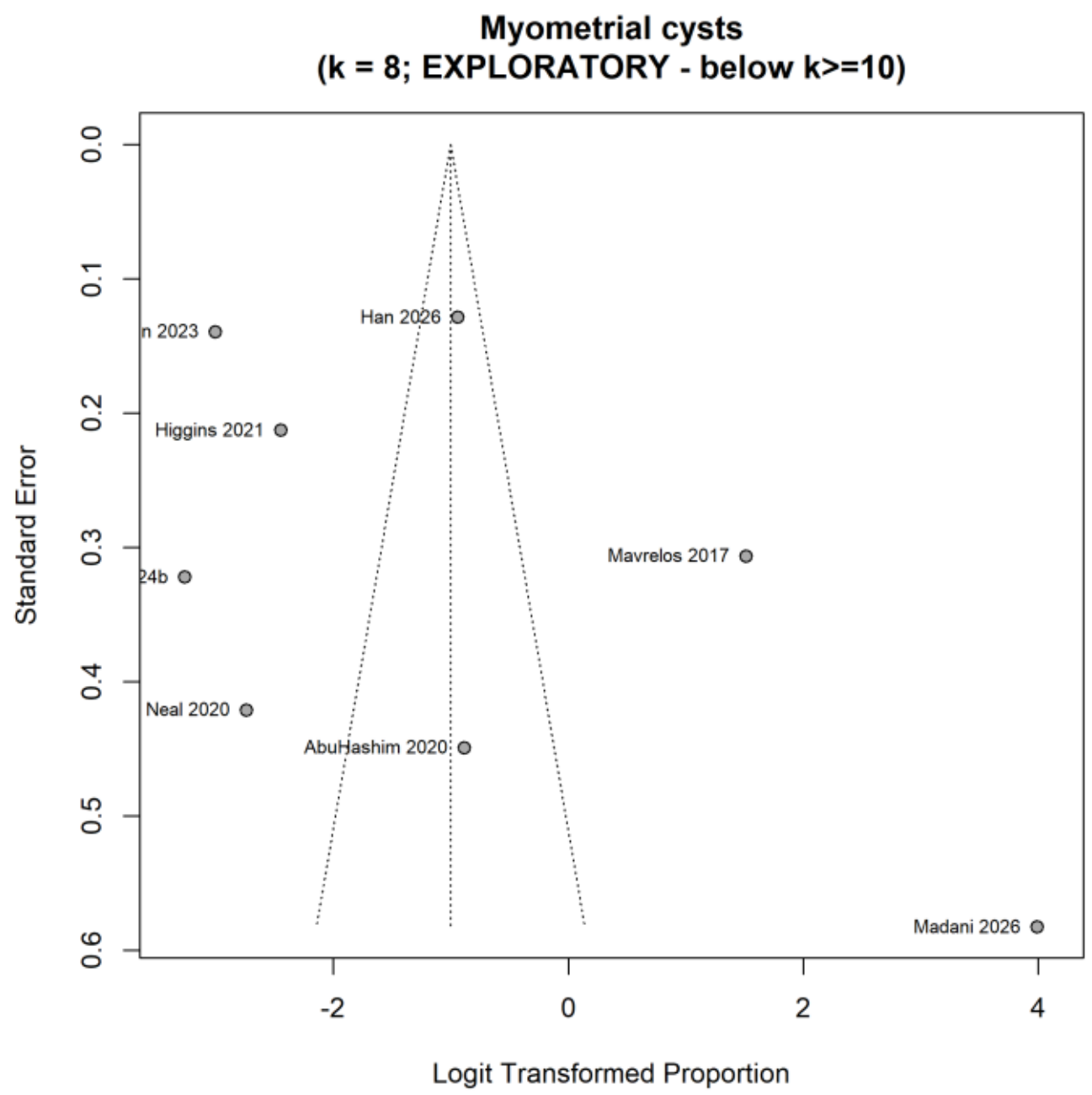

Fig. M: Hyperechogenic islands

All studies

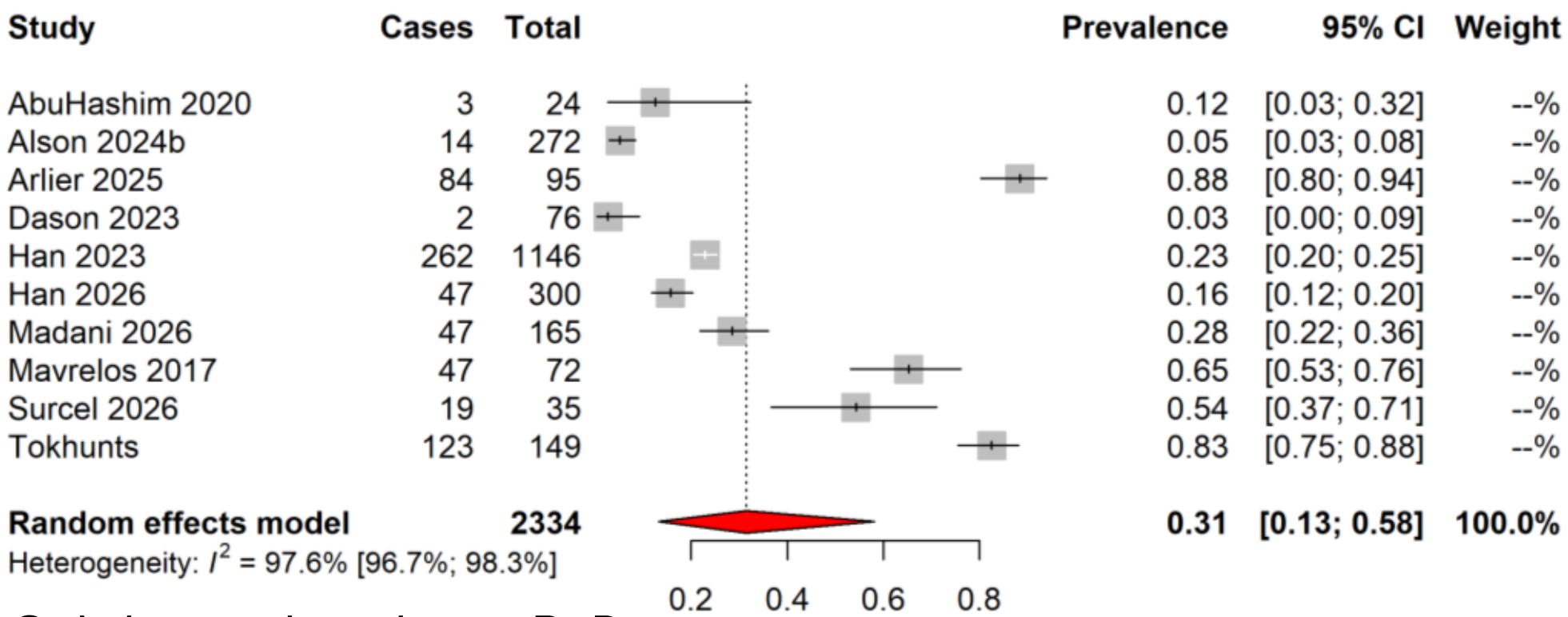

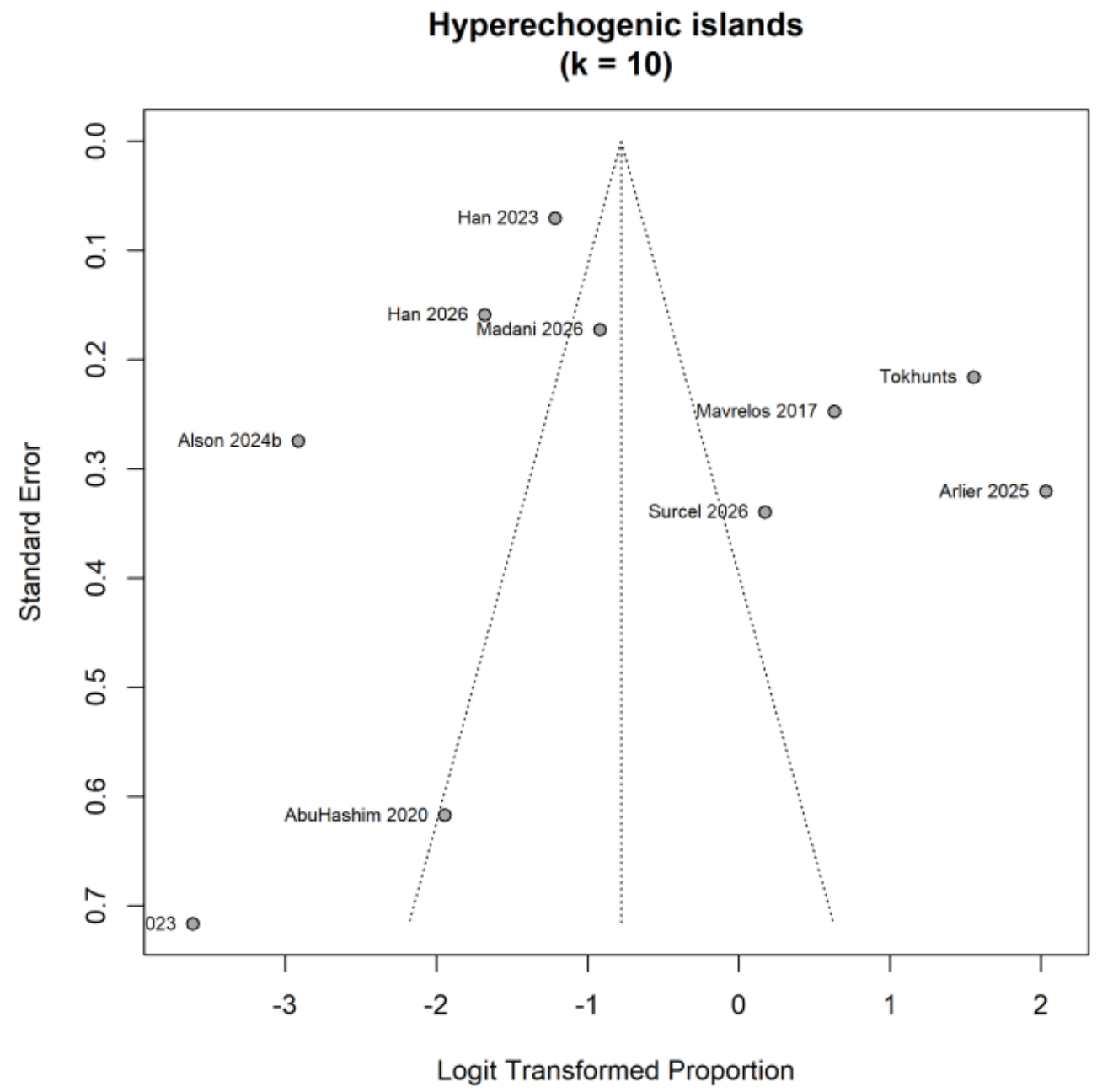

Only low and moderate RoB

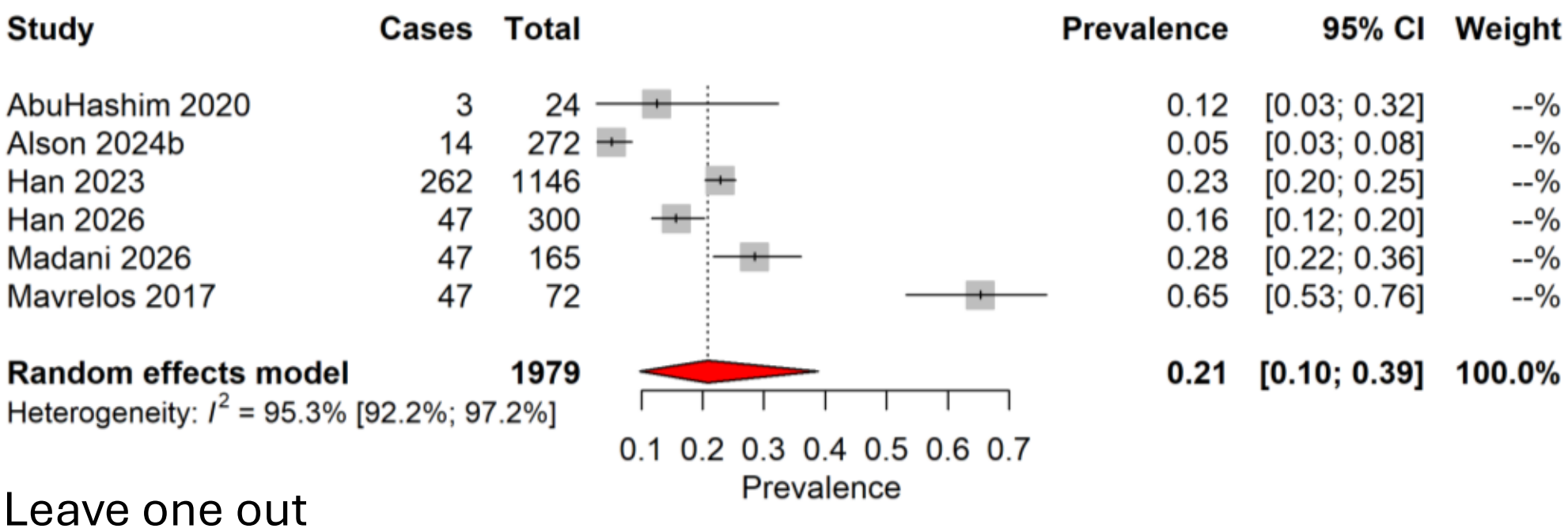

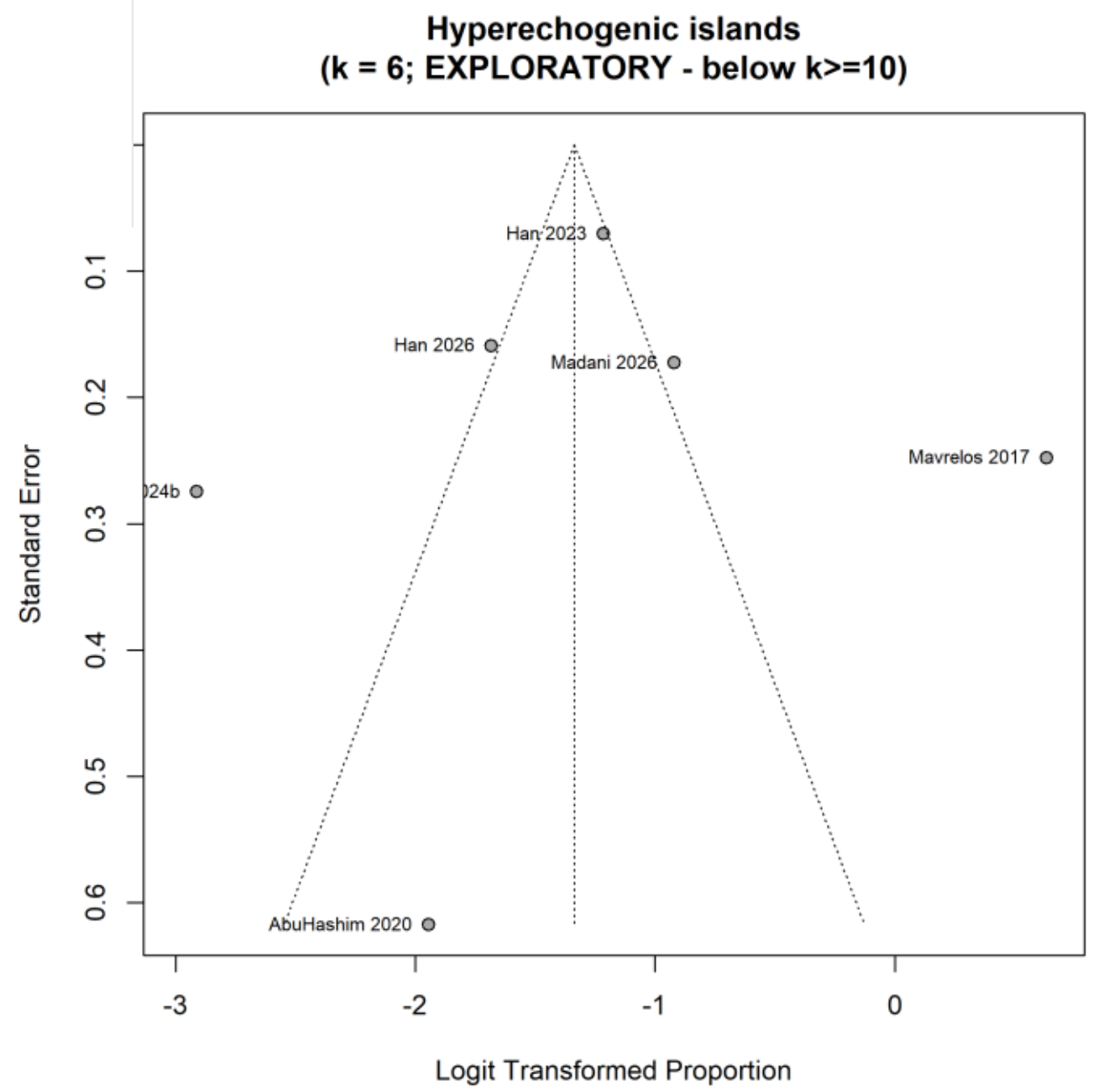

Leave one out

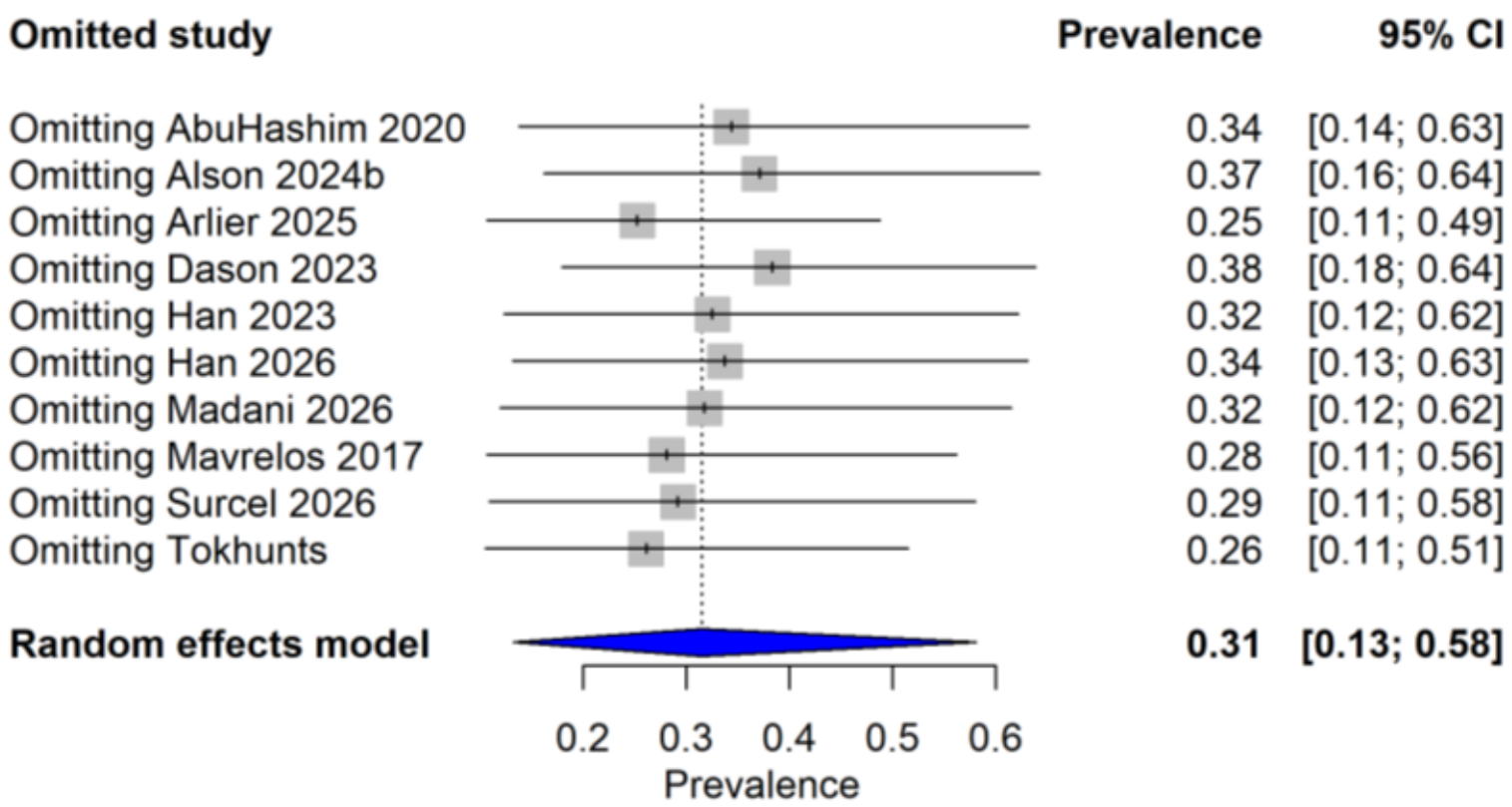

Fig. N: Lines and buds

All studies

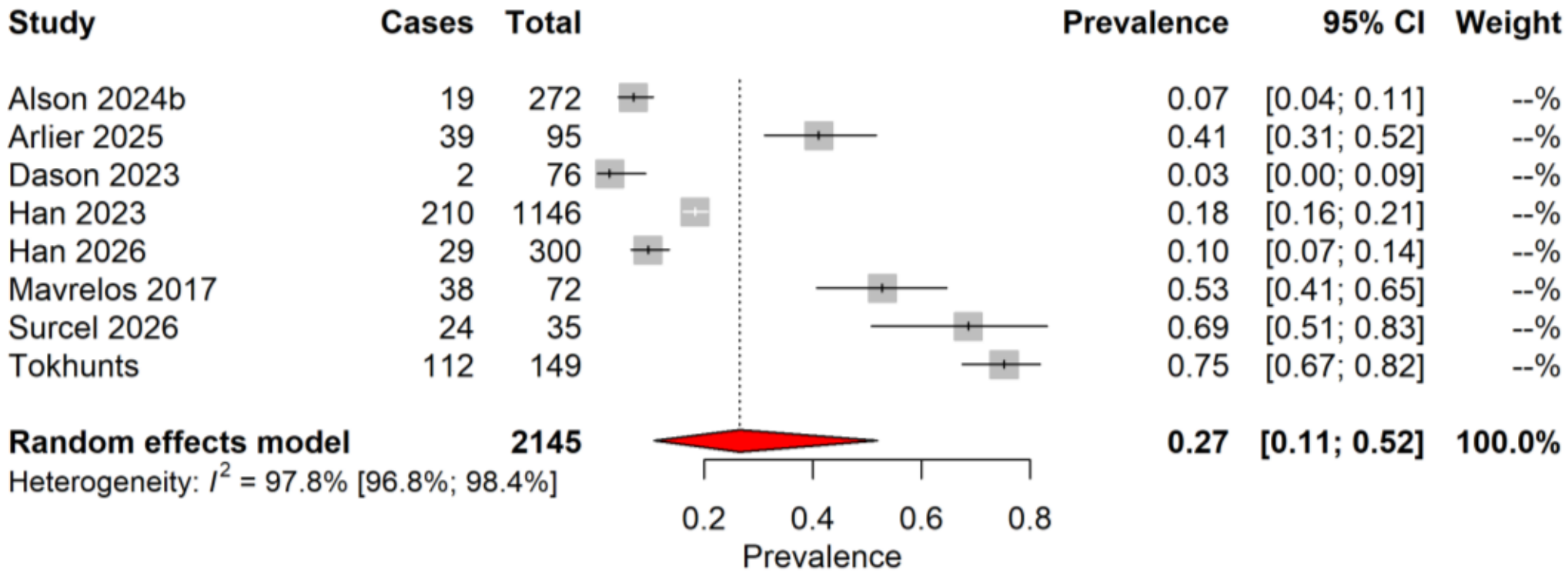

Only low and moderate RoB

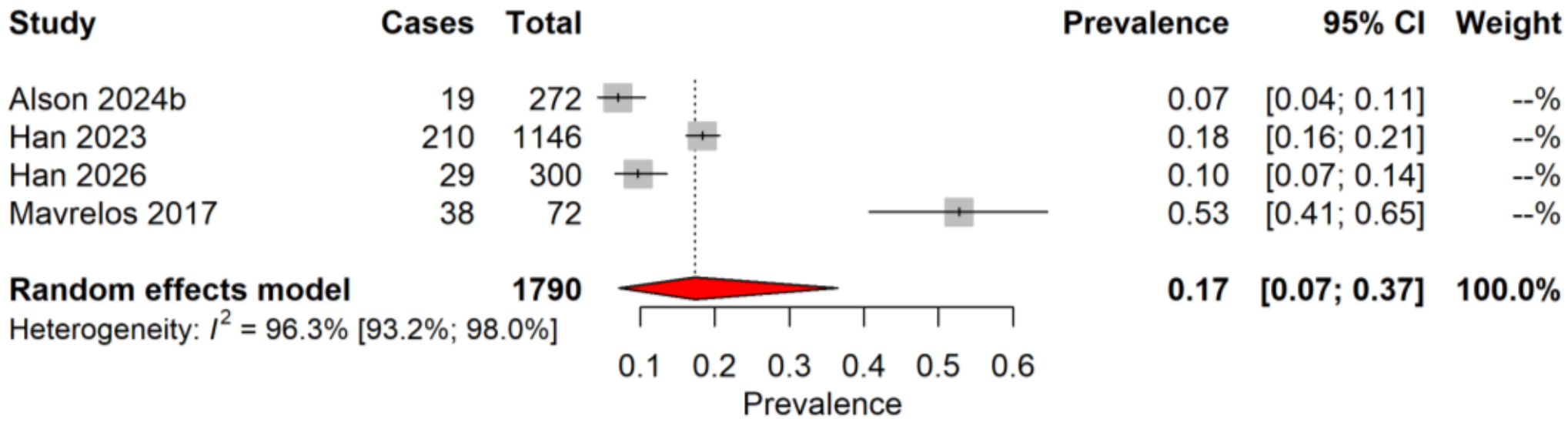

Leave one out

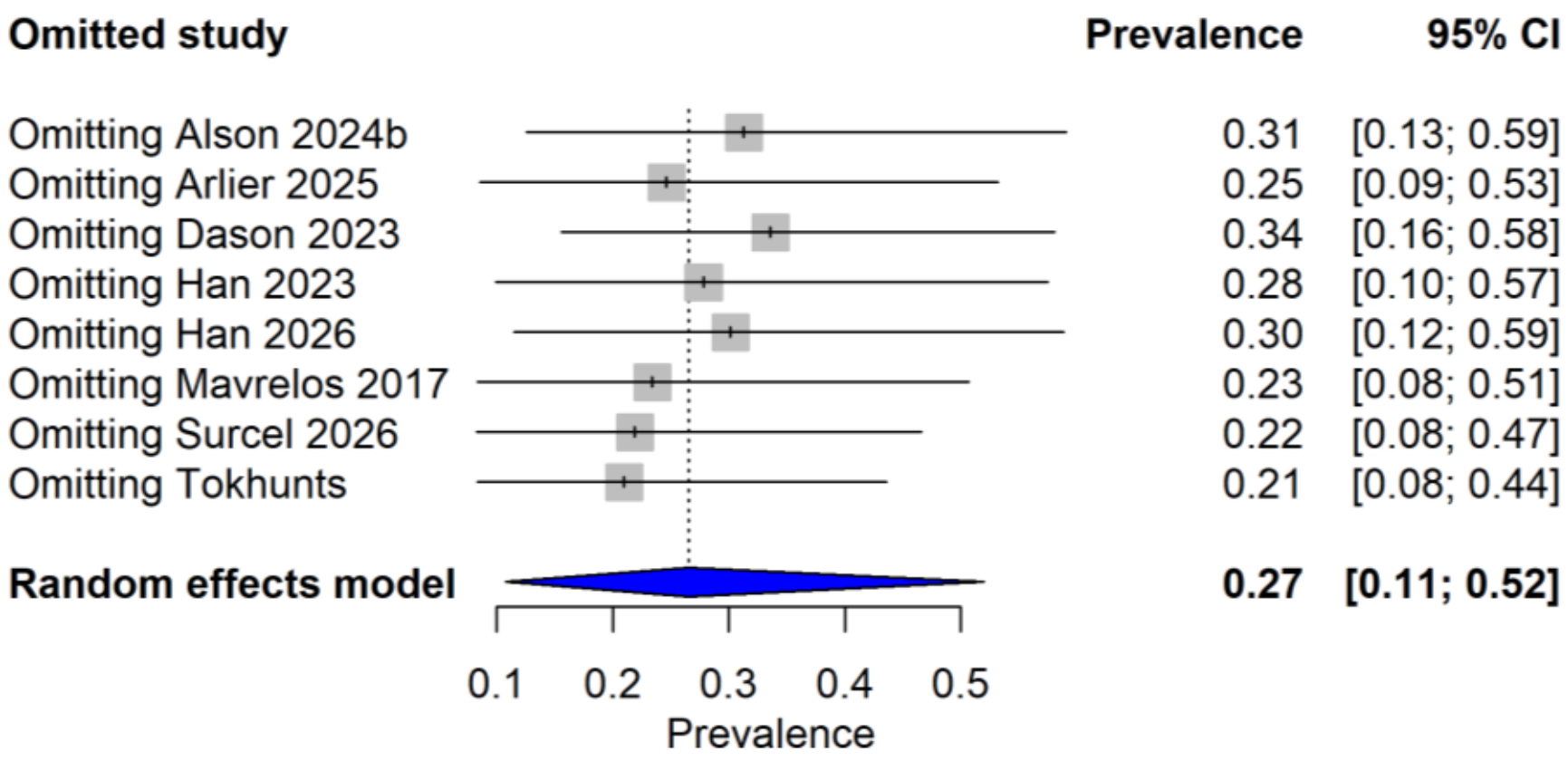

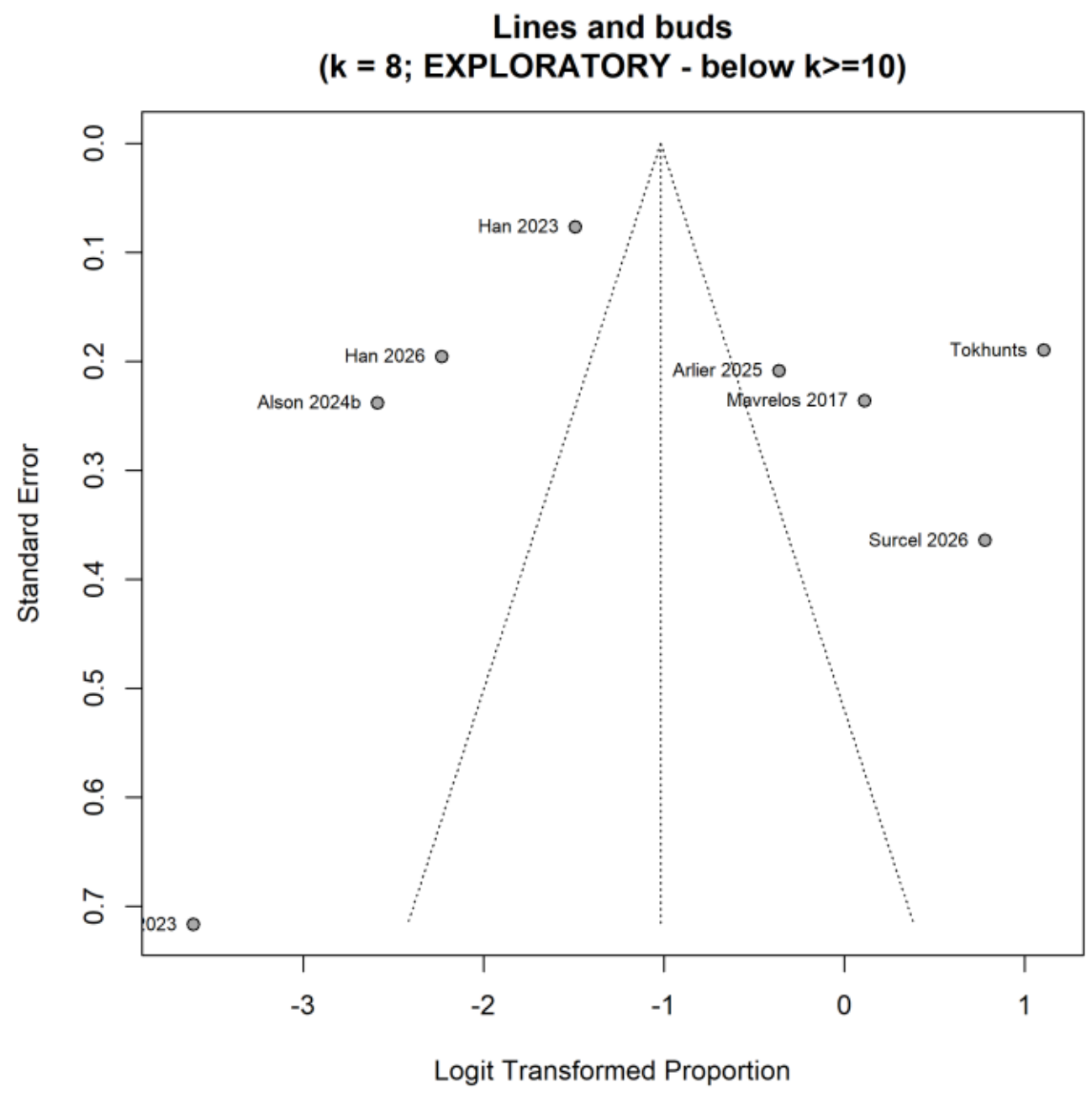

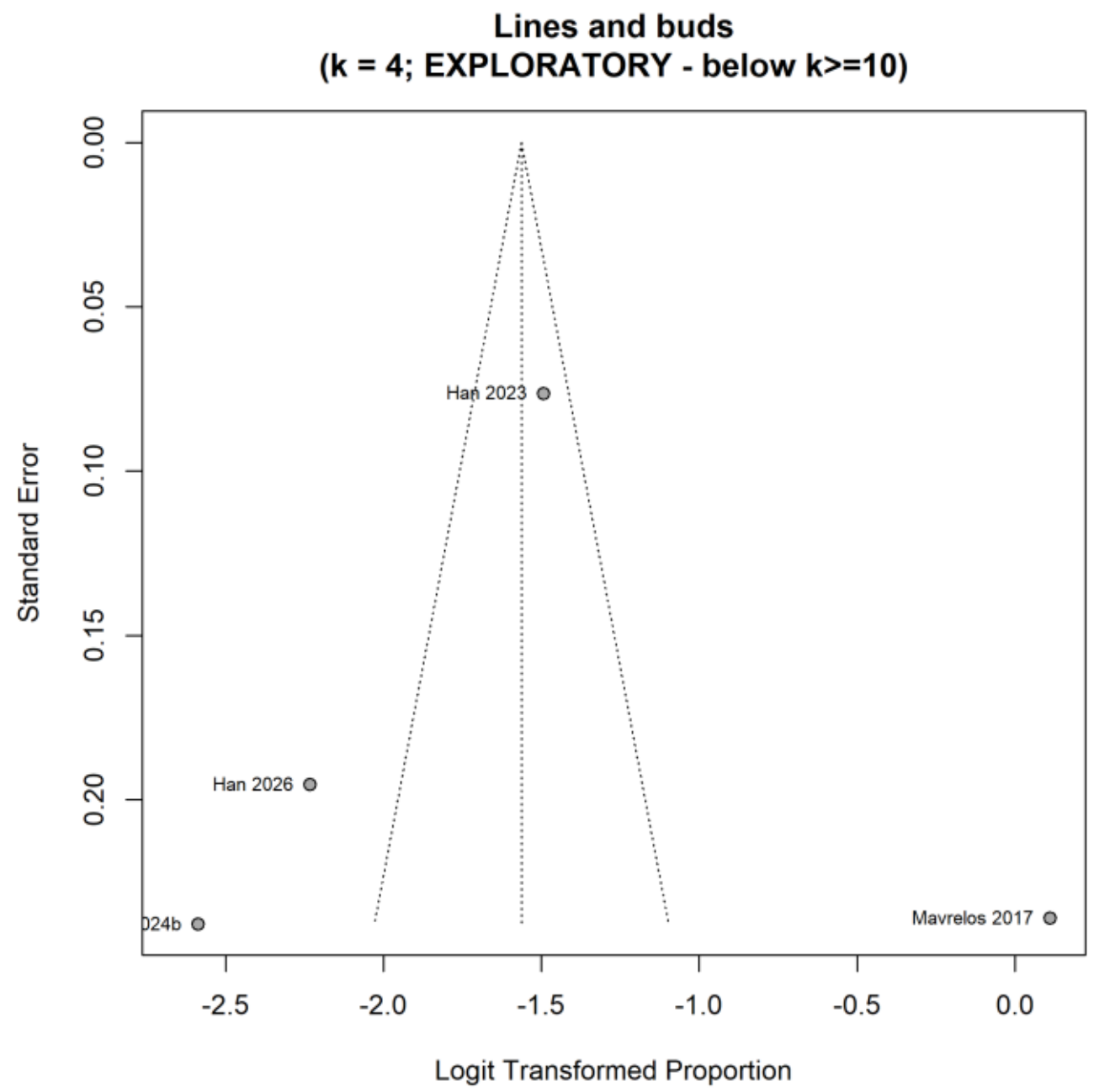

Fig. O: Asymmetrical thickening

All studies

Study	Cases	Total	Prevalence	95% CI	Weight
AbuHashim 2020	14	24	0.58	[0.37; 0.78]	--%
Alson 2024b	36	272	0.13	[0.09; 0.18]	--%
Arlier 2025	30	95	0.32	[0.22; 0.42]	--%
Dason 2023	49	76	0.64	[0.53; 0.75]	--%
Han 2023	1043	1146	0.91	[0.89; 0.93]	--%
Han 2026	85	300	0.28	[0.23; 0.34]	--%
Madani 2026	3	165	0.02	[0.00; 0.05]	--%
Mavrelos 2017	46	72	0.64	[0.52; 0.75]	--%
Neal 2020	5	99	0.05	[0.02; 0.11]	--%
Surcel 2026	18	35	0.51	[0.34; 0.69]	--%
Tokhunts	76	149	0.51	[0.43; 0.59]	--%
**Random effects model**		**2433**	**0.36**	**[0.17; 0.60]**	**100.0%**

Heterogeneity: I^2 = 98.7% [98.3%; 99.0%]

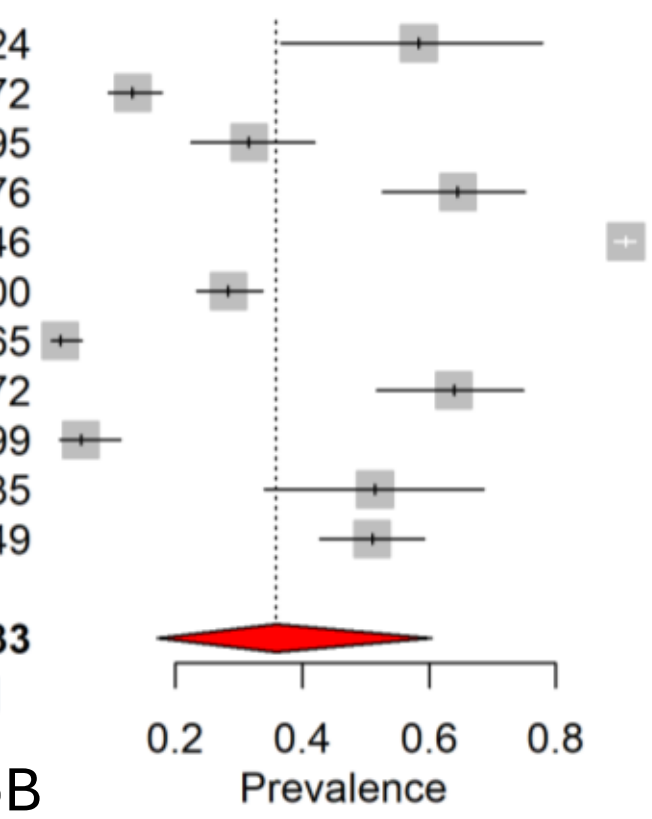

Only low and moderate RoB

Study	Cases	Total	Prevalence	95% CI	Weight
AbuHashim 2020	14	24	0.58	[0.37; 0.78]	--%
Alson 2024b	36	272	0.13	[0.09; 0.18]	--%
Han 2023	1043	1146	0.91	[0.89; 0.93]	--%
Han 2026	85	300	0.28	[0.23; 0.34]	--%
Madani 2026	3	165	0.02	[0.00; 0.05]	--%
Mavrelos 2017	46	72	0.64	[0.52; 0.75]	--%
Neal 2020	5	99	0.05	[0.02; 0.11]	--%
**Random effects model**		**2078**	**0.28**	**[0.08; 0.64]**	**100.0%**

Heterogeneity: I^2 = 99.2% [98.9%; 99.4%]

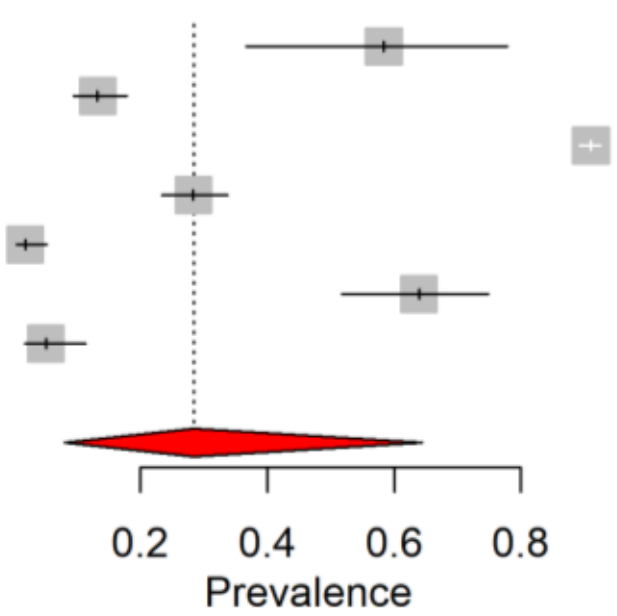

Leave one out

Omitted study	Prevalence	95% CI
Omitting AbuHashim 2020	0.34	[0.15; 0.60]
Omitting Alson 2024b	0.39	[0.18; 0.65]
Omitting Arlier 2025	0.36	[0.16; 0.63]
Omitting Dason 2023	0.33	[0.14; 0.59]
Omitting Han 2023	0.29	[0.14; 0.51]
Omitting Han 2026	0.37	[0.16; 0.64]
Omitting Madani 2026	0.44	[0.25; 0.65]
Omitting Mavrelos 2017	0.33	[0.14; 0.59]
Omitting Neal 2020	0.41	[0.21; 0.65]
Omitting Surcel 2026	0.34	[0.15; 0.61]
Omitting Tokhunts	0.34	[0.15; 0.61]
**Random effects model**	**0.36**	**[0.17; 0.60]**

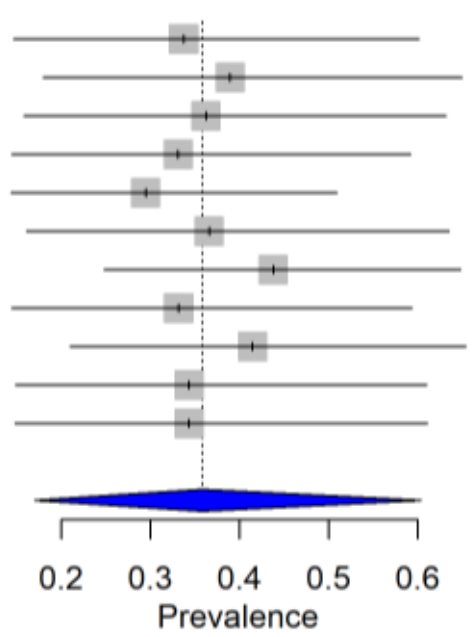

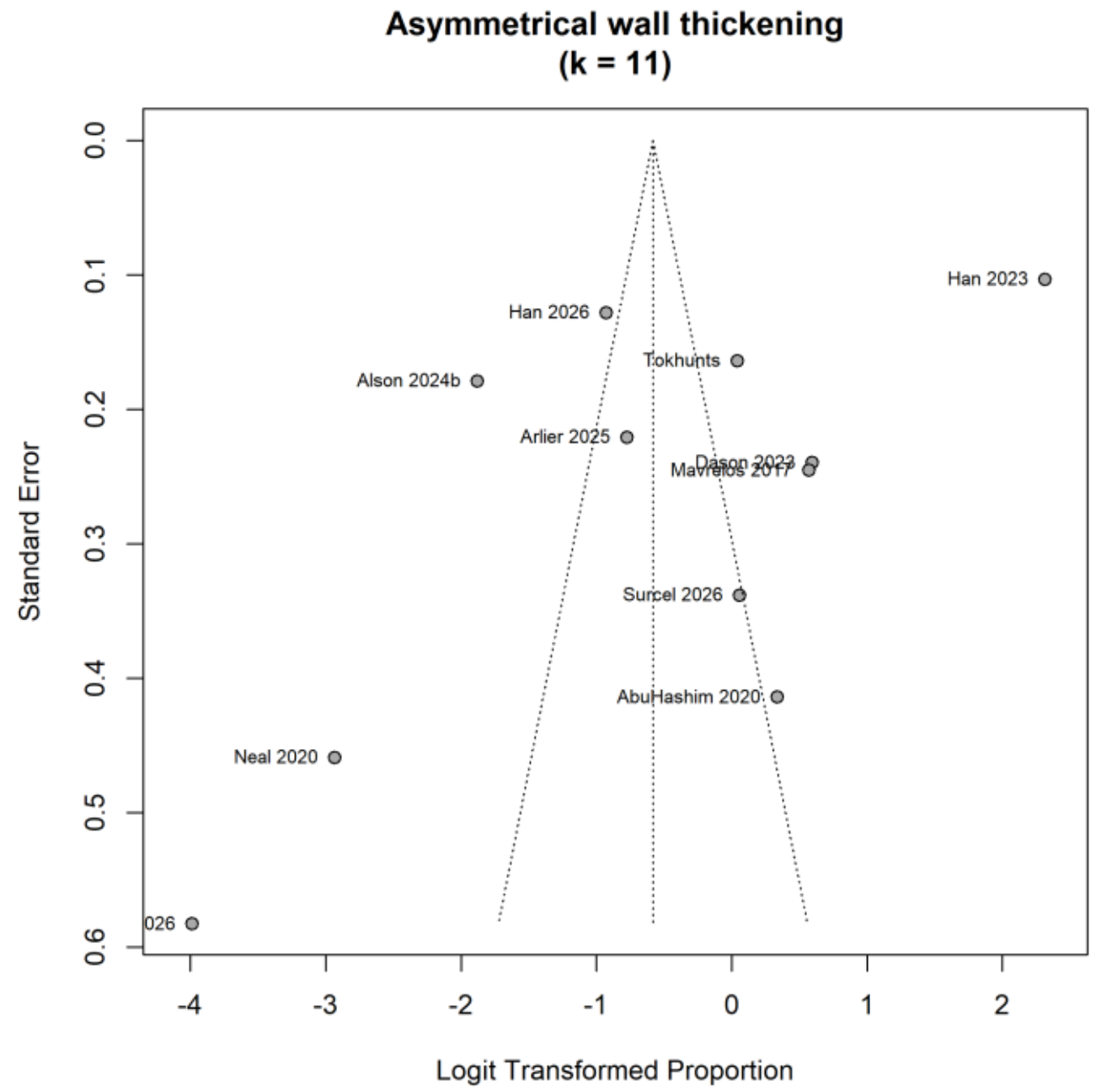

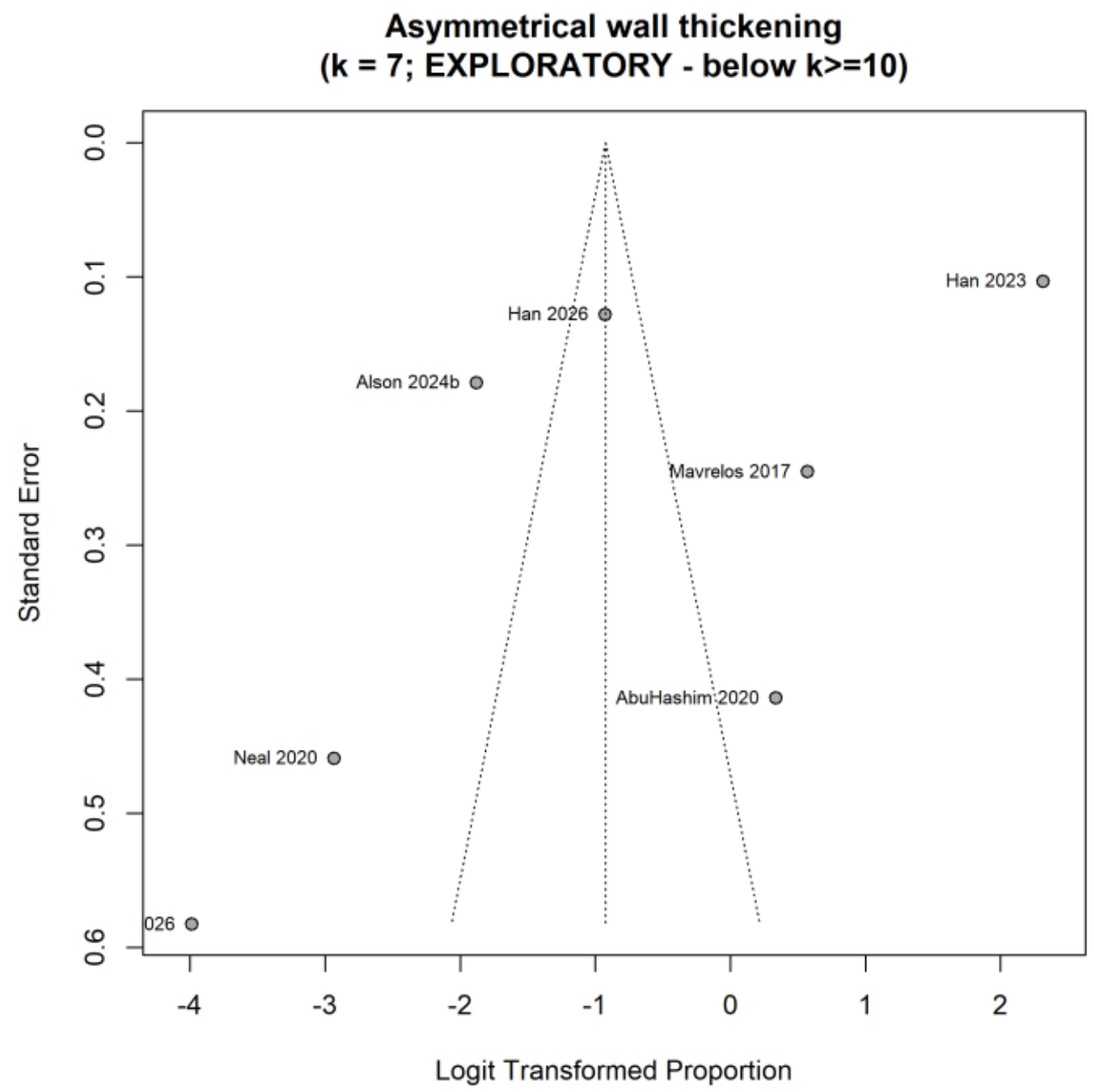

Fig. P: Globular uterus

All studies

Study	Cases	Total	Prevalence	95% CI	Weight
Alson 2024b	12	272	0.04	[0.02; 0.08]	--%
Arlier 2025	18	95	0.19	[0.12; 0.28]	--%
Dason 2023	68	76	0.89	[0.80; 0.95]	--%
Han 2023	738	1146	0.64	[0.62; 0.67]	--%
Han 2026	76	300	0.25	[0.21; 0.31]	--%
Madani 2026	18	165	0.11	[0.07; 0.17]	--%
Neal 2020	2	99	0.02	[0.00; 0.07]	--%
**Random effects model**		**2153**	**0.22**	**[0.06; 0.54]**	**100.0%**

Heterogeneity: I^2 = 98.6% [98.1%; 99.0%]

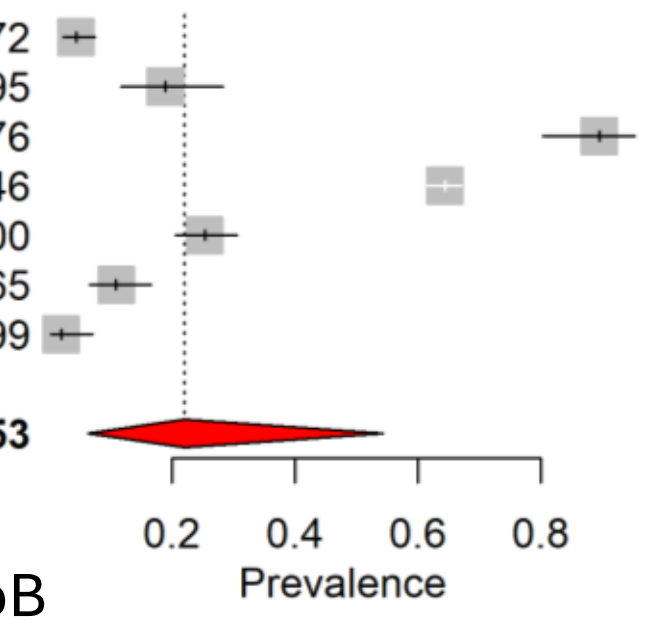

Only low and moderate RoB

Study	Cases	Total	Prevalence	95% CI	Weight
Alson 2024b	12	272	0.04	[0.02; 0.08]	--%
Han 2023	738	1146	0.64	[0.62; 0.67]	--%
Han 2026	76	300	0.25	[0.21; 0.31]	--%
Madani 2026	18	165	0.11	[0.07; 0.17]	--%
Neal 2020	2	99	0.02	[0.00; 0.07]	--%
**Random effects model**		**1982**	**0.13**	**[0.04; 0.38]**	**100.0%**

Heterogeneity: I^2 = 98.9% [98.4%; 99.2%]

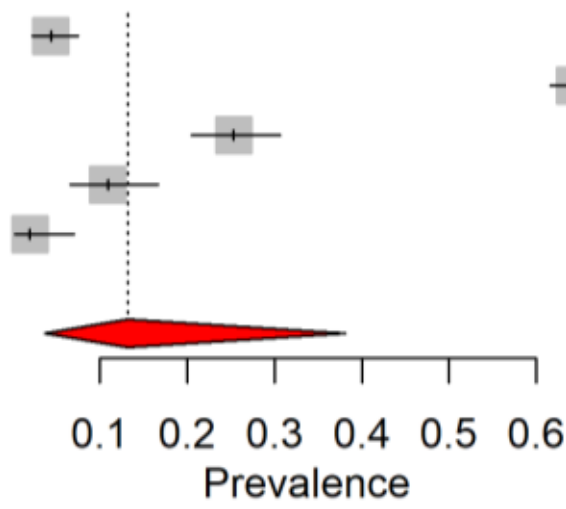

Leave one out

Omitted study	Prevalence	95% CI
Omitting Alson 2024b	0.28	[0.08; 0.64]
Omitting Arlier 2025	0.23	[0.05; 0.61]
Omitting Dason 2023	0.14	[0.05; 0.34]
Omitting Han 2023	0.17	[0.04; 0.49]
Omitting Han 2026	0.21	[0.05; 0.60]
Omitting Madani 2026	0.25	[0.06; 0.63]
Omitting Neal 2020	0.30	[0.10; 0.63]
**Random effects model**	**0.22**	**[0.06; 0.54]**

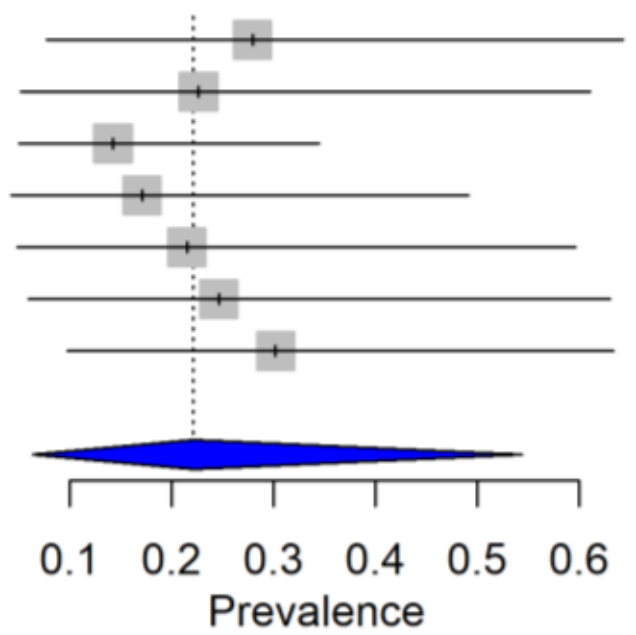

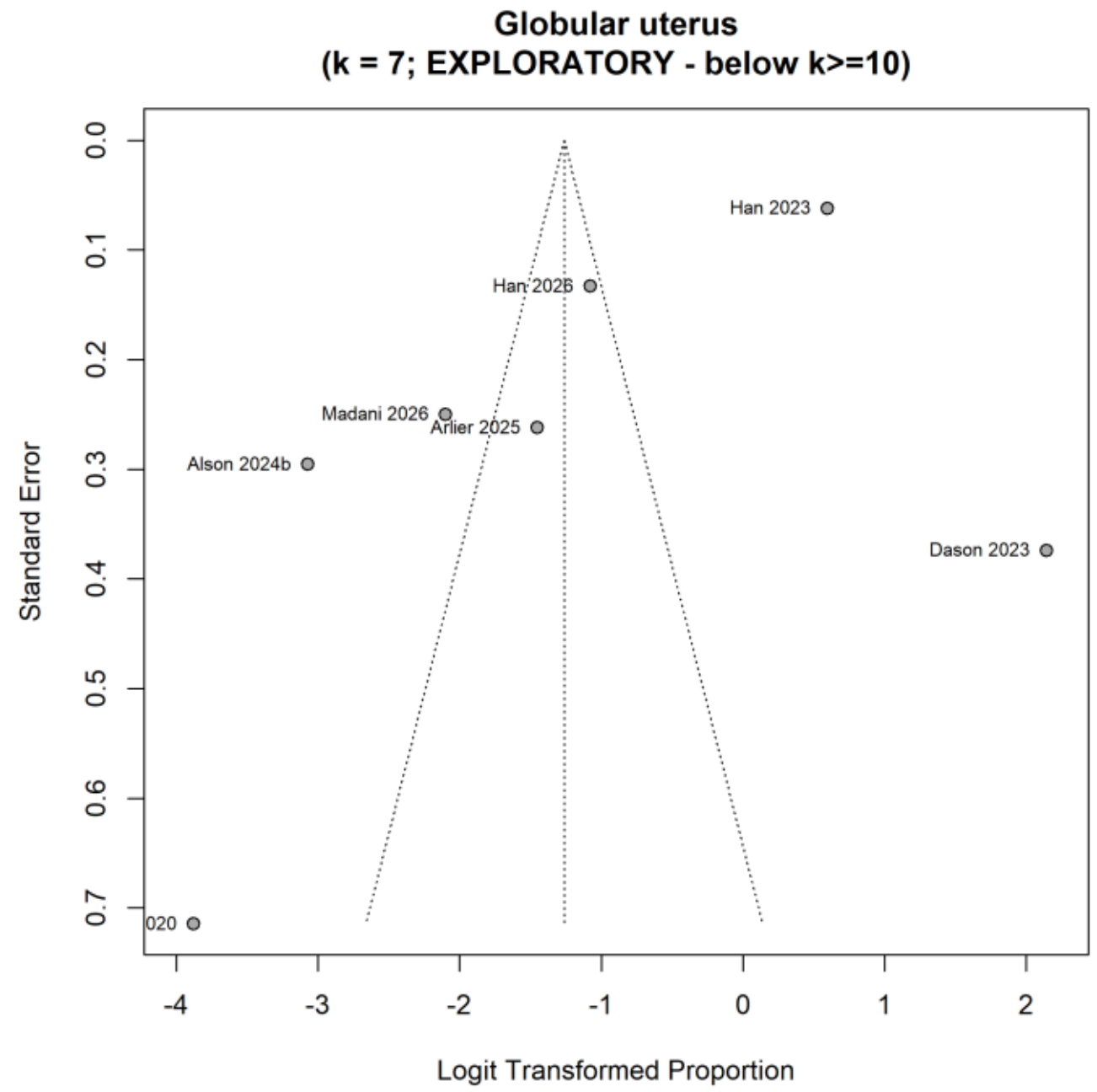

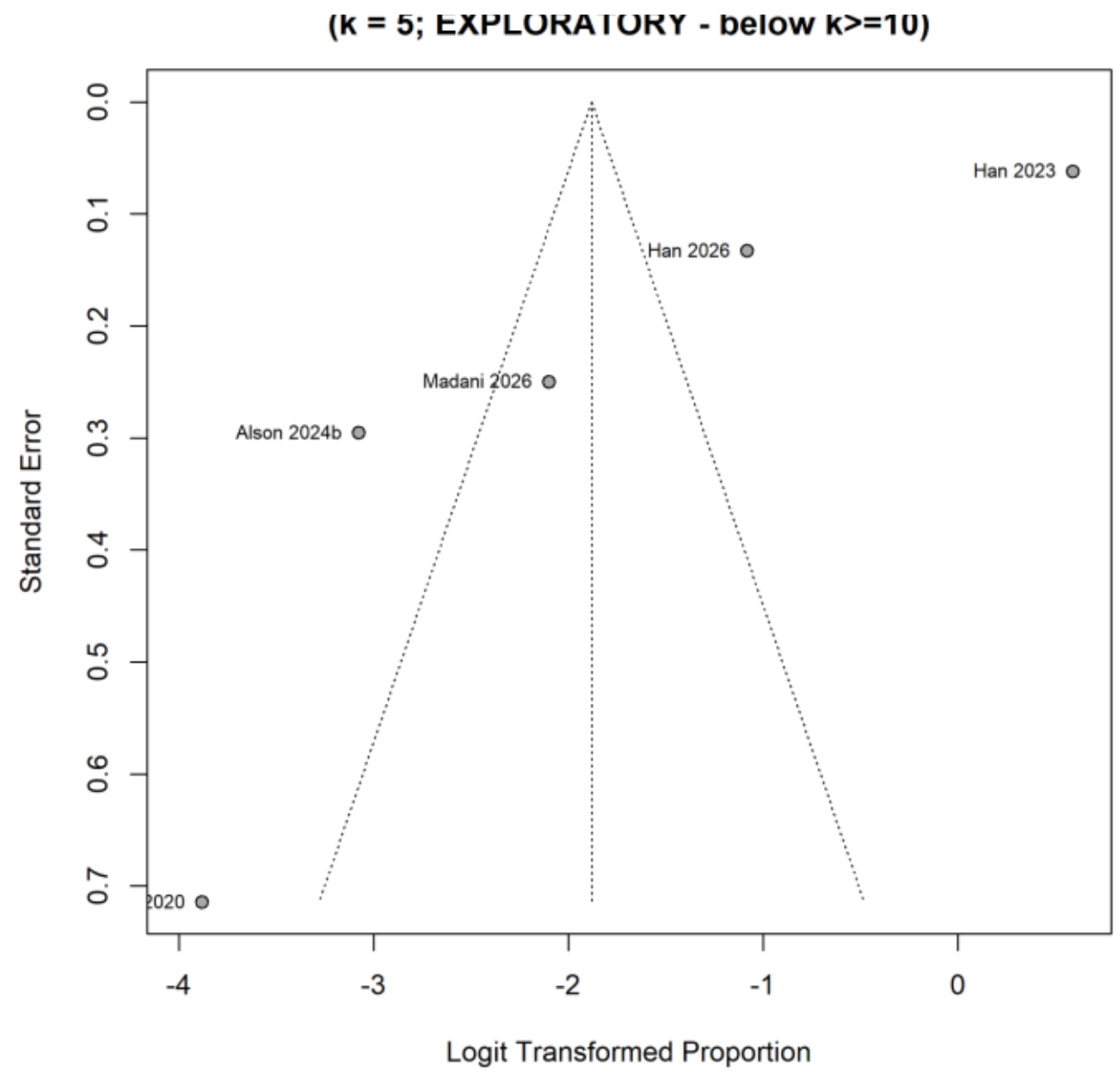

Fig. Q: Transleisional vascularity

All studies

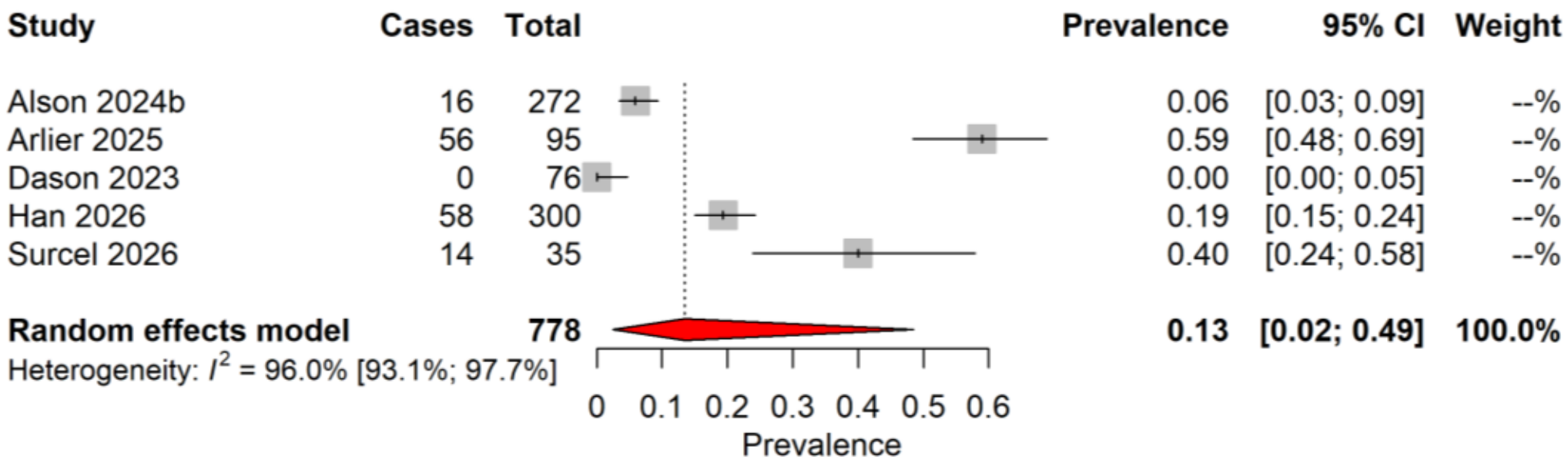

Only low and moderate RoB

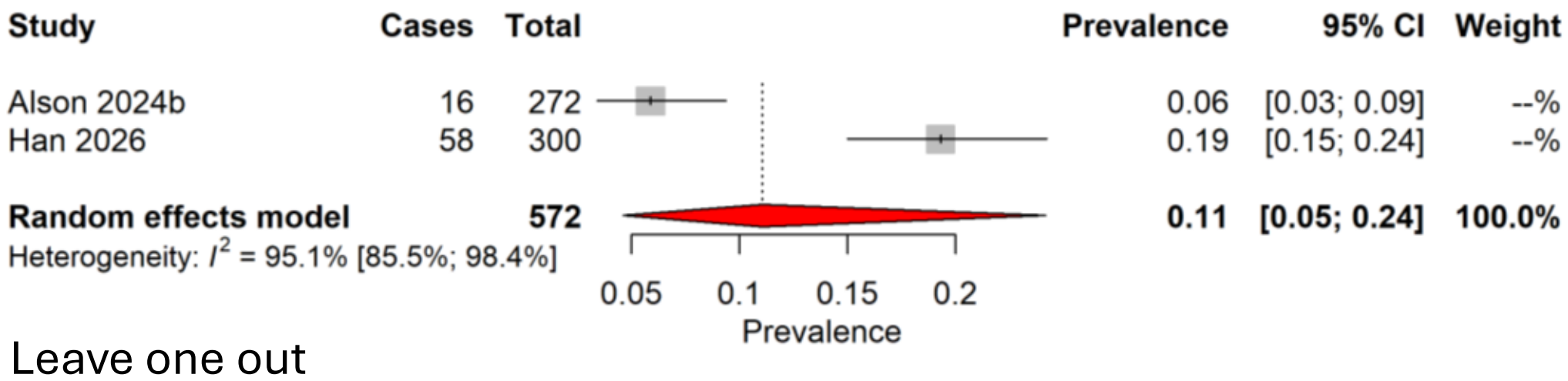

Leave one out

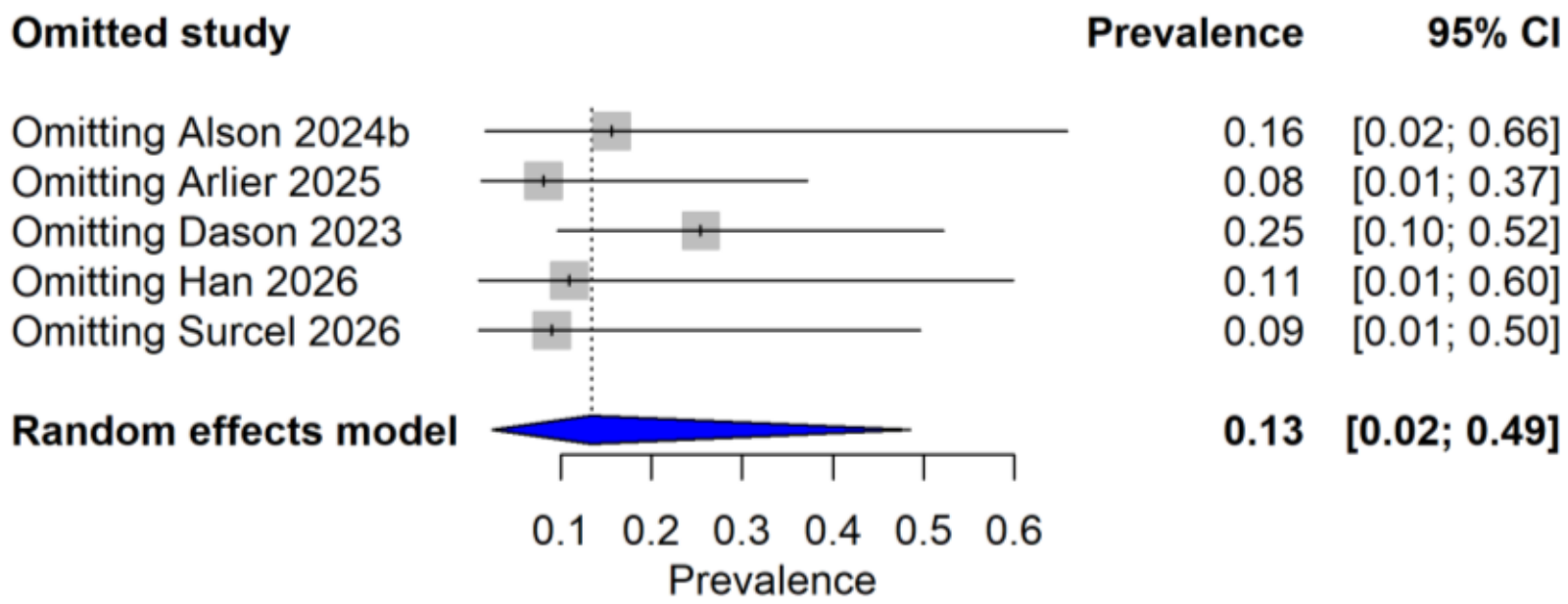

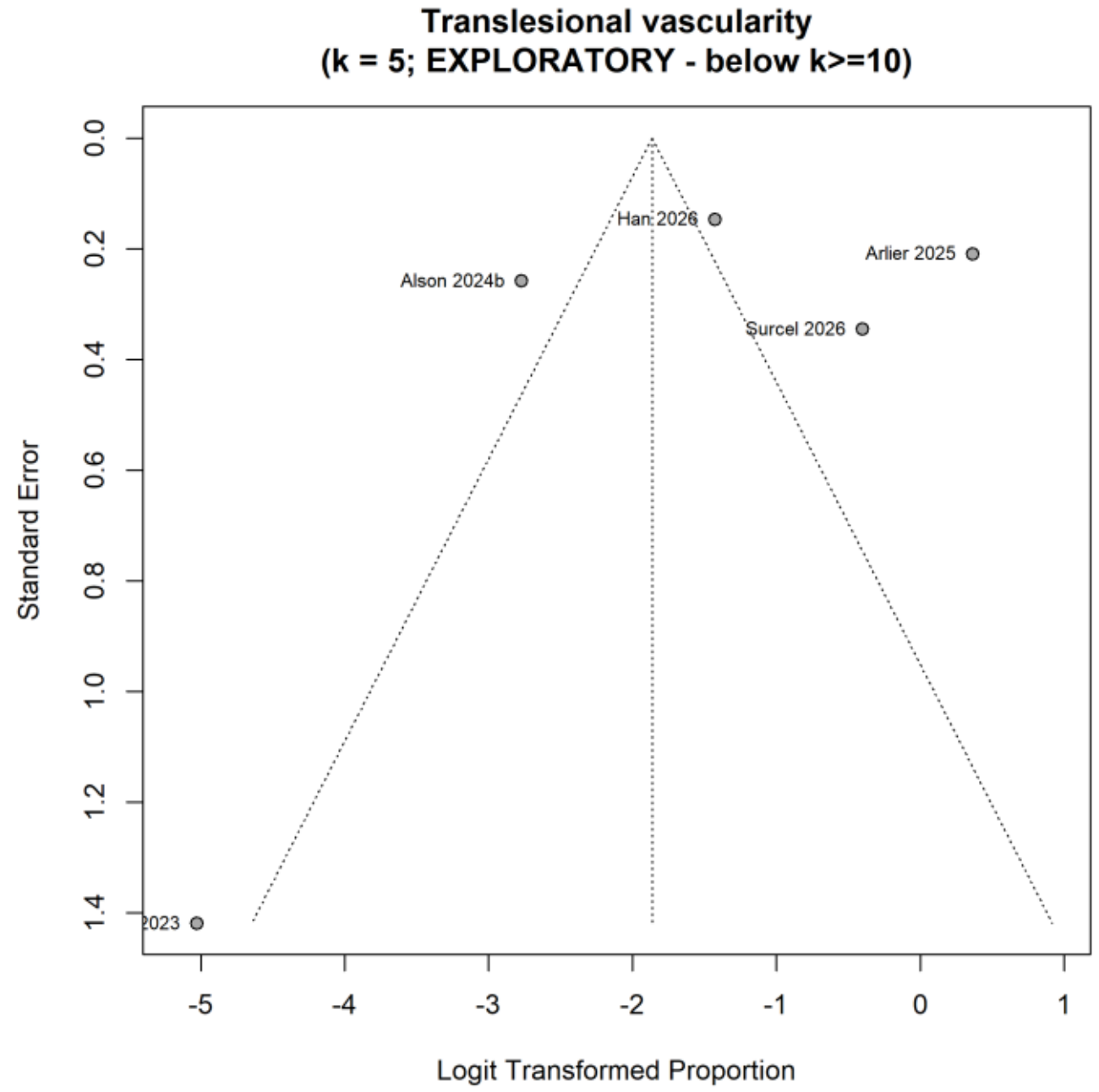

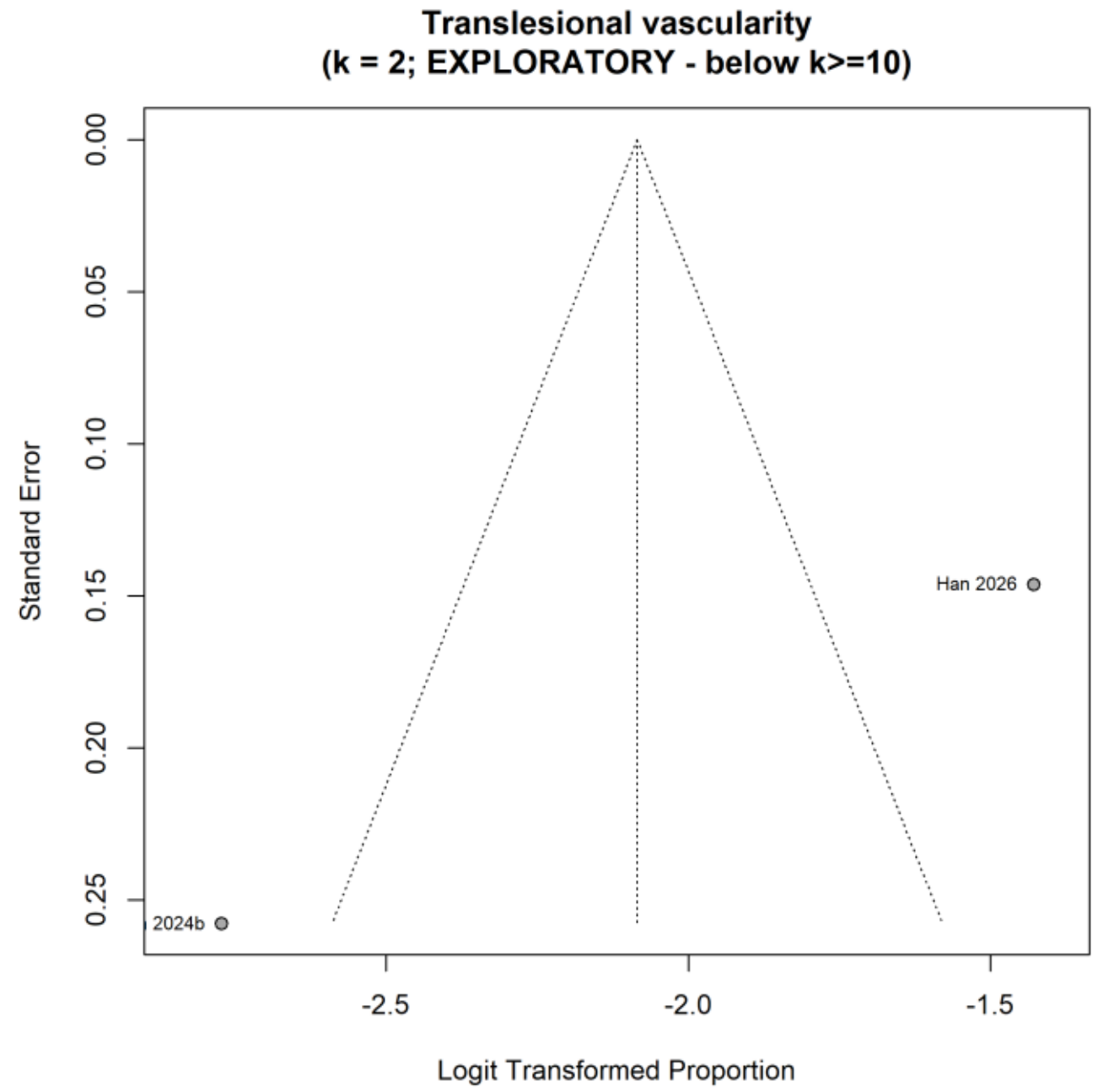

Fig. R: Fan shaped shadowing

All studies

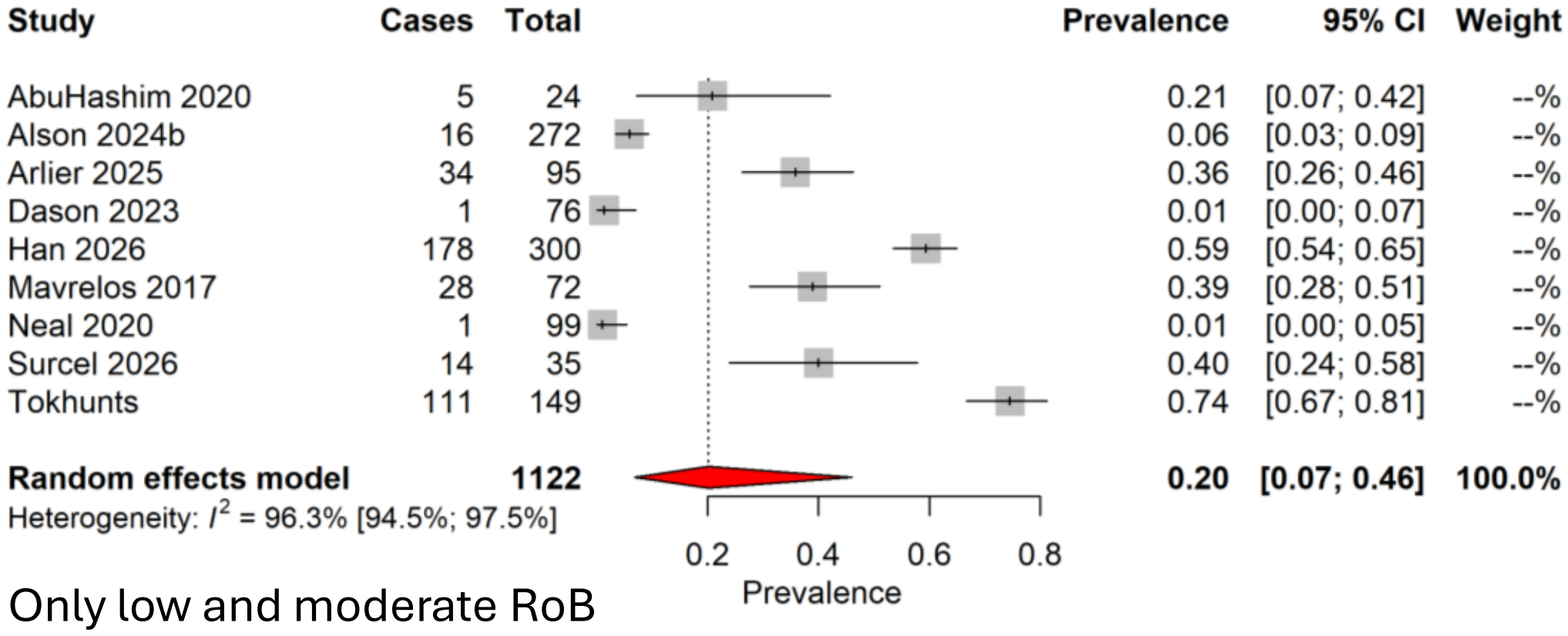

Study	Cases	Total	Prevalence	95% CI	Weight
AbuHashim 2020	5	24	0.21	[0.07; 0.42]	--%
Alson 2024b	16	272	0.06	[0.03; 0.09]	--%
Arlier 2025	34	95	0.36	[0.26; 0.46]	--%
Dason 2023	1	76	0.01	[0.00; 0.07]	--%
Han 2026	178	300	0.59	[0.54; 0.65]	--%
Mavrelos 2017	28	72	0.39	[0.28; 0.51]	--%
Neal 2020	1	99	0.01	[0.00; 0.05]	--%
Surcel 2026	14	35	0.40	[0.24; 0.58]	--%
Tokhunts	111	149	0.74	[0.67; 0.81]	--%
**Random effects model**		**1122**	**0.20**	**[0.07; 0.46]**	**100.0%**

Heterogeneity: I^2 = 96.3% [94.5%; 97.5%]

Only low and moderate RoB

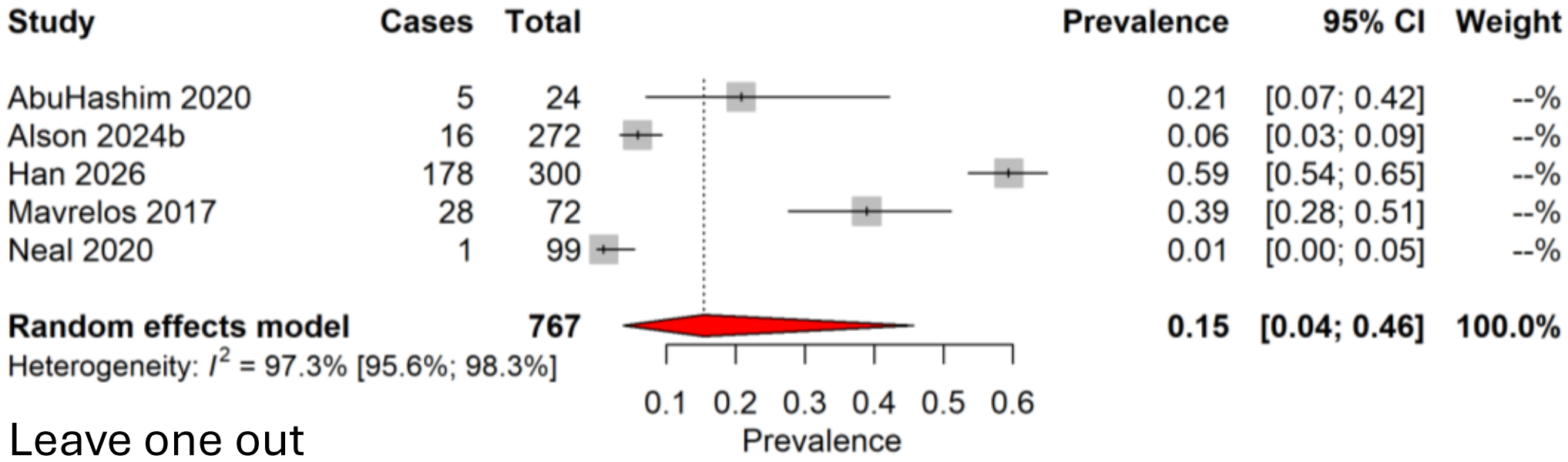

Study	Cases	Total	Prevalence	95% CI	Weight
AbuHashim 2020	5	24	0.21	[0.07; 0.42]	--%
Alson 2024b	16	272	0.06	[0.03; 0.09]	--%
Han 2026	178	300	0.59	[0.54; 0.65]	--%
Mavrelos 2017	28	72	0.39	[0.28; 0.51]	--%
Neal 2020	1	99	0.01	[0.00; 0.05]	--%
**Random effects model**		**767**	**0.15**	**[0.04; 0.46]**	**100.0%**

Heterogeneity: I^2 = 97.3% [95.6%; 98.3%]

Leave one out

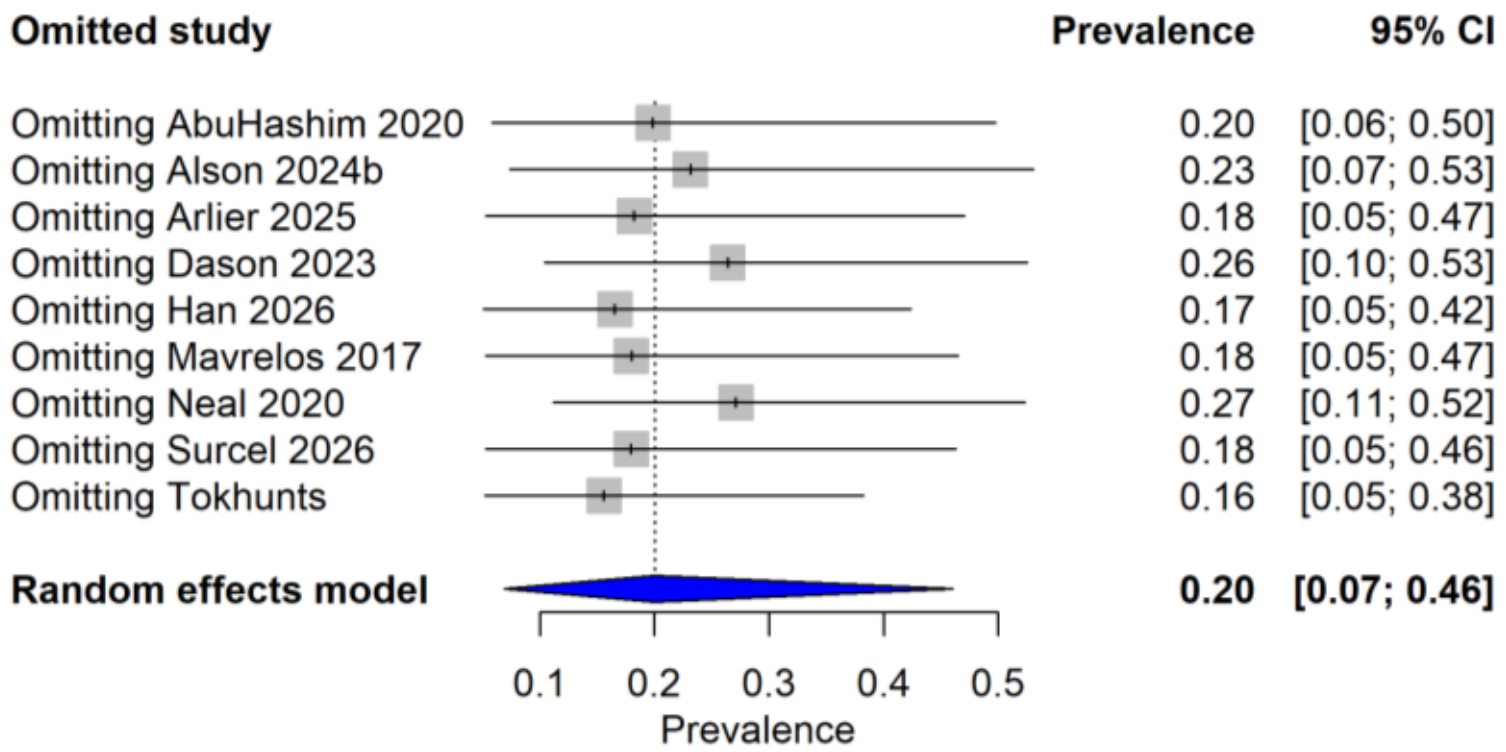

Omitted study	Prevalence	95% CI
Omitting AbuHashim 2020	0.20	[0.06; 0.50]
Omitting Alson 2024b	0.23	[0.07; 0.53]
Omitting Arlier 2025	0.18	[0.05; 0.47]
Omitting Dason 2023	0.26	[0.10; 0.53]
Omitting Han 2026	0.17	[0.05; 0.42]
Omitting Mavrelos 2017	0.18	[0.05; 0.47]
Omitting Neal 2020	0.27	[0.11; 0.52]
Omitting Surcel 2026	0.18	[0.05; 0.46]
Omitting Tokhunts	0.16	[0.05; 0.38]
**Random effects model**	**0.20**	**[0.07; 0.46]**

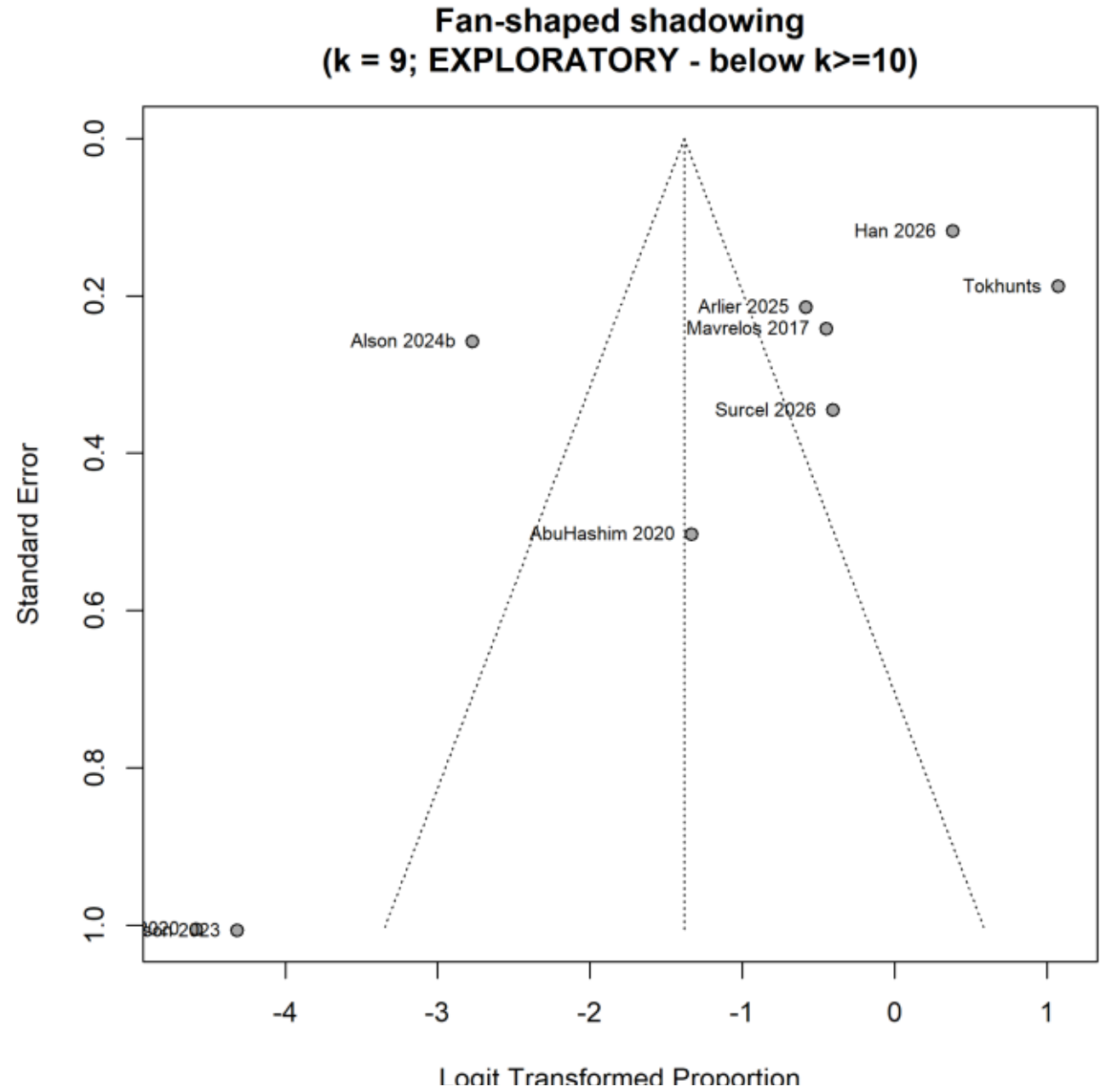

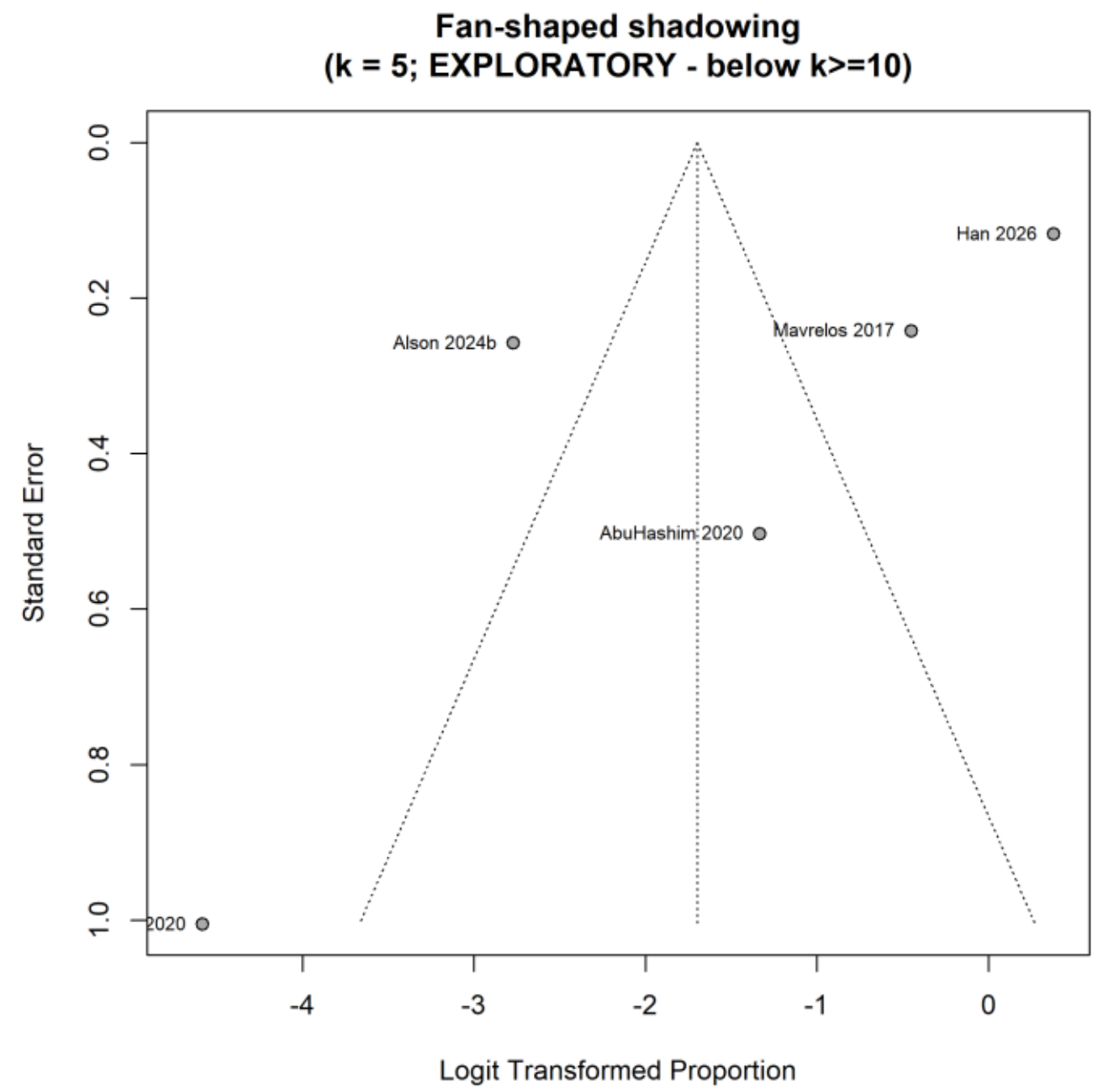

Fig. S: Irregular JZ

All studies

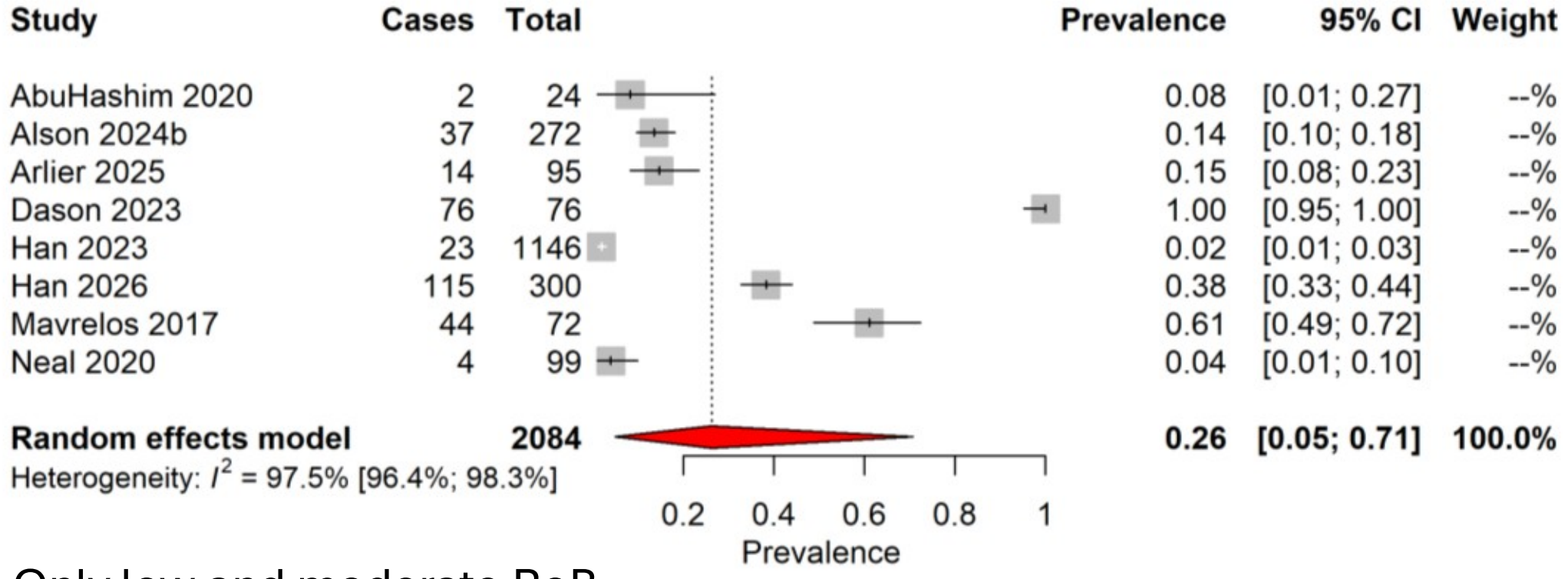

Only low and moderate RoB

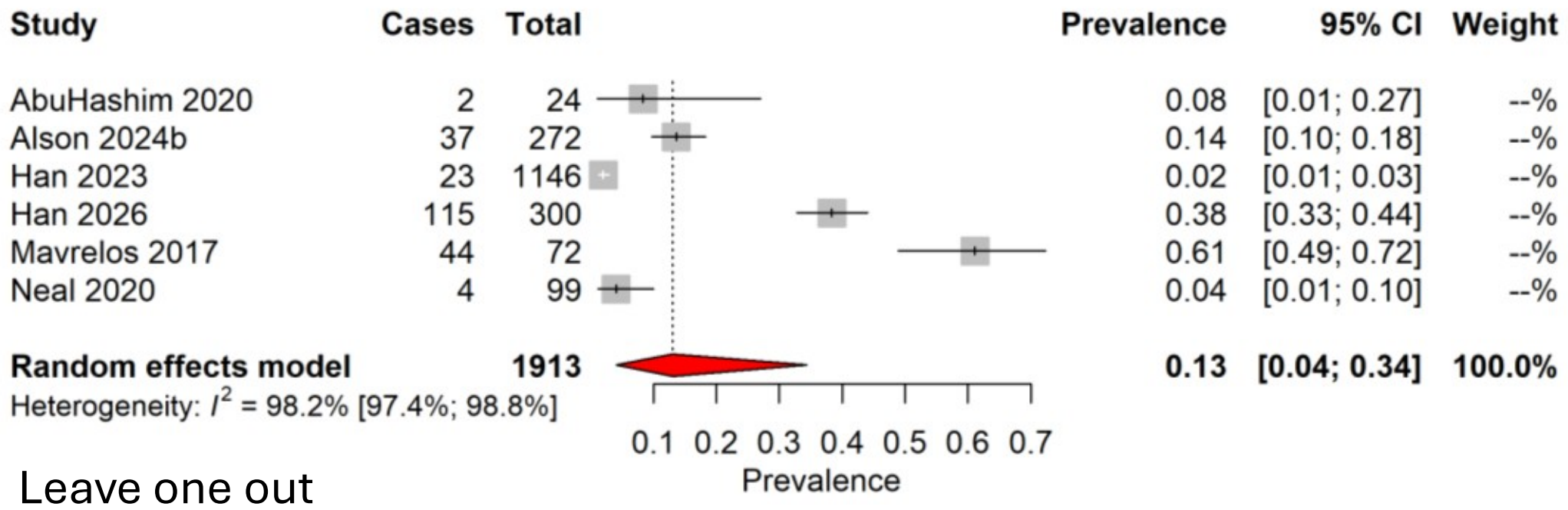

Leave one out

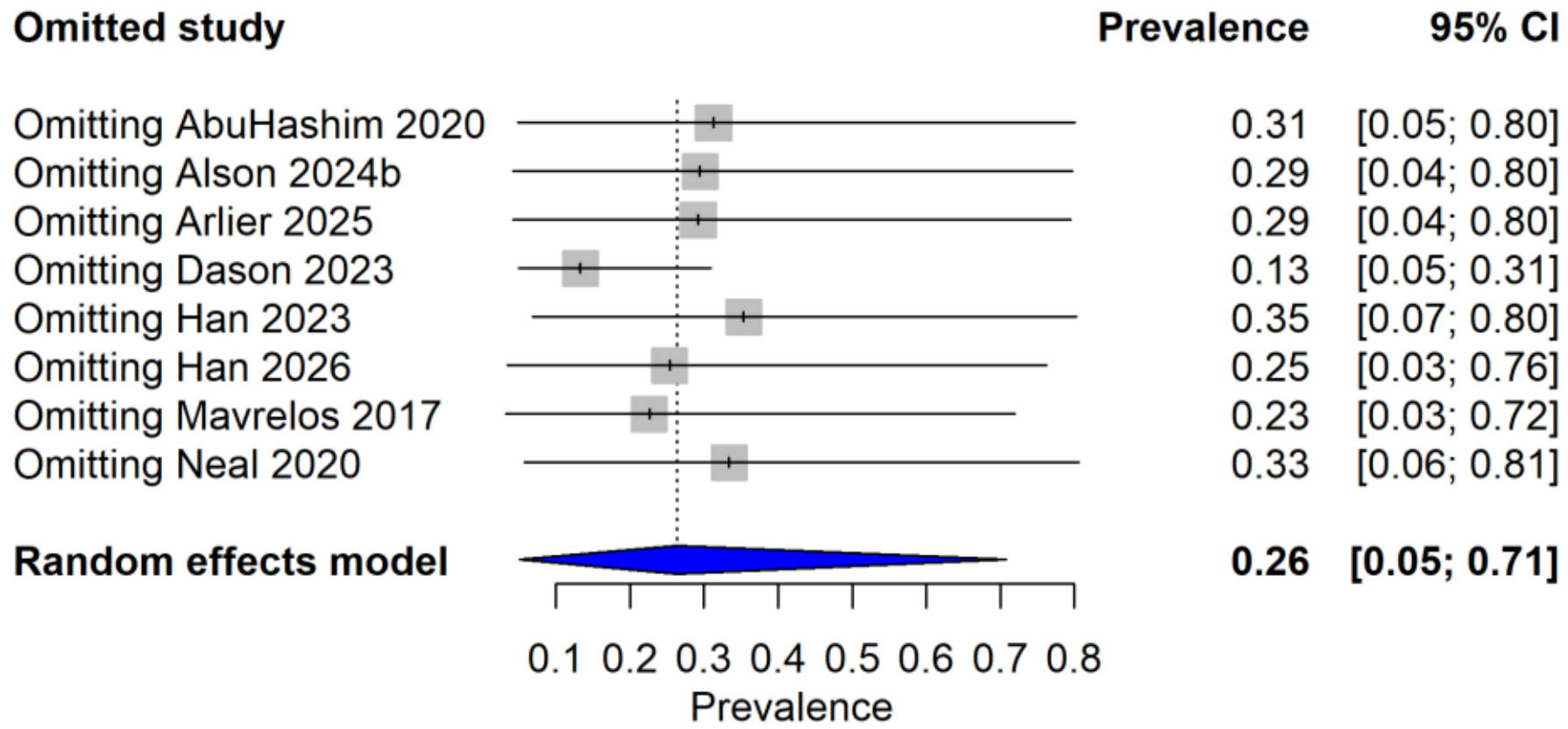

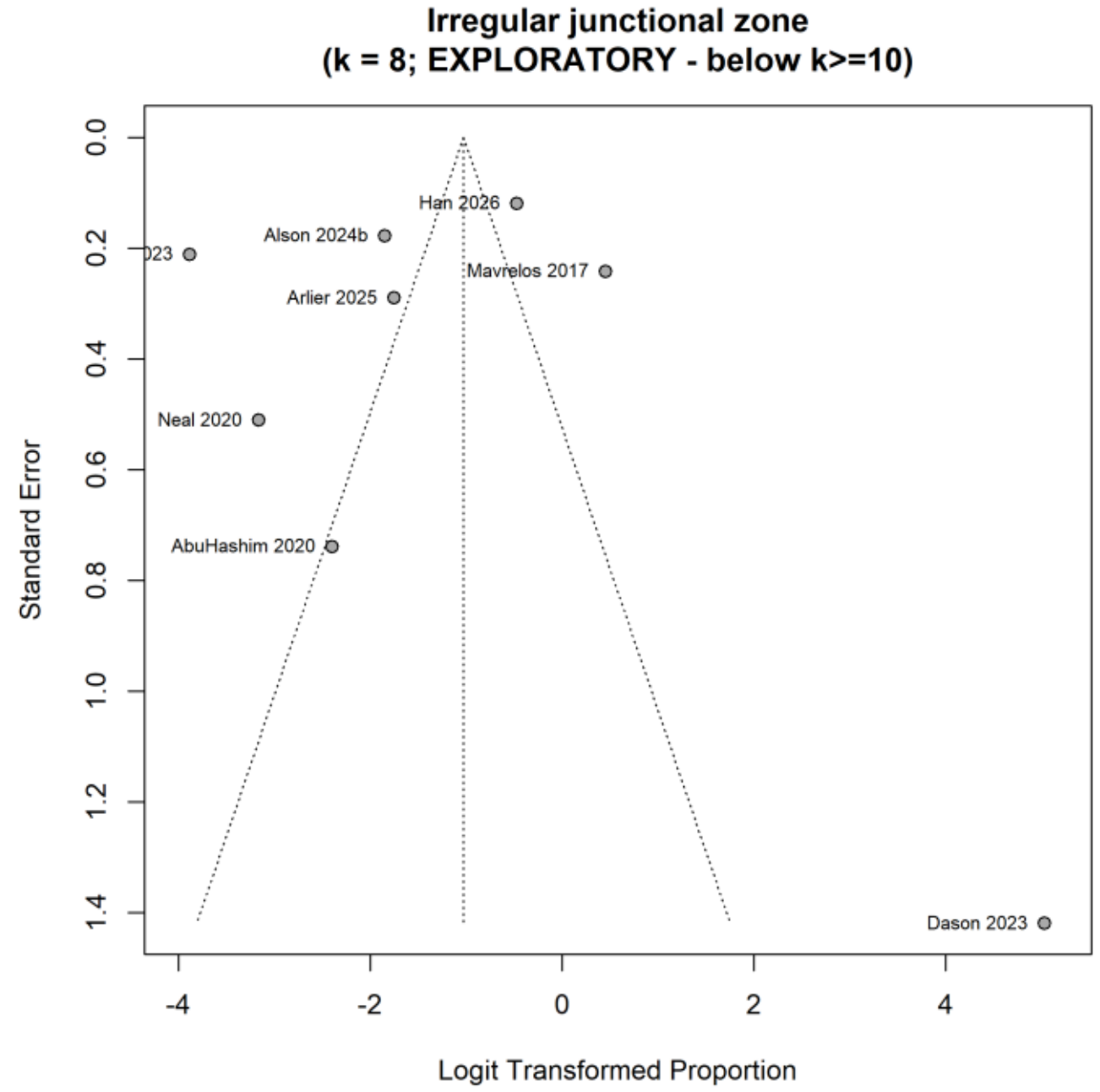

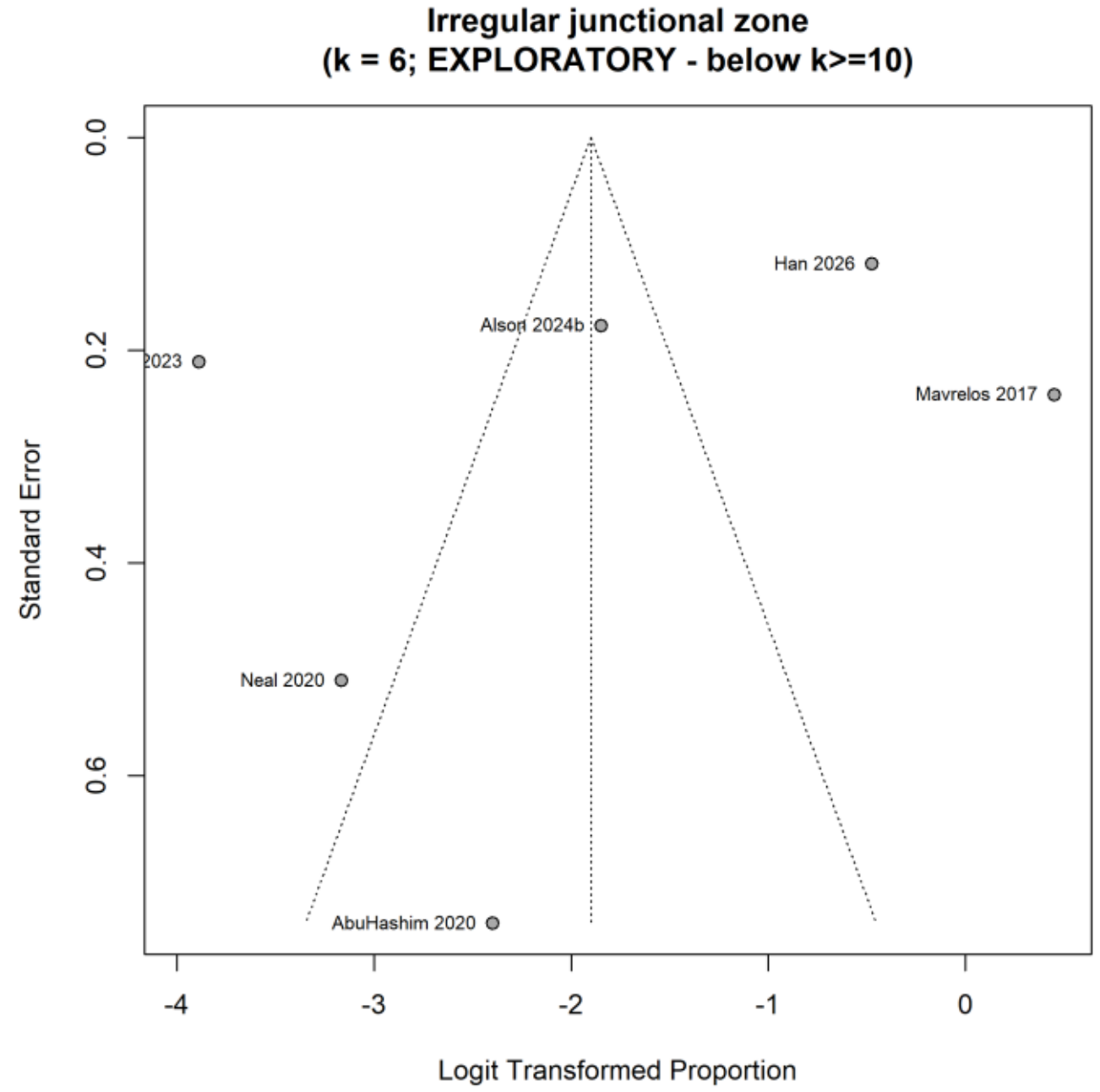

Fig. T: Interrupted JZ

All studies

Study	Cases	Total	Prevalence	95% CI	Weight
Alson 2024b	36	272	0.13	[0.09; 0.18]	--%
Arlier 2025	18	95	0.19	[0.12; 0.28]	--%
Dason 2023	76	76	1.00	[0.95; 1.00]	--%
Han 2026	62	300	0.21	[0.16; 0.26]	--%
**Random effects model**		**743**	**0.57**	**[0.04; 0.98]**	**100.0%**

Heterogeneity: I^2 = 46.4% [0.0%; 82.2%]

Only low and moderate RoB

Study	Cases	Total	Prevalence	95% CI	Weight
Alson 2024b	36	272	0.13	[0.09; 0.18]	--%
Han 2026	62	300	0.21	[0.16; 0.26]	--%
**Random effects model**		**572**	**0.17**	**[0.12; 0.23]**	**100.0%**

Heterogeneity: I^2 = 81.7% [22.6%; 95.7%]

Leave one out

Omitted study	Prevalence	95% CI
Omitting Alson 2024b	0.78	[0.03; 1.00]
Omitting Arlier 2025	0.77	[0.02; 1.00]
Omitting Dason 2023	0.17	[0.13; 0.22]
Omitting Han 2026	0.76	[0.02; 1.00]
**Random effects model**	**0.57**	**[0.04; 0.98]**

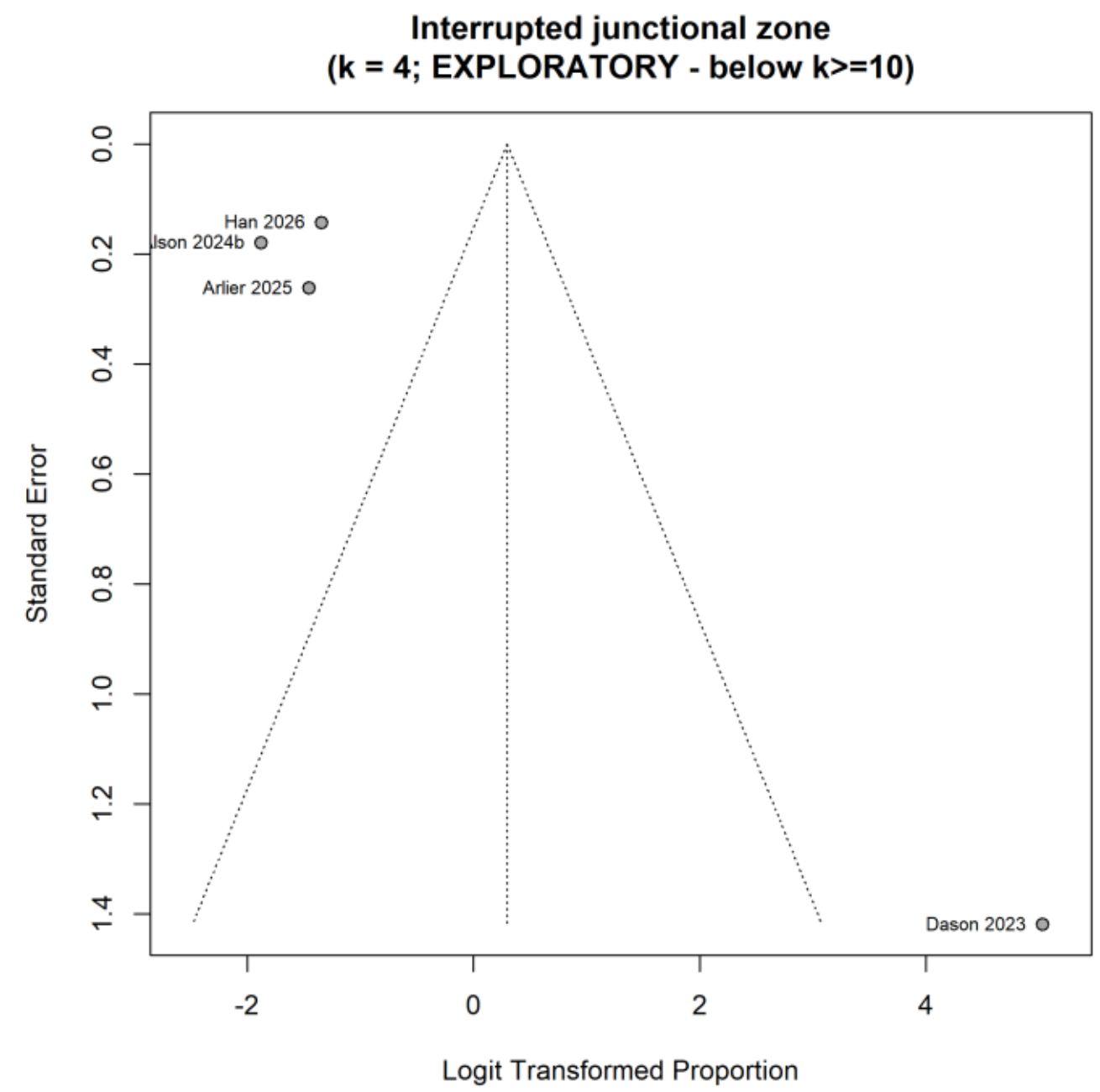

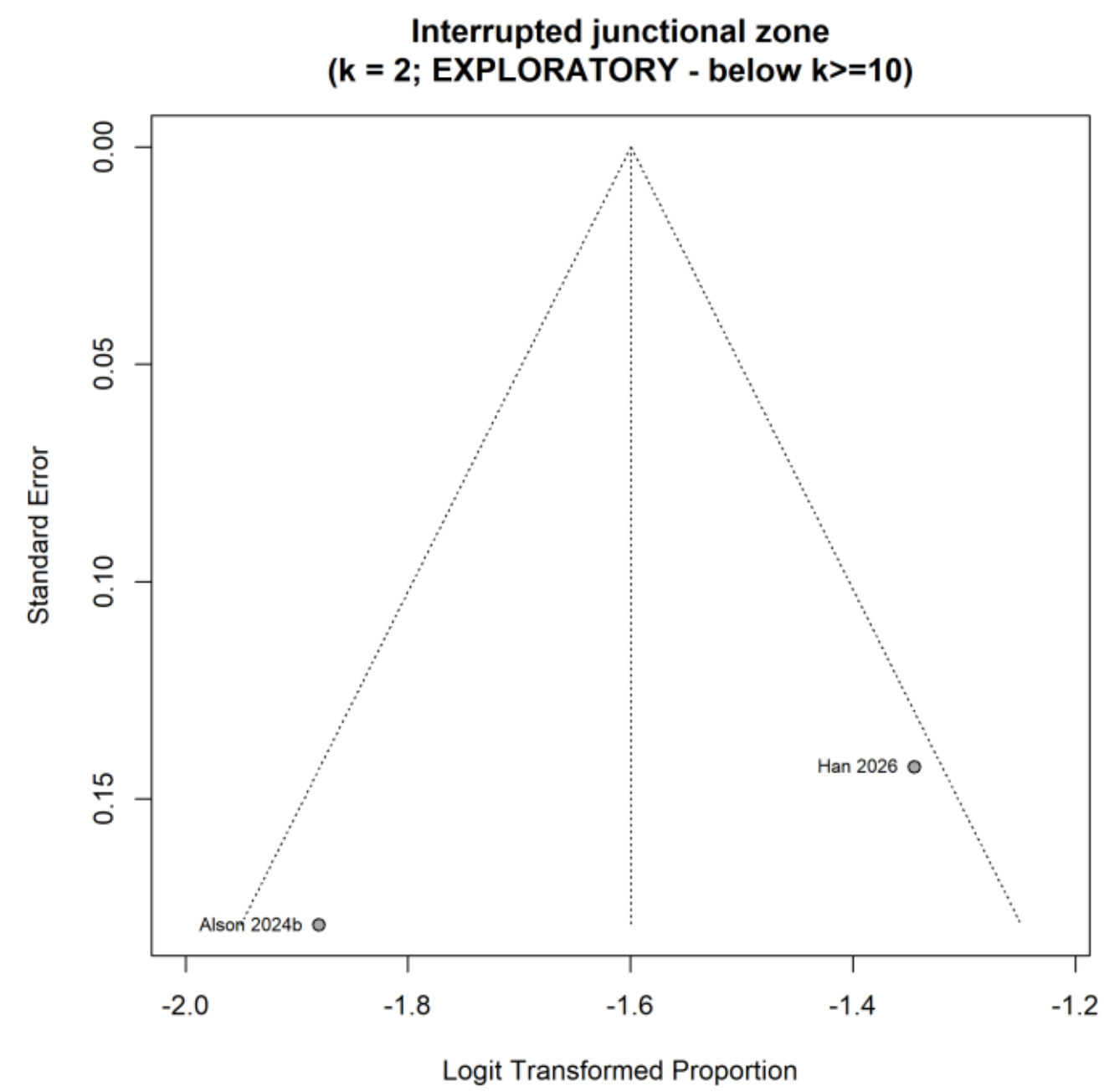

Supplement: Supplementary file 1 [file mmc2.pdf]
