# Supplementary material for: Adenomyosis and association between ultrasonographic features and pregnancy outcomes in women with infertility or pregnancy loss: a systematic review and meta-analysis
Source: eBioMedicine. 2026 Oct 21;133:106518. doi: 10.1016/j.ebiom.2026.106518 (PMC13716959; doi:10.1016/j.ebiom.2026.106518)
Supplement: Supplementary file 2 [file mmc1.docx]

# Adenomyosis and association Between Ultrasonographic Features and IVF Outcomes in Women With Infertility or Pregnancy Loss: A Systematic Review

**Registration** The protocol is registered in PROSPERO (CRD420251077005)

**Update** One similar systematic review from 2020 has been found in PROSPERO and published 2021: Fertility, pregnancy and neonatal outcomes of patients with adenomyosis: a systematic review and meta-analysis.

No similar systematic reviews were found in ZENODO (15.06.2025)

________________________________________________________________________________*Authors:*

Nicolaj Bruun Brandt (Nicolaj.brandt@rsyd.dk)*^1,2,3^, Cecilia Lindner (cecilia.ebba.clara.ellinor.lindner@regionh.dk)*^4-5^, Karen Høgh Abrahamsen (Karen.Hogh.Abrahamsen@rsyd.dk)^1,2^,Giuliana Beneduce (dott.ssagiulianabeneduce@gmail.com)^4,6^, Sofie Bliddal (anne.sofie.plesner.bliddal.mortensen@regionh.dk)^4-5^, Luisa Schaefer (luisa.schaefer@regionh.dk)^5^ Mette Nyegaard (nyegaard@hst.aau.dk)^7^, Steen Bonnema (steen.bonnema@rsyd.dk)^3^, Marianne Andersen (Marianne.Andersen1@rsyd.dk)^3^ Pernille Ravn (Pernille.Ravn@rsyd.dk)^2^ Dorte Glintborg (Dorte.Glintborg@rsyd.dk)^3^ Henriette Svarre (Henriette.Svarre.Nielsen@regionh.dk)^4-5^

*shared 1^st^ authorship

^1^ Department of Gynecology and Obstetrics, Odense University Hospital, Denmark

^2^ Department of Health, University of Southern Denmark

^3^ Department of Endocrinology, Odense University Hospital, Denmark.

^4^ Department of Obstetrics and Gynecology, Copenhagen University Hospital Hvidovre, DK-2650, Denmark

^5^ Department of Clinical Medicine, University of Copenhagen, Denmark.

^6^ Unit of Obstetrics and Gynecology- Università Cattolica del Sacro Cuore, Department of Women and Child Health- Women Health Area, Rome, Italy

^7^ Institute of Medicin and Health technology, Aalborg University, Denmark

Corresponding author:

Nicolaj Bruun Brandt – Nicolaj.bruun.brandt@rsyd.dk

Kloevervaenget 23,

5000 Odense C, DENMARK

________________________________________________________________________________*Contributions*

Author contributions: NBB, KHA, CL conceived the protocol and critically revised it with input from DG, GB, SB, PR, MN, SB and HS. CL and NBB screened the literature using Covidence with help from KHA. NBB and CL, built the tables and made the meta-analysis with LS.

All authors will have to approve the final version of the article. NBB is the guarantor of the review.

*Amendments:* N/A

________________________________________________________________________________*Support*

*Sponsor*

Main authors NBB and CLs salary are funded by the Novo Nordisk foundation.

*Role of sponsor or funder*

None of the funders or supporters have had any contributions to writing and submitting the protocol.

________________________________________________________________________________

**INTRODUCTION**

________________________________________________________________________________*Rationale*

Adenomyosis is a benign gynecological condition characterized by the presence of ectopic endometrial glands and stroma within the myometrium. Advances in imaging technology, particularly high-resolution transvaginal ultrasound (TVUS), have enabled in vivo diagnosis of adenomyosis with increasing reliability. TVUS is the most accessible and widely used modality in routine gynecological and fertility practice. Different systems of classification have been proposed for diagnosis, including the revised Morphological Uterus Sonographic Assessment (MUSA) criteria, inner or outer myometrial layer involvement, focal or diffuse and etc.

Historically considered a condition predominantly affecting multiparous women in their fourth and fifth decades, increasing evidence now indicates that adenomyosis can also be diagnosed in younger, reproductive-age women, many of whom present with infertility and recurrent pregnancy loss.

The pathophysiology linking adenomyosis to subfertility and adverse pregnancy outcomes remains incompletely understood. Proposed mechanisms include altered uterine contractility, chronic inflammation, impaired endometrial receptivity, and disruption of the junctional zone architecture, which may collectively hinder embryo implantation and early placental development. Recent studies suggest that certain ultrasound characteristics may have distinct clinical relevance in predicting reproductive outcomes, particularly infertility and early pregnancy loss. However, the heterogeneity of diagnostic criteria and study designs has limited the ability to draw definitive conclusions.

Several placebo controlled studies and concurrent systematic reviews and meta-analysis’ have sought to determine if different treatments of adenomyosis increases the chance of a live birth, however results are varying, highlighting the need to investigate if there are certain specific features of adenomyosis that influences chance of live birth more than others.

Given the growing clinical significance of adenomyosis in reproductive medicine and the evolving role of ultrasound in its diagnosis, there is a critical need to systematically examine the existing evidence on the relationship between specific ultrasound features of adenomyosis and reproductive outcomes.

This systematic review aims to identify and evaluate the different ultrasound-diagnosed features of adenomyosis and assess their individual association with infertility and pregnancy loss.

________________________________________________________________________________

**METHODS**

**________________________________________________________________________________**

**Eligibility criteria**

We define our eligibility criteria as stated below:

P: Women with pregnancy loss or infertility

I: Ultrasound examination describing presence of adenomyosis features: MUSA features, focal/diffuse, question mark sign, inner / outer myometrial involvement, adenomyoma.

C: Women with pregnancy loss or infertility with an ultrasound without any adenomyosis features

O: IVF outcomes: Biochemical pregnancy rate, clinical pregnancy rate (US confirmed), ongoing pregnancy rate (+12 weeks), Live birth rate, miscarriage rate

S: Prospective and retrospective observational cohorts and randomized controlled studies (meta analyses and literature reviews will be used as background information to make sure that relevant previous studies are included).

**Inclusion and exclusion criteria:**

Inclusion:

All original studies published from inception of database to June 10th 2025.

We will only include literature published in peer reviewed journals in the analysis.

Exclusion:

Women with adenomyosis and co-existing endometriosis, where data are not separately available for women with adenomyosis

Studies that does not report on specific ultrasound features in diagnosing adenomyosis

Studies without that does not report on desired outcome

Studies that only uses MRI.

Studies written in non-latin alphabet

Reviews, opinion papers

If only abstract is available.

I**nformation sources**

A research librarian provided guidance on structuring the literature search and selecting databases

Embase (Ovid), Medline (Ovid), Cochrane Library (Wiley) and Web of Science (Clarivate) and Scopus were searched and evaluated systematically according to the PRISMA search guideline[1] .

Grey literature (conference abstracts, preprints, clinical trial protocols) will be assessed to evaluate publication bias. We will include grey literature from Embase and Google scholar (the 200 newest entries).

________________________________________________________________________________**Search strategy**

PRISMAs guideline for protocols is used to ensure transparency. PRISMA’s guidelines are also used for literature search and systematic review[5, 6].

We are combining three search blocks. The first one is about infertility / miscarriage, the second is about adenomyosis and the third is about diagnostic criteria: ultrasound.

The search strategy is peer reviewed by NBB, CL and KHA. PRISMA Statement for Reporting Literature Searches in Systematic Reviews (PRISMA-S) is used[1].

Finally we will be using core articles to validate our search.

***Search block 1: Infertility / miscarriage***

Fertility disorder or infecundity or primary infertility or secondary infertility or uterine infertility or uterus factor infertility or uterus infertility or uterus-related infertility or absolute uterine infertility or IVF or in vitro fertilization or live birth rate or biochemical pregnancy or ongoing pregnancy or abortion, spontaneous or loss of a pregnancy or loss of pregnancy	or loss of the pregnancy or miscarriage or miscarriage of pregnancy or miscarriages or pregnancy loss or spontaneous pregnancy loss, recurrent pregnancy loss or habitual abort* or recurrent abortion or recurrent fetal loss or recurrent miscarriage or repeated miscarriage or repeated pregnancy loss

***Search block 2: Adenomyosis***

Adenomyosis or Adenomyoses or adenomyotic or adenomyosis uteri or MUSA, endometrial adenoma or endometriosis interna or adenomyoma or endometriosis stroma or endometriosis uterina or internal endometriosis or myometrial wall asymmetry or global uterine enlargement or heterogeneous echogenicity or irregular junctional zone or myometrial cysts or fan-shaped shadowing or myometrial lesion* or adenomyosis

***Search block 3: Ultrasound***

Transvaginal sonography or transvaginal echography or transvaginal ultrasonography or transvaginal ultrasound or vaginal echography or vaginal sonography or vaginal ultrasonography or vaginal ultrasound or three dimensional echography or 3 dimensional echography or 3D echography or 3D sonography or 3D ultrasonogram or 3D ultrasonography or 3D ultrasound or 3D-US or 3DUS or threedimensional sonography or threedimensional ultrasonic evaluation or threedimensional ultrasonography or threedimensional ultrasound or two dimensional echography or 2 dimensional echography or 2D echography or 2D sonography or 2D ultrasonogram or 2D ultrasonography or 2D ultrasound or 2D-US or 2DUS or twodimensional sonography or twodimensional ultrasonic evaluation or twodimensional ultrasonography or twodimensional ultrasound or gynaecological imag* or gynecological imag*

**Updating the searches**

Before submission to a journal, we will update the searches in order to include recently published studies, not available at the time of the original search.

________________________________________________________________________________**Study records**

**Data management:**

Records will be managed through Covidence and EndNote. Manuel screening of references and citation tracking will be performed on included studies with forward and backward citation search. This will be done in Web of Science or Scopus allowing us to manage the results in Covidence. References will be screened based on title/ abstract and full text level.

**Selection process**:

Two independent reviewers (NBB and CL) will complete the study selection. Screening will be done in Covidence. In Covidence merging and duplication removal will also be done.

In the screening process we will only exclude obviously irrelevant reports. We prefer to be overinclusive of potentially relevant reports. We will find full texts and exclude ineligible studies with reasons according the exclusion criteria. A third author KHA will resolve potential disagreements.

NBB and CL will extract data on study settings, study design, study participants/group characteristics, primary outcomes. Effect sizes will be calculated when possible.

________________________________________________________________________________**Data collection process**

Data are extracted in a Covidence. We will be extracting data in our customized data extraction template.

**Data items:**

Variables:

- Study:
  - Title, design, author, publication year and journal
- Participants:
  - number, population, demographics, setting, country, sites, inclusion and exclusion
- Exposure: Different features of adenomyosis including size of the uterus.
- Outcome:
  - Primary outcome: Miscarriage and Live birth rate
  - Secondary outcomes: Biochemical pregnancy rate, clinical pregnancy rate (US confirmed), ongoing pregnancy rate (+12 weeks),
- Publication status
- Extra

________________________________________________________________________________**Outcomes and prioritization**

Primary outcome is live birth- and miscarriage rate by specific adenomyosis features (MUSA features, questionmark sign, heterogeneous myometrium, junctional zone changes. The rationality is that these features can be assessed bedside by a sonographer and offer direct advise to the patient.

The secondary outcomes are: Live birth- and miscarriage rate according to uterine size, location of the adenomyosis features and if the sum of features might affect the outcome.

________________________________________________________________________________**Risk of bias in individual studies**

Two authors (NBB and CL.) will independently asses the included studies for risk of bias using the Newcastle-Ottawa Quality Assessment Scale (NOS) for cohort and case–control studies and the Cochrane ‘Risk of bias’ assessment tool for RCTs (i.e. the Risk of Bias 2 (RoB 2) tool). Results from studies with high risk of bias will be interpreted as such, with appropriate caution of results.

________________________________________________________________________________**Data synthesis**

Criteria: The aim is to group observational studies allowing us to compare the studies. If studies are sufficiently homogeneous in terms of design and comparator, we will conduct meta-analyses using a random-effects model.

If quantitative synthesis is possible:

- Dichotomous data will be determined by using odds ratio (OR) with 95% confidence interval (CI).
- Continuous outcomes will be analysed using weighted mean differences (with 95% CI) or standardized mean differences (95% CI) if different measurement scales are used. Skewed data and non-quantitative data will be presented descriptively.

If quantitative synthesis is not appropriate:

A qualitative data synthesis by will extracted on data on settings, demographics, study participants, adenomyosis features and primary study outcomes . Effect sizes will be calculated when possible.

________________________________________________________________________________**Meta-bias**

In order to determine whether reporting bias is present, we will determine whether the protocol of the RCT was published before recruitment of patients of the study was started. For studies published after July 1st 2005, we will screen the Clinical Trial Register at the International Clinical Trials Registry Platform of the World Health Organisation (http://apps.who.int/trialssearch). We will evaluate whether selective reporting of outcomes is present (outcome reporting bias).

________________________________________________________________________________**Confidence in cumulative evidence**

The quality of evidence for all outcomes will be judged using the NOS for cohort studies and rob2 for randomizes trials.

**Acknowledgements**

A special thanks to the library at University of Southern Denmark with evolving and refining the search string. There were no conflicts of interest in this and no funding was provided for the support.

**PRISMA flowchart systematic reviews.**

Studies included in quantitative synthesis (meta-analysis)
(n =	)

Records excluded
(n =	)

Records screened
(n =	)

Records after duplicates removed
(n =	)

## Identification

Additional records identified through other sources
(n =	)

Records identified through database searching
(n =	)

## Screening

Full-text articles assessed for eligibility
(n =	)

Full-text articles excluded, with reasons
(n =	)

## Eligibility

Studies included in qualitative synthesis
(n =	)

## Included

References:

1.	Rethlefsen, M.L., et al., *PRISMA-S: an extension to the PRISMA Statement for Reporting Literature Searches in Systematic Reviews.* Syst Rev, 2021. **10**(1): p. 39.

2.	Gambadauro, P., M. Milenkovic, and G. Hadlaczky, *Simulation for Training and Assessment in Hysteroscopy: A Systematic Review.* J Minim Invasive Gynecol, 2018. **25**(6): p. 963-973.

3.	Research, N.N.I.f.H.a.C. *PROSPERO International prospective register of systematic reviews*. 2024 [cited 2024 28.02.24]; Available from: <https://www.crd.york.ac.uk/PROSPERO/>.

4.	Prathiba De Silva, H.S., Paul Smith, T. Justin Clark., *Training and standards in outpatient hysteroscopy*. 2019, NIHR National Institute for Health and Care Research: PROSPERO.

5.	Page, M.J., et al., *The PRISMA 2020 statement: an updated guideline for reporting systematic reviews.* Bmj, 2021. **372**: p. n71.

6.	Moher, D., et al., *Preferred reporting items for systematic review and meta-analysis protocols (PRISMA-P) 2015 statement.* Syst Rev, 2015. **4**(1): p. 1.

7.	Reed, D.A., et al., *Association between funding and quality of published medical education research.* Jama, 2007. **298**(9): p. 1002-9.
