# Supplementary material for: Adenomyosis and association between ultrasonographic features and pregnancy outcomes in women with infertility or pregnancy loss: a systematic review and meta-analysis
Source: eBioMedicine. 2026 Oct 21;133:106518. doi: 10.1016/j.ebiom.2026.106518 (PMC13716959; doi:10.1016/j.ebiom.2026.106518)
Supplement: Supplementary file 3 [file mmc3.docx]

Supplementary tables.

**Supplementary Table A. Search string	2**

Updated Literature Search — 11 May 2026	2

MEDLINE / PubMed	2

Embase (Ovid)	2

Cochrane Library	3

Scopus	4

Web of Science	5

Google Scholar	6

First Literature Search — 26 June 2025	6

MEDLINE	6

Embase	7

Cochrane Library	8

Web of Science	9

Scopus	10

Google Scholar	11

**Supplementary Table B. Extracted crude prevalences and odds ratios	12**

**Supplementary Table C. Studies excluded at full-text screening	37**

**Supplementary Table D. Risk of Bias - Newcastle Ottawa scale	53**

**Supplementary Table E. Risk of Bias - Joan Briggs Institute	55**

**Supplementary Table F. GRADE assessment	56**

# Supplementary Table A. Search string

## Updated Literature Search — 11 May 2026

The original search strategies were re-run on 11 May 2026 with date restrictions applied from 27 June 2025 onward where possible.

### **MEDLINE / PubMed**

| **Database** | **Search string** |
| --- | --- |
| MEDLINE/PubMed | ((infertility OR "fertility disorder" OR infecundity OR "primary infertility" OR "secondary infertility" OR "uterine infertility" OR "uterus factor infertility" OR "uterus infertility" OR "uterus-related infertility" OR "absolute uterine infertility" OR IVF OR "in vitro fertilization" OR "live birth rate" OR "biochemical pregnancy" OR "ongoing pregnancy" OR abortion OR "spontaneous abortion" OR miscarriage OR "pregnancy loss" OR "loss of pregnancy" OR "loss of the pregnancy" OR "miscarriage of pregnancy" OR miscarriages OR "spontaneous pregnancy loss" OR "recurrent pregnancy loss" OR "habitual abortion" OR "recurrent abortion" OR "recurrent fetal loss" OR "recurrent miscarriage" OR "repeated miscarriage" OR "repeated pregnancy loss")) AND (adenomyosis OR adenomyoses OR adenomyotic OR "adenomyosis uteri" OR MUSA OR "endometrial adenoma" OR "endometriosis interna" OR adenomyoma OR "endometriosis stroma" OR "endometriosis uterina" OR "internal endometriosis" OR "myometrial wall asymmetry" OR "global uterine enlargement" OR "heterogeneous echogenicity" OR "irregular junctional zone" OR "myometrial cysts" OR "fan-shaped shadowing" OR "myometrial lesion" OR "myometrial lesions") AND (ultrasound OR sonography OR ultrasonography OR "transvaginal sonography" OR "transvaginal ultrasound" OR "transvaginal ultrasonography" OR "transvaginal echography" OR "vaginal ultrasound" OR "vaginal sonography" OR "vaginal ultrasonography" OR "3D ultrasound" OR "3-dimensional ultrasound" OR "3D sonography" OR "3D ultrasonography" OR "2D ultrasound" OR "2-dimensional ultrasound" OR "2D sonography" OR "2D ultrasonography" OR "gynecological imaging" OR "gynaecological imaging") AND ("2025/06/27"[Date - Publication] : "3000"[Date - Publication]) |

### **Embase (Ovid)**

| **#** | **Query** | **Results** |
| --- | --- | --- |
| 1 | exp infertility/ |  |
| 2 | miscarriage.mp. or exp spontaneous abortion/ |  |
| 3 | (fertility disorder or infecundity or primary infertility or secondary infertility or uterine infertility or uterus factor infertility or uterus infertility or uterus-related infertility or absolute uterine infertility or IVF or in vitro fertilization or live birth rate or biochemical pregnancy or ongoing pregnancy or abortion, spontaneous or loss of a pregnancy or loss of pregnancy or loss of the pregnancy or miscarriage or miscarriage of pregnancy or miscarriages or pregnancy loss or spontaneous pregnancy loss or recurrent pregnancy loss or habitual abort* or recurrent abortion or recurrent fetal loss or recurrent miscarriage or repeated miscarriage or repeated pregnancy loss).mp. |  |
| 4 | 1 or 2 or 3 |  |
| 5 | exp adenomyosis/ |  |
| 6 | (adenomyosis or adenomyoses or adenomyotic or adenomyosis uteri or MUSA or endometrial adenoma or endometriosis interna or adenomyoma or endometriosis stroma or endometriosis uterina or internal endometriosis or myometrial wall asymmetry or global uterine enlargement or heterogeneous echogenicity or irregular junctional zone or myometrial cysts or fan-shaped shadowing or myometrial lesion* or adenomyosis).mp. |  |
| 7 | 5 or 6 |  |
| 8 | exp ultrasound/ |  |
| 9 | (transvaginal sonography or transvaginal echography or transvaginal ultrasonography or transvaginal ultrasound or vaginal echography or vaginal sonography or vaginal ultrasonography or vaginal ultrasound or three dimensional echography or 3 dimensional echography or 3D echography or 3D sonography or 3D ultrasonogram or 3D ultrasonography or 3D ultrasound or 3D-US or 3DUS or threedimensional sonography or threedimensional ultrasonic evaluation or threedimensional ultrasonography or threedimensional ultrasound or two dimensional echography or 2 dimensional echography or 2D echography or 2D sonography or 2D ultrasonogram or 2D ultrasonography or 2D ultrasound or 2D-US or 2DUS or twodimensional sonography or twodimensional ultrasonic evaluation or twodimensional ultrasonography or twodimensional ultrasound or gynaecological imag* or gynecological imag*).mp. |  |
| 10 | 8 or 9 |  |
| 11 | 4 and 7 and 10 |  |
| 12 | limit 11 to yr="2025 -Current" |  |

### **Cochrane Library**

| **#** | **Query** | **Results** |
| --- | --- | --- |
| #1 | ((infertility OR "fertility disorder" OR infecundity OR "primary infertility" OR "secondary infertility" OR "uterine infertility" OR "uterus factor infertility" OR "uterus infertility" OR "uterus-related infertility" OR "absolute uterine infertility" OR IVF OR "in vitro fertilization" OR "live birth rate" OR "biochemical pregnancy" OR "ongoing pregnancy" OR abortion OR "spontaneous abortion" OR miscarriage OR "pregnancy loss" OR "loss of pregnancy" OR "loss of the pregnancy" OR "miscarriage of pregnancy" OR miscarriages OR "spontaneous pregnancy loss" OR "recurrent pregnancy loss" OR "habitual abortion" OR "recurrent abortion" OR "recurrent fetal loss" OR "recurrent miscarriage" OR "repeated miscarriage" OR "repeated pregnancy loss")):ti,ab,kw |  |
| #2 | MeSH descriptor: [Infertility] explode all trees |  |
| #3 | MeSH descriptor: [Abortion, Spontaneous] explode all trees |  |
| #4 | #1 OR #2 OR #3 |  |
| #5 | MeSH descriptor: [Adenomyosis] explode all trees |  |
| #6 | ((adenomyosis OR adenomyoses OR adenomyotic OR "adenomyosis uteri" OR MUSA OR "endometrial adenoma" OR "endometriosis interna" OR adenomyoma OR "endometriosis stroma" OR "endometriosis uterina" OR "internal endometriosis" OR "myometrial wall asymmetry" OR "global uterine enlargement" OR "heterogeneous echogenicity" OR "irregular junctional zone" OR "myometrial cysts" OR "fan-shaped shadowing" OR "myometrial lesion" OR "myometrial lesions")):ti,ab,kw |  |
| #7 | #5 OR #6 |  |
| #8 | MeSH descriptor: [Ultrasonography] explode all trees |  |
| #9 | ((transvaginal sonography OR transvaginal echography OR transvaginal ultrasonography OR transvaginal ultrasound OR vaginal echography OR vaginal sonography OR vaginal ultrasonography OR vaginal ultrasound OR three dimensional ultrasound OR "3D ultrasound" OR "3-dimensional ultrasound" OR "3D sonography" OR "3D ultrasonography" OR "3D-US" OR 3DUS OR two dimensional ultrasound OR "2D ultrasound" OR "2-dimensional ultrasound" OR "2D sonography" OR "2D ultrasonography" OR "2D-US" OR 2DUS OR "gynaecological imaging" OR "gynecological imaging")):ti,ab,kw |  |
| #10 | #8 OR #9 |  |
| #11 | #4 AND #7 AND #10 |  |

*Publication year filter applied: 2025–2026.*

### **Scopus**

| **Database** | **Search string** |
| --- | --- |
| Scopus | TITLE-ABS-KEY ( infertil* OR "fertility disorder*" OR infecund* OR "primary infertil*" OR "secondary infertil*" OR "uterine infertil*" OR "uterus factor infertil*" OR "uterus infertil*" OR "uterus-related infertil*" OR "absolute uterine infertil*" OR IVF OR "in vitro fertilization" OR "live birth rate*" OR "biochemical pregnanc*" OR "ongoing pregnanc*" OR abortion* OR "spontaneous abortion*" OR miscarriage* OR "pregnancy loss*" OR "loss of pregnancy" OR "loss of the pregnancy" OR "miscarriage of pregnancy" OR "spontaneous pregnanc* loss*" OR "recurrent pregnanc* loss*" OR "habitual abortion*" OR "recurrent abortion*" OR "recurrent fetal loss*" OR "recurrent miscarriage*" OR "repeated miscarriage*" OR "repeated pregnanc* loss*" ) AND TITLE-ABS-KEY ( adenomyos* OR "adenomyosis uteri" OR MUSA OR "endometrial adenoma*" OR "endometriosis interna" OR adenomyoma* OR "endometriosis stroma*" OR "endometriosis uterina" OR "internal endometriosis" OR "myometrial wall asymmetr*" OR "global uterine enlargement*" OR "heterogeneous echogenicit*" OR "irregular junctional zone*" OR "myometrial cyst*" OR "fan-shaped shadowing" OR "myometrial lesion*" OR "myometrial lesions" ) AND TITLE-ABS-KEY ( ultrasound* OR sonograph* OR ultrasonograph* OR "transvaginal sonograph*" OR "transvaginal ultrasound*" OR "transvaginal ultrasonograph*" OR "transvaginal echograph*" OR "vaginal ultrasound*" OR "vaginal sonograph*" OR "vaginal ultrasonograph*" OR "three dimensional ultrasound*" OR "3D ultrasound*" OR "3-dimensional ultrasound*" OR "3D sonograph*" OR "3D ultrasonograph*" OR "3D-US" OR 3DUS OR "two dimensional ultrasound*" OR "2D ultrasound*" OR "2-dimensional ultrasound*" OR "2D sonograph*" OR "2D ultrasonograph*" OR "2D-US" OR 2DUS OR "gynaecological imaging" OR "gynecological imaging" ) |

*Publication year filter applied: 2025–2026.*

### **Web of Science**

| **Database** | **Search string** |
| --- | --- |
| Web of Science | TS=(infertility OR "fertility disorder" OR infecundity OR "primary infertility" OR "secondary infertility" OR "uterine infertility" OR "uterus factor infertility" OR "uterus infertility" OR "uterus-related infertility" OR "absolute uterine infertility" OR IVF OR "in vitro fertilization" OR "live birth rate" OR "biochemical pregnancy" OR "ongoing pregnancy" OR abortion OR "spontaneous abortion" OR miscarriage OR "pregnancy loss" OR "loss of pregnancy" OR "loss of the pregnancy" OR "miscarriage of pregnancy" OR miscarriages OR "spontaneous pregnancy loss" OR "recurrent pregnancy loss" OR "habitual abortion" OR "recurrent abortion" OR "recurrent fetal loss" OR "recurrent miscarriage" OR "repeated miscarriage" OR "repeated pregnancy loss") AND TS=(adenomyosis OR adenomyoses OR adenomyotic OR "adenomyosis uteri" OR MUSA OR "endometrial adenoma" OR "endometriosis interna" OR adenomyoma OR "internal endometriosis" OR "myometrial wall asymmetry" OR "global uterine enlargement" OR "heterogeneous echogenicity" OR "irregular junctional zone" OR "myometrial cysts" OR "fan-shaped shadowing" OR "myometrial lesion" OR "myometrial lesions") AND TS=(ultrasound OR sonography OR ultrasonography OR "transvaginal sonography" OR "transvaginal ultrasound" OR "transvaginal ultrasonography" OR "transvaginal echography" OR "vaginal ultrasound" OR "3D ultrasound" OR "3-dimensional ultrasound" OR "3D sonography" OR "3D ultrasonography" OR "3D-US" OR 3DUS OR "2D ultrasound" OR "2-dimensional ultrasound" OR "2D sonography" OR "2D ultrasonography" OR "2D-US" OR 2DUS OR "gynecological imaging" OR "gynaecological imaging") AND PY=(2025-2026) |

### **Google Scholar**

| **Database** | **Search string** |
| --- | --- |
| Google Scholar | (adenomyosis OR "adenomyosis uteri" OR MUSA) AND (infertility OR miscarriage OR "pregnancy loss" OR IVF OR "in vitro fertilization") AND (ultrasound OR sonography OR "transvaginal ultrasound" OR "gynecological imaging") |

*Results were sorted by date and the first 200 records screened.*

## First Literature Search — 26 June 2025

### **MEDLINE**

| **#** | **Query** | **Results** |
| --- | --- | --- |
| 1 | exp infertility/ | 77,892 |
| 2 | miscarriage.mp. or exp spontaneous abortion/ | 50,510 |
| 3 | (fertility disorder or infecundity or primary infertility or secondary infertility or uterine infertility or uterus factor infertility or uterus infertility or uterus-related infertility or absolute uterine infertility or IVF or in vitro fertilization or live birth rate or biochemical pregnancy or ongoing pregnancy or abortion, spontaneous or loss of a pregnancy or loss of pregnancy or loss of the pregnancy or miscarriage or miscarriage of pregnancy or miscarriages or pregnancy loss or spontaneous pregnancy loss, recurrent pregnancy loss or habitual abort* or recurrent abortion or recurrent fetal loss or recurrent miscarriage or repeated miscarriage or repeated pregnancy loss).mp. | 92,756 |
| 4 | 1 or 2 or 3 | 166,667 |
| 5 | exp adenomyosis/ | 1,580 |
| 6 | (Adenomyosis or Adenomyoses or adenomyotic or adenomyosis uteri or MUSA, endometrial adenoma or endometriosis interna or adenomyoma or endometriosis stroma or endometriosis uterina or internal endometriosis or myometrial wall asymmetry or global uterine enlargement or heterogeneous echogenicity or irregular junctional zone or myometrial cysts or fan-shaped shadowing or myometrial lesion* or adenomyosis).mp. | 5,503 |
| 7 | 5 or 6 | 5,503 |
| 8 | exp Ultrasonography/ | 511,569 |
| 9 | (Transvaginal sonography or transvaginal echography or transvaginal ultrasonography or transvaginal ultrasound or vaginal echography or vaginal sonography or vaginal ultrasonography or vaginal ultrasound or three dimensional echography or 3 dimensional echography or 3D echography or 3D sonography or 3D ultrasonogram or 3D ultrasonography or 3D ultrasound or 3D-US or 3DUS or threedimensional sonography or threedimensional ultrasonic evaluation or threedimensional ultrasonography or threedimensional ultrasound or two dimensional echography or 2 dimensional echography or 2D echography or 2D sonography or 2D ultrasonogram or 2D ultrasonography or 2D ultrasound or 2D-US or 2DUS or twodimensional sonography or twodimensional ultrasonic evaluation or twodimensional ultrasonography or twodimensional ultrasound or gynaecological imag* or gynecological imag*).mp. | 14,091 |
| 10 | 8 or 9 | 516,736 |
| 11 | 4 and 7 and 10 | 105 |

### **Embase**

| **#** | **Query** | **Results** |
| --- | --- | --- |
| 1 | exp infertility/ | 171,805 |
| 2 | miscarriage.mp. or exp spontaneous abortion/ | 83,107 |
| 3 | (fertility disorder or infecundity or primary infertility or secondary infertility or uterine infertility or uterus factor infertility or uterus infertility or uterus-related infertility or absolute uterine infertility or IVF or in vitro fertilization or live birth rate or biochemical pregnancy or ongoing pregnancy or abortion, spontaneous or loss of a pregnancy or loss of pregnancy or loss of the pregnancy or miscarriage or miscarriage of pregnancy or miscarriages or pregnancy loss or spontaneous pregnancy loss, recurrent pregnancy loss or habitual abort* or recurrent abortion or recurrent fetal loss or recurrent miscarriage or repeated miscarriage or repeated pregnancy loss).mp. | 156,039 |
| 4 | 1 or 2 or 3 | 316,556 |
| 5 | exp adenomyosis/ | 8,670 |
| 6 | (Adenomyosis or Adenomyoses or adenomyotic or adenomyosis uteri or MUSA, endometrial adenoma or endometriosis interna or adenomyoma or endometriosis stroma or endometriosis uterina or internal endometriosis or myometrial wall asymmetry or global uterine enlargement or heterogeneous echogenicity or irregular junctional zone or myometrial cysts or fan-shaped shadowing or myometrial lesion* or adenomyosis).mp. | 11,153 |
| 7 | 5 or 6 | 11,354 |
| 8 | exp ultrasound/ | 273,698 |
| 9 | (Transvaginal sonography or transvaginal echography or transvaginal ultrasonography or transvaginal ultrasound or vaginal echography or vaginal sonography or vaginal ultrasonography or vaginal ultrasound or three dimensional echography or 3 dimensional echography or 3D echography or 3D sonography or 3D ultrasonogram or 3D ultrasonography or 3D ultrasound or 3D-US or 3DUS or threedimensional sonography or threedimensional ultrasonic evaluation or threedimensional ultrasonography or threedimensional ultrasound or two dimensional echography or 2 dimensional echography or 2D echography or 2D sonography or 2D ultrasonogram or 2D ultrasonography or 2D ultrasound or 2D-US or 2DUS or twodimensional sonography or twodimensional ultrasonic evaluation or twodimensional ultrasonography or twodimensional ultrasound or gynaecological imag* or gynecological imag*).mp. | 40,710 |
| 10 | 8 or 9 | 310,119 |
| 11 | 4 and 7 and 10 | 614 |

### **Cochrane Library**

| **#** | **Query** | **Results** |
| --- | --- | --- |
| #1 | ((infertility OR "fertility disorder" OR infecundity OR "primary infertility" OR "secondary infertility" OR "uterine infertility" OR "uterus factor infertility" OR "uterus infertility" OR "uterus-related infertility" OR "absolute uterine infertility" OR IVF OR "in vitro fertilization" OR "live birth rate" OR "biochemical pregnancy" OR "ongoing pregnancy" OR abortion OR "spontaneous abortion" OR miscarriage OR "pregnancy loss" OR "loss of pregnancy" OR "loss of the pregnancy" OR "miscarriage of pregnancy" OR miscarriages OR "spontaneous pregnancy loss" OR "recurrent pregnancy loss" OR "habitual abortion" OR "recurrent abortion" OR "recurrent fetal loss" OR "recurrent miscarriage" OR "repeated miscarriage" OR "repeated pregnancy loss")):ti,ab,kw | 26,049 |
| #2 | MeSH descriptor: [Infertility] explode all trees | 4,598 |
| #3 | MeSH descriptor: [Abortion, Spontaneous] explode all trees | 1,345 |
| #4 | #1 OR #2 OR #3 | 26,295 |
| #5 | MeSH descriptor: [Adenomyosis] explode all trees | 78 |
| #6 | ((adenomyosis OR adenomyoses OR adenomyotic OR "adenomyosis uteri" OR MUSA OR "endometrial adenoma" OR "endometriosis interna" OR adenomyoma OR "endometriosis stroma" OR "endometriosis uterina" OR "internal endometriosis" OR "myometrial wall asymmetry" OR "global uterine enlargement" OR "heterogeneous echogenicity" OR "irregular junctional zone" OR "myometrial cysts" OR "fan-shaped shadowing" OR "myometrial lesion" OR "myometrial lesions")):ti,ab,kw | 601 |
| #7 | #5 OR #6 | 601 |
| #8 | MeSH descriptor: [Ultrasonography] explode all trees | 19,433 |
| #9 | ((ultrasound OR sonography OR ultrasonography OR "transvaginal sonography" OR "transvaginal ultrasound" OR "transvaginal ultrasonography" OR "transvaginal echography" OR "vaginal ultrasound" OR "vaginal sonography" OR "vaginal ultrasonography" OR "three dimensional ultrasound" OR "3D ultrasound" OR "3-dimensional ultrasound" OR "3D sonography" OR "3D ultrasonography" OR "3D-US" OR 3DUS OR "two dimensional ultrasound" OR "2D ultrasound" OR "2-dimensional ultrasound" OR "2D sonography" OR "2D ultrasonography" OR "2D-US" OR 2DUS OR "gynaecological imaging" OR "gynecological imaging")):ti,ab,kw | 63,134 |
| #10 | #8 OR #9 | 68,703 |
| #11 | #4 AND #7 AND #10 | 43 |

### **Web of Science**

| **Database** | **Search string** |
| --- | --- |
| Web of Science | ((TS=((infertility OR "fertility disorder" OR infecundity OR "primary infertility" OR "secondary infertility" OR "uterine infertility" OR "uterus factor infertility" OR "uterus infertility" OR "uterus-related infertility" OR "absolute uterine infertility" OR IVF OR "in vitro fertilization" OR "live birth rate" OR "biochemical pregnancy" OR "ongoing pregnancy" OR abortion OR "spontaneous abortion" OR miscarriage OR "pregnancy loss" OR "loss of pregnancy" OR "loss of the pregnancy" OR "miscarriage of pregnancy" OR miscarriages OR "spontaneous pregnancy loss" OR "recurrent pregnancy loss" OR "habitual abortion" OR "recurrent abortion" OR "recurrent fetal loss" OR "recurrent miscarriage" OR "repeated miscarriage" OR "repeated pregnancy loss"))) AND TS=((adenomyosis OR adenomyoses OR adenomyotic OR "adenomyosis uteri" OR MUSA OR "endometrial adenoma" OR "endometriosis interna" OR adenomyoma OR "internal endometriosis" OR "myometrial wall asymmetry" OR "global uterine enlargement" OR "heterogeneous echogenicity" OR "irregular junctional zone" OR "myometrial cysts" OR "fan-shaped shadowing" OR "myometrial lesion" OR "myometrial lesions"))) AND TS=((ultrasound OR sonography OR ultrasonography OR "transvaginal sonography" OR "transvaginal ultrasound" OR "transvaginal ultrasonography" OR "transvaginal echography" OR "vaginal ultrasound" OR "3D ultrasound" OR "3-dimensional ultrasound" OR "3D sonography" OR "3D ultrasonography" OR "3D-US" OR 3DUS OR "2D ultrasound" OR "2-dimensional ultrasound" OR "2D sonography" OR "2D ultrasonography" OR "2D-US" OR 2DUS OR "gynecological imaging" OR "gynaecological imaging")) |

### **Scopus**

| **Database** | **Search string** |
| --- | --- |
| Scopus | TITLE-ABS-KEY ( infertil* OR "fertility disorder*" OR infecund* OR "primary infertil*" OR "secondary infertil*" OR "uterine infertil*" OR "uterus factor infertil*" OR "uterus infertil*" OR "uterus-related infertil*" OR "absolute uterine infertil*" OR IVF OR "in vitro fertilization" OR "live birth rate*" OR "biochemical pregnanc*" OR "ongoing pregnanc*" OR abortion* OR "spontaneous abortion*" OR miscarriage* OR "pregnancy loss*" OR "loss of pregnancy" OR "loss of the pregnancy" OR "miscarriage of pregnancy" OR "spontaneous pregnanc* loss*" OR "recurrent pregnanc* loss*" OR "habitual abortion*" OR "recurrent abortion*" OR "recurrent fetal loss*" OR "recurrent miscarriage*" OR "repeated miscarriage*" OR "repeated pregnanc* loss*" ) AND TITLE-ABS-KEY ( adenomyos* OR "adenomyosis uteri" OR MUSA OR "endometrial adenoma*" OR "endometriosis interna" OR adenomyoma* OR "endometriosis stroma*" OR "endometriosis uterina" OR "internal endometriosis" OR "myometrial wall asymmetr*" OR "global uterine enlargement*" OR "heterogeneous echogenicit*" OR "irregular junctional zone*" OR "myometrial cyst*" OR "fan-shaped shadowing" OR "myometrial lesion*" OR "myometrial lesions" ) AND TITLE-ABS-KEY ( ultrasound* OR sonograph* OR ultrasonograph* OR "transvaginal sonograph*" OR "transvaginal ultrasound*" OR "transvaginal ultrasonograph*" OR "transvaginal echograph*" OR "vaginal ultrasound*" OR "vaginal sonograph*" OR "vaginal ultrasonograph*" OR "three dimensional ultrasound*" OR "3D ultrasound*" OR "3-dimensional ultrasound*" OR "3D sonograph*" OR "3D ultrasonograph*" OR "3D-US" OR 3DUS OR "two dimensional ultrasound*" OR "2D ultrasound*" OR "2-dimensional ultrasound*" OR "2D sonograph*" OR "2D ultrasonograph*" OR "2D-US" OR 2DUS OR "gynaecological imaging" OR "gynecological imaging" ) |

### **Google Scholar**

| **Database** | **Search string** |
| --- | --- |
| Google Scholar | (adenomyosis OR "adenomyosis uteri" OR MUSA) AND (infertility OR miscarriage OR "pregnancy loss" OR IVF OR "in vitro fertilization") AND (ultrasound OR sonography OR "transvaginal ultrasound" OR "gynecological imaging") |

| Supplementary Table B. Extracted crude prevalences and odds ratios | | | | | | | | |
| --- | --- | --- | --- | --- | --- | --- | --- | --- |
| **Author (year) study design** | **N-adenomyosis/N-total (%)** | **Comparators** | **Outcome** | **Prevalence** | **P-value** | **Effect Estimate (95% CI)** | **P-value** | **Notes** |
| Li et al. (2021)  Single-center retrospective cohort study | 158/158 (100) | A: Uterine volume ≤98.81 cm³  B: Uterine volume >98.81 cm³ | Implantation rate | 43.3% (A) vs 40% (B) | 0.66 | N/A | N/A | aOR is adjusted for age, BMI, duration of infertility, primary infertility, type of AM, endometrial thickness and number of embryos transferred. |
|  |  |  | Clinical pregnancy rate | 51.8% (A) vs 52.0% (B) | 1 | aOR 1.149 (0.577–2.286) | 0.693 |  |
|  |  |  | Pregnancy loss rate | **16.3% (A) vs 51.3% (B)** | **0.001** | **aOR 8.509 (2.290–2.575)** | **0.001** |  |
|  |  |  | Live birth rate | **43.4% (A) vs 25.3% (B)** | **0.02** | **aOR 0.445 (0.206–0.965)** | **0.04** |  |
| Sharma et al. (2025)  Single-center prospective cohort study | 368/585 (62.9) | A: Diffuse AM of outer myometrium  B: Diffuse AM of junctional zone | Chemical pregnancy rate | 47.9% (A) vs 26.4% (B) | N/A | **OR: 0.39 (0.25–0.60)** | **<0.0001** | Not adjusted. |
|  |  |  | Clinical pregnancy rate | 42.5% (A) vs 23.4% (B) | N/A | **OR: 0.41 (0.26–0.65)** | **0.0001** |  |
|  |  |  | Pregnancy loss rate | 21.1% (A) vs 23.4% (B) | N/A | OR: 1.14 (0.47–2.76) | NS |  |
|  |  |  | Live birth rate | 25.8% (A) vs 16.4% (B) | N/A | **OR: 0.57 (0.34–0.94)** | **0.029** |  |
| Zhang et al. (2023)  Single-center retrospective cohort study | 1155/1155 (100) | A: Uterine volume ≤130 cm³  B: Uterine volume >130 cm³ | Clinical pregnancy rate | 40.8% (A) vs 35.2% (B) | 0.152 | aOR: 1.002 (0.798 - 1.257) | 0.989 | aOR is adjusted for age, BMI, embryos/blastocysts, and COH protocol/GnRHa pretreatment. |
|  |  |  | Pregnancy loss rate | **23.6% (A) vs 36.2% (B)** | **0.031** | **aOR: 1.862 (1.278-2.711)** | **0.001** |  |
|  |  |  | Live birth rate | **31.2% (A) vs 22.4% (B)** | **0.017** | **aOR: 0.794 (0.634-0.995)** | **0.033** |  |
| Sharma et al. (2019)  Single-center retrospective cohort study | 152/973 (15.6) | A: 3 MUSA features+endometriosis  B: 4 MUSA features+endometriosis  C: 5 MUSA features+endometriosus | Clinical pregnancy rate | 9.1% (A) vs 6.8% (B) vs 6.8% (C) | NS | N/A | N/A | Baseline characteristics for these subgroups were not reported. |
|  |  |  | Pregnancy loss rate | 5% (A) vs 15% (B) vs 15% (C) | NS | N/A | N/A |  |
|  |  |  | Live birth rate | 4.5% vs 3.4% vs 3.4% | NS | N/A | N/A |  |
|  |  | A: 3 MUSA features  B: 4 MUSA features  C: 5 MUSA features | Clinical pregnancy rate | 7.8% (A) vs 9.4% (B) vs 6.3% (C) | NS | N/A | N/A |  |
|  |  |  | Pregnancy loss rate | 0% (A) vs 13.3% (B) vs 26.7% (C) | NS | N/A | N/A |  |
|  |  |  | Live birth rate | 6.3% (A) vs 3.1% (B) vs 3.1% (C) | NS | N/A | N/A |  |
| Benaglia et al. (2014)  Single-center prospective cohort study | 49/98 (50) | A: Focal AM  B: Diffuse AM | Implantation rate | 32% (A) vs 32% (B) | NS | N/A | N/A | Baseline characteristics for these subgroups were not reported. |
|  |  |  | Clinical pregnancy rate | 40% (A) vs 46% (B) | NS | N/A | N/A |  |
|  |  |  | Live birth rate | 36% (A) vs 33% (B) | NS | N/A | N/A |  |
|  |  | A: Uterine volume above median (61ml)  B: Uterine volume equal to/below median | Implantation rate | 31% (A) vs 33% (B) | NS | N/A | N/A |  |
|  |  |  | Clinical pregnancy rate | 42% (A) vs 44% (B) | NS | N/A | N/A |  |
|  |  |  | Live birth rate | 33% (A) vs 36% (B) | NS | N/A | N/A |  |
| Park et al. (2016)  Single-center retrospective cohort study | 241/241 (100) | A: Focal AM  B: Diffuse AM | Clinical pregnancy rate | 32.9% (A) vs 25.7% (B) | NS | N/A | N/A | Baseline characteristics for these subgroups were not reported. |
|  |  |  | Pregnancy loss rate | 7.1% (A) vs 14.3% (B) | NS | N/A | N/A |  |
| Mavrelos et al. (2017)  Multi-center prospective cohort study | 72/375 (19.2) | No-AM vs Endometrial striae | Clinical pregnancy rate | N/A | N/A | aOR 3.80 (0.84–13.60) | N/A | aOR is adjusted for age, BMI, AMH, FSH, and AFC |
|  |  | No-AM vs Myometrial cysts | Clinical pregnancy rate | N/A | N/A | OR 0.81 (0.27–2.40) | N/A |  |
|  |  | No-AM vs Asymmetric thickening | Clinical pregnancy rate | N/A | N/A | OR 0.63 (0.22–1.80) | N/A |  |
|  |  | No-AM vs Parallel shadowing | Clinical pregnancy rate | N/A | N/A | OR 0.63 (0.13–3.10) | N/A |  |
|  |  | No-AM vs Adenomyoma | Clinical pregnancy rate | N/A | N/A | OR 2.70 (0.29–24.50) | N/A |  |
|  |  | No-AM vs Irregular junctional zone | Clinical pregnancy rate | N/A | N/A | OR 0.13 (0.02–1.00) | N/A |  |
|  |  | No-AM vs Endometrial islands | Clinical pregnancy rate | N/A | N/A | OR 1.90 (0.37–10.60) | N/A |  |
|  |  | No-AM vs 1 feature | Clinical pregnancy rate | N/A | N/A | OR 0.85 (0.23–3.20) | N/A |  |
|  |  | No-AM vs 2 features | Clinical pregnancy rate | N/A | N/A | OR 0.95 (0.31–2.80) | N/A |  |
|  |  | No-AM vs 3 features | Clinical pregnancy rate | N/A | N/A | OR 1.00 (0.23–4.60) | N/A |  |
|  |  | No-AM vs 4 features | Clinical pregnancy rate | N/A | N/A | OR 0.22 (0.26–1.80) | N/A |  |
|  |  | No-AM vs 5 features | Clinical pregnancy rate | N/A | N/A | OR 0.39 (0.12–1.30) | N/A |  |
|  |  | No-AM vs 6 features | Clinical pregnancy rate | N/A | N/A | OR 0.30 (0.07–1.60) | N/A |  |
|  |  | No-AM vs 7 features | Clinical pregnancy rate | N/A | N/A | OR 0.24 (0.09–6.40) | N/A |  |
| Higgins et al. (2021)  Multi-center prospective cohort study | 301/944 (31.9) | A: No-AM  B: Severe AM (defined as ≥ 3 MUSA) | Clinical pregnancy rate | 8.3% (A) vs 17.3% (B) | N/A | **OR 0.7 (0.5-0.9)**  **aOR 0.5 (0.3-1.0)** | **0.01**  **0.052** | aOR is adjusted for age, smoking status, treatment cycle, aetiology status [endometriosis, ovulation defect, polycystic ovaries, polycystic ovary syndrome, BMI, parity status. |
|  |  |  | Live birth rate | 6.8% (A) vs 8.9% (B) | N/A | OR 0.9 (0.6-1.3)  aOR 0.9 (0.4-2.0) | 0.44  0.84 |  |
| Neal et al. (2020)  Single-center prospective cohort study | 99/652 (15.3) | A: No-AM  B: Global uterine enlargement | Clinical pregnancy rate | N/A | N/A | aOR 1.3 (0.7–1.4) | N/A | Adjusted for age at transfer, BMI, previous failed transfer, endometrial preparation protocol and blastocyst quality |
|  |  |  | Pregnancy loss rate | N/A | N/A | aOR 0* (0–5.4) | N/A |  |
|  |  |  | Live birth rate | N/A | N/A | aOR 1.5 (0.8–1.6) | N/A |  |
|  |  | A: No-AM  B: Myometrial wall asymmetry | Clinical pregnancy rate | N/A | N/A | aOR 0.8 (0.4–1.1) | N/A |  |
|  |  |  | Pregnancy loss rate | N/A | N/A | aOR 1.0 (0.2–4.5) | N/A |  |
|  |  |  | Live birth rate | N/A | N/A | aOR 0.7 (0.4–1.1) | N/A |  |
|  |  | A: No-AM  B: Heterogeneous echogenicity | Clinical pregnancy rate | N/A | N/A | aOR 1.1 (0.7–1.3) | N/A |  |
|  |  |  | Pregnancy loss rate | N/A | N/A | aOR 2.1 (0.6–5.6) | N/A |  |
|  |  |  | Live birth rate | N/A | N/A | aOR 1.0 (0.6–1.3) | N/A |  |
|  |  | A: No-AM  B: Irregular junctional zone | Clinical pregnancy rate | N/A | N/A | aOR 1.3 (0.7–1.4) | N/A |  |
|  |  |  | Pregnancy loss rate | N/A | N/A | aOR 0* (0–6.2) | N/A |  |
|  |  |  | Live birth rate | N/A | N/A | aOR 1.5 (0.8–1.6) | N/A |  |
|  |  | A: No-AM  B: Myometrial cysts | Clinical pregnancy rate | N/A | N/A | aOR 1.1 (0.9–1.3) | N/A |  |
|  |  |  | Pregnancy loss rate | N/A | N/A | aOR 1.7 (0.6–4.4) | N/A |  |
|  |  |  | Live birth rate | N/A | N/A | aOR 1.1 (0.8–1.3) | N/A |  |
|  |  | A: No-AM  B: Fan-shaped shadowing | Clinical pregnancy rate | N/A | N/A | aOR 1.3 (0.5–1.4) | N/A |  |
|  |  |  | Pregnancy loss rate | N/A | N/A | aOR 0* (0–8.4) | N/A |  |
|  |  |  | Live birth rate | N/A | N/A | aOR 1.5 (0.5–1.6) | N/A |  |
|  |  | A: No-AM  B: Ill-defined myometrial lesion | Clinical pregnancy rate | N/A | N/A | aOR 0.9 (0.3–1.2) | N/A |  |
|  |  |  | Pregnancy loss rate | N/A | N/A | aOR 0* (0–7.1) | N/A |  |
|  |  |  | Live birth rate | N/A | N/A | aOR 1.0 (0.3–1.4) | N/A |  |
|  |  | A: No-AM  B: 1 MUSA feature  C: 2 or more MUSA features | Clinical pregnancy rate | 75% (A) vs 80% (B) vs 65% (C) | 0.35 | N/A | N/A | Baseline characteristics for these subgroups were not reported. |
|  |  |  | Pregnancy loss rate | 10% (A) vs 12% (B) vs 5% (C) | 0.49 | N/A | N/A |  |
|  |  |  | Live birth rate | 65% (A) vs 70% (B) vs 65% (C) | 0.76 | N/A | N/A |  |
| Trinchant et al. (2022)  Multi-center retrospective cohort study | 179/3307 (5.4) | A: Focal AM  B: Diffuse AM | Chemical pregnancy rate | 50% (A) vs 50% (B) | 0.9 | OR 0.99 (0.38–2.57) | 0.976 | Not adjusted. |
|  |  |  | Implantation rate | 41% (A) vs 46% (B) | 0.82 | OR 0.82 (0.31–2.14) | 0.661 |  |
|  |  |  | Pregnancy loss rate | 30% (A) vs 17% (B) | 0.556 | OR 2.12 (0.31–11.10) | 0.556 |  |
|  |  |  | Live birth rate | 29% (A) vs 39% (B) | 0.37 | OR 0.63 (0.21–1.73) | 0.369 |  |
| Han et al. (2023)  Single-center retrospective cohort study | 1146/1665 (68.8) | A: Diffuse AM  B: Focal AM  C: No-AM with tubal infertility | Clinical pregnancy rate | **32.5% (A) vs 41.5% (B) vs 47.4% (C)** | **<0.001** | **A vs B: aOR 0.708 (0.539 to 0.931)**  **A vs C: aOR 0.663 (0.489 to 0.899)** | **0.013**  **0.008** | aOR is adjusted for age, FSH level, BMI, AFC, AMH, the number of transferred embryos, uterine volume and endometrial thickness on transfer  day. |
|  |  |  | Pregnancy loss rate | **41.7% (A) vs 27.2% (B) vs 15.4% (C)** | **<0,001** | **A vs B: aOR 1.875 (1.177 to 2.988)**  **A vs C: aOR 3.422 (1.962 to 5.969)**  **B vs C: aOR 1.825 (1.112 to 2.995)** | **0.008**  **0.001**  **0.017** |  |
|  |  |  | Live birth rate | **18.7% (A) vs 30.2% (B) vs 40.1% (C)** | **<0.001** | **A vs B: aOR 0.530 (0.385 to 0.730)**  **A vs C: aOR 0.441 (0.313 to 0.623)** | **0.001**  **0.001** |  |
| Cozzolino et al. (2024)  Single-center prospective cohort study | 114/228 (50) | A: No-AM  B: Diffuse AM | Chemical pregnancy rate | B: 52.3% | N/A | RR 1.02 (0.67-1.55) | 1 | Not adjusted. |
|  |  |  | Pregnancy loss rate | B: 56.6% | N/A | RR 1.19 (0.61-2.31) | 0.796 |  |
|  |  | A: No-AM  B: Focal AM | Chemical pregnancy rate | B: 38.5% | N/A | RR 0.91 (0.58-1.43) | 0.7 |  |
|  |  |  | Pregnancy loss rate | B: 34.7% | N/A | RR 0.85 (0.42-1.71) | 0.791 |  |
|  |  | A: No-AM  B: Diffuse+focal AM | Chemical pregnancy rate | B: 9.2% | N/A | RR 1.19 (0.64-2.18) | 0.76 |  |
|  |  |  | Pregnancy loss rate | B: 13.0% | N/A | RR 1.48 (0.61-3.54) | 0.657 |  |
|  |  | A: No-AM  B: Junctional zone only AM | Chemical pregnancy rate | B: 26.2% | N/A | RR 1.19 (0.64-2.18) | 0.76 |  |
|  |  |  | Pregnancy loss rate | B: 34.8% | N/A | RR 1.51 (0.78-2.90) | 0.255 |  |
|  |  | A: No-AM  B: Outer myometrium only AM | Chemical pregnancy rate | B: 47.7% | N/A | RR 0.69 (0.43-1.10) | 0.126 |  |
|  |  |  | Pregnancy loss rate | B: 21.7% | N/A | **RR 0.30 (0.13-0.72)** | **0.004** |  |
|  |  | A: No-AM  B: J + outer myometrium AM | Chemical pregnancy rate | B: 26.2% | N/A | **RR 1.57 (1.04-2.36)** | **0.047** |  |
|  |  |  | Pregnancy loss rate | B: 58.8% | N/A | **RR 2.17 (1.18-4.00)** | **0.037** |  |
|  |  | A: No-AM  B: Outer myometrium total AM | Chemical pregnancy rate | B: 73.8% | N/A | RR 1.12 (0.67-1.89) | 0.668 |  |
|  |  |  | Pregnancy loss rate | B: 65.2% | N/A | RR 0.66 (0.34-1.28) | 0.255 |  |
|  |  | A: No-AM  B: Junctional zone total AM | Chemical pregnancy rate | B: 52.3% | N/A | RR 1.45 (0.91-2.31) | 0.126 |  |
|  |  |  | Pregnancy loss rate | B: 78.3% | N/A | **RR 3.28 (1.38-7.78)** | **0.004** |  |
|  |  | A: No-AM  B: Diffuse  junctional zone AM | Chemical pregnancy rate | B: 32.3% | N/A | RR 1.17 (0.76-1.80) | 0.553 |  |
|  |  |  | Pregnancy loss rate | B: 52.1% | N/A | **RR 2.29 (1.22-4.30)** | **0.015** |  |
|  |  | A: No-AM  B: Focal Junctional zone AM | Chemical pregnancy rate | B: 23.1% | N/A | RR 1.17 (0.74-1.85) | 0.522 |  |
|  |  |  | Pregnancy loss rate | B: 30.4% | N/A | RR 1.46 (0.74-2.86) | 0.361 |  |
|  |  | A: No-AM  B: Diffuse outer myometrium AM | Chemical pregnancy rate | B: 54.2% | N/A | RR 1.14 (0.75-1.75) | 0.547 |  |
|  |  |  | Pregnancy loss rate | B: 56.5% | N/A | RR 1.34 (0.69-2.61) | 0.443 |  |
|  |  | A: No-AM  B: Focal outer myometrium AM | Chemical pregnancy rate | B: 49.2% | N/A | RR 0.85 (0.50-1.43) | 0.665 |  |
|  |  |  | Pregnancy loss rate | B: 13.0% | N/A | RR 0.39 (0.13-1.16) | 0.081 |  |
|  |  | A: No-AM  B: Adenomyoma | Chemical pregnancy rate | B: 3.1% | N/A | RR 0.56 (0.48-0.66) | 0.604 |  |
|  |  |  | Pregnancy loss rate | B: 4.3% | N/A | RR 1.43 (0.34-5.96) | 1 |  |
|  |  | A: No-AM  B: Mild AM** | Chemical pregnancy rate | B: 66.2% | N/A | RR 0.85 (0.55-1.29) | 0.557 |  |
|  |  |  | Pregnancy loss rate | B: 56.5% | N/A | RR 0.67 (0.35-1.27) | 0.277 |  |
|  |  | A: No-AM  B: Moderate AM** | Chemical pregnancy rate | B: 20.0% | N/A | RR 1.49 (0.98-2.25) | 0.089 |  |
|  |  |  | Pregnancy loss rate | B: 17.4% | N/A | RR 0.84 (0.35-2.05 | 0.758 |  |
|  |  | A: No-AM  B: Severe AM** | Chemical pregnancy rate | B: 13.8% | N/A | RR 0.55 (0.20-1.51) | 0.228 |  |
|  |  |  | Pregnancy loss rate | B: 26.1% | N/A | **RR 2.20 (1.19-4.04)** | **0.005** |  |
| Dason et al. (2023)  Single-center retrospective cohort study | 76/100 (76) | A: No-AM  B: Interrupted junctional zone in the fundal uterus | Chemical pregnancy rate | N/A | N/A | **aOR 0.41 (0.18-0.93)** | **0.03** | aOR adjusted for age, BMI, total motile sperm, donor age, endometriosis, endometrial thickness, and blastocyst vs. morula. |
|  |  |  | Clinical pregnancy rate | N/A | N/A | **aOR 0.42 (0.18-0.95)** | **0.04** |  |
|  |  |  | Pregnancy loss rate | N/A | N/A | aOR 0.41 (0.14-1.23) | 0.11 |  |
|  |  |  | Live birth rate | N/A | N/A | aOR 0.62 (0.26-1.467) | 0.28 |  |
|  |  | A: No-AM  B: Interrupted Junctional zone in the posterior uterus | Chemical pregnancy rate | N/A | N/A | **aOR 0.40 (0.19-0.85)** | **0.02** |  |
|  |  |  | Clinical pregnancy rate | N/A | N/A | **aOR 0.40 (0.18-0.88)** | **0.02** |  |
|  |  |  | Pregnancy loss rate | N/A | N/A | aOR 0.42 (0.13-1.33) | 0.14 |  |
|  |  |  | Live birth rate | N/A | N/A | aOR 0.56 (0.23-1.37) | 0.2 |  |
|  |  | A: No-AM  B: One MUSA-feature | Chemical pregnancy rate | 66.0% (A) vs 60.0% (B) | 0.156 | OR 0.65 (0.25–1.71)  aOR 0.65 (0.17–2.53) | 0.39  0.53 |  |
|  |  |  | Clinical pregnancy rate | 50.9 (A) vs 40.0% (B) | 0.435 | OR 0.57 (0.16–2.03)  aOR 0.61 (0.16–2.29) | 0.39  0.46 |  |
|  |  |  | Pregnancy loss rate | N/A | N/A | OR 0.96 (0.22–4.21)  aOR 0.72 (0.10–5.31) | 0.95  0.75 |  |
|  |  |  | Live birth rate | 36.5% (A) vs 26.7% (B) | 0.553 | OR 0.65 (0.14–3.08)  aOR 0.67 (0.10–4.45) | 0.58  0.68 |  |
|  |  | A: No-AM  B: Two or more MUSA-features | Chemical pregnancy | 66.0% (A) vs 51.0 (B) | 0.156 | OR 0.56 (0.27–1.15)  aOR 0.50 (0.20–1.24) | 0.11  0.14 |  |
|  |  |  | Clinical pregnancy rate | 50.9% (A) vs 40.9% (B) | 0.435 | OR 0.72 (0.34–1.5)  aOR 0.84 (0.34–2.06) | 0.38  0.7 |  |
|  |  |  | Pregnancy loss rate | N/A | N/A | OR 0.87 (0.32–2.40)  aOR 0.51 (0.134–1.88) | 0.8  0.31 |  |
|  |  |  | Live birth rate | 36.5% (A) vs 28.9% (B) | 0.553 | OR 0.79 (0.389–1.61)  aOR 1.12 (0.46–2.73) | 0.52  0.81 |  |
| Trinchant et al. (2025)  Multi-center retrospective cohort study | 228/456 (50) | A: Focal  B: Diffuse | Chemical pregnancy rate | 49% (A) vs 59% (B) | N/A | OR 0.626 (0.33–1.17) | 0.179 | aOR is adjusted for age, BMI, smoking, presence of endometriosis, presence of fibroids, previous deliveries and cesarean sections. |
|  |  |  | Implantation rate | 37% (A) vs 49% (B) | N/A | OR 0.627 (0.33–1.18)  aOR 0.41 (0.17–0.94) | 0.178  0.045 |  |
|  |  |  | Pregnancy loss rate | 61% (A) vs 41% (B) | N/A | OR 2.24 (0.79–6.63) | 0.153 |  |
|  |  |  | Live birth rate | 15% (A) vs 28% (B) | N/A | **OR 0.422 (0.17–0.95)**  **aOR 0.298 (0.10–0.87)** | **0.03**  **0.034** |  |
|  |  | A: Focal  B: No-AM | Implantation rate | N/A | N/A | **aOR 2.84 (1.53–5.28)** | **0.002** |  |
|  |  |  | Live birth rate | N/A | N/A | **aOR 5.98 (2.69–13.27)** | **0.001** |  |
|  |  | A: Diffuse  B: No-AM | Implantation rate | N/A | N/A | **aOR 1.66 (1.08–2.54)** | **0.013** |  |
|  |  |  | Live birth rate | N/A | N/A | **aOR 2.33 (1.49–3.66)** | **<0.001** |  |
| Alson et al. (2024)  Single-center prospective cohort study | 290/1037 (28) | A: No-AM  B: Focal | Live birth rate | N/A | N/A | aRR 1.37 (0.68–2.75) | 0.43 | aRR is adjusted for age, s-AMH, BMI, AFC, protocol, FSH dose, stimulation days, ET day, endometriosis. |
|  |  | A: No-AM  B: Diffuse | Live birth rate | N/A | N/A | aRR 0.68 (0.36–1.3 | 0.244 |  |
|  |  | A: No direct feature of AM  B: At least one direct feature of AM | Fertilization rate | 42% (A) vs 39% (B) | 0.722 | N/A | N/A | aRR is adjusted for age. |
|  |  |  | Cumulative pregnancy rate | 49.9% (A) vs 36.3% (B) | N/A | **RR 0.73 (0.56–0.95)**  **aRR 0.99 (0.97–1.00)** | **0.019**  **0.066** |  |
|  |  |  | Pregnancy loss rate | 10.2 (A) vs 14.7% (B) | N/A | RR 1.46 (0.88–2.42)  aRR 1.34 (0.81–2.2) | 0.14  0.26 |  |
|  |  |  | Live birth rate | 9.7 (A) vs 11.8% (B) | N/A | RR 0.82 (0.47–1.44)  aRR 1.07 (0.33–3.49) | 0.49  0.92 |  |
|  |  | A: No direct or indirect  feature of AM  B: Indirect features  without direct features  of AM | Cumulative pregnancy rate | 54.1 (A) vs 33.5% (B) | N/A | **RR 0.63 (0.51–0.78)**  **aRR 0.64 (0.52–0.79)** | **<0.001**  **<0.001** |  |
|  |  |  | Pregnancy loss rate | 10.6 (A) vs 8.5% (B) | N/A | RR 0.81 (0.49–1.35)  aRR 0.79 (0.48–1.31) | 0.420  0.361 |  |
|  |  |  | Live birth rate | 46.7 (A) vs 26.6 (B) | N/A | **RR 0.57 (0.45–0.73)**  **aRR 0.58 (0.45–0.75)** | **<0.001**  **<0.001** |  |
|  |  | A: No-AM  B: At least one feature of AM located in the junctional zone | Live birth rate | N/A | N/A | **aRR 0.29 (0.11–0.74)** | **0.01** | aRR is adjusted for age, s-AMH, BMI, AFC, protocol, FSH dose, stimulation days, ET day, endometriosis. |
|  |  | A: No-AM  B: AM in the IM | Live birth rate | N/A | N/A | **RR 0.39 (0.11-0.74)**  **aRR 0.29 (0.11-0.74)** | **0.01**  **0.01** |  |
|  |  | A: No-AM  B: AM in the OM | Live birth rate | N/A | N/A | **RR 2.61 (1.42-4.80)**  **aRR 2.61 (1.42-4.80)** | **0.002**  **0.002** |  |
|  |  | A: No-AM  B: Interrupted junctional zone AM | Live birth rate | N/A | N/A | **RR 0.36 (0.23–0.58)**  **aRR 0.34 (0.28–0.51)** | **<0.001**  **<0.001** |  |
|  |  | A: No-AM  B: Mild disease (extent <25%) | Live birth rate | N/A | N/A | **RR 1.0 (0.15–0.93)** | **0.03** | Not adjusted. |
|  |  | A: No-AM  B: Moderate disease  (extent 25%–50%) | Live birth rate | N/A | N/A | RR 1.04 (0.55–1.98) | 0.62 |  |
|  |  | A: No-AM  B: Severe disease (extent >50%) | Live birth rate | N/A | N/A | RR 0.89 (0.27–2.98) | 0.85 |  |
|  |  | A: No-AM  B: 1 feature of AM | Live birth rate | N/A | N/A | **RR 0.47 (0.23-0.98)**  **aRR 0.45 (0.21-0.95)** | **0.043**  **0.037** | aRR is adjusted for age. |
|  |  | A: No-AM  B: 2 features of AM | Live birth rate | N/A | N/A | **RR 0.35 (0.15-0.83)**  **aRR 0.33 (0.14-0.79)** | **0.017**  **0.013** |  |
|  |  | A: No-AM  B: 3 features of AM | Live birth rate | N/A | N/A | **RR 0.13 (0.03-0.55)**  **aRR 0.12 (0.03-0.55)** | **0.006**  **0.006** |  |
| Benlioglu et al. (2026)  Single-center prospective cohort study | 76/314 (24) | A: No-AM  B: Direct features of AM | Live birth rate | 40.3% (A) vs 22.2% (B) | N/A | **OR 0.42 (0.20-0.82)**  **aOR 0.42 (0.20-0.84)** | **0.015**  **0.017** | aOR = adjusted for age, BMI, and embryo transfer stage |
|  |  |  | Pregnancy loss rate | 22.6% (A) vs 48.0% (B) | N/A | **OR 3.17 (1.30-7.72)**  **aOR 3.77 (1.44-10.21)** | **0.011**  **0.007** |  |
| Etrusco et al. (2026)  Single-center retrospective cohort study | 120/240 (50) | A: No-AM  B: JZ-AM | Implantation rate | 75.0% (A) vs 68.3% (B) | 0.25 | NR | NR | aOR = adjusted for age, endometrial preparation protocol, endometrial thickness, progesterone on ET day, and type of ET. |
|  |  |  | Biochemical pregnancy loss rate | 15.8% (A) vs 11.7% (B) | 0.35 | NR | NR |  |
|  |  |  | Live birth rate | **50.8% (A) vs 34.2% (B)** | **0.009** | **aOR 0.43 (0.24–0.76)** | **0.004** |  |
|  |  |  | Pregnancy loss rate | **11.1% (A) vs 32.9% (B)** | **<0.001** | **aOR 3.33 (1.42–7.82)** | **0.005** |  |
|  |  | A: Mild JZ-AM (<25%)  B: No-AM | Ongoing pregnancy rate | NR | NR | RR 0.85 (0.40–1.78) | 0.62 | Not adjusted. |
|  |  |  | Pregnancy loss rate | NR | NR | RR 1.32 (0.43–4.05) | 0.58 |  |
|  |  | A: Moderate JZ-AM (25–50%)  B: No-AM | Ongoing pregnancy rate | NR | NR | RR 0.65 (0.33–1.27) | 0.21 |  |
|  |  |  | Pregnancy loss rate | NR | NR | **RR 2.41 (1.05–5.55)** | **0.04** |  |
|  |  | A: Severe JZ-AM (>50%)  B: No-AM | Ongoing pregnancy rate | NR | NR | **RR 0.17 (0.04–0.67)** | **0.007** |  |
|  |  |  | Pregnancy loss rate | NR | NR | **RR 4.05 (1.78–9.12)** | **0.001** |  |
| Han et al. (2026)  Single-center retrospective cohort study | 300/600 (50) | A: No direct features of AM  B: Direct features of AM | Clinical pregnancy rate | NR | NR | OR 1.20 (0.75–1.93) | NR |  |
|  |  |  | Ongoing pregnancy rate | NR | NR | OR 1.50 (0.95–2.37) | NR |  |
|  |  |  | Pregnancy loss rate | NR | NR | OR 0.53 (0.26–1.08) | NR |  |
|  |  |  | Biochemical pregnancy loss rate | NR | NR | OR 0.48 (0.22–1.04) | NR |  |
|  |  | A: No indirect features of AM  B: Indirect features of AM | Clinical pregnancy rate | NR | NR | OR 0.59 (0.26–1.31) | NR |  |
|  |  |  | Ongoing pregnancy rate | NR | NR | OR 0.50 (0.24–1.05) | NR |  |
|  |  |  | Pregnancy loss rate | NR | NR | OR 2.19 (0.62–7.73) | NR |  |
|  |  |  | Biochemical pregnancy loss rate | NR | NR | OR 2.19 (0.50–9.57) | NR |  |
|  |  | A: No myometrial cyst  B: Myometrial cyst | Clinical pregnancy rate | NR | NR | OR 1.33 (0.80–2.20) | NR |  |
|  |  |  | Ongoing pregnancy rate | NR | NR | OR 1.33 (0.80–2.20) | NR |  |
|  |  |  | Pregnancy loss rate | NR | NR | OR 0.72 (0.33–1.60) | NR |  |
|  |  |  | Biochemical pregnancy loss rate | NR | NR | OR 0.64 (0.27–1.52) | NR |  |
|  |  | A: No hyperechoic island  B: Hyperechoic island | Clinical pregnancy rate | NR | NR | OR 0.87 (0.47–1.63) | NR |  |
|  |  |  | Ongoing pregnancy rate | NR | NR | OR 0.87 (0.47–1.63) | NR |  |
|  |  |  | Pregnancy loss rate | NR | NR | OR 0.62 (0.20–1.93) | NR |  |
|  |  |  | Biochemical pregnancy loss rate | NR | NR | OR 0.69 (0.23–2.06) | NR |  |
|  |  | A: No subendometrial buds and lines  B: Subendometrial buds and lines | Clinical pregnancy rate | NR | NR | OR 1.67 (0.72–3.89) | NR |  |
|  |  |  | Ongoing pregnancy rate | NR | NR | OR 1.59 (0.74–3.43) | NR |  |
|  |  |  | Pregnancy loss rate | NR | NR | OR 0.79 (0.25–2.47) | NR |  |
|  |  |  | Biochemical pregnancy loss rate | NR | NR | OR 0.25 (0.03–1.88) | NR |  |
|  |  | A: No asymmetrical thickening  B: Asymmetrical thickening | Clinical pregnancy rate | NR | NR | OR 0.88 (0.52–1.48) | NR |  |
|  |  |  | Ongoing pregnancy rate | NR | NR | OR 0.86 (0.52–1.43) | NR |  |
|  |  |  | Pregnancy loss rate | NR | NR | OR 1.13 (0.53–2.42) | NR |  |
|  |  |  | Biochemical pregnancy loss rate | NR | NR | OR 1.24 (0.58–2.67) | NR |  |
|  |  | A: No fan-shaped shadowing  B: Fan-shaped shadowing | Clinical pregnancy rate | NR | NR | OR 0.90 (0.56–1.46) | NR |  |
|  |  |  | Ongoing pregnancy rate | NR | NR | OR 0.83 (0.52–1.31) | NR |  |
|  |  |  | Pregnancy loss rate | NR | NR | OR 1.31 (0.64–2.66) | NR |  |
|  |  |  | Biochemical pregnancy loss rate | NR | NR | OR 0.85 (0.42–1.75) | NR |  |
|  |  | A: No globular uterus  B: Globular uterus | Clinical pregnancy rate | NR | NR | OR 0.85 (0.50–1.46) | NR |  |
|  |  |  | Ongoing pregnancy rate | NR | NR | OR 0.88 (0.53–1.49) | NR |  |
|  |  |  | Pregnancy loss rate | NR | NR | OR 1.01 (0.45–2.27) | NR |  |
|  |  |  | Biochemical pregnancy loss rate | NR | NR | OR 2.00 (0.95–4.21) | NR |  |
|  |  | A: No translesional vascularity  B: Translesional vascularity | Clinical pregnancy rate | NR | NR | OR 0.94 (0.52–1.69) | NR |  |
|  |  |  | Ongoing pregnancy rate | NR | NR | OR 0.79 (0.44–1.40) | NR |  |
|  |  |  | Pregnancy loss rate | NR | NR | OR 1.53 (0.67–3.50) | NR |  |
|  |  |  | Biochemical pregnancy loss rate | NR | NR | OR 1.59 (0.70–3.63) | NR |  |
|  |  | A: No irregular JZ  B: Irregular JZ | Clinical pregnancy rate | NR | NR | OR 1.53 (0.95–2.55) | NR |  |
|  |  |  | Ongoing pregnancy rate | NR | NR | OR 1.25 (0.78–1.99) | NR |  |
|  |  |  | Pregnancy loss rate | NR | NR | OR 1.25 (0.62–2.50) | NR |  |
|  |  |  | Biochemical pregnancy loss rate | NR | NR | OR 1.00 (0.48–2.08) | NR |  |
|  |  | A: No interrupted JZ  B: Interrupted JZ | Clinical pregnancy rate | NR | NR | OR 0.50 (0.28–0.88) | NR |  |
|  |  |  | Ongoing pregnancy rate | NR | NR | OR 0.52 (0.30–0.93) | NR |  |
|  |  |  | Pregnancy loss rate | NR | NR | OR 1.33 (0.55–3.24) | NR |  |
|  |  |  | Biochemical pregnancy loss rate | NR | NR | OR 2.02 (0.92–4.40) | NR |  |
|  |  |  | Clinical pregnancy rate | NR | NR | **aRR 0.75 (0.58–0.95)** | **0.02** | aRR = adjusted for age, AMH, number of embryos transferred, and PGT-A utilization. |
|  |  |  | Ongoing pregnancy rate | NR | NR | **aRR 0.72 (0.52–0.99)** | **0.049** |  |
|  |  |  | Pregnancy loss rate | NR | NR | aRR 1.21 (0.64–2.32) | 0.557 |  |
|  |  |  | Biochemical pregnancy loss rate | NR | NR | **aRR 1.98 (1.02–3.84)** | **0.044** |  |
|  |  | A: No focal adenomyoma  B: Focal adenomyoma | Clinical pregnancy rate | NR | NR | OR 1.63 (0.89–2.99) | NR | Not adjusted. |
|  |  |  | Ongoing pregnancy rate | NR | NR | OR 1.65 (0.94–2.88) | NR |  |
|  |  |  | Pregnancy loss rate | NR | NR | OR 0.71 (0.30–1.67) | NR |  |
|  |  |  | Biochemical pregnancy loss rate | NR | NR | OR 1.15 (0.50–2.69) | NR |  |
|  |  | A: Lower number of features  B: Higher number of features | Clinical pregnancy rate | NR | NR | OR 0.96 (0.80–1.15) | NR |  |
|  |  |  | Ongoing pregnancy rate | NR | NR | OR 0.94 (0.79–1.12) | NR |  |
|  |  |  | Pregnancy loss rate | NR | NR | OR 1.06 (0.83–1.36) | NR |  |
|  |  |  | Biochemical pregnancy loss rate | NR | NR | OR 1.07 (0.82–1.40) | NR |  |
| Wang et al. (2025)  Single-center retrospective cohort study | 537/537 (100) | A: No-AM  B: External AM | Implantation rate | 50.68% (A) vs 48.75% (B) | 0.563 | OR 0.93 (0.74–1.16) | 0.563 | Not adjusted. |
|  |  |  | Clinical pregnancy rate | 56.93% (A) vs 56.72% (B) | 1 | OR 0.99 (0.77–1.28) | 1 |  |
|  |  |  | Early pregnancy loss rate | 14.76% (A) vs 15.93% (B) | 0.707 | OR 1.09 (0.69–1.75) | 0.707 |  |
|  |  |  | Late pregnancy loss rate | 2.21% (A) vs 1.48% (B) | 0.752 | OR 0.66 (0.19–2.38) | 0.752 |  |
|  |  |  | Live birth rate | 46.85% (A) vs 45.80% (B) | 0.795 | OR 0.96 (0.74–1.24) | 0.795 |  |
|  |  | A: No-AM  B: Internal AM | Implantation rate | **44.77% (A) vs 36.23% (B)** | **0.043** | **OR 0.70 (0.50–0.99)** | **0.043** |  |
|  |  |  | Clinical pregnancy rate | **53.95% (A) vs 42.33% (B)** | **0.016** | **OR 0.63 (0.43–0.92)** | **0.016** |  |
|  |  |  | Early pregnancy loss rate | **14.66% (A) vs 27.47% (B)** | **0.023** | **OR 2.21 (1.11–4.40)** | **0.023** |  |
|  |  |  | Late pregnancy loss rate | 4.31% (A) vs 4.40% (B) | 1 | OR 1.02 (0.27–3.92) | 1 |  |
|  |  |  | Live birth rate | **42.33% (A) vs 27.44% (B)** | **0.002** | **OR 0.52 (0.34–0.77)** | **0.002** |  |
|  |  | A: No-AM  B: Mixed AM | Implantation rate | **48.05% (A) vs 28.94% (B)** | **<0.001** | **OR 0.44 (0.30–0.65)** | **<0.001** |  |
|  |  |  | Clinical pregnancy rate | **55.85% (A) vs 35.11% (B)** | **<0.001** | **OR 0.43 (0.28–0.65)** | **<0.001** |  |
|  |  |  | Early pregnancy loss rate | **19.05% (A) vs 40.91% (B)** | **0.002** | **OR 2.94 (1.47–5.87)** | **0.002** |  |
|  |  |  | Late pregnancy loss rate | 0.95% (A) vs 4.55% (B) | 0.3 | OR 4.95 (0.50–48.65) | 0.3 |  |
|  |  |  | Live birth rate | **44.15% (A) vs 18.09% (B)** | **<0.001** | **OR 0.28 (0.17–0.45)** | **<0.001** |  |
| Significant results are written in bold. | | | | | | | | |
| OR = odds ratio; RR = risk ratio; AM = adenomyosis; AFC = antral follicle count; AMH = anti-Müllerian hormone; BMI = body mass index; N/A = not available. | | | | | | | | |
| *only 5/8 MUSA criteria were assessed  **severity scored according to Lazzeri L, Morosetti G, Centini G, Monti G, Zupi E, Piccione E, et al. A sonographic classification of adenomyosis: interobserver reproducibility in the evaluation of type and degree of the myometrial involvement. Fertil Steril  2018;110:1154–61.e3. | | | | | | | | |

# Supplementary Table C. Studies excluded at full-text screening

*A total of 119 studies were excluded during full-text screening. Reasons for exclusion are listed below.*

| **No.** | **Study** | **Title** | **Journal** | **Reason for Exclusion** |
| --- | --- | --- | --- | --- |
| 1 | Abdel Wahed et al. (2023) | Sonographic findings in symptomatic patient with adenomyosis uteri | Egyptian Journal of Medical Research | Wrong patient population |
| 2 | Adesiyun et al. (2008) | Adenomyosis uteri in infertile women: experience in a tropical community teaching hospital | Pakistan Journal of Medical Sciences | No available data on only ultrasound |
| 3 | Alson (2024) | Endometriosis and adenomyosis diagnosed by ultrasonography. Aspects related to fertility treatment outcomes |  | Wrong study design |
| 4 | Alson et al. (2024) | OC11.04: Direct MUSA features of adenomyosis impact live birth rates after first in vitro fertilization / intracytoplasmic sperm injection treatment | Ultrasound in Obstet & Gyne | Only abstract available |
| 5 | Alson et al. (2024) | OC11.06: Indirect MUSA features of adenomyosis are associated with reduced chances of live birth after first in vitro fertilization treatment | Ultrasound in Obstet & Gyne | Only abstract available |
| 6 | Alson et al. (2024) | OP11.06: The predictive value of MUSA features of adenomyosis on live birth is poor, using a machine learning algorithm | Ultrasound in Obstet & Gyne | Only abstract available |
| 7 | Alson et al. (2025) | Cumulative live birth rates under three consecutive IVF/ICSI treatment cycles are reduced in women with endometriosis and/or adenomyosis diagnosed by ultrasonography. | Human Reproduction | Same cohort as previously included article. |
| 8 | Alson et al. (2026) | Machine learning prediction of live birth after IVF using the morphological uterus sonographic assessment group features of adenomyosis | Scientific Reports | Same cohort as previously included article. |
| 9 | Ansaripour et al. (2024) | Comparison of ultra-long gonadotropin releasing hormone agonist versus standard downregulation in women with adenomyosis undergoing frozen-thawed embryo transfer: a randomized clinical trial | Contracept. Reproductive Med. | No info on adenomyosis features |
| 10 | Arya et al. (2017) | Sonographic features of adenomyosis | Donald Sch. J. Ultrasound Obstet. Gynecol. | Wrong patient population |
| 11 | Atabekoğlu et al. (2020) | The association between adenomyosis and recurrent miscarriage | Eur. J. Obstet. Gynecol. Reprod. Biol. | Wrong study design |
| 12 | Benaglia et al. (2013) | Adenomyosis does not influence pregnancy rate in women undergoing IVF | Fertility and Sterility | Only abstract available |
| 13 | Beneduce et al. (2025) | P-473 Adenomyosis progression over time in patients with repeated pregnancy loss: ultrasound-based observations | Human Reproduction | Only abstract available |
| 14 | Bertucci et al. (2023) | How adenomyosis changes throughout pregnancy: A retrospective cohort study | Int. J. Gynecol. Obstet. | Wrong patient population |
| 15 | Bourdon et al. (2023) | Does the 'freeze all' strategy improve the chances of birth in the presence of adenomyosis? | Human Reproduction | Only abstract available |
| 16 | Bourdon et al. (2024) | The “freeze-all” strategy seems to improve the chances of birth in adenomyosis-affected women | Fertil. Steril. | No available data on only ultrasound |
| 17 | Brandt et al. (2025) | O-218 Symptoms and signs of adenomyosis according to the revised MUSA criteria in 375 women with pregnancy loss and known ploidy of the pregnancy loss | Human Reproduction | Only abstract available |
| 18 | Changmei et al. (2022) | Pregnancy outcomes in patients with adenomyosis with fertility requirements: retrospective analysis of clinical data from real world | Chinese J. Obstet. Gynecol. Pediatr. | Wrong language |
| 19 | Chen et al. (2025) | Adenomyosis Patterns on Transvaginal Sonography Could Predict the Obstetrical Outcomes of Fertility-Sparing Surgeries: A Retrospective Cohort Study | J. Minimally Invasive Gynecol. | Wrong patient population |
| 20 | Chen et al. (2026) | Impact of adenomyosis on pregnancy outcomes following frozen embryo transfer: development and validation of a single-center clinical predictive model | Frontiers in Endocrinology | No available data on only ultrasound |
| 21 | Chiang et al. (1999) | Effect of a sonographically diffusely enlarged uterus without distinct uterine masses on the outcome of in vitro fertilization-embryo transfer | JOURNAL OF ASSISTED REPRODUCTION AND GENETICS | No info on adenomyosis features |
| 22 | ChiCTR2300068940 (2023) | Effects of different time periods of GnRH-a pretreatment on cumulative live birth rate in IVF patients with adenomyosis with normal ovarian reserve: an RCT study |  | Protocol for RCT |
| 23 | Costello et al. (2011) | The effect of adenomyosis on in vitro fertilisation and intra-cytoplasmic sperm injection treatment outcome | Eur. J. Obstet. Gynecol. Reprod. Biol. | No info on adenomyosis features |
| 24 | Cozzolino et al. (2024) | IMPACT OF ADENOMYOSIS ON IVF CUMULATIVE LIVE BIRTH RATE: A RETROSPECTIVE COHORT STUDY | Fertility and Sterility | Only abstract available |
| 25 | CTIS2024-516089-12-00 (2024) | Assisted reproductive technology (ART) and pregnancy outcomes in women with adenomyosis (internal endometriosis) according to stimulation protocol in relation to immunological and endometrial features: a prospective, randomized study |  | Protocol for RCT |
| 26 | Dian et al. (2022) | Comparison of oocyte count, fertilization, and pregnancy rates in adenomyosis patients undergoing In Vitro Fertilization with short and long protocol controlled ovarian stimulation – Restospective study | Ann. Med. Surg. | Wrong patient population |
| 27 | Dinçer et al. (2024) | P-296 The relationship between serum HE4 and ultrasound findings of adenomyosis in infertility patients and its correlation with implantation and pregnancy outcomes in those undergoing IVF-ET | Human Reproduction | Only abstract available |
| 28 | Dinçer et al. (2024) | The relationship between serum HE4 and ultrasound findings of adenomyosis in infertility patients and its correlation with implantation and pregnancy outcomes in those undergoing IVF-ET | HUMAN REPRODUCTION | Only abstract available |
| 29 | Donadio et al. (2006) | Application of a levonorgestrel-releasing intrauterine device prior to in vitro fertilization cycles in women with adenomyosis | Rev. Bras. Ginecol. Obstet. | No info on adenomyosis features |
| 30 | Eisenberg et al. (2017) | Sonographic Signs of Adenomyosis Are Prevalent in Women Undergoing Surgery for Endometriosis and May Suggest a Higher Risk of Infertility | BioMed Res. Int. | Wrong patient population |
| 31 | EUCTR2019-001830-32-FR (2019) | Impact of ultra-long versus long down-regulation protocol on IVF/ICSI outcomes in adenomyosis |  | Protocol for RCT |
| 32 | EUCTR2021-005005-28-IT (2022) | Assisted reproductive technology (ART) and pregnancy outcomes in women with adenomyosis (internal endometriosis) according to stimulation protocol in relation to immunological and endometrial features: a prospective, randomized study |  | Protocol for RCT |
| 33 | Exacoustos et al. (2010) | Three dimensional sonographic evaluation of adenomyosis: The junctional zone features in infertile patients | Fertility and Sterility | Only abstract available |
| 34 | Exacoustos et al. (2020) | New Sonographic Classification of Adenomyosis: Do Type and Degree of Adenomyosis Correlate to Severity of Symptoms? | J. Minimally Invasive Gynecol. | Wrong patient population |
| 35 | Exacoustos et al. (2020) | IMPACT OF ULTRASOUND DIAGNOSIS OF ADENOMYOSIS ON PREGNANCY RATE AND OUTCOME IN IVF CYCLES WITH DONATED OOCYTES: A PROSPECTIVE STUDY. | FERTILITY AND STERILITY | Only abstract available |
| 36 | Exacoustos et al. (2021) | Ultrasound diagnosis of adenomyosis: impact on pregnancy rate in ivf cycles with donated oocytes | HUMAN REPRODUCTION | Only abstract available |
| 37 | Exacoustos et al. (2024) | Type and Location of Adenomyosis in Women with Recurrent Pregnancy Loss: A Transvaginal Ultrasonographic Assessment | Reprod. Sci. | Wrong study design |
| 38 | Fernandes et al. (2013) | The association of sonographically diagnosed adenomyosis on ovarian response and the outcome of in vitro fertilisation-embryo transfer | Fertility and Sterility | Only abstract available |
| 39 | Fernandes et al. (2017) | The association between sonographically diagnosed adenomyosis and in vitro fertilisation-embryo transfers | Fertility and Sterility | Only abstract available |
| 40 | Gokturk et al. (2022) | Effect on pregnancy outcomes of depot GnRH agonist pretreatment before frozen-thawed embryo transfer cycles in patients with adenomyosis | Human Reproduction | Only abstract available |
| 41 | Guo (2020) | CLINICAL OUTCOMES AFTER SINGLE VERSUS DOUBLE EMBRYO TRANSFERS IN WOMEN WITH ADENOMYOSIS: A RETROSPECTIVE STUDY | Fertility and Sterility | No info on adenomyosis features |
| 42 | Guo et al. (2016) | Pregnancy outcomes and neonatal outcomes after pituitary down-regulation in patients with adenomyosis receiving IVF/ICSI and FET: Results of a retrospective cohort study | Int. J. Clin. Exp. Med. | No info on adenomyosis features |
| 43 | Guo et al. (2021) | Clinical outcomes after single-versus double-embryo transfers in women with adenomyosis: a retrospective study | ARCHIVES OF GYNECOLOGY AND OBSTETRICS | No info on adenomyosis features |
| 44 | Guo et al. (2025) | Analysis of cumulative live birth rate outcomes of four ovarian stimulation protocols in Poseidon groups 3/4 patients with adenomyosis | JOURNAL OF ASSISTED REPRODUCTION AND GENETICS | No info on adenomyosis features |
| 45 | Harada et al. (2016) | The impact of adenomyosis on women's fertility | Obstetrical & gynecological survey | Wrong study design |
| 46 | Hashimoto et al. (2018) | Adenomyosis and adverse perinatal outcomes: increased risk of second trimester miscarriage, preeclampsia, and placental malposition | JOURNAL OF MATERNAL-FETAL & NEONATAL MEDICINE | No info on adenomyosis features |
| 47 | Hashimoto et al. (2018) | Diffuse type adenomyosis is associated with the high inci-dence of pregnancy complications | Journal of Obstetrics and Gynaecology Research | Article could not be found |
| 48 | Hou et al. (2020) | The effect of adenomyosis on IVF after long or ultra-long GnRH agonist treatment | Reproductive biomedicine online | No info on adenomyosis features |
| 49 | Huseynova (2022) | СOMPARATIVE CHARACTERISTICS OF CLINICAL AND DIAGNOSTIC SIGNS IN PATIENTS WITH ADENOMYOSIS AND REPRODUCTIVE DYSFUNCTION | Azerbaijan Med. J. | Wrong study design |
| 50 | Ishchenko et al. (2025) | EVALUATION OF THE COURSE OF CLINICAL SYMPTOMS AND REPRODUCTIVE OUTCOMES AFTER LASER DRILLING OF THE UTERUS WITH A HOLMIUM LASER IN PATIENTS OF REPRODUCTIVE AGE | Akusherstvo i Ginekologiya (Russian Federation) | Wrong language |
| 51 | Juárez-Barber et al. (2023) | Adjustment of progesterone administration after endometrial transcriptomic analysis does not improve reproductive outcomes in women with adenomyosis | Reprod. BioMed. Online | No info on adenomyosis features |
| 52 | Kadam et al. (2025) | How Reproducible Are the Ultrasound Features of Adenomyosis Defined by the Revised MUSA Consensus? | J. Clin. Med. | Wrong study design |
| 53 | Kahraman et al. (2026) | Effect of endometriosis and adenomyosis on oocyte quality: an evaluation of 205,978 oocytes | Reproductive BioMedicine Online | No info on adenomyosis features |
| 54 | Ke et al. (2025) | Endometrial thickness/volume and uterine peristalsis in adenomyosis patients undergoing frozen embryo transfer to predict pregnancy outcomes: A retrospective study | MEDICINE | No info on adenomyosis features |
| 55 | Khan et al. (2012) | Does adenomyosis impair implantation? A cohort study | Fertility and Sterility | Only abstract available |
| 56 | Krasnopolskaya et al. (2013) | IVF infertility treatment outcomes are negatively influenced by the severity of diffuse adenomyosis established by ultrasound examination | HUMAN REPRODUCTION | Article could not be found |
| 57 | Latif et al. (2022) | The impact of adenomyosis and co-existing endometriosis on IVF outcomes | BJOG: An International Journal of Obstetrics and Gynaecology | Only abstract available |
| 58 | Li et al. (2023) | Effects of duration of long-acting GnRH agonist downregulation on assisted reproductive technology outcomes in patients with adenomyosis: a retrospective cohort study | Front. Med. | No info on adenomyosis features |
| 59 | Liang et al. (2019) | Effect of pretreatment with a levonorgestrel-releasing intrauterine system on IVF and vitrified–warmed embryo transfer outcomes in women with adenomyosis | Reprod. BioMed. Online | No info on adenomyosis features |
| 60 | Liang et al. (2022) | Reproductive outcomes of in vitro fertilization and fresh embryo transfer in infertile women with adenomyosis: a retrospective cohort study | Frontiers in Endocrinology | No info on adenomyosis features |
| 61 | Liu et al. (2025) | Clinical features and perinatal outcomes in women with adenomyosis | European Journal of Obstetrics and Gynecology and Reproductive Biology | No available data on only ultrasound |
| 62 | Liu et al. (2025) | Peri-implantation treatment with TNF-α inhibitor for endometriosis and/or adenomyosis women undergoing frozen-thawed embryo transfer: A retrospective cohort study | J. Reprod. Immunol. | Wrong comparator |
| 63 | Liu et al. (2025) | Peri-implantation treatment with TNF-alpha inhibitor for endometriosis and/or adenomyosis women undergoing frozen-thawed embryo transfer: A retrospective cohort study | Journal of Reproductive Immunology | No info on adenomyosis features |
| 64 | Lu et al. (2023) | Three-dimensional ultrasound combined with three-dimensional power Doppler ultrasound evaluation of endometrial-myometrial interface in infertility patients with adenomyosis for predicting pregnancy outcome of in vitro fertilization-embryo transfer | Chin. J. Intervent. Imaging Ther. | Wrong language |
| 65 | Maged et al. (2017) | 3D ultrasound assessment of endometrial junctional zone anatomy as a predictor of the outcome of ICSI cycles | EUROPEAN JOURNAL OF OBSTETRICS & GYNECOLOGY AND REPRODUCTIVE BIOLOGY | Wrong patient population |
| 66 | Marques et al. (2021) | Association of 2d and 3d transvaginal ultrasound findings with adenomyosis in symptomatic women of reproductive age: A prospective study | Clinics | Wrong patient population |
| 67 | Mattei (2026) | Impatto della stimolazione ovarica sulla dimensione degli endometriomi e sulle variabili ecografiche dell'adenomiosi durante l'IVF |  | Only abstract available |
| 68 | McCormack et al. (2016) | 3D ultrasound findings in women attending a South Australian recurrent miscarriage clinic. | Australasian journal of ultrasound in medicine | No info on adenomyosis features |
| 69 | Messelt et al. (2024) | MILD ADENOMYOSIS DOES NOT IMPACT PREGNANCY RATE OR PREGNANCY OUTCOMES IN SINGLE EUPLOID FROZEN EMBRYO TRANSFERS | Fertility and Sterility | Only abstract available |
| 70 | Mijatovic et al. (2010) | Adenomyosis has no adverse effects on IVF/ICSI outcomes in women with endometriosis treated with long-term pituitary down-regulation before IVF/ICSI | Eur. J. Obstet. Gynecol. Reprod. Biol. | No info on adenomyosis features |
| 71 | Moayed et al. (2023) | Pregnancy outcomes in women with adenomyosis, undergoing artificial endometrial preparation with and without gonadotropin-releasing hormone agonist pretreatment in frozen embryo transfer cycles: An RCT | Int. J. Reprod. Biomed. | Wrong study design |
| 72 | Moliner et al. (2016) | New classification of adenomyosis | Human Fertility | Only abstract available |
| 73 | Nagireddy et al. (2021) | Adenomyosis is Associated with Sub-Optimal Outcomes in Fresh Embryo | JOURNAL OF RESEARCH IN MEDICAL AND DENTAL SCIENCE | No info on adenomyosis features |
| 74 | NCT03946722 (2019) | Modified Downregulation for Women With Adenomyosis of the Uterus Prior to Frozen-thawed Embryo Transfer |  | Protocol for RCT |
| 75 | NCT04878224 (2021) | Junctional Zone Evaluation by 3D Ultrasound and Its Relation to Clinical Outcomes in Assisted Reproductive Technology | clinicaltrials.gov | Protocol for RCT |
| 76 | NCT05418140 (2022) | Adenomyosis and Pregnancy Outcomes in Women Undergoing Assisted Reproductive Technology Treatment: a Prospective Multicentre Cohort Study (AdAPT-ART) | clinicaltrials.gov | Protocol for RCT |
| 77 | NCT05937490 (2023) | Assisted Reproductive Technology (ART) and Pregnancy Outcomes in Women With Adenomyosis (Internal Endometriosis) According to Stimulation Protocol in Relation to Immunological and Endometrial Features: a Prospective, Randomized Study | clinicaltrials.gov | Protocol for RCT |
| 78 | NCT06164015 (2023) | Association of Acquired Uterine Abnormalities With Recurrent Miscarriage | clinicaltrials.gov | Protocol for RCT |
| 79 | Neal et al. (2019) | THREE-DIMENSIONAL ULTRASOUND DIAGNOSIS OF ADENOMYOSIS IS NOT ASSOCIATED WITH DIMINISHED LIVE BIRTH FOLLOWING SINGLE THAWED EUPLOID BLASTOCYST TRANSFER: A PROSPECTIVE COHORT STUDY | Fertility and Sterility | Only abstract available |
| 80 | Niu et al. (2013) | Long-term pituitary downregulation before frozen embryo transfer could improve pregnancy outcomes in women with adenomyosis | Gynecol. Endocrinol. | No info on adenomyosis features |
| 81 | O'Connor et al. (2016) | Outcomes for in vitro fertilization in uterine adenomyosis: A retrospective cohort study | Fertility and Sterility | Only abstract available |
| 82 | Onofriescu et al. (2019) | Transvaginal sonography for the diagnosis of adenomyosis determines the choice of the IVF protocol | European Journal of Obstetrics Gynecology and Reproductive Biology | Only abstract available |
| 83 | Pacu et al. (2025) | Effect of Treatment with Progestins and Antiplatelet Agents on IVF in Women with Adenomyosis and Recurrent Implantation Failure | Diagn. | No info on adenomyosis features |
| 84 | Puente et al. (2016) | Adenomyosis in infertile women: Prevalence and the role of 3D ultrasound as a marker of severity of the disease | Reprod. Biol. Endocrinol. | No info on adenomyosis features |
| 85 | PuenteAgueda et al. (2014) | High prevalence of adenomyosis in recurrent pregnancy loss and previous ART failure | Human Reproduction | No info on adenomyosis features |
| 86 | Qi et al. (2026) | Obstetric and neonatal outcomes in women with internal and non-internal adenomyosis. | Reproductive Biomedicine Online | Wrong patient population |
| 87 | Rao et al. (2018) | Adenomyosis, art and reproductive outcome | International Journal of Gynecology and Obstetrics | Only abstract available |
| 88 | Sachs-Guedj et al. (2023) | Presence of Adenomyosis Impairs Clinical Outcomes in Women Undergoing Frozen Embryo Transfer: A Retrospective Cohort Study | J. Clin. Med. | No info on adenomyosis features |
| 89 | Salim et al. (2012) | Adenomyosis reduces pregnancy rates in infertile women undergoing IVF | Reprod. BioMed. Online | No info on adenomyosis features |
| 90 | Sbracia et al. (2018) | A CONTROLLED TRIAL ON UTERINE ADENOMYOSIS TREATMENT COMPARING AROMATASE INHIBITOR PLUS GNRH ANALOGUE VERSUS DIENOGEST IN WOMEN UNDERGOING IVF |  | Only abstract available |
| 91 | Sharma et al. (2015) | Does presence of adenomyosis effect reproductive outcome-a retrospective analysis of 607 patients having endometriosis, endometriosis with adenomyosis and only adenomyosis who underwent IVF/ICSI | Fertility and Sterility | Only abstract available |
| 92 | Sharma et al. (2023) | Low-dose letrozole – an effective option for women with symptomatic adenomyosis awaiting IVF: a pilot randomized controlled trial | Reprod. BioMed. Online | Wrong patient population |
| 93 | Sharma et al. (2023) | Impact of localization of diffuse adenomyosis on reproductive outcomes and pregnancy complications: A prospective cohort study of 585 patients after frozen embryo replacement cycle | Human Reproduction | Only abstract available |
| 94 | Shi et al. (2023) | Effects of localization of uterine adenomyosis on clinical features and pregnancy outcome | Sci. Rep. | Wrong patient population |
| 95 | Shunji et al. (2025) | Focal Adenomyosis and Uterine Junctional Zone Abnormalities: Predictors of Live Birth Rates in Women with Infertility and Adenomyosis Undergoing Assisted Reproduction Technologies |  | No available data on only ultrasound |
| 96 | Silva et al. (2020) | Junctional Zone in Infertile Women: A Three-dimensional Ultrasound Study | REVISTA BRASILEIRA DE GINECOLOGIA E OBSTETRICIA | No info on adenomyosis features |
| 97 | Stanekova et al. (2018) | The rate of euploid miscarriage is increased in the setting of adenomyosis | Hum. Reprod. Open | No available data on only ultrasound |
| 98 | Stonberg et al. (2026) | Adenomyosis Identified On Initial Saline Sonography In Patients Undergoing In -Vitro Fertilization Is Associated With Lower Live Birth Rates and Cumulative Live Birth Rates When Compared to Controls | REPRODUCTIVE SCIENCES | Article could not be found |
| 99 | Sudhakar et al. (2022) | Does Adenomyosis Influence ICSI Clinical Outcome? A Systematic Analysis and Impact of GnRH Agonist Pretreatment for Women with Adenomyosis in ICSI-FET Cycle: A Retrospective Cohort Study | JOURNAL OF OBSTETRICS AND GYNECOLOGY OF INDIA | No info on adenomyosis features |
| 100 | Tandulwadkar et al. (2024) | Proposed classification of adenomyosis in Infertile women to simplify management options undergoing ART | J. Bras. Reproducao Assistida | No info on adenomyosis features |
| 101 | Tao et al. (2015) | Effects of uterine adenomyosis on clinical outcomes of infertility patients treated with in vitro fertilization/intracytoplasmic sperm injection-embryo transfer (IVF/ICSI-ET) | Nan Fang Yi Ke Da Xue Xue Bao | Wrong language |
| 102 | Thalluri et al. (2012) | Ultrasound diagnosed adenomyosis has a negative impact on successful implantation following GnRH antagonist IVF treatment | Hum. Reprod. | No info on adenomyosis features |
| 103 | Trinchant et al. (2021) | Uterine adenomyosis does not affect perinatal outcomes in ART treatments | Human Reproduction | Only abstract available |
| 104 | Valdés-Bango et al. (2024) | Internal and external adenomyosis phenotypes: ultrasound features and association with clinical outcomes | Hum. Reprod. | Wrong patient population |
| 105 | Vasiliu (2016) | The link between adenomyosis and infertility - From theory to practical issues | Gynecological Endocrinology | Only abstract available |
| 106 | Viana et al. (2024) | Adenomyosis and your implications on fertility and on the results of assisted reproduction techniques (ART) |  | Wrong study design |
| 107 | Wang et al. (2023) | Impact of adenomyosis and endometriosis on IVF/ICSI pregnancy outcome in patients undergoing gonadotropin-releasing hormone agonist treatment and frozen embryo transfer | SCIENTIFIC REPORTS | No info on adenomyosis features |
| 108 | Wei et al. (2023) | Comparison of pregnancy outcomes in infertile patients with different types of adenomyosis treated with high-intensity focused ultrasound | Int. J. Hyperthermia | No available data on only ultrasound |
| 109 | Wu et al. (2022) | Long-term GnRH agonist pretreatment before frozen embryo transfer improves pregnancy outcomes in women with adenomyosis | Reprod. BioMed. Online | No info on adenomyosis features |
| 110 | Xiong et al. (2023) | Pregnancy outcomes of adenomyotic patients with primary infertility after high-intensity focused ultrasound treatment | Int. J. Hyperthermia | No info on adenomyosis features |
| 111 | Yan et al. (2014) | Effect of adenomyosis on in vitro fertilization/intracytoplasmic sperm injection outcomes in infertile women: A retrospective cohort study | Gynecol. Obstet. Invest. | No info on adenomyosis features |
| 112 | Yoldemir (2020) | Adenomyosis and fertility outcomes | Gynecol. Endocrinol. | Wrong study design |
| 113 | Youm et al. (2011) | In vitro fertilization and embryo transfer outcomes in relation to myometrial thickness | J. Assisted Reprod. Genet. | No info on adenomyosis features |
| 114 | Zhang et al. (2021) | Pregnancy outcomes of infertile women with ultrasound-diagnosed adenomyosis for in vitro fertilization and frozen–thawed embryo transfer | Arch. Gynecol. Obstet. | No info on adenomyosis features |
| 115 | Zhang et al. (2021) | Impact of Uterine Adenomyosis on Pregnancy Outcomes in Women Undergoing In Vitro Fertilization Treated With a Long-Term Pituitary Downregulation Protocol | Front. Endocrinol. | No info on adenomyosis features |
| 116 | Zhang et al. (2022) | Comparison of pregnancy outcomes between GnRH antagonist protocol with freeze-all strategy and long-acting GnRH agonist protocol in women with adenomyosis undergoing IVF/ICSI: a propensity-score matching analysis | BMC Pregnancy Childbirth | No info on adenomyosis features |
| 117 | Zhang et al. (2022) | Effect of GnRH-a pretreatment before frozen-thawed embryo transfer on pregnancy outcome of adenomyosis-associated infertile patients with 56 cm3<= uterine volume <=100 cm3 | Annals of Translational Medicine | No info on adenomyosis features |
| 118 | Zhou et al. (2016) | Pregnancy outcomes and symptom improvement of patients with adenomyosis treated with high intensity focused ultrasound ablation | Zhonghua Fu Chan Ke Za Zhi | Wrong language |
| 119 | Zhu et al. (2025) | Pregnancy outcomes and influencing factors following high-intensity focused ultrasound treatment for adenomyosis: a single-center retrospective study | International Journal of Hyperthermia | No available data on only ultrasound |

# Supplementary Table D. Risk of Bias - Newcastle Ottawa scale

| Author (year) | The exposed cohort | Non exposed cohort | Ascertainment of exposure | Outcome of interest was not present at start of study | Comparability of cohorts | | Assessment of outcome | Was follow-up long enough? | Adequacy of follow up of cohorts | Total: |
| --- | --- | --- | --- | --- | --- | --- | --- | --- | --- | --- |
|  |  |  |  |  | Main factor (age) | Additional factor (BMI, S-AMH, Parity) |  |  |  |  |
| Li X et al. (2021) | * | * | * | 0 | * | * | * | * | * | 8/9 |
| Sharma et al. (2025) | * | * | * | * | 0 | 0 | * | * | * | 7/9 |
| Zhang et al. (2023) | * | * | * | 0 | * | * | * | * | 0 | 7/9 |
| Sharma et al. (2019) | * | * | * | 0 | 0 | 0 | * | * | * | 6/9 |
| Benaglia et al. (2014) | * | * | * | * | 0 | 0 | * | * | * | 7/9 |
| Park et al. (2016) | * | * | * | 0 | 0 | 0 | * | * | * | 6/9 |
| Mavrelos et al. (2017) | * | * | * | * | * | * | * | * | * | 9/9 |
| Higgins et al. (2021) | * | * | * | * | * | * | * | * | * | 9/9 |
| Neal et al. (2020) | 0 | * | * | * | * | * | * | * | * | 8/9 |
| Trinchant et al. (2022) | 0 | * | * | 0 | 0 | 0 | * | * | * | 5/9 |
| Han B et al. (2023) | * | * | * | 0 | * | * | * | * | * | 8/9 |
| Cozzolino et al. (2024) | * | * | * | * | 0 | 0 | * | * | * | 7/9 |
| Dason et al. (2023) | 0 | * | * | 0 | * | * | * | * | * | 7/9 |
| Trinchant et al. (2025) | 0 | * | * | 0 | * | * | * | * | * | 7/9 |
| Alson et al. (2024a) | * | * | * | * | * | * | * | * | * | 9/9 |
| Arlıer et al. (2025) | * | * | * | 0 | 0 | 0 | * | * | * | 6/9 |
| Etrusco et al. (2026) | * | * | * | * | * | * | * | * | * | 9/9 |
| Wang et al. (2025) | * | * | * | * | 0 | 0 | * | * | * | 7/9 |
| Benlioglu et al. (2026) | * | * | * | * | 0 | 0 | * | * | * | 7/9 |
| Han SJ et al. (2026) | * | * | * | * | * | * | * | * | 0 | 8/9 |
| Li Z et al. (2025) | * | * | * | * | * | * | * | * | * | 9/9 |
| Li J et al. (2025) | 0 | * | * | * | * | * | * | * | * | 8/9 |

# Supplementary Table E. Risk of Bias - Joan Briggs Institute

| Author (year) | Appropriate sample frame? | Study participants recruited in an appropriate way? | Sample size adequate? | Subjects, setting described in detail? | Coverage bias? | Valid methods used for identification of AM? | Condition measured in a standard, reliable way? | Appropriate statistical analysis? | Response rate adequate? | Total |
| --- | --- | --- | --- | --- | --- | --- | --- | --- | --- | --- |
| Mavrelos et al. (201) | Yes | Unclear | Yes | Yes | NA | Yes | Yes | Yes | Yes | 7/8 |
| AbuHashim et al. (2020) | Yes | Yes | No | Yes | NA | Yes | Yes | Yes | Yes | 7/8 |
| Higgins et al. (2021) | Unclear | Yes | Yes | Yes | NA | Yes | Unclear | No | Yes | 5/8 |
| Neal et al. (2020) | Yes | Yes | No | Yes | NA | Yes | Yes | No | Yes | 6/8 |
| Han B et al. (2023) | Yes | Unclear | Yes | Yes | NA | Yes | No | No | Unclear | 4/8 |
| Dason et al. (2023) | No | Unclear | No | Yes | NA | Yes | No | Yes | Unclear | 3/8 |
| Alson et al. (2024b) | Yes | Yes | Yes | Yes | NA | Yes | Yes | Yes | Yes | 8/8 |
| Busnelli et al. (2024) | Yes | Yes | Yes | Yes | NA | Yes | Yes | Yes | Yes | 8/8 |
| Arlıer et al. (2025) | No | Unclear | Yes | No | NA | Yes | Unclear | Unclear | Unclear | 2/8 |
| Berbecaru et al. (2025) | No | Unclear | Yes | No | NA | Yes | No | No | Unclear | 2/8 |
| Tokhunts et al. (2026) | Unclear | Unclear | No | Yes | NA | Unclear | No | No | Yes | 2/8 |
| Madani et al. (2026) | No | Yes | No | Yes | NA | Yes | Yes | No | Yes | 5/8 |
| Surcel et al. (2026) | No | Unclear | No | No | NA | Yes | Unclear | No | Unclear | 1/8 |
| Han SJ et al. (2026) | No | Yes | Yes | Yes | NA | Yes | Unclear | No | Yes | 5/8 |
| Yes = 1 point, Unclear/No = 0 points. 0-3 points = high risk of bias, 4-6 points = moderate risk of bias, 7-8 points = low risk of bias | | | | | | | | | | |

# Supplementary Table F. GRADE assessment

| **Comparison** | **Outcome** | **No. of studies (participants)** | **Effect estimate** | **Risk of bias** | **Inconsistency** | **Indirectness** | **Imprecision** | **Publication bias** | **Certainty** |
| --- | --- | --- | --- | --- | --- | --- | --- | --- | --- |
| **Diffuse vs focal AM** | | | | | | | | | |
|  | Clinical pregnancy | 5 studies (n=1750) | OR 0.92 (95% CI 0.56–1.50) | Not serious | Serious (a) | Very serious (bc) | Very serious (b) | Not serious | ⨁◯◯◯ Very low |
|  | Live birth | 5 studies (n=1750) | OR 0.95 (95% CI 0.40 - 2.29) | Not serious | Very serious (d) | Very serious (bc) | Very serious (b) | Not serious | ⨁◯◯◯ Very low |
|  | Pregnancy loss | 6 studies (n=752) | OR 1.15 (95% CI 0.51 - 2.63) | Not serious | Serious (ab) | Very serious (bc) | Very serious (b) | Not serious | ⨁◯◯◯ Very low |
| **Focal vs no AM** | | | | | | | | | |
|  | Clinical pregnancy | 4 studies (n=4752) | OR 0.70 (95% CI 0.25-1.96) | Not serious | Serious (bc) | Very serious (bc) | Very serious (b) | Not serious | ⨁◯◯◯ Very low |
|  | Live birth | 4 studies (n=4752) | OR 0.46 (95% CI 0.17-1.27) | Not serious | Serious (bc) | Very serious (bc) | Very serious (b) | Not serious | ⨁◯◯◯ Very low |
|  | Pregnancy loss | 5 studies (n=2687) | OR 2.40 (95% CI 0.75 - 7.70) | Not serious | Serious (bc) | Very serious (bc) | Serious (a) | Not serious | ⨁◯◯◯ Very low |
| **Diffuse vs no AM** | | | | | | | | | |
|  | Clinical pregnancy | 5 studies (n=5283) | OR 0.67 (95% CI 0.45-1.00) | Not serious | Serious (bc) | Very serious (bc) | Serious (a) | Not serious | ⨁◯◯◯ Very low |
|  | Live birth | 5 studies (n=5283) | OR 0.52 (95% CI 0.34-0.79) | Not serious | Serious (b) | Very serious (bc) | Serious (a) | Not serious | ⨁◯◯◯ Very low |
|  | Pregnancy loss | 6 studies (n=2851) | OR 2.25 (95% CI 0.93-5.43) | Not serious | Serious (bc) | Very serious (bc) | Serious (a) | Not serious | ⨁◯◯◯ Very low |
| **High vs low uterine volume** | | | | | | | | | |
|  | Clinical pregnancy | 3 studies (n=2083) | OR 0.93 (95% CI 0.86-1.01) | Not serious | Not serious | Serious (a) | Not serious | Not serious | ⨁⨁⨁◯ Mode |
|  | Live birth | 3 studies (n=2083) | OR 0.67 (95% CI 0.42-1.07) | Not serious | Not serious | Serious (a) | Serious (a) | Not serious | ⨁⨁◯◯ Low |
|  | Pregnancy loss | 3 studies (n=821) | OR 2.54 (95% CI 0.51-12.51) | Not serious | Not serious | Serious (a) | Very serious (b) | Not serious | ⨁◯◯◯ Very low |
| **Internal AM vs no AM** | | | | | | | | | |
|  | Clinical pregnancy | 3 studies (n=1088) | OR 0.62 (95% CI 0.48-0.81) | Not serious | Not serious | Very serious (ac) | Serious (a) | Not serious | ⨁◯◯◯ Very low |
|  | Live birth | 3 studies (n=1088) | OR 0.49 (95% CI 0.42-0.57) | Not serious | Not serious | Very serious (ac) | Not serious | Not serious | ⨁⨁◯◯ Low |
|  | Pregnancy loss | 3 studies (n=502) | OR 2.67 (95% CI 1.07-6.70) | Not serious | Not serious | Very serious (ac) | Serious (a) | Not serious | ⨁◯◯◯ Very low |
| **Internal AM vs external AM** | | | | | | | | | |
|  | Clinical pregnancy | 2 studies (n=1059) | OR 0.50 (95% CI 0.08-3.25) | Not serious | Not serious | Serious (a) | Very serious (b) | Not serious | ⨁◯◯◯ Very low |
|  | Live birth | 2 studies (n=1059) | OR 0.48 (95% CI 0.12 - 1.95) | Not serious | Not serious | Serious (a) | Very serious (b) | Not serious | ⨁◯◯◯ Very low |
|  | Pregnancy loss | 2 studies (n=479) | OR 1.75 (95% CI 0.03-100.59) | Not serious | Not serious | Serious (a) | Very serious (b) | Not serious | ⨁◯◯◯ Very low |
| **External AM vs no AM** | | | | | | | | | |
|  | Clinical pregnancy | 2 studies (n=1075) | OR 1.21 (95% CI 0.34-4.26) | Not serious | Not serious | Serious (a) | Very serious (b) | Not serious | ⨁◯◯◯ Very low |
|  | Live birth | 2 studies (n=1075) | OR 1.00 (95% CI 0.11-8.85) | Not serious | Serious (a) | Serious (a) | Very serious (b) | Not serious | ⨁◯◯◯ Very low |
|  | Pregnancy loss | 2 studies (n=533) | OR 1.43 (95% CI 0.00-1245.26) | Not serious | Serious (ab) | Serious (a) | Very serious (b) | Not serious | ⨁◯◯◯ Very low |

Legend: AM (adenomyosis), OR (odds ratio).

**Inconsistency**

We assessed inconsistency through visual inspection of the forest plots and statistical heterogeneity quantified using I². Visual assessment considered the variability and direction of the point estimates and the degree of overlap between confidence intervals. Certainty was downgraded by one level in the presence of substantial heterogeneity (I² 50–74.9%) and by two levels in the presence of considerable heterogeneity (I² 75–100%). Subgroup analyses stratified by study design (prospective vs. retrospective) were performed to explore whether differences in study design could explain the observed heterogeneity. When the subgroup analysis provided a plausible explanation for the observed heterogeneity, we did not downgrade the certainty of evidence for inconsistency.

| One-level downgrades | |  |
| --- | --- | --- |
| a | Downgraded one level due to inconsistent effect estimates across studies. | or |
| b | Downgraded one level due to substantial heterogeneity (I²=>50%). | or |
| c | Downgraded one level due to wide variation in point estimates, with heterogeneity partly explained by differences in diagnostic criteria, study design, or intervention. | or |
| Two-level downgrades | |  |
| d | Downgraded two levels due to considerable, unexplained heterogeneity (I² ≥75%). | or |
| e | Downgraded two levels due to conflicting results across studies with minimal confidence interval overlap. | or |

**Indirectness**

We assessed indirectness by evaluating the applicability of the exposure, comparator, population, and outcome definitions across studies to the prespecified PICO question. Certainty was downgraded by one level when diagnostic criteria, comparator definitions, or outcome measures differed meaningfully across studies in ways that could affect the applicability of the evidence to the review question, and by two levels when important differences affected multiple PICO elements or substantially limited the applicability of the evidence. Certainty was not downgraded when differences in definitions were considered unlikely to affect the applicability or interpretation of the effect estimates.

|  | One-level downgrades | or |
| --- | --- | --- |
| a | Downgraded one level due to variation in exposure or comparator definition (e.g., diagnostic modality or group classification) across studies. | or |
| b | Downgraded one level due to partial reliance on surrogate outcomes not fully representative of the outcome of interest. | or |
| Two-level downgrades | |  |
| c | Downgraded two levels due to substantial variation across multiple PICO elements (exposure, comparator, and outcome definitions) between studies. | or |

**Imprecision**

As no established minimally important effect threshold exists for associations between ultrasound features of adenomyosis and reproductive outcomes in regards to rating imprecision, a clinical important threshold was prespecified as an odds ratio above 1.25 or below 0.75. If the confidence interval crossed the threshold, the certainty of evidence was downgraded by one level. If it crossed both thresholds, the certainty was downgraded by two levels.
